# Computational models predicting the early development of the COVID-19 pandemic in Sweden: systematic review, data synthesis, and secondary validation of accuracy

**DOI:** 10.1038/s41598-022-16159-6

**Published:** 2022-08-02

**Authors:** Philip Gerlee, Anna Jöud, Armin Spreco, Toomas Timpka

**Affiliations:** 1 Mathematical Sciences, Chalmers University of Technology, Gothenburg, Sweden; 2 Mathematical Sciences, University of Gothenburg, Gothenburg, Sweden; 3 Department of Laboratory Medicine, Lund University, Lund, Sweden; 4 Department of Research and Development, Skåne University Hospital, Lund, Sweden; 5 Department of Health, Medicine and Caring Sciences, Linköping University, Linköping, Sweden; 6 Regional Executive Office, Region Östergötland, Linköping, Sweden

**Keywords:** Epidemiology, Public health

## Abstract

Computational models for predicting the early course of the COVID-19 pandemic played a central role in policy-making at regional and national levels. We performed a systematic review, data synthesis, and secondary validation of studies that reported on prediction models addressing the early stages of the COVID-19 pandemic in Sweden. A literature search in January 2021 based on the search triangle model identified 1672 peer-reviewed articles, preprints and reports. After applying inclusion criteria 52 studies remained out of which 12 passed a Risk of Bias Opinion Tool. When comparing model predictions with actual outcomes only 4 studies exhibited an acceptable forecast (mean absolute percentage error, MAPE < 20%). Models that predicted disease incidence could not be assessed due to the lack of reliable data during 2020. Drawing conclusions about the accuracy of the models with acceptable methodological quality was challenging because some models were published before the time period for the prediction, while other models were published during the prediction period or even afterwards. We conclude that the forecasting models involving Sweden developed during the early stages of the COVID-19 pandemic in 2020 had limited accuracy. The knowledge attained in this study can be used to improve the preparedness for coming pandemics.

## Introduction

Less than 2 months—this is the typical time frame from the discovery of a new virus with pandemic potential to global spread of the virus at an exponential rate^1,2^. During this initial phase of the pandemic computational models can provide important information for early response policies and interventions at national and regional levels. However, lack of knowledge about the biological nature and origin of the infectious agent, its transmission routes, and the efficacy of interventions during the early phases of a pandemic poses difficulties for infectious disease modelers^3^.

SARS-CoV-2 reached Sweden in February 2020, and the first wave of hospital admissions due to COVID-19 reached its peak in May 2020 and a second wave appeared at the end of the year. To limit the spread of the virus, the Public Health Authority of Sweden (PHAS) recommended, unlike their counterparts in many other countries, voluntary social distancing and self-quarantining rather than enforcing a strict legal lockdown. To guide their policy-decisions and recommendations PHAS used computational forecasting models during the early pandemic stages and have since November 2020 been commissioned by the Swedish government to continually produce scenarios for the remainder of the pandemic.

Because most nations use computational forecasting models in their policy-making during the early phases of pandemics, it is important to clarify the validity and accuracy of these models in relation to the factual pandemic development during the initial pandemic stages. By increased knowledge of the virus, e.g. the discovery that a large fraction of cases are asymptomatic and their contribution to disease transmission^3^, and a better understanding of transmission routes^4^, the early models developed during the beginning of the pandemic become obsolete. Despite their drawbacks compared to later models, it is of importance to study these early efforts in order to learn about their validity and prediction accuracy^5^, since this knowledge will improve our preparedness for coming pandemics.

With this background, we set out to evaluate forecasting models involving Sweden published early during the COVID-19 pandemic. The specific aim was to evaluate COVID-19 forecasting models published in 2020 involving the Swedish population with regards to their prediction accuracy. To date, no systematic review of such models has been reported, a gap we intend to fill with this study.

## Methods

The study was conducted as a systematic review of published literature followed by a data synthesis^6,7^. For this purpose, searches were carried out for scientific publications (scientifically reviewed before publication), preprints (i.e. articles of a scientific nature that are published openly without prior review) and the gray literature (i.e. reports and documents published by organizations and authorities). The study protocol is registered in the database for structured literature syntheses and meta-analyzes PROSPERO (International prospective register of systematic reviews) no. CRD42021229514 (see Supplement S1).

### Database searches

The literature searches were based on the search triangle model^6^. Systematic searches were conducted between 22 January 2021 and 29 January 2021 of databases (PubMed, Cochrane Library, Embase, Love platform/Epistemikos), containing peer-reviewed scientific publications and systematic reviews in areas relevant to the review issue, exploratory searches were performed ﻿in preprint archives, while look-up searches were performed in the gray literature. The literature searches were reported according to the PRISMA-S protocol (see Supplement S2).

The systematic search (keywords: prediction, nowcast, forecast, simulation model, model, modeling, estimation, scenario, surveillance, Epidemiology, COVID-19, SARS-cov-2, swed*) of the collegially assessed scientific literature had the goal to identify all relevant publications (within the criteria of the study) in a transparent and reproducible manner.

The explorative searches in the preprint archives were initiated by asking a preliminary question via a tool specifically designed for searches in these archives (search.biopreprint) and then reviewing the recovered records. Thereafter, the searches were repeated iteratively until adjustments no longer led to significant changes in the set of identified preprints. A separate supplementary search was performed against the two largest preprint databases bioRxiv (which also includes preprints from medRxiv) and arXiv. Finally, a search (directed search) of the gray literature was performed. The search—also called search for known documents—was carried out with the aim of obtaining documents from the websites of relevant Swedish and international authorities active in the area: PHAS, the National Board of Health and Welfare, the Swedish Civil Contingencies Agency and the European Center for Disease Prevention and Control (ECDC). Local and regionally produced forecast data in different healthcare regions are not included in this report. These are regarded as internal working material since they are not published and not publicly available.

### Inclusion criteria

- scientific articles that report epidemiological results regarding actual or scenario-based predictions of morbidity, mortality, or healthcare burden caused by COVID-19 in Sweden or parts of Sweden in 2020.
- reports of COVID-19 modelling published by the PHAS.

### Exclusion criteria

- non-original analyzes (e.g. reviews, perspective articles, editorials, recommendations and guidelines).
- duplicate studies.
- in silico studies (pure simulations without comparison with data).
- descriptive epidemiological publications (e.g. description of case incidences and geographical distributions).
- models that only examine the effect of interventions (rather than predicting risk or disease burden).
- articles or reports that present new mathematical models or software tools, unless an explicit central purpose of the study is to predict COVID-19 phenomena.
- articles or reports from which predictions could not be extracted as a time series.
- articles or reports that present predictions that are adjacent to or fall completely outside of 2020.

### Data extraction

The systematic searches in the peer-reviewed scientific literature, the exploratory searches of preprint archives and the look-up searches in the gray literature resulted in document material being examined prior to data extraction. In this inclusion-confirming step, titles and summaries of the documents obtained were reviewed against the study criteria (inclusion/exclusion) by two independent reviewers. Documents that both reviewers considered to be included were included and those that both excluded were excluded from further analysis. In case of disagreement, the articles were downloaded in full text and a new assessment was made. If the disagreement persisted, this was resolved through discussions between the reviewers and, if necessary, with the research group. For data extraction from the final set of documents, a tool for retrieving data from each article in full text was developed. The tool included data on the authors' country of origin, study design, forecast methodology (type of model), study population, data sources, forecast period, forecast results, measures of prediction accuracy/performance (if applicable) and model documentation. One reviewer initially extracted data from each included article and then two other reviewers checked the data obtained. The data extracted from the articles were documented in a spreadsheet.

### Risk of bias assessment

All models were assessed for systematic sources of error (bias). In articles that addressed several models, each model was assessed separately. For the assessment, a form, ROBOT (Risk of Bias Opinion Tool), was developed, based on previous guidelines for evaluations of forecast studies^8,22^. In summary, the following topics were examined at model level: relevance and quality of data, time frame for prediction, assumptions, and model development methods (verification and validation). The assessment of assumptions included reproduction rates, latency period, incubation period, serial interval, infectious period, population immunity, and impact of interventions during the prediction period. Model validation was classified as one out of three: retrospective/internal validation, external validation, or no validation.

The assessment of systematic sources of error was performed by two independent assessors, where another assessor assisted in case of disagreement. Each sub-aspect was given a score rating in an assessment form, ROBOT, (see Supplement S3). The partial assessments were added up to a total score for each model. To qualify for further result synthesis, a total score below a heuristically defined limit value was required (ROBOT < 4). Given the impact of predictions made by PHAS these were included in the result synthesis even if they failed the ROBOT cut-off.

### Data synthesis

A secondary validation of model performance was made, where reported predictions were compared with factual outcome data. The data on the forecasting variables were retrieved from published figures using WebPlotDigitizer (v. 4.4, https://apps.automeris.io/wpd/). The models in the final set addressed the total incidence of COVID-19 cases, ICU-occupancy, and incidence of COVID-19 deaths. A simultaneous evaluation of prediction accuracy that included all models was not feasible due to differences in study populations, modeled outcome, and time period. The secondary validation was therefore broken down into subgroups based on the reported outcome variables. Data on the actual outcomes on deaths and ICU-occupancy were obtained from PHAS. Regarding the total case incidence, no source for reliable outcome data was available due to the variable testing strategy employed in Sweden during 2020. When possible, the model performance was quantified by measuring the Mean Absolute Percentage Error (MAPE) between model predictions and the outcome for the entire time period covered by each separate model. We classified the performance according to the following scheme: 0% ≤ MAPE ≤ 10%—excellent, 10% < MAPE ≤ 20%—good, 20% < MAPE ≤ 30%—acceptable and MAPE > 30%—poor. Based on experiences from public health practitioners during the pandemic, as well as the fact that Sweden already before the pandemic lacked healthcare resources (for instance, at average 103 patients share 100 available hospital beds^9^), these limits was considered reasonable. The dates when the predictions were made (models finally calibrated) were retrieved from the articles. We acknowledge that measures have been developed that avoid some of the drawbacks of MAPE (e.g. divergence for outcomes close to zero)^23^, but for clarity and interpretability we opted for MAPE. To determine if difference in prediction errors had statistical significance, we employed the Diedbold-Mariano test. This test requires that the predictions are made for the exact same time period, and we therefore applied the test to the intersection of all prediction dates.

### Total incidence of COVID-19 cases

Not all predictions of the total number of cases did include entire Sweden, but all included the Stockholm region. The evaluation was therefore restricted to forecasting the pandemic development in this region (population 2.3 million). In order to be able to compare predictions of the total incidence of COVID-19 cases from PHAS, we had to adjust the predictions from PHAS, which are in terms of the number of reported cases. In the reports from PHAS (e.g. 35 in Table 1), the proportion of unconfirmed cases was estimated to be 98.7%, which made it possible to rescale the predictions of reported cases by dividing those predictions by (1–0.987), and thus obtaining the total number of cases.

**Table 1:** Studies that passed the assessment of systematic bias (ROBOT evaluation) and additional PHAS reports (references 32,33,34 and 38 added at the end of the listing) considered in the data synthesis and secondary validation .

1a	Sjödin, H, Johansson A, Brännström Å, Farooq Z, Kriit HK, Wilder-Smith A, et al. COVID-19 healthcare demand and mortality in Sweden in response to non-pharmaceutical (NPIs) mitigation and suppression scenarios | medRxiv [Internet]. [citerad 01 mars 2021]. Available from: https://www.medrxiv.org/content/10.1101/2020.03.20.20039594v3
1b	Sjödin H, Johansson AF, Brännström Å, Farooq Z, Kriit HK, Wilder-Smith A, et al. COVID-19 healthcare demand and mortality in Sweden in response to non-pharmaceutical mitigation and suppression scenarios. Int J Epidemiol. 01 oktober 2020;49(5):1443–5﻿3. Available from: https://academic.oup.com/ije/article/49/5/1443/5909271
4	Bryant P, Elofsson A. Estimating the impact of mobility patterns on COVID-19 infection rates in 11 European countries. PeerJ [Internet]. 15 september 2020 [citerad 02 mars 2021];8. Available from: https://www.ncbi.nlm.nih.gov/pmc/articles/PMC7500353/
13a	Gardner J, Willem L, Van Der Wijngaart W, Kamerlin S, Brusselaers N, Kasson P. Intervention strategies against COVID-19 and their estimated impact on Swedish healthcare capacity | medRxiv [Internet]. [citerad 01 mars 2021]. Available from: https://www.medrxiv.org/content/10.1101/2020.04.11.20062133v1
13b	Kamerlin SCL, Kasson PM. Managing Coronavirus Disease 2019 Spread With Voluntary Public Health Measures: Sweden as a Case Study for Pandemic Control. Clin Infect Dis. 15 december 2020;71(12):3174–81. Available from: https://academic.oup.com/cid/article/71/12/3174/5866094
19	Hult H, Favero M. Estimates of the proportion of SARS-CoV-2 infected individuals in Sweden. arXiv:200,513,519 [physics, q-bio] [Internet]. 25 maj 2020 [citerad 02 mars 2021]; Available from: http://arxiv.org/abs/2005.13519
30	Soubeyrand S, Ribaud M, Baudrot V, Allard D, Pommeret D, Roques L. The current COVID-19 wave will likely be mitigated in the second-line European countries. medRxiv. 22 april 2020;2020.04.17.20069179. Available from: https://www.medrxiv.org/content/10.1101/2020.04.17.20069179v1
35	Skattning av peakdag och antal infekterade i COVID-19-utbrottet i Stockholms län februari-april 2020 [Elektronisk resurs] [Internet]. 2020. Available from: http://www.folkhalsomyndigheten.se/publicerat-material/publikationsarkiv/s/skattning-av-peakdag-och-antal-infekterade-i-COVID-19-utbrottet-i-stockholms-lan-februari-april-2020
36	Estimates of the number of infected individuals during the COVID-19 outbreak in the Dalarna region, Skåne region, Stockholm region, and Västra Götaland region, Sweden [Elektronisk resurs] [Internet]. 2020. Available from: http://www.folkhalsomyndigheten.se/publicerat-material/publikationsarkiv/e/estimates-of-the-number-of-infected-individuals-during-the-COVID-19-outbreak
37	Effekt av ökade kontakter och ökat resande i Sverige sommaren 2020 [Elektronisk resurs] [Internet]. 2020. Available from: http://www.folkhalsomyndigheten.se/publicerat-material/publikationsarkiv/e/effekt-av-okade-kontakter-och-okat-resande-i-sverige-sommaren-2020
45	ECDC. Projected baselines of COVID-19 in the EU/EEA and the UK for assessing the impact of de-escalation of measures [Internet]. s. 31. Available from: https://www.ecdc.europa.eu/sites/default/files/documents/Projected-baselines-COVID-19-for-assessing-impact-measures.pdf
46	ECDC. Baseline projections of COVID-19 in the EU/EEA and the UK: update [Internet]. s. 34. Available from: https://www.ecdc.europa.eu/sites/default/files/documents/ECDC-30-day-projections-Sept-2020.pdf
**PHAS REPORTS WITH ROBOT SCORES > 3**	
32	Skattning av behov av slutenvårdsplatser COVID-19 (den 20 mars 2020, uppdaterad 27 mars 2020) [Internet]. 2020 mar s. 46. Available from: https://www.folkhalsomyndigheten.se/contentassets/1887947af0524fd8b2c6fa71e0332a87/skattning-av-vardplatsbehov-folkhalsomyndigheten.pdf
33	Skattning av behov av slutenvårdsplatser COVID-19 (den 3 april 2020) [Internet]. 2020 apr s. 4. Available from: https://www.folkhalsomyndigheten.se/contentassets/4b4dd8c7e15d48d2be744248794d1438/skattning-av-behov-av-slutenvardsplatser-covid-lombardiet.pdf
34	Skattning av behov av slutenvårdsplatser COVID-19 (den 13 maj 2020) [Internet]. 2020 maj. Available from: https://www.folkhalsomyndigheten.se/contentassets/4b4dd8c7e15d48d2be744248794d1438/vardbehov-scenarier-vardbelastning-baserat-svenska-data-20200514.pdf
38	Scenarier – Tre smittspridningsscenarier inom regeringsuppdraget ”Plan inför eventuella nya utbrott av COVID-19″ [Elektronisk resurs] [Internet]. 2020. Available from: http://www.folkhalsomyndigheten.se/publicerat-material/publikationsarkiv/s/scenarier--tre-smittspridningsscenarier-inom-regeringsuppdraget-plan-infor-eventuella-nya-utbrott-av-COVID-19

### ICU-occupancy

All predictions of ICU-occupancy did not include the entire country but did include the Stockholm region. Also, this evaluation was therefore restricted to the Stockholm region. While acknowledging that assumptions regarding epidemiological homogeneity introduce uncertainty, we multiplied the predictions by the proportion of the total Swedish population that lived in the Stockholm region to allow comparisons with the entire country.

### Incidence of COVID-19 deaths

We compared predictions of the number of deaths in COVID-19 during the spring of 2020. In relation to this, we also analysed how much historical data was used to calibrate the models in relation to the length of the prediction by calculating the ratio of the number of days of data used in the calibration and the length of the prediction (in days).

## Results

The systematic search identified a total of 1,086 peer-reviewed scientific articles published during the period 1 January 2020–31 December 2020. After duplicate control and exclusion of preprints indexed in databases for scientific publications, 892 articles remained.

**Figure 1:**
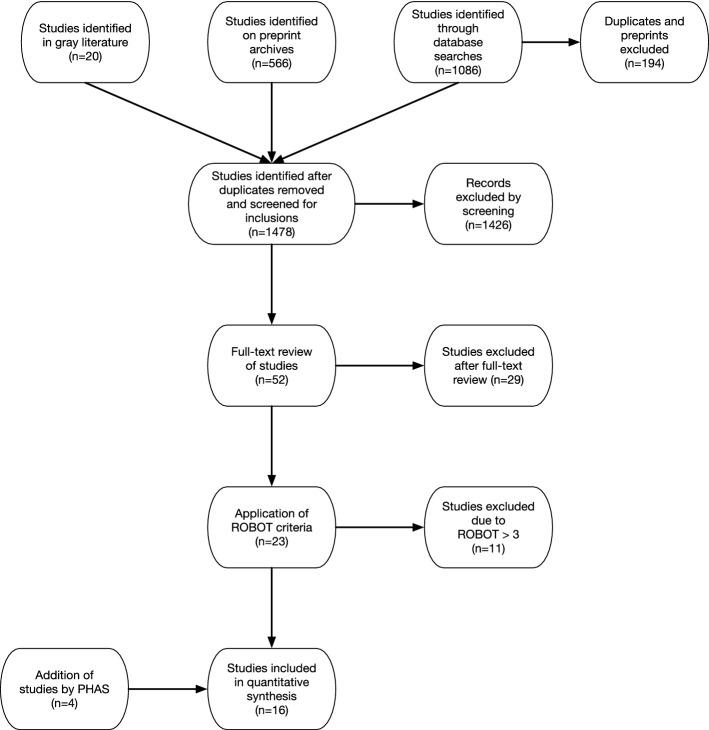
PRISMA flow-chart indicating the number of studies identified, screened, and confirmed for eligibility into this systematic review.

From the exploratory search in the preprint archive, 566 articles were identified. In addition, the search in the gray literature resulted in 20 additional reports for inclusion assessment. A complete list of identified titles can be found in Supplement S4. A flowchart of the selection process can be seen in Fig. 1.

At the data extraction stage there was disagreement regarding inclusion/exclusion of five articles. Discussion among the reviewers was sufficient to resolve these disagreements. After assessment of titles and abstracts for inclusion, 52 unique articles found to have met the study criteria (of which 4 studies were published twice, first as preprints and then as peer-reviewed articles). These were divided into scientific articles (n = 9), pre-prints (n = 19), articles with previous pre-prints (n = 4 [total number of titles n = 8]) and gray literature (n = 20), Table 1. After a full text review with regards to the study criteria, 29 titles were excluded. The main reasons for exclusion were that no predictions or scenarios were presented, these did not include Sweden, or that no Swedish data were used in the analysis. Assessment of the remaining 23 titles with respect to systematic bias according to ROBOT criteria resulted in elimination of 11 articles. Among the articles that went into the evaluation using the ROBOT-criteria the reviewers obtained ROBOT-scores that led to disagreement on the inclusion/exclusion in two cases (articles 13a and 45). After a review by an independent assessor these studies were included for further analysis.

### Forecasting accuracy

We included the 4 reports by PHAS that failed the ROBOT limit in the evaluation of forecasting accuracy, which resulted in that the data synthesis and secondary validation incorporated 16 studies (see Table 1).

### Total daily incidence of COVID-19 cases

**Figure 2:**
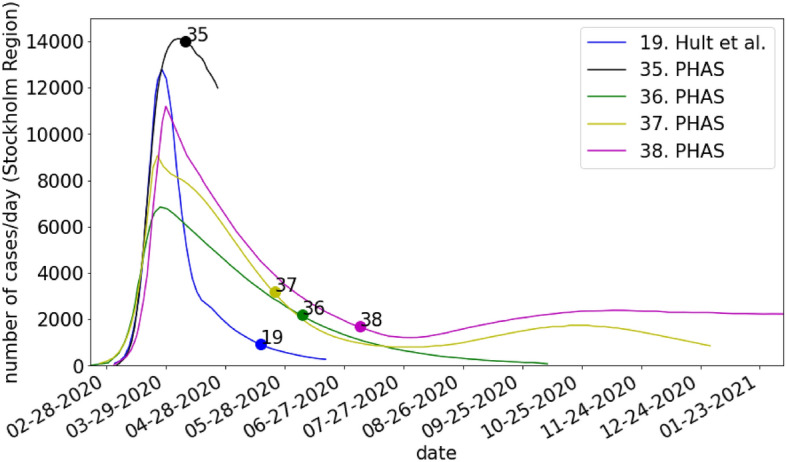
Comparison of the predicted incidence of COVID-19 cases/day in Stockholm Region. The circles on the curves show the date up until which data was used for calibrating the model. No consistent data on the number of factual cases for the entire time period exist.

The five predictions included in the secondary validation of the total daily incidence of COVID-19 cases are shown in Fig. 2. One of the predictions included was presented in a PHAS report (38) that did not pass the ROBOT evaluation. At the time of prediction (between April and July 2020), all models estimated retrospectively that the peak of the first wave already had occurred during the first half of April. The predictions of total daily case incidence thus only covered the decreasing phase of the pandemic wave. The early published models (19 Hult et al., 35 PHAS) indicated symmetrical shapes of the pandemic wave, while the later models (36–38 PHAS) generated skewed epi curves.

### ICU-occupancy

**Figure 3:**
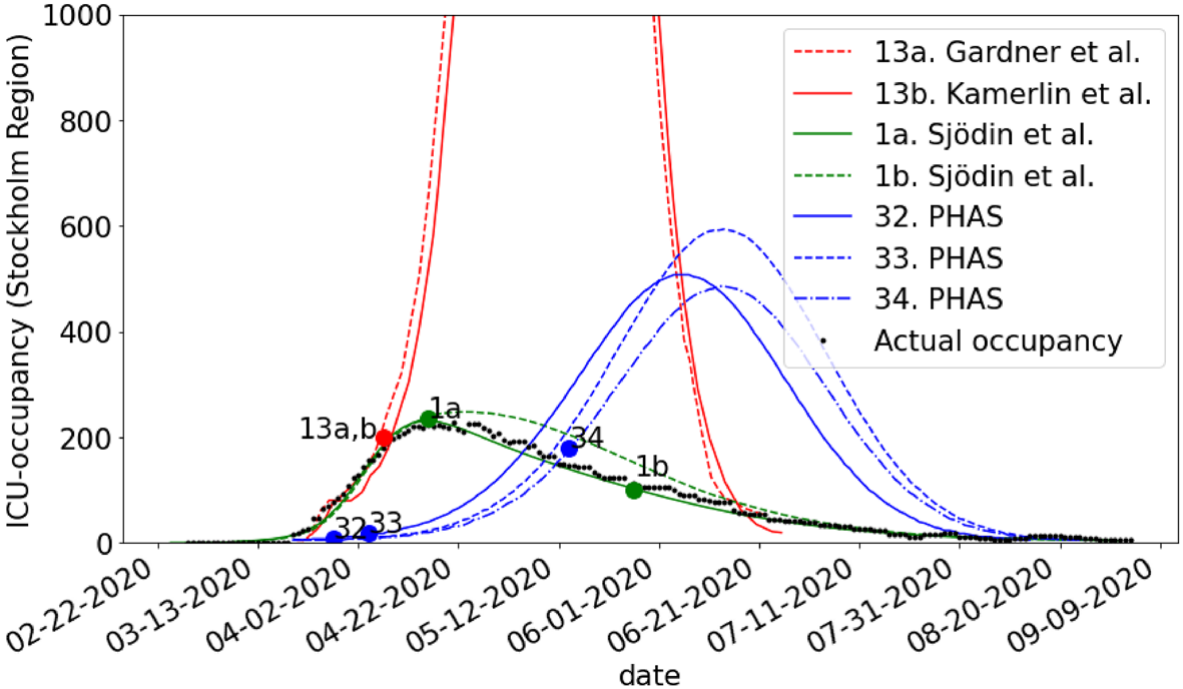
Predicted and actual outcome for the ICU-occupancy in Stockholm Region. The circles on the curves show the date up until which data was used in order to calibrate the model. Since PHAS did not calibrate these models using data we instead show the publication date for each report.

Figure 3 exhibits the predictions of ICU-occupancy (the four models that passed the ROBOT evaluation and three additional PHAS models (32–34)) together with the factual occupancy. The model performance in terms of MAPE is summarized in Table 2. Two models(1a, 1b Sjödin et al.) achieves good or acceptable accuracy, whereas the accuracy of all other models was poor. Four models (13a Gardner et al.), (13b Kammerlin et al.), (32 and 33 PHAS) were calibrated prior to the peak, whereas (1a Sjödin et al.) was calibrated with data up to and including the peak and (1b Sjödin et al.) used data until end of May. Prediction accuracy for all pairs of models were significantly different (Diebold-Mariano test, p < 0.05) except for the pairs (13a,13b) and (32,34), which visually also are very similar.

**Table 2:** Comparison of model accuracy for models that predicted the ICU-occupancy in Stockholm Region during the first wave of the pandemic in 2020.

Study	Prediction accuracy (MAPE) (%)
1b. Sjödin et al	14
1a. Sjödin et al	22
32. PHAS	224
34. PHAS	277
33. PHAS	346
13a. Gardner et al	868
13b. Kamerlin et al	931

### Incidence of deaths in COVID-19

**Figure 4:**
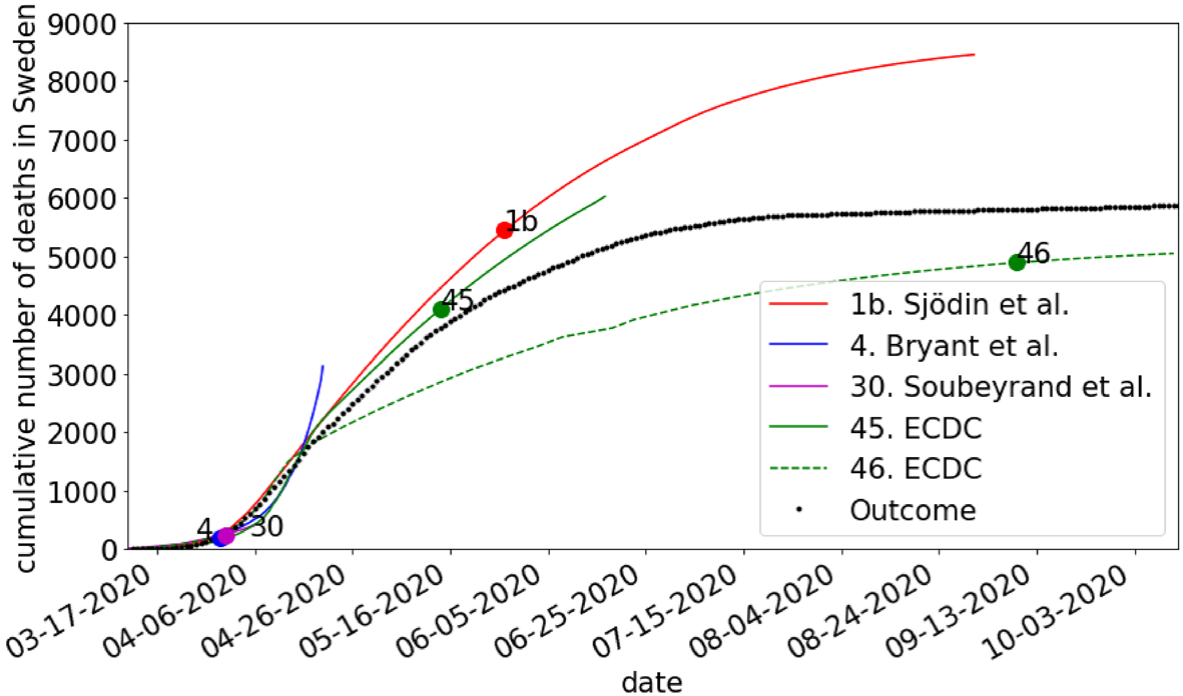
Comparison of modelled and actual number of deaths in Sweden during 2020. The circles on the curves show the date up until which data was used for calibrating the model.

Five models predicted the incidence of COVID-19 deaths during the early pandemic stages (Fig. 4). The factual peak in incidence of deaths occurred in mid-April. All predictions except one (30 Soubeyrand et al.) cover this period. The model performance in terms of MAPE is summarized in Table 3. One model (4 Bryant et al.) achieved good accuracy and another model (45 ECDC) acceptable accuracy, whereas the accuracy of the remaining models was poor. It is noteworthy that the two earliest models (4, 30) exhibited the best and worst accuracy, respectively. Although one model (46 ECDC) was published three months later than the other models, it still achieves poor accuracy. Regarding the amount of historical data used to calibrate the models in relation to the length of the prediction, (4 Bryant et al.) used the least amount data, whereas (46 ECDC) uses the most data (see Table 3). Prediction accuracy for all pairs of models were significantly different (Diebold-Mariano test, p < 0.05) except for the pair (4,45). In the analysis of significant prediction accuracy, one prediction model (30. Soubeyrand et al.) was excluded because it only provided a week-long prediction.

**Table 3:** Comparison of model accuracy for models that predicted the cumulative number of death due to COVID-19 in Sweden during 2020.

Study	Prediction accuracy (MAPE) (%)	Length of prediction/length of calibration
4. Bryant et al	18	1.67
45. ECDC	23	0.59
46. ECDC	43	0.18
1b. Sjödin et al	49	1.26
30. Soubeyrand et al	390	0.37

## Discussion

This study set out to review and assess computational forecasting models involving Sweden developed during the early stages of the COVID-19 pandemic. We found that from 1478 initially assessed unique articles, 1455 were excluded after full-text review and only 12 remained after the ROBOT evaluation. With regards to potential use as support for policy-making, the average methodological quality of the reporting from the early pandemic modeling was thus generally disappointing. Also drawing conclusions about the accuracy of the models with acceptable methodological quality was challenging because some models were published before the time period for the prediction, while other models were published during the prediction period or even afterwards.

Shifts in testing strategies poses a critical problem when attempting to validate models of the total incidence of infected individuals during the early phases of a pandemic^10^. In Sweden, only persons with severe symptoms admitted to hospital were tested during the first month of the pandemic. As the testing capacity was increased, cases with mild symptoms came to dominate the incidence data. In their efforts to validate predictions against standardized outcome data, PHAS manually excluded cases detected through mass-testing and calibrated their models against “admitted patients”. However, we note that the predictions made by PHAS are similar to the only model published by researchers that passed the ROBOT evaluation (19 Hult et al.) which was calibrated against data on COVID-19 deaths. The issue that the factual transmission of the infection is unknown in the general population during the early phases of a pandemic is a generic problem. One solution to this problem includes rapid development of reliable tests and administrating these in sufficient amounts to representative population samples^11,12^.

Regarding predictions of ICU-occupancy, we found in our secondary validation the accuracy of two models to be at least acceptable. These models were in practice identical as (1a Sjödin et al.) is the preprint version of (1b Sjödin et al.). The later model was however calibrated with data from a later date, which improved the apparent accuracy. With regards to predicting the cumulative number of deaths due to COVID-19, we found the accuracy of two models to be at least acceptable. Of these, the model (4 Bryant et al.) that used the least amount of training data in relation to the length of the prediction yet obtained the lowest prediction error in terms of MAPE. A possible explanation of this finding is that a dynamic compartmental model (e.g. SEIR-model) that fits the beginning of the pandemic poorly will usually not perform better if it is calibrated on a longer time series. This is likely why the model issued by the ECDC (46 ECDC) performs poorly and also the reason why the models from PHAS (32,33,34 PHAS) fits the data poorly. Although for the latter studies a transparent documentation is lacking making it difficult to draw conclusions.

An explanation of the overall limited accuracy of the forecasting models is that although not explicitly stated, most of the modeling was performed as scenario analyses, rather than as predictions. In modeling, prediction refers to establishment of the most probable development of a studied phenomenon. The term scenario, on the other hand, typically refers to a simplified description of how the phenomenon may develop in the future depending on the different assumptions made^13^. However, the reporting of the models was often unclear regarding whether the modeling referred to real-world predictions or analyses of virtual scenarios. For example, the stated overall aim for one study (1b Sjödin et al.) was to create pandemic scenarios, but at the same time the terms “predict” or “prediction” were used 16 times in the article. The forecasting was performed by fitting the empirical data available at the time to a framework for infectious disease modelling (‘Scenario d’, Personal communication with the authors). Additional scenarios were then generated from this model by adjusting the parameter values for social distancing and infectious period. Similar ambiguities between prediction and scenario were found in one other study (13b Kamerlin et al.), while in reports from the ECDC (45,46) "scenario", "prediction" and "projection" were used interchangeably. This ambiguity is problematic, because the default purpose (assumed by non-modelers) of pandemic predictions is to indicate the most probable outcome, i.e. to point out the most probable development of the pandemic. Scenario models, on the other hand, are part of systems that include both computational models and human decision-makers, where the decision-makers evaluate the relevance of different assumptions. This type of analysis and decision-making in human-model systems is well known from areas where decisions need to be made in complex environments with limited availability to information, e.g. analyses of global warming^14^ and medical decision-making^15^. A prerequisite for effective human-model systems is that the human decision-maker's situational understanding is allowed to be taken into account^16^. This means that if they are to be used in practical decision-making, the goals and assumptions underpinning each individual scenario model must be clearly described, e.g. if the model is intended to describe a “worst-case development”.

At the global level, few systematic evaluations of the accuracy of computational modeling performed during the early phases of the COVID-19 pandemic have been reported. Nonetheless, the studies available provide interesting insights. For instances, the COVID-19 Forecast hub program (https://covid19forecasthub.org/) in the United States collected in 2020 pandemic predictions from models developed by more than 50 research groups^17,18^. The evaluation also included a naïve basic model and an ensemble model, which combined all predictions made at any given time. Probabilistic predictions were recorded at the time they were performed. Predictions from 23 different models were evaluated prospectively with respect to actual outcome, that is, morbidity and mortality in COVID-19 at the state and national level^17^. Only half of the evaluated models showed better accuracy than the naïve basic model, while the ensemble model showed the best prediction outcome. The advantage of ensemble models over single models was later confirmed in^18^. A similar study was carried out in Poland and Germany during the second wave^19^. They collected prospective predictions ranging from one to four weeks and used probabilistic outcome measures. In contrast to the evaluation in the United States^17,18^ they did not find an ensemble model to be optimal, but instead different individual models performed best. These observations imply that consensus is needed on protocols for evaluations of early pandemic modeling efforts, where issues ranging from primary endpoint measurements to management of prediction periods and reporting practices are defined.

Regarding limitations of our study, it should be taken into consideration that there is no established consensus on methods for systematic review and secondary validation of early pandemic modeling. It must be taken into consideration that our study was limited to one country and that the results cannot be immediately generalized directly to other settings.

Another limitation that relates to generalizability is the definition and registration of cases and deaths. The number of reported cases is, as discussed earlier, related to testing capacity and this changed over time during the study period^24^. Moreover, the registration of deaths during the first year of the pandemic differed between countries regarding mainly two aspects; (1) if deaths at all were registered and, (2) if deaths were registered as deceased ”caused by” COVID-19 (i.e. as the underlying cause of death) or deceased “associated with” COVID-19. In Sweden, through 2020, individuals with verified COVID-19 that passed away within 30 days from registered diagnosis were regarded as deaths ”caused by” COVID-19.

We have not considered unpublished models that were used by local and regional health authorities in our systematic review. Therefore, our conclusions regarding the poor accuracy of predictions might not reflect the accuracy of predictions that were used in the actual pandemic response practice.

Our results have some important implications for future research. The minute attention paid to the to the question “How trustworthy is the forecast?” in the studies assessed is noteworthy. Only four (35, 46, 4 Bryant et al., 30 Soubeyrand et al.) studies included some kind of self-validation, while the outcome of the validation was quantified in two studies (4 Bryant et al., 30 Soubeyrand et al.). Among PHAS's reports, self-validation was limited to one study (35), whereas for another study (38) the outcome was validated later in a separate publication. This implies that more attention should be paid to the validity and accuracy of predictions in future pandemic forecasting. Furthermore, from a health policy perspective, the studies would have contributed more useful information to policy-making during the first pandemic wave if it would have been clear what was considered as predictions and what was considered as alternative developments given different underpinning assumptions. Our results thus showcase the need for further research on methods for pandemic scenario modeling. In order to evaluate a scenario analysis based on its purpose (i.e. to generate realistic scenarios) plausibility and variation of outcomes must be included in the assessment criteria. Our results indicate that future reports of pandemic scenario models should focus on properties such as internal logic^20^, plausibility^14^ and pluralism^21^.

We conclude that the forecasting models involving Sweden developed during the early stages of the COVID-19 pandemic in 2020 had limited accuracy. Despite their present limitations, it is of importance to attain knowledge about early pandemic models to be able to prepare more effective modelling strategies in settings with large uncertainties^5^. The knowledge attained in this study can thus be used to improve our preparedness for coming pandemics.

## Supplementary Information

Supplementary Information 1.

Supplementary Information 2.

Supplementary Information 3.

Supplementary Information 4.
