# Supplementary material for: Computational models predicting the early development of the COVID-19 pandemic in Sweden: systematic review, data synthesis, and secondary validation of accuracy
Source: Sci Rep. 2022 Aug 2;12:13256. doi: 10.1038/s41598-022-16159-6 (PMC9345013; doi:10.1038/s41598-022-16159-6)
Supplement: Supplementary file 1 — Supplementary Information 1. [file 41598_2022_16159_MOESM1_ESM.pdf]

Citation

Toomas Timpka, Anna Joud, Armin Spreco, Philip Gerlee. Compilation and assessment of models for forecasting the progress of the COVID-19 pandemic in Sweden during 2020. PROSPERO 2021 CRD42021229514 Available from:
https://www.crd.york.ac.uk/prospero/display_record.php?ID=CRD42021229514

Review question

How useful and accurate were published models for forecasting the progress of the Covid-19 pandemic in Sweden in 2020? The specific research questions are:

1.What different types of models for forecasts of the Covid-19 pandemic were published between 2020-01-01 and 2020-12-31 where Sweden is explicitly distinguishable in the results, and

2.What is the accuracy of forecasts of the Covid-19 pandemic published between 2020-01-01 and 2020-12-31 where Sweden is explicitly distinguishable in the results.

Searches

The searches of the literature for the review and compilation were planned using the 'Search Triangle" model (Gusenbaum 2020). Systematic searches will between 21-01-22 and 21-01-29 be performed of databases containing peer-reviewed publications in for the review question relevant areas (PubMed, Cochrane Library, Embase, Love platform / Epistemikos), explorative searches be performed of preprint archives, and look-up searches be performed of the grey literature. The procedure will be reported according to the PRISMA-S protocol (version September, 2020). The systematic search of the peer-reviewed literature will have the goal to identify all relevant records (within the resource constraints) in a transparent and reproducible manner. The exploratory searches of preprint archives will be initiated by submitting a tentative query using a discovery tool (search.bioPreprint) to the archives, then explore the retreived documents to better understand how to exploit the archive contents, and finally iteratively seek and obtain cues about the next steps until adjustments no longer lead to significant changes in the retreived set of documents. The exploratory searching will thus be characterized by learning, where the researchers expect to be exposed to different, sometimes contradicting, reports of forecasting models when extracting data from the preprint databases. Finally, a look-up search of the grey literature will be performed. The lookup search — also called “known item search” — will be conducted with a clear goal of retreving documents from the websites of relevant Swedish and international health service organisations and complemented with document retrieval from reference lists.

Gusenbauer M, Haddaway NR. What every Researcher should know about Searching - Clarified Concepts, Search Advice, and an Agenda to improve Finding in Academia. Res Synth Methods. 2020 Oct 8. doi: 10.1002/jrsm.1457. Epub ahead of print.

Types of study to be included

Inclusion criteria

Studies must report real-world or scenario-based Covid-19 forecasting models involving Swedish populations.

We will include studies that forecast relevant epidemiological outcomes during the Covid-19 pandemic in 2020. We require studies to explicitly forecast a future outcome and include also estimations of key epidemic parameters (for instance R).

Exclusion criteria

• The study does not include original analyses (e.g. review articles, perspective pieces, editorials, recommendations, and guidelines)

• Duplicated studies

• In vitro studies (pure simulations)

• Descriptive epidemiological publications (e.g. describing case positive proportions, total case numbers, descriptive mapping of incidence by geographic information systems)

• Models which only examined effect of interventions (rather than forecasting risk or burden)

• Studies which only present matemathical models or software tools, unless the central aim of the study was to explicitly forecast Covid-19 phenomena

Condition or domain being studied

The Covid-19 pandemic during 2020.

Participants/population

The total Swedish population.

Intervention(s), exposure(s)

Exposure to SARS-Cov-2 in Sweden causing Covid-19 in 2020.

Comparator(s)/control

Forecasts are compared with factual outcomes according to data recorded in healthcare and public health databases.

Context

The included studies are expected to report of at least one of two model types, each with different sets of main characteristics:

a) real-world forecasting models used to analyse Swedish populations determined in time and by physical location with a reported health outcome for these populations at a future time.
b) forecasting models used in scenario settings to analyse Swedish populations not completely defined in time and / or physical location, but with a forecasted health outcome for the population (what-if models).

Main outcome(s)

Covid-19 patient loads in health care; Covid-19 morbidity; Covid-19 mortality; R (Effective reproduction number).

Measures of effect

Accuracy of correlations between time series of forecasted outcomes and observed outcomes and mean absolute percentage error of forecasted outcomes compared with observed outcomes.

Additional outcome(s)

None.

Measures of effect

Not applicable.

Data extraction (selection and coding)

The materials for the data extraction will be produced by systematic searches of the peer-reviewed literature, exploratory searches of preprint archives, and look-up searches of the grey literature on the review topic. Titles and abstracts of the retreived documents will be screened for compliance with the review criteria / inclusion by two independent (blinded) reviewers. Documents found eligible by both reviewers will be directly selected and those discarded by both reviewers excluded from further consideration. In cases of

disagreement, full-text articles will be retrieved and new assessment made. If the disagreement remains, it will resolved, first, by internal discussions between the reviewers, and, second, if needed, by a senior researcher. For the data extraction from the final set of selected documents, a tool has been developed for recording from each full text article information about authors' country of origin, the study design, forecasting methodology (type of model), study population, sources of data, forecasting period, forecasted outcomes, measures of forecasting accuracy / performance (where applicable), and model documentation. Missing information in the full text article on any data extraction item in the tool will be recorded. One reviewer will initially extract data on each selected article and thereafter two other reviewers will check the extracted data. The article selection and data extraction processes will be documented in Excel spreadsheets.

Risk of bias (quality) assessment

The studies reporting a) real-world forecasting models and b) scenario-based forecasting models will be assessed separately. For articles addressing multiple models and not explicitly addressing a single model, all models will be included and assessed. For the quality assessment, we have developed a form based on previous infectious disease forecasting studies, current methodological consensus, and the TRIPOD Exploration and Elaboration document. In summary, the following topics will be assessed at model level: relevance and quality of data, time frame for forecasts, assumptions made, model development methods (verification), and validation. The assessment of assumptions will include reproduction numbers, latency period, incubation period, serial interval, infectious period, population immunity proportions, and interventions during the forecasting period. The model validation will be graded with regards to retrospective / internal validation, prospective / external validation, or no validation. Assessment of the bias evaluation form items will be performed by two independent assessors and an additional senior assessor when disagreements occur. Each model will be provided a a risk of bias (RoB) grading (low / medium / high) for each item in the assessment form. These gradings will be computed to a RoB score for each model.

Strategy for data synthesis

Proceeding to the data synthesis stage, all models with a RoB score above the cut-off level will excluded. For the models included in the final set, full model documentation will be retreived. In each model category, we will assess the forecasting performance of the models. If needed, authors will be asked for the additional information that is required to enable a reliable data synthesis. The data synthesis (quantitative and qualitative) will be guided by a synthesis protocol covering areas ranging from model output corrobation to data evaluation.

Real-world forecasting studies

Model output corroboration - Model forecasts will be compared with observed outcomes, also those that were not known when the model was parameterised and reported.

Data evaluation - The quality of numerical data and assumptions used to parameterise the model and of the sources that were used to design the overall model structure will be assessed.

Reproducability - Whether the information about the computerised implementation of the model made available is sufficient to reproduce the reported results will be verified.

Scenario-based forecasting studies

Conceptual model evaluation - The (simplifying) assumptions underlying the scenario design and forming its building blocks, including whether the structure, essential theories, and causal relationships are reasonable to form a logically consistent model, will be assessed.

Model output verification - How well the forecasting model output in the scenario matches observations and to what degree calibration was involved in obtaining good fits of model output and data will be assessed. The aim is to ensure that the scenario models respond to virus features and environmental conditions in a sufficiently similar way as their real counterparts.

Data evaluation - The quality of numerical and qualitative data used to parameterise the forecasting models included in the scenario and of the sources used to design the overall scenario structure will be assessed.

Analysis of subgroups or subsets

Forecasting studies addressing the populations in Swedish regions and counties will be analysed separately.

Contact details for further information

Toomas Timpka
toomas.timpka@liu.se

Organisational affiliation of the review

Linkoping University
www.liu.se

Review team members and their organisational affiliations

Professor Toomas Timpka. Linkoping University
Assistant/Associate Professor Anna Joud. Lund University
Dr Armin Spreco. Linkoping University
Assistant/Associate Professor Philip Gerlee. Chalmers Institute of Technology

Type and method of review

Epidemiologic, Prognostic, Systematic review

Anticipated or actual start date

11 January 2021

Anticipated completion date

15 February 2021

Funding sources/sponsors

Public Health Agency of Sweden (Folkhälsomyndigheten)

Conflicts of interest

Language

English, Swedish

Country

Sweden

Stage of review

Review Ongoing

Subject index terms status

Subject indexing assigned by CRD

Subject index terms

COVID-19; Forecasting; Humans; Pandemics; SARS-CoV-2; Sweden

Date of registration in PROSPERO

19 January 2021

Date of first submission

17 January 2021

Stage of review at time of this submission

Stage	Started	Completed
Preliminary searches	Yes	No
Piloting of the study selection process	Yes	No
Formal screening of search results against eligibility criteria	Yes	No
Data extraction	No	No
Risk of bias (quality) assessment	No	No
Data analysis	No	No

The record owner confirms that the information they have supplied for this submission is accurate and complete and they understand that deliberate provision of inaccurate information or omission of data may be construed as scientific misconduct.
The record owner confirms that they will update the status of the review when it is completed and will add publication details in due course.

Versions

19 January 2021
