# Supplementary material for: Computational models predicting the early development of the COVID-19 pandemic in Sweden: systematic review, data synthesis, and secondary validation of accuracy
Source: Sci Rep. 2022 Aug 2;12:13256. doi: 10.1038/s41598-022-16159-6 (PMC9345013; doi:10.1038/s41598-022-16159-6)
Supplement: Supplementary file 2 — Supplementary Information 2. [file 41598_2022_16159_MOESM2_ESM.docx]

# Supplementary material 2. PRISMA-S protocol

| **Section/topic** | | | **#** | **Checklist item** |
| --- | --- | --- | --- | --- |
| **Information sources and methods** | | | | |
| Database name | | | 1 | PubMed, Cochrane Library, Embase, Love platform / Epistemikos |
| Multi-database searching | | | 2 |  |
| Study registries | | | 3 |  |
| Online resources and browsing | | | 4 | We used three different but partially overlapping databases for pre-prints. In these the following search terms were used:  [https://www.medrxiv.org/](https://www.medrxiv.org/%0d)   Search term: #covid-19 #Sweden #model #forecast   Search term: #Covid-19 #nowcast  <https://arxiv.org/>   Search term: #Covid-19 #model (abstract) #Sweden (abstract)   Search term: #Covid-19 (abstract) #Sweden (all fields)   Search term: #Covid-19 #nowcast  [https://www.hsls.pitt.edu/preprint](https://www.hsls.pitt.edu/preprint%0d)  inkluderar: [AAS Open Res](https://aasopenresearch.org/), [bioRxiv](https://www.biorxiv.org/), [ChemRxiv](https://chemrxiv.org/), [F1000Res](https://f1000research.com/), [Gates Open Res](https://gatesopenresearch.org/), [HRB Open Res](https://hrbopenresearch.org/), [medRxiv](https://www.medrxiv.org/), [MNI Open Res](https://mniopenresearch.org/), [PeerJ Preprints](https://peerj.com/preprints/), [Preprints.org](https://www.preprints.org/), [Research Square](https://www.researchsquare.com/), [Wellcome Open Res.](https://wellcomeopenresearch.org/)  Search term: #COVID-19 #Sweden #forecast #prediction |
| Citation searching | | | 5 |  |
| Contacts | | | 6 |  |
| Other methods | | | 7 | A direct search of the grey literature was performed at the Public Health Authority of Sweden (PHAS), the National Board of Health and Welfare, the Swedish Civil Contingencies Agency and the European Center for Disease Prevention and Control (ECDC). |
| **Search strategies** | | | | |
| Full search strategies | 8 | For published articles the below search matrix was used   PubMed  #1  (prediction[Title/Abstract] OR predicting[Title/Abstract] OR nowcast[Title/Abstract] OR nowcasting[Title/Abstract] OR modeling[Title/Abstract] OR modelling[Title/Abstract] OR model[Title/Abstract] OR models[Title/Abstract] OR modell[Title/Abstract] OR forecast[Title/Abstract] OR forecasting[Title/Abstract] OR epidemiology[Title/Abstract] OR "simulation model"[Title/Abstract] OR estimating[Title/Abstract] OR estimation[Title/Abstract] OR scenario[Title/Abstract] OR surveillance[Title/Abstract]) OR (epidemiology[MeSH Terms])  #2  (covid-19[Title/Abstract] OR SARS-CoV-2 OR"severe acute respiratory syndrome coronavirus 2"[Title/Abstract]) OR (covid-19 OR SARS-CoV-2[MeSH Terms])  #3  Swed*  #4  #1 AND #2 AND #3  Filters applied: Case Reports, Clinical Conference, Clinical Study, Clinical Trial, Clinical Trial Protocol, Clinical Trial, Phase I, Clinical Trial, Phase II, Clinical Trial, Phase III, Clinical Trial, Phase IV, Comparative Study, Controlled Clinical Trial, Corrected and Republished Article, Evaluation Study, Guideline, Multicenter Study, Observational Study, Practice Guideline, Pragmatic Clinical Trial, Preprint, Published Erratum, Randomized Controlled Trial, Technical Report.  #5  #1 AND #2 AND #3 NOT review  #6  #4 AND #5  =166 references    Embase  #1  prediction:ab,ti OR predicting:ab,ti OR nowcast:ab,ti OR nowcasting:ab,ti OR modeling:ab,ti OR modelling:ab,ti OR model:ab,ti OR models:ab,ti OR modell:ab,ti OR forecast:ab,ti OR forecasting:ab,ti OR epidemiology:ab,ti OR 'simulation model':ab,ti OR estimating:ab,ti OR estimation:ab,ti OR scenario:ab,ti OR surveillance:ab,ti OR 'epidemiology'/exp  #2  'covid 19':ab,ti OR 'sars cov 2':ab,ti OR 'severe acute respiratory syndrome coronavirus 2':ab,ti OR 'covid 19'/exp OR 'severe acute respiratory syndrome coronavirus 2'/exp OR 'sars cov 2'/exp  #3  Swed*  #4  #1 AND #2 AND #3  #5  'review'/exp OR review OR commentary  #6  #4 NOT #5  =319 references    Scopus  #1  TITLE-ABS-KEY ( prediction  OR  predicting  OR  nowcast  OR  nowcasting  OR modeling  OR  modelling  OR  model  OR  models  OR  modell  OR  forecast  OR  forecasting  OR  epidemiology  OR  "simulation model"  OR  estimating  OR  estimation  OR  scenario  OR  surveillance )  #2  TITLE-ABS-KEY ( covid-19  OR  sars-cov-2  OR  "severe acute respiratory syndrome coronavirus 2" )  #3  Swed*  #4  #1 AND #2 AND #3  ( TITLE-ABS-KEY ( prediction  OR  predicting  OR  nowcast  OR  nowcasting  OR  modeling  OR  modelling  OR  model  OR  models  OR  modell  OR  forecast  OR  forecasting  OR  epidemiology  OR  "simulation model"  OR  estimating  OR  estimation  OR  scenario  OR  surveillance ) )  AND  ( TITLE-ABS-KEY ( covid-19  OR  sars-cov-2  OR  "severe acute respiratory syndrome coronavirus 2" ) )  AND  ( ALL ( swed* ) )  AND  ( LIMIT-TO ( DOCTYPE ,  "ar" )  OR  LIMIT-TO ( DOCTYPE ,  "le" )  OR  LIMIT-TO ( DOCTYPE ,  "cp" )  OR  LIMIT-TO ( DOCTYPE ,  "no" )  OR  LIMIT-TO ( DOCTYPE ,  "ed" )  OR  LIMIT-TO ( DOCTYPE ,  "er" )  OR  LIMIT-TO ( DOCTYPE ,  "sh" ) )  = 453 references  #5  Review  #6  #4 AND NOT #5  ( ( TITLE-ABS-KEY ( prediction  OR  predicting  OR  nowcast  OR  nowcasting  OR  modeling  OR  modelling  OR  model  OR  models  OR  modell  OR  forecast  OR  forecasting  OR  epidemiology  OR  "simulation model"  OR  estimating  OR  estimation  OR  scenario  OR  surveillance ) )  AND  ( TITLE-ABS-KEY ( covid-19  OR  sars-cov-2  OR  "severe acute respiratory syndrome coronavirus 2" ) )  AND  ( ALL ( swed* ) ) )  AND NOT  ( TITLE-ABS-KEY ( review ) )  AND  ( LIMIT-TO ( DOCTYPE ,  "ar" )  OR  LIMIT-TO ( DOCTYPE ,  "le" )  OR  LIMIT-TO ( DOCTYPE ,  "cp" )  OR  LIMIT-TO ( DOCTYPE ,  "no" )  OR  LIMIT-TO ( DOCTYPE ,  "ed" )  OR  LIMIT-TO ( DOCTYPE ,  "er" )  OR  LIMIT-TO ( DOCTYPE ,  "sh" ) )=427 references | | |
| Limits and restri-ctions | 9 | We employed limitations with respect to the time period (January 2020-December 2020) and format according to section 10 below. | | |
| Search filters | 10 | Case Reports, Clinical Conference, Clinical Study, Clinical Trial, Clinical Trial Protocol, Clinical Trial, Phase I, Clinical Trial, Phase II, Clinical Trial, Phase III, Clinical Trial, Phase IV, Comparative Study, Controlled Clinical Trial, Corrected and Republished Article, Evaluation Study, Guideline, Multicenter Study, Observational Study, Practice Guideline, Pragmatic Clinical Trial, Preprint, Published Erratum, Randomized Controlled Trial, Technical Report. | | |
| Prior work | 11 |  | | |
| Updates | 12 |  | | |
| Dates of earches | 13 | 2020-01-22/2020-01-29 | | |
| **Scientific review** | | | | |
| Peer review | 14 |  | | |
| **Identified articles** | | | | |
| Total records | 15 | Articles: 1086  Preprints: 670  Reports (grey literature): 20 | | |
| Deduplication | 16 | Articles: 944 (excluded doubles and preprints that were indexed in PubMed)  Preprints: 566  Grey literature: 20 | | |
| PRISMA-S: An Extension to the PRISMA Statement for Reporting Literature Searches in Systematic Reviews  Rethlefsen ML, Kirtley S, Waffenschmidt S, Ayala AP, Moher D, Page MJ, Koffel JB, PRISMA-S Group.Last updated February 27, 2020./Updated 21 jan 2010 <https://systematicreviewsjournal.biomedcentral.com/articles/10.1186/s13643-020-01542-z#Sec3> | | | | |
