# Supplementary material for: Computational models predicting the early development of the COVID-19 pandemic in Sweden: systematic review, data synthesis, and secondary validation of accuracy
Source: Sci Rep. 2022 Aug 2;12:13256. doi: 10.1038/s41598-022-16159-6 (PMC9345013; doi:10.1038/s41598-022-16159-6)
Supplement: Supplementary file 4 — Supplementary Information 4. [file 41598_2022_16159_MOESM4_ESM.docx]

# Supplementary Material 4. Complete literature list

## Scientific publications n=892

1.     (2020). "Correction to: Early dinner or “dinner like a pauper”: Evidence, the habitual time of the largest meal of the day – dinner – is predisposing to severe COVID-19 outcome – death (Chronobiology International, (2020), 37, 6, (804-808), 10.1080/07420528.2020.1772810)." Chronobiology International **37**(6): 952.

2.      (2020). "Note from the editors: Don't stop thinking about tomorrow." Eurosurveillance **25**(1).

3.      (2020). "Rapid risk assessment from ECDC: Resurgence of reported cases of COVID-19 in the EU/EEA, the UK and EU candidate and potential candidate countries." Eurosurveillance **25**(26).

4.      (2020). "The first wave of the COVID-19 pandemic in Spain: characterisation of cases and risk factors for severe outcomes, as at 27 April 2020." Euro surveillance : bulletin Europeen sur les maladies transmissibles = European communicable disease bulletin **25**(50).

5.      (2020). "Updated rapid risk assessment from ECDC on coronavirus disease 2019 (COVID-19) pandemic: increased transmission in the EU/EEA and the UK." Eurosurveillance **25**(12).

6.      (2020). Eurosurveillance 10.2807/1560-7917.ES.2020.25.50.2001431 "COVID-19 and lessons learned from the pandemic wave of meningococcal meningitis (19851990)."  **20**(1).

7.      Abd-Elaziz, K., et al. (2021). "Revisiting matrix metalloproteinase 12: its role in pathophysiology of asthma and related pulmonary diseases." Curr Opin Pulm Med **27**(1): 54-60.

8.      Abrucio, F. L., et al. (2020). "Combating covid-19 under bolsonaro’s federalism: A case of intergovernmental incoordination." Revista de Administracao Publica **54**(4): 663-677.

9.      Achdut, N. and T. Refaeli (2020). "Unemployment and psychological distress among young people during the covid‐19 pandemic: Psychological resources and risk factors." International Journal of Environmental Research and Public Health **17**(19): 1-21.

10.    Adams, L., et al. (2020). "Australia can use population level mobility data to fight COVID-19." Medical Journal of Australia **213**(7): 296-297.e291.

11.    Adarkwah, M. A. (2020). "“I’m not against online teaching, but what about us?”: ICT in Ghana post Covid-19." Education and Information Technologies.

12.    Adiga, A., et al. (2020). "Mathematical Models for COVID-19 Pandemic: A Comparative Analysis." Journal of the Indian Institute of Science **100**(4): 793-807.

13.    Adlhoch, C. and R. Pebody (2020). "What to expect for the influenza season 2020/21 with the ongoing COVID-19 pandemic in the World Health Organization European Region." Euro Surveill **25**(42).

14.    Adongo, C. A., et al. (2021). "Beyond fragmentary: A proposed measure for travel vaccination concerns." Tourism Management **83**.

15.    Ahlbom, A. (2020). "Epidemiology is about disease in populations." European Journal of Epidemiology **35**(12): 1111-1113.

16.    Ahlén, G., et al. (2020). "The SARS-CoV-2 N protein is a good component in a vaccine." Journal of Virology **94**(18).

17.    Ahlsson, A. (2020). "Why change? Lessons in leadership from the COVID-19 pandemic." European Journal of Cardio-thoracic Surgery **58**(3): 411-413.

18.    Ahmadpour, D., et al. (2020). "Impact of circulating SARS-CoV-2 mutant G614 on the COVID-19 pandemic." Iranian Journal of Kidney Diseases **14**(5): 331-334.

19.    Ahmed, A., et al. (2020). "COVID-19 and Financial Markets: The Stories of Several Countries." SSRN.

20.    Ahmed, H. U., et al. (2020). "PMD27 Cost Comparison of Surgical Interventions to TREAT Lower Urinary TRACT Symptoms (LUTS) Secondary to Benign Prostatic Hyperplasia (BPH) in the UK, Sweden, and South Africa." Value in Health **23**: S580.

21.    Ahmed, M. (2020). "Introduction to Modern Climate Change. Andrew E. Dessler: Cambridge University Press, 2011, 252 pp, ISBN-10: 0521173159." Science of the Total Environment **734**.

22.    Ahorsu, D. K., et al. (2020). "Associations Between Fear of COVID-19, Mental Health, and Preventive Behaviours Across Pregnant Women and Husbands: An Actor-Partner Interdependence Modelling." Int J Ment Health Addict: 1-15.

23.    Ahorsu, D. K., et al. (2020). "The Association Between Health Status and Insomnia, Mental Health, and Preventive Behaviors: The Mediating Role of Fear of COVID-19." Gerontology and Geriatric Medicine **6**.

24.    Ahrenfeldt, L. J., et al. (2020). "Sex and age differences in COVID-19 mortality in Europe." Wiener Klinische Wochenschrift.

25.    Akour, A., et al. (2020). "The Impact of the COVID-19 Pandemic and Emergency Distance Teaching on the Psychological Status of University Teachers: A Cross-Sectional Study in Jordan." The American journal of tropical medicine and hygiene **103**(6): 2391-2399.

26.    Al Awaidy, S. T., et al. (2020). "Addressing influenza vaccination in MENA region during the COVID-19 pandemic: Decreasing the effects of the collision." Oman Medical Journal **35**(6): 1-3.

27.    Aldaco, R., et al. (2020). "Food waste management during the COVID-19 outbreak: a holistic climate, economic and nutritional approach." Science of the Total Environment **742**.

28.    Alexander, C., et al. (2020). "A Counterfactual Economic Analysis of Covid-19 Using a Threshold Augmented Multi-Country Model." SSRN.

29.    Älgå, A., et al. (2020). "Analysis of Scientific Publications During the Early Phase of the COVID-19 Pandemic: Topic Modeling Study." J Med Internet Res **22**(11): e21559.

30.    Al-Hasan, A., et al. (2020). "Threat, coping, and social distance adherence during COVID-19: Cross-continental comparison using an online cross-sectional survey." Journal of Medical Internet Research **22**(11).

31.    Ali Maher, O. and S. Bellizzi (2020). "Pandemic declaration, definition versus process." Public Health **185**: 265.

32.    Alkhamis, M. A., et al. (2020). "Spatiotemporal dynamics of the COVID-19 pandemic in the State of Kuwait." International Journal of Infectious Diseases **98**: 153-160.

33.    Alm, E., et al. (2020). "Geographical and temporal distribution of SARS-CoV-2 clades in the WHO European Region, January to June 2020." Euro Surveill **25**(32).

34.    Almqvist, J., et al. (2020). "Neurological manifestations of coronavirus infections – a systematic review." Annals of Clinical and Translational Neurology **7**(10): 2057-2071.

35.    Almskog, L. M., et al. (2020). "Rotational thromboelastometry results are associated with care level in COVID-19." Journal of Thrombosis and Thrombolysis.

36.    Alonso-Fernandez, F., et al. (2020). Soft-Biometrics Estimation in the Era of Facial Masks. BIOSIG 2020 - Proceedings of the 19th International Conference of the Biometrics Special Interest Group.

37.    Amaku, M., et al. (2021). "Modelling the test, trace and quarantine strategy to control the COVID-19 epidemic in the state of São Paulo, Brazil." Infect Dis Model **6**: 46-55.

38.    Ameis, S. H., et al. (2020). "Coping, fostering resilience, and driving care innovation for autistic people and their families during the COVID-19 pandemic and beyond." Molecular Autism **11**(1).

39.    Amér, S., et al. (2020). "Almost two-thirds of the elderly with covid-19 surviving in nursing homes." Lakartidningen **117**.

40.    Amini Moghadam, S., et al. (2020). "Clinical features of pregnant women in Iran who died due to COVID-19." International Journal of Gynecology and Obstetrics.

41.    Aminizadeh, M., et al. (2020). "Hospital preparedness challenges in biological disasters: A qualitative study." Disaster Med Public Health Prep: 1-13.

42.    Ammon, A. (2020). "Contributing to health security in Europe since 2005 – ECDC’s 15th anniversary." Eurosurveillance **25**(20).

43.    Andersson, E. and A. Sönnerborg (2020). "[Future testing for SARS-CoV-2: not only more but smarter]." Lakartidningen **117**.

44.    Andrius, K., et al. (2020). "Swedish Policy Analysis for COVID-19." SSRN.

45.    Angelino, E., et al. (2020). "Risk communication during the COVID-19 pandemic: Lessons for lifestyle interventions in cardiovascular prevention." Giornale Italiano di Cardiologia **21**(6): 401-407.

46.    Ankarali, H., et al. (2020). "Modeling and short-term forecasts of indicators for COVID-19 outbreak in 25 countries at the end of march." Bangladesh J. Med. Sci. **19**(Special issue): 6-20.

47.    Arnold, M. and I. Kerridge (2020). "Accelerating the De-Personalization of Medicine: The Ethical Toxicities of COVID-19." Journal of Bioethical Inquiry.

48.    Arzate-Mejía, R. G., et al. (2020). "Long-Term Impact of Social Isolation and Molecular Underpinnings." Frontiers in Genetics **11**.

49.    Asadikia, A., et al. (2020). "Systematic prioritisation of SDGs: Machine learning approach." World Development.

50.    Aschwanden, C. (2020). "The false promise of herd immunity for COVID-19." Nature **587**(7832): 26-28.

51.    Asif, S., et al. (2020). "Weak anti-SARS-CoV-2 antibody response is associated with mortality in a Swedish cohort of COVID-19 patients in critical care." Critical Care **24**(1).

52.    Atkinson, K. M., et al. (2020). "The digital immunization system of the future: imagining a patient-centric, interoperable immunization information system." Therapeutic Advances in Vaccines and Immunotherapy **8**.

53.    Awad, M. E., et al. (2020). "Modeling of the adsorption of a protein-fragment on kaolinite with potential antiviral activity." Appl Clay Sci **199**: 105865.

54.    Azoulay, E., et al. (2020). "Symptoms of burnout in intensive care unit specialists facing the COVID-19 outbreak." Annals of Intensive Care **10**(1).

55.    Azzam, D. B., et al. (2020). "Oculofacial plastic surgery-related online search trends including the impact of the COVID-19 pandemic." Orbit (London).

56.    Baidya, A., et al. (2020). "Diabetes and covid-19: A review." Journal of the ASEAN Federation of Endocrine Societies **35**(1): 40-48.

57.    Baker, M. G., et al. (2020). "Estimating the burden of United States workers exposed to infection or disease: A key factor in containing risk of COVID-19 infection." PLoS One **15**(4).

58.    Bakker, A., et al. (2020). "E-health applications in the field of traumatic stress." European Journal of Psychotraumatology **11**(1).

59.    Balakrishnan, A., et al. (2020). "Delivery of hepato-pancreato-biliary surgery during the COVID-19 pandemic: an European-African Hepato-Pancreato-Biliary Association (E-AHPBA) cross-sectional survey." HPB **22**(8): 1128-1134.

60.    Bambra, C., et al. (2020). "COVID-19 and the gender health paradox." Scandinavian journal of public health: 1403494820975604.

61.    Banerjee, S., et al. (2020). "The impact of COVID-19 on oncology professionals: Initial results of the ESMO resilience task force survey collaboration." Annals of Oncology **31**: S1200-S1201.

62.    Barakat, S., et al. (2020). A Three-Stage Periodic Model: An Initial Analysis of Government Preventive Measures against COVID-19 Epidemics. ACM International Conference Proceeding Series.

63.    Baral, R., et al. (2020). "Effect of Renin-Angiotensin-Aldosterone System Inhibitors in Patients with COVID-19: a Systematic Review and Meta-analysis of 28,872 Patients." Current Atherosclerosis Reports **22**(10).

64.    Baral, S., et al. (2020). "Leveraging epidemiological principles to evaluate Sweden's COVID-19 response." Ann Epidemiol **54**: 21-26.

65.    Bardosh, K. L., et al. (2020). "Integrating the social sciences in epidemic preparedness and response: A strategic framework to strengthen capacities and improve Global Health security." Globalization and Health **16**(1).

66.    Barone, S., et al. (2020). "Building a statistical surveillance dashboard for COVID-19 infection worldwide." Quality Engineering **32**(4): 754-763.

67.    Barrett, P. M., et al. (2020). "Measuring the effectiveness of an automated text messaging active surveillance system for COVID-19 in the south of Ireland, March to April 2020." Eurosurveillance **25**(23).

68.    Bartos, M. (2020). "Australia and the rhythm of the COVID-19 epidemic." Asia-Pasific Journal: Japan Focus **18**(14): 1-8.

69.    Bartoszek, K., et al. (2020). "Are official confirmed cases and fatalities counts good enough to study the COVID-19 pandemic dynamics? A critical assessment through the case of Italy." Nonlinear Dyn: 1-29.

70.    Bastard, P., et al. (2020). "Autoantibodies against type I IFNs in patients with life-threatening COVID-19." Science **370**(6515).

71.    Battisti, N. M. L., et al. (2020). "Adapting care for older cancer patients during the COVID-19 pandemic: Recommendations from the International Society of Geriatric Oncology (SIOG) COVID-19 Working Group." J Geriatr Oncol **11**(8): 1190-1198.

72.    Bauer, J., et al. (2020). "Access to intensive care in 14 European countries: a spatial analysis of intensive care need and capacity in the light of COVID-19." Intensive Care Medicine.

73.    Bauer, W., et al. (2020). "Outcome prediction by serum calprotectin in patients with COVID-19 in the emergency department." J Infect.

74.    Beauté, J. and G. Spiteri (2020). "Travel-associated COVID-19: a challenge for surveillance?" Euro Surveill **25**(37).

75.    Beck, B. R., et al. (2020). "Predicting commercially available antiviral drugs that may act on the novel coronavirus (SARS-CoV-2) through a drug-target interaction deep learning model." Computational and Structural Biotechnology Journal **18**: 784-790.

76.    Bedford, J., et al. (2020). "COVID-19: towards controlling of a pandemic." The Lancet **395**(10229): 1015-1018.

77.    Bedford, J., et al. (2020). "Living with the COVID-19 pandemic: act now with the tools we have." The Lancet **396**(10259): 1314-1316.

78.    Begić, D., et al. (2020). "Mental health of physicians in Croatia during the COVID-19 pandemic." Lijecnicki Vjesnik **142**(78): 189-198.

79.    Behrouzi, B., et al. (2020). "Influenza Vaccination to Reduce Cardiovascular Morbidity and Mortality in Patients With COVID-19: JACC State-of-the-Art Review." Journal of the American College of Cardiology **76**(15): 1777-1794.

80.    Belli, L. S., et al. (2020). "Protective role of tacrolimus, deleterious role of age and comorbidities in liver transplant recipients with Covid-19: results from the ELITA/ELTR multi-center European study." Gastroenterology.

81.    Belokrinitskaya, T. E., et al. (2020). "Characteristics of the COVID-19 in pregnant women of the far east and Siberia." Russian Journal of Human Reproduction **26**(3): 85-91.

82.    Belot, A., et al. (2020). "SARS-CoV-2-related paediatric inflammatory multisystem syndrome, an epidemiological study, France, 1 March to 17 May 2020." Eurosurveillance **25**(22).

83.    Benskin, L. L. (2020). "A Basic Review of the Preliminary Evidence That COVID-19 Risk and Severity Is Increased in Vitamin D Deficiency." Frontiers in public health **8**.

84.    Berdud, M., et al. (2020). "PNS99 A THEORY ON ICER PRICING AND OPTIMAL LEVEL OF COST-EFFECTIVENESS THRESHOLD." Value in Health **23**: S301-S302.

85.    Bergquist, R. and L. Rinaldi (2020). "Covid-19: Pandemonium in our time." Geospatial health **15**(1).

86.    Bernstein, J. (2020). "Not the Last Word: How Necessary COVID-19 Lockdowns Can Go Too Far." Clinical Orthopaedics and Related Research **478**(8): 1719-1724.

87.    Berzuini, C., et al. (2020). "Value of dynamic clinical and biomarker data for mortality risk prediction in COVID-19: a multicentre retrospective cohort study." BMJ Open **10**(9): e041983.

88.    Best, J. H., et al. (2020). "Baseline Demographics and Clinical Characteristics Among 3471 US Patients Hospitalized with COVID-19 and Pulmonary Involvement: A Retrospective Study." Advances in Therapy.

89.    Bhanot, G. and C. DeLisi (2020). "Analysis of Covid-19 Data for Eight European Countries and the United Kingdom Using a Simplified SIR Model." Res Sq.

90.    Bhaskar, S., et al. (2020). "At the Epicenter of COVID-19-the Tragic Failure of the Global Supply Chain for Medical Supplies." Front Public Health **8**: 562882.

91.    Bialer, M., et al. (2020). "Progress report on new antiepileptic drugs: A summary of the Fifteenth Eilat Conference on New Antiepileptic Drugs and Devices (EILAT XV). I. Drugs in preclinical and early clinical development." Epilepsia **61**(11): 2340-2364.

92.    Bilinski, A. and E. J. Emanuel (2020). "COVID-19 and Excess All-Cause Mortality in the US and 18 Comparison Countries." JAMA - Journal of the American Medical Association **324**(20): 2100-2102.

93.    Biswal, A., et al. (2020). "COVID-19 lockdown and its impact on tropospheric NO2 concentrations over India using satellite-based data." Heliyon **6**(9).

94.    Bivins, A., et al. (2020). "Wastewater-Based Epidemiology: Global Collaborative to Maximize Contributions in the Fight Against COVID-19." Environ Sci Technol **54**(13): 7754-7757.

95.    Blanco-Arana, M. C. (2020). "Socio-economic factors on the evolution of mortality in Europe in the XXI century: Policy proposals to face the COVID-19 crisis." Revista de Economia Mundial **2020**(56): 86-100.

96.    Blangiardo, M., et al. (2020). "Estimating weekly excess mortality at subnational level in Italy during the COVID-19 pandemic." PLoS One **15**(10).

97.    Blomström-Lundqvist, C. (2020). "Effects of COVID-19 lockdown strategies on management of atrial fibrillation." European Heart Journal **41**(32): 3080-3082.

98.    Boëlle, P. Y., et al. (2020). "Excess cases of influenza-like illnesses synchronous with coronavirus disease (COVID-19) epidemic, France, March 2020." Eurosurveillance **25**(14).

99.    Bogogiannidou, Z., et al. (2020). "Repeated leftover serosurvey of SARS-CoV-2 IgG antibodies, Greece, March and April 2020." Eurosurveillance **25**(31): 1-6.

100. Boncz, I., et al. (2020). "PIN112 Geographical Inequalities of the Incidence of Coronavirus Disease 2019 (COVID-19) Caused By Sars-COV-2 VIRUS in the European Union." Value in Health **23**: S562.

101. Bordi, L., et al. (2020). "Differential diagnosis of illness in patients under investigation for the novel coronavirus (SARS-CoV-2), Italy, February 2020." Eurosurveillance **25**(8).

102. Boretti, A. (2020). "After Less Than 2 Months, the Simulations That Drove the World to Strict Lockdown Appear to be Wrong, the Same of the Policies They Generated." Health Serv Res Manag Epidemiol **7**: 2333392820932324.

103. Borges do Nascimento, I. J., et al. (2020). "Novel Coronavirus Infection (COVID-19) in Humans: A Scoping Review and Meta-Analysis." J Clin Med **9**(4).

104. Borkowski, P., et al. (2021). "Lockdowned: Everyday mobility changes in response to COVID-19." Journal of Transport Geography **90**.

105. Bose, S., et al. (2020). "Atypical Presentation of Novel Coronavirus Disease 2019 in a Peritoneal Dialysis Patient." Journal of Investigative Medicine High Impact Case Reports **8**.

106. Bousquet, J., et al. (2020). "Spices to control COVID-19 symptoms: Yes, but not only." International Archives of Allergy and Immunology.

107. Bowen, J. D., et al. (2020). "COVID-19 in MS: Initial observations from the Pacific Northwest." Neurology(R) neuroimmunology & neuroinflammation **7**(5).

108. Brainard, J., et al. (2020). "Community use of face masks and similar barriers to prevent respiratory illness such as COVID-19: A rapid scoping review." Eurosurveillance **25**(49).

109. Bram, J. T., et al. (2020). "Where Have All the Fractures Gone? The Epidemiology of Pediatric Fractures during the COVID-19 Pandemic." Journal of Pediatric Orthopaedics **40**(8): 373-379.

110. Brandenburg, A. (2020). "Piecewise quadratic growth during the 2019 novel coronavirus epidemic." Infect Dis Model **5**: 681-690.

111. Brandenburg, J. E., et al. (2020). "Why individuals with cerebral palsy are at higher risk for respiratory complications from COVID-19." Journal of Pediatric Rehabilitation Medicine **13**(3): 317-327.

112. Brandl, M., et al. (2020). "Mass gathering events and undetected transmission of SARS-CoV-2 in vulnerable populations leading to an outbreak with high case fatality ratio in the district of Tirschenreuth, Germany." Epidemiol Infect **148**: e252.

113. Brar, G., et al. (2020). "COVID-19 Severity and Outcomes in Patients With Cancer: A Matched Cohort Study." J Clin Oncol **38**(33): 3914-3924.

114. Brar, G., et al. (2020). "COVID-19 severity and outcomes inhospitalized patients with cancer at a New York Citytertiary medical center: A matched cohort study." Clinical Cancer Research **26**(18 SUPPL).

115. Brenner, H., et al. (2020). "Vitamin D insufficiency and deficiency and mortality from respiratory diseases in a cohort of older adults: Potential for limiting the death toll during and beyond the COVID-19 pandemic?" Nutrients **12**(8): 1-11.

116. Brett, T. S. and P. Rohani (2020). "Transmission dynamics reveal the impracticality of COVID-19 herd immunity strategies." Proceedings of the National Academy of Sciences of the United States of America **117**(41): 25897-25903.

117. Britton, T., et al. (2020). "A mathematical model reveals the influence of population heterogeneity on herd immunity to SARS-CoV-2." Science **369**(6505): 846-849.

118. Brüssow, H. (2020). "COVID-19: test, trace and isolate-new epidemiological data." Environmental Microbiology **22**(7): 2445-2456.

119. Bryant, P. and A. Elofsson (2020). "Estimating the impact of mobility patterns on COVID-19 infection rates in 11 European countries." PeerJ **8**: e9879.

120. Buemann, B., et al. (2020). "Can intravenous oxytocin infusion counteract hyperinflammation in COVID-19 infected patients?" World J Biol Psychiatry: 1-12.

121. Bullerdiek, J. (2020). "Blood type A associated with critical COVID-19 and death in a Swedish cohort - A critical comment." Critical Care **24**(1).

122. Burn, E., et al. (2020). "Deep phenotyping of 34,128 adult patients hospitalised with COVID-19 in an international network study." Nature Communications **11**(1).

123. Burns, J., et al. (2020). "Travel‐related control measures to contain the COVID‐19 pandemic: a rapid review." Cochrane Database of Systematic Reviews(9).

124. Burzynska, K. and G. Contreras (2020). "Gendered effects of school closures during the COVID-19 pandemic." The Lancet **395**(10242): 1968.

125. Buscema, P. M., et al. (2020). "COVID-19 in Italy and extreme data mining." Physica A: Statistical Mechanics and its Applications **557**.

126. Byass, P. (2020). "Eco-epidemiological assessment of the COVID-19 epidemic in China, January-February 2020." Glob Health Action **13**(1): 1760490.

127. Caini, S., et al. (2020). "Meta-analysis of diagnostic performance of serological tests for SARS-CoV-2 antibodies up to 25 April 2020 and public health implications." Eurosurveillance **25**(23).

128. Calderón-Larrañaga, A., et al. (2020). "COVID-19: risk accumulation among biologically and socially vulnerable older populations." Ageing Research Reviews **63**.

129. Calderón-Larrañaga, A., et al. (2020). "High excess mortality in areas with young and socially vulnerable populations during the COVID-19 outbreak in Stockholm Region, Sweden." BMJ Glob Health **5**(10).

130. Cameli, M., et al. (2020). "Safe performance of echocardiography during the COVID-19 pandemic: a practical guide." Rev Cardiovasc Med **21**(2): 217-223.

131. Cameron, E. E., et al. (2020). "Maternal psychological distress & mental health service use during the COVID-19 pandemic." Journal of Affective Disorders **276**: 765-774.

132. Campedelli, G. M., et al. (2020). "Disentangling community-level changes in crime trends during the COVID-19 pandemic in Chicago." Crime Science **9**(1).

133. Cannarella, R., et al. (2020). "Systemic effects of the hormonal treatment of male hypogonadism with preliminary indications for the management of COVID-19 patients." Therapeutic Advances in Endocrinology and Metabolism **11**.

134. Cao, Y., et al. (2020). "COVID-19 case-fatality rate and demographic and socioeconomic influencers: worldwide spatial regression analysis based on country-level data." BMJ Open **10**(11): e043560.

135. Cappellano, F. and J. Kurowska-Pysz (2020). "The mission-oriented approach for (cross-border) regional development." Sustainability (Switzerland) **12**(12).

136. Carey, N., et al. (2020). "Exploring views and experiences of how infections are detected and managed in practice by nurses, care workers and manager's in nursing homes in England and Sweden: a survey protocol." BMJ Open **10**(10): e038390.

137. Carli, G., et al. (2020). "Asthma phenotypes, comorbidities, and disease activity in COVID-19: The need of risk stratification. Reply to Morais-Almeida." Allergy: European Journal of Allergy and Clinical Immunology.

138. Casella, F. (2021). "Can the COVID-19 Epidemic Be Controlled on the Basis of Daily Test Reports?" IEEE Control Systems Letters **5**(3): 1079-1084.

139. Cassaniti, I., et al. (2020). "Authors' response: COVID-19: how accurate are seroprevalence studies?" Euro surveillance : bulletin Europeen sur les maladies transmissibles = European communicable disease bulletin **25**(30).

140. Catalano, R., et al. (2020). "Non-COVID-19 deaths after social distancing in Norway." Eur J Epidemiol **35**(11): 1021-1024.

141. Cauchois, R., et al. (2020). "Early IL-1 receptor blockade in severe inflammatory respiratory failure complicating COVID-19." Proceedings of the National Academy of Sciences of the United States of America **117**(32): 18951-18953.

142. Cavalli, G., et al. (2020). "Interleukin-1 blockade with high-dose anakinra in patients with COVID-19, acute respiratory distress syndrome, and hyperinflammation: a retrospective cohort study." The Lancet Rheumatology **2**(6): e325-e331.

143. Cawthorn, D. M., et al. (2020). "The future of sustainability in the context of COVID-19." Ambio.

144. Caze, T., II, et al. (2020). "Management and treatment of concussions via tele-concussion in a pediatric setting: Methodological approach and descriptive analysis." JMIR Pediatrics and Parenting **3**(2).

145. Cerbin-Koczorowska, M., et al. (2020). "Pharmacists’ preparedness to patients education at the time of pandemic—a cross-sectional study with an example of sars-cov-2 outbreak in Poland." International Journal of Environmental Research and Public Health **17**(18): 1-11.

146. Ceulemans, M., et al. (2020). "SARS-CoV-2 infections and impact of the COVID-19 pandemic in pregnancy and breastfeeding: Results from an observational study in primary care in Belgium." International Journal of Environmental Research and Public Health **17**(18): 1-10.

147. Chamberlain, L. R., et al. (2020). "Therapist-supported online interventions for children and young people with tic disorders: Lessons learned from a randomized controlled trial and considerations for future practice." JMIR Mental Health **7**(10).

148. Chan, A. T., et al. (2020). "The COronavirus Pandemic Epidemiology (COPE) Consortium: A Call to Action." Cancer Epidemiol Biomarkers Prev **29**(7): 1283-1289.

149. Chan, D. K. C., et al. (2020). "Why People Failed to Adhere to COVID-19 Preventive Behaviors? Perspectives from an Integrated Behavior Change Model." Infection Control and Hospital Epidemiology.

150. Chandler, R. E. (2020). "Optimizing safety surveillance for COVID-19 vaccines." Nat Rev Immunol **20**(8): 451-452.

151. Chandler, R. E., et al. (2020). "The Role of Pharmacovigilance and ISoP During the Global COVID-19 Pandemic." Drug Safety **43**(6): 511-512.

152. Chang, K. C., et al. (2020). "Factors related to preventive COVID-19 infection behaviors among people with mental illness." J Formos Med Assoc **119**(12): 1772-1780.

153. Charles, C. M., et al. (2020). "The SARS-CoV-2 pandemic scenario in Africa: What should be done to address the needs of pregnant women?" International Journal of Gynecology and Obstetrics **151**(3): 468-470.

154. Chauhan, R. P., et al. (2020). "Systematic Review of Important Viral Diseases in Africa in Light of the 'One Health' Concept." Pathogens **9**(4).

155. Chen, H., et al. (2021). "Collective self-esteem and perceived stress among the non-infected general public in China during the 2019 coronavirus pandemic: A multiple mediation model." Personality and Individual Differences **168**.

156. Cheng, A., et al. (2020). "Diagnostic performance of initial blood urea nitrogen combined with D-dimer levels for predicting in-hospital mortality in COVID-19 patients." Int J Antimicrob Agents **56**(3): 106110.

157. Chernozhukov, V., et al. (2020). "Causal impact of masks, policies, behavior on early covid-19 pandemic in the U.S." Journal of Econometrics.

158. Chernyshov, P. V., et al. (2020). "Position statement of the European Academy of Dermatology and Venereology Task Force on Quality of Life and Patient Oriented Outcomes on quality of life issues in dermatologic patients during the COVID-19 pandemic." Journal of the European Academy of Dermatology and Venereology **34**(8): 1666-1671.

159. Chieffo, A., et al. (2020). "EAPCI position statement on invasive management of acute coronary syndromes during the COVID-19 pandemic." EuroIntervention **16**(3): 233-246.

160. Chintala, S., et al. (2020). "COVID-19 spatiotemporal research with workflow-based data analysis." Infection, genetics and evolution : journal of molecular epidemiology and evolutionary genetics in infectious diseases **88**: 104701.

161. Chiruţă, C., et al. (2020). "Comparison of the evolution of the COVID‐19 disease between Romania and Italy." Applied System Innovation **3**(4): 1-21.

162. Choi, E. Y., et al. (2020). "Wnt5a and Wnt11 as acute respiratory distress syndrome biomarkers for severe acute respiratory syndrome coronavirus 2 patients." European Respiratory Journal **56**(5).

163. Ciabattini, A., et al. (2020). "Shelter from the cytokine storm: pitfalls and prospects in the development of SARS-CoV-2 vaccines for an elderly population." Seminars in Immunopathology **42**(5): 619-634.

164. Clark, A., et al. (2020). "Global, regional, and national estimates of the population at increased risk of severe COVID-19 due to underlying health conditions in 2020: a modelling study." The Lancet Global Health **8**(8): e1003-e1017.

165. Clemente, V., et al. (2020). "Deubiquitinating enzymes in coronaviruses and possible therapeutic opportunities for COVID-19." International Journal of Molecular Sciences **21**(10).

166. Cohen, J. B., et al. (2020). "Randomized elimination and prolongation of ACE inhibitors and ARBs in coronavirus 2019 (REPLACE COVID) Trial Protocol." Journal of Clinical Hypertension **22**(10): 1780-1788.

167. Colaneri, M., et al. (2020). "Clinical characteristics of coronavirus disease (COVID-19) early findings from a teaching hospital in Pavia, North Italy, 21 to 28 February 2020." Eurosurveillance **25**(16).

168. Copiello, S. and C. Grillenzoni (2020). "The spread of 2019-nCoV in China was primarily driven by population density. Comment on “Association between short-term exposure to air pollution and COVID-19 infection: Evidence from China” by Zhu et al." Science of the Total Environment **744**.

169. Córdoba-Cabús, A., et al. (2020). "Data journalism during the COVID-19 health crisis in the Spanish press." RISTI - Revista Iberica de Sistemas e Tecnologias de Informacao **2020**(E35): 325-337.

170. Corrêa Giron, C., et al. (2020). "On the interactions of the receptor-binding domain of SARS-CoV-1 and SARS-CoV-2 spike proteins with monoclonal antibodies and the receptor ACE2." Virus Research **285**.

171. Crameri, G. A. G., et al. (2020). "Reduced maximal aerobic capacity after COVID-19 in young adult recruits, Switzerland, May 2020." Eurosurveillance **25**(36).

172. Credit, K. (2020). "Neighbourhood inequity: Exploring the factors underlying racial and ethnic disparities in COVID-19 testing and infection rates using ZIP code data in Chicago and New York." Regional Science Policy and Practice **12**(6): 1249-1271.

173. Cross, M., et al. (2020). "Trading health for wealth: The effect of COVID-19 response stringency." International Journal of Environmental Research and Public Health **17**(23): 1-15.

174. D’Angelo, F., et al. (2020). "Management of Traumatology Patients During the Coronavirus (COVID-19) Pandemic: Experience in a Hub Trauma Hospital in Northern Italy." Indian Journal of Orthopaedics **54**: 397-402.

175. D’ettorre, G., et al. (2020). "Post-traumatic stress disorder symptoms in healthcare workers: A ten-year systematic review." Acta Biomedica **91**(12-S): 1-10.

176. D’Souza, R., et al. (2020). "A critical review of the pathophysiology of thrombotic complications and clinical practice recommendations for thromboprophylaxis in pregnant patients with COVID-19." Acta Obstetricia et Gynecologica Scandinavica **99**(9): 1110-1120.

177. Daluwathumullagamage, D. J. and A. Sims (2020). "Blockchain-enabled corporate governance and regulation." International Journal of Financial Studies **8**(2): 1-41.

178. D'Amario, D., et al. (2020). "Experience of remote cardiac care during the COVID-19 pandemic: the V-LAP™ device in advanced heart failure." European Journal of Heart Failure **22**(6): 1050-1052.

179. Damas, J., et al. (2020). "Broad host range of SARS-CoV-2 predicted by comparative and structural analysis of ACE2 in vertebrates." Proc Natl Acad Sci U S A **117**(36): 22311-22322.

180. Danchenko, N., et al. (2020). "PND10 INDIRECT TREATMENT COMPARISON OF BOTULINUM TOXINS A FOR THE TREATMENT OF PEDIATRIC UPPER LIMB SPASTICITY." Value in Health **23**: S260.

181. Dandekar, R., et al. (2020). "A Machine Learning-Aided Global Diagnostic and Comparative Tool to Assess Effect of Quarantine Control in COVID-19 Spread." Patterns.

182. Danielson, M. and L. Ekenberg (2020). Automatic Criteria Weight Generation for Multi-criteria Decision Making Under Uncertainty. Lecture Notes in Business Information Processing. **405:** 1-14.

183. Danis, K., et al. (2020). "High impact of COVID-19 in long-term care facilities, suggestion for monitoring in the EU/EEA, May 2020." Euro Surveill **25**(22).

184. Das, O., et al. (2020). "The need for fully bio-based facemasks to counter coronavirus outbreaks: A perspective." Science of the Total Environment **736**.

185. Daughton, C. G. (2020). "Wastewater surveillance for population-wide Covid-19: The present and future." Science of the Total Environment **736**.

186. Davis, P. A., et al. (2020). "Written Emotional Disclosure Can Promote Athletes' Mental Health and Performance Readiness During the COVID-19 Pandemic." Front Psychol **11**: 599925.

187. de Avila, M. A. G., et al. (2020). "Children’s anxiety and factors related to the covid-19 pandemic: An exploratory study using the children’s anxiety questionnaire and the numerical rating scale." International Journal of Environmental Research and Public Health **17**(16): 1-13.

188. de Chaisemartin, C. and L. de Chaisemartin (2020). "BCG vaccination in infancy does not protect against COVID-19. Evidence from a natural experiment in Sweden." Clinical Infectious Diseases.

189. de Figueiredo, C. S., et al. (2020). "COVID-19 pandemic impact on children and adolescents' mental health: Biological, environmental, and social factors." Progress in Neuro-Psychopharmacology and Biological Psychiatry.

190. De Jong, A., et al. (2020). "How to ventilate obese patients in the ICU." Intensive Care Medicine.

191. de las Heras-Pedrosa, C., et al. (2020). "Sentiment analysis and emotion understanding during the COVID-19 pandemic in Spain and its impact on digital ecosystems." International Journal of Environmental Research and Public Health **17**(15): 1-22.

192. De Leon, J., et al. (2020). "A Rational Use of Clozapine Based on Adverse Drug Reactions, Pharmacokinetics, and Clinical Pharmacopsychology." Psychotherapy and Psychosomatics **89**(4): 200-214.

193. de Melo, G. C. and K. C. G. M. de Araújo (2020). "COVID-19 infection in pregnant women, preterm delivery, birth weight, and vertical transmission: A systematic review and meta-analysis." Cadernos de Saude Publica **36**(7).

194. De Piero, M. E., et al. (2020). "Has Venoarterial ECMO Been Underutilized in COVID-19 Patients?" Innovations: Technology and Techniques in Cardiothoracic and Vascular Surgery **15**(4): 317-321.

195. de Sousa, E., et al. (2020). "Mortality in COVID-19 disease patients: Correlating the association of major histocompatibility complex (MHC) with severe acute respiratory syndrome 2 (SARS-CoV-2) variants." International Journal of Infectious Diseases **98**: 454-459.

196. de Vito, A., et al. (2020). "Epidemiology, clinical aspects, laboratory diagnosis and treatment of rickettsial diseases in the mediterranean area during COVID-19 pandemic: A review of the literature." Mediterranean Journal of Hematology and Infectious Diseases **12**(1).

197. Dehghan Shabani, Z. and R. Shahnazi (2020). "Spatial distribution dynamics and prediction of COVID-19 in Asian countries: spatial Markov chain approach." Regional Science Policy and Practice **12**(6): 1005-1025.

198. Del Castillo, R., et al. (2020). "Low-dose radiotherapy for COVID-19 pneumonia treatment: case report, procedure, and literature review." Strahlentherapie und Onkologie **196**(12): 1086-1093.

199. Delavari, S., et al. (2020). "Impact of SARS-CoV-2 Pandemic on Patients with Primary Immunodeficiency." Journal of Clinical Immunology.

200. Dickson, E. M., et al. (2020). "Do point-of-care tests (POCTs) offer a new paradigm for the management of patients with influenza?" Euro Surveill **25**(44).

201. Diep, P. T., et al. (2020). "Oxytocin, a possible treatment for COVID-19? Everything to gain, nothing to lose." Clinical Neuropsychiatry **17**(3): 192-195.

202. Dignum, F., et al. (2020). "Analysing the Combined Health, Social and Economic Impacts of the Corovanvirus Pandemic Using Agent-Based Social Simulation." Minds Mach (Dordr): 1-18.

203. Dirk, K., et al. (2020). "Macroeconomic Dynamics and Reallocation in an Epidemic." SSRN.

204. Donaldsson, S., et al. (2020). "COVID-19: minimising contaminated aerosol spreading during CPAP treatment." Arch Dis Child Fetal Neonatal Ed **105**(6): 669-671.

205. Dong, Y. M., et al. (2020). "Development and Validation of a Nomogram for Assessing Survival in Patients with COVID-19 Pneumonia." Clin Infect Dis.

206. Dorofeev, V. P., et al. (2021). Super Intelligence to Solve COVID-19 Problem. Studies in Computational Intelligence. **925 SCI:** 293-300.

207. Dovey, Z., et al. (2020). "Impact of COVID-19 on Prostate Cancer Management: Guidelines for Urologists." European Urology Open Science **20**: 1-11.

208. Dowie, J. (2020). "COVID-19, the Swedish 'Experiment', and Me." Stud Health Technol Inform **273**: 211-216.

209. Drefahl, S., et al. (2020). "A population-based cohort study of socio-demographic risk factors for COVID-19 deaths in Sweden." Nature Communications **11**(1).

210. Drew, D. A., et al. (2020). "Cancer and race: Two important riskfactors for COVID-19 incidence as captured by theCOVID Symptom Study real-time epidemiology tool." Clinical Cancer Research **26**(18 SUPPL).

211. Duarte, M. B. O., et al. (2020). "Outcomes of covid-19 patients under cytotoxic cancer chemotherapy in brazil." Cancers **12**(12): 1-13.

212. Dube, R. and S. S. Kar (2020). "COVID-19 in pregnancy: The foetal perspective- A systematic review." BMJ Paediatrics Open **4**(1).

213. Dufour, C., et al. (2020). "Occupational health and safety division of responsibility: A conceptual model for the implementation of the OHSAS 18001:2007 standard." Human Systems Management **39**(4): 549-563.

214. Durrheim, D. N. and M. G. Baker (2020). "COVID-19—a very visible pandemic." The Lancet **396**(10248): e17.

215. Džiugys, A., et al. (2020). "Simplified model of Covid-19 epidemic prognosis under quarantine and estimation of quarantine effectiveness." Chaos, Solitons and Fractals **140**.

216. Ebada, S. S., et al. (2020). "Anti-inflammatory, antiallergic and COVID-19 protease inhibitory activities of phytochemicals from the Jordanian hawksbeard: Identification, structure-Activity relationships, molecular modeling and impact on its folk medicinal uses." RSC Advances **10**(62): 38128-38141.

217. Eck, K. and S. Hatz (2020). "State surveillance and the COVID-19 crisis." Journal of Human Rights **19**(5): 603-612.

218. Effendi, M. I., et al. (2020). "Social Media Adoption in SMEs Impacted by COVID-19: The TOE Model*." Journal of Asian Finance, Economics and Business **7**(11): 915-925.

219. Eikhof, D. R. (2020). "COVID-19, inclusion and workforce diversity in the cultural economy: what now, what next?" Cultural Trends **29**(3): 234-250.

220. Eivazi, S., et al. (2020). "Predicting COVID-19 preventive healthy behaviors based on dysfunctional attitudes in five countries." Journal of Kermanshah University of Medical Sciences **24**(4): 1-7.

221. El Deeb, O. and M. Jalloul (2020). "The dynamics of COVID-19 spread: Evidence from Lebanon." Mathematical Biosciences and Engineering **17**(5): 5618-5632.

222. El Zowalaty, M. E. and J. D. Järhult (2020). "From SARS to COVID-19: A previously unknown SARS- related coronavirus (SARS-CoV-2) of pandemic potential infecting humans – Call for a One Health approach." One Health **9**.

223. El Zowalaty, M. E., et al. (2020). "Environmental impact of the COVID-19 pandemic–a lesson for the future." Infection Ecology and Epidemiology **10**(1).

224. Elbarbary, N. S., et al. (2020). "COVID-19 outbreak and pediatric diabetes: Perceptions of health care professionals worldwide." Pediatric Diabetes **21**(7): 1083-1092.

225. El-Boghdadly, K., et al. (2020). "Risks to healthcare workers following tracheal intubation of patients with COVID-19: a prospective international multicentre cohort study." Anaesthesia **75**(11): 1437-1447.

226. Elhadi, Y. A. M., et al. (2020). "The formidable task of fighting covid-19 in sudan." Pan African Medical Journal **35**: 1-5.

227. Elisa, B., et al. (2020). "The Trade-Off Behaviours between Virtual and Physical Activities during COVID-19 Pandemic Period." SSRN.

228. El-Khatib, Z., et al. (2020). "The Association between Out-of-Pocket Expenditure and COVID-19 Mortality Globally." Journal of epidemiology and global health **10**(3): 192-193.

229. El-Khatib, Z., et al. (2020). "The disproportionate effect of COVID-19 mortality on ethnic minorities: Genetics or health inequalities?" EClinicalMedicine **23**.

230. Erik, A., et al. (2020). "Who Is Still Travelling by Public Transport during COVID-19? Socioeconomic Factors Explaining Travel Behaviour in Stockholm Based on Smart Card Data." SSRN.

231. Esteban-Gonzalo, S., et al. (2020). "Psychosocial Correlates of Mental Health and Well-Being During the COVID-19: The Spanish Case." Frontiers in Psychology **11**.

232. Esteves, S. C., et al. (2020). "SARS-CoV-2 pandemic and repercussions for male infertility patients: a proposal for the individualized provision of andrological services." Andrology.

233. Etard, J. F., et al. (2020). "Potential lethal outbreak of coronavirus disease (COVID-19) among the elderly in retirement homes and long-term facilities, France, March 2020." Eurosurveillance **25**(15).

234. Eurosurveillance Editorial, T. (2020). "Updated rapid risk assessment from ECDC on the novel coronavirus disease 2019 (COVID-19) pandemic: increased transmission in the EU/EEA and the UK." Euro surveillance : bulletin Europeen sur les maladies transmissibles = European communicable disease bulletin **25**(10).

235. Evans, L. M., et al. (2020). "PDB16 A POPULATION-ADJUSTED INDIRECT COMPARISON OF CARDIOVASCULAR BENEFITS BETWEEN ONCE-WEEKLY SEMAGLUTIDE AND DULAGLUTIDE IN PATIENTS WITH TYPE 2 DIABETES AND HIGH CARDIOVASCULAR RISK." Value in Health **23**: S110.

236. Ewing, A. (2020). "COVID-19—a very visible pandemic." The Lancet **396**(10248): e18.

237. Faccincani, R., et al. (2020). "How to surge to face SARS-CoV-2 outbreak. Lessons learned from Lumbardy, Italy." Disaster medicine and public health preparedness.

238. Falk, M. T. and E. Hagsten (2020). "The unwanted free rider: Covid-19." Current Issues in Tourism.

239. Farzaneh, M., et al. (2020). "Pore-Scale Transport and Two-Phase Fluid Structures in Fibrous Porous Layers: Application to Fuel Cells and Beyond." Transp Porous Media: 1-26.

240. Fasano, A., et al. (2020). "Management of Advanced Therapies in Parkinson's Disease Patients in Times of Humanitarian Crisis: The COVID-19 Experience." Movement Disorders Clinical Practice **7**(4): 361-372.

241. Fazeli, S., et al. (2020). "Depression, anxiety, and stress mediate the associations between internet gaming disorder, insomnia, and quality of life during the COVID-19 outbreak." Addictive Behaviors Reports **12**.

242. Feng, S., et al. (2020). "NOx Emission Changes Over China During the COVID-19 Epidemic Inferred From Surface NO2 Observations." Geophysical Research Letters **47**(19).

243. Ferrando, C., et al. (2020). "Clinical features, ventilatory management, and outcome of ARDS caused by COVID-19 are similar to other causes of ARDS." Intensive Care Medicine **46**(12): 2200-2211.

244. Filippini, T., et al. (2020). "Associations between mortality from COVID-19 in two Italian regions and outdoor air pollution as assessed through tropospheric nitrogen dioxide." Sci Total Environ: 143355.

245. Fischer, B., et al. (2020). "SARS-CoV-2 IgG seroprevalence in blood donors located in three different federal states, Germany, March to June 2020." Eurosurveillance **25**(28).

246. Foddai, A., et al. (2020). "Surveillance to improve evidence for community control decisions during the COVID-19 pandemic - Opening the animal epidemic toolbox for Public Health." One Health **9**: 100130.

247. Fokas, A. S., et al. (2020). "Mathematical models and deep learning for predicting the number of individuals reported to be infected with SARS-CoV-2." J R Soc Interface **17**(169): 20200494.

248. Fong, M. W., et al. (2020). "Letter to the editor: COVID-19 cases among school-aged children and school-based measures in Hong Kong, July 2020." Eurosurveillance **25**(37).

249. Fooladi, E. C. (2020). "Between Education and Opinion-Making: Dialogue between Didactic/Didaktik Models from Science Education and Science Communication in the Times of a Pandemic." Sci Educ (Dordr): 1-22.

250. Forte, G., et al. (2020). "COVID-19 pandemic in the italian population: Validation of a post-traumatic stress disorder questionnaire and prevalence of PTSD symptomatology." International Journal of Environmental Research and Public Health **17**(11): 1-16.

251. Fouillet, A., et al. (2020). "Excess all-cause mortality during the first wave of the COVID-19 epidemic in France, March to May 2020." Eurosurveillance **25**(34).

252. Franceschi, V. B., et al. (2020). "Population-based prevalence surveys during the Covid-19 pandemic: A systematic review." Reviews in Medical Virology.

253. Fretheim, A., et al. (2020). "COVID-19: We need randomised trials of school closures." Journal of Epidemiology and Community Health **74**(12): 1078-1079.

254. Fröberg, A. (2020). "The COVID-19 pandemic: The importance of physical activity among faculty members." Journal of American college health : J of ACH: 1-4.

255. Furutani, H., et al. (2020). "Simple method for estimating daily and  total COVID-19 deaths using a Gumbel model." ResearchSquare.

256. Gajdzik, B. (2020). Modelling the impact of investments on energy intensity in the polish steel industry as a step towards industry 4.0. METAL 2020 - 29th International Conference on Metallurgy and Materials, Conference Proceedings.

257. Gallagher, K. P., et al. (2020). "Safety First: Expanding the Global Financial Safety Net in Response to COVID-19." Global Policy.

258. Gámbaro, F., et al. (2020). "Introductions and early spread of SARS-CoV-2 in france, 24 january to 23 march 2020." Eurosurveillance **25**(26).

259. Ganyani, T., et al. (2020). "Estimating the generation interval for coronavirus disease (COVID-19) based on symptom onset data, March 2020." Eurosurveillance **25**(17).

260. Garazzino, S., et al. (2020). "Multicentre Italian study of SARS-CoV-2 infection in children and adolescents, preliminary data as at 10 April 2020." Eurosurveillance **25**(18): 1-4.

261. Garcia de Avila, M. A., et al. (2020). "Children's Anxiety and Factors Related to the COVID-19 Pandemic: An Exploratory Study Using the Children's Anxiety Questionnaire and the Numerical Rating Scale." International Journal of Environmental Research and Public Health **17**(16).

262. Garcia, S., et al. (2020). "Impact of COVID-19 pandemic on STEMI care: An expanded analysis from the United States." Catheter Cardiovasc Interv.

263. Garcia-Olivé, I., et al. (2020). "D-dimer in patients infected with COVID-19 and suspected pulmonary embolism." Respiratory medicine **169**: 106023.

264. García-Salido, A. (2020). "SARS-CoV-2 children transmission: The evidence is that today we do not have enough evidence." Acta Paediatrica, International Journal of Paediatrics **109**(9): 1912.

265. Gary, H. (2020). "Covid-19 USA and World Death Rates Analysis, Modeling, and Recommendations." SSRN.

266. Gémes, K., et al. (2020). "Burden and prevalence of prognostic factors for severe COVID-19 in Sweden." Eur J Epidemiol **35**(5): 401-409.

267. Geng, J., et al. (2020). "A Silent Infection Pandemic of COVID-19: Epidemiological Investigation and Hypothetical Models." Can J Infect Dis Med Microbiol **2020**: 5120253.

268. Gerkin, R. C., et al. (2020). "Recent smell loss is the best predictor of COVID-19 among individuals with recent respiratory symptoms." Chem Senses.

269. Gharaibeh, M. K. and N. K. Gharaibeh (2020). "An empirical study on factors influencing the intention to use mobile learning." Advances in Science, Technology and Engineering Systems **5**(5): 1261-1265.

270. Ghayda, R. A., et al. (2020). "Estimation of global case fatality rate of coronavirus disease 2019 (COVID-19) using meta-analyses: Comparison between calendar date and days since the outbreak of the first confirmed case." Int J Infect Dis **100**: 302-308.

271. Ghisolfi, S., et al. (2020). "Predicted COVID-19 fatality rates based on age, sex, comorbidities and health system capacity." BMJ Global Health **5**(9).

272. Ghosal, S., et al. (2020). "Impact of complete lockdown on total infection and death rates: A hierarchical cluster analysis." Diabetes and Metabolic Syndrome: Clinical Research and Reviews **14**(4): 707-711.

273. Gibson, J. (2020). "Government mandated lockdowns do not reduce Covid-19 deaths: implications for evaluating the stringent New Zealand response." New Zealand Economic Papers.

274. Gidlöf, S., et al. (2020). "COVID-19 in pregnancy with comorbidities: More liberal testing strategy is needed." Acta Obstetricia et Gynecologica Scandinavica **99**(7): 948-949.

275. Giesecke, J. (2020). "The invisible pandemic." The Lancet **395**(10238): e98.

276. Gilles Pech de, L., et al. (2020). "Non Pharmaceutical Interventions ‘NPIs’, Hospital Overload and Excess Mortality: Statistical Analysis and Mathematical Study of the NPIs Results in ‘COVID 19’ Outbreak." SSRN.

277. Gleda, K., et al. (2020). "The Origin, Diffusion and the Comparison of Ode Numerical Solutions Used by SIR Model in Order to Predict SARS-CoV-2 in Nordic Countries." SSRN.

278. Gleissman, H., et al. (2020). "Prone positioning in mechanically ventilated patients with severe acute respiratory distress syndrome and coronavirus disease 2019." Acta Anaesthesiologica Scandinavica.

279. Godman, B. (2020). "Combating covid-19: Lessons learnt particularly among developing countries and the implications." Bangladesh Journal of Medical Science **19**(Special issue): 103-108.

280. Goldman, J. D., et al. (2020). "Impact of baseline alanine aminotransferase levels on the safety and efficacy of remdesivir in severe COVID-19 patients." Hepatology **72**(1 SUPPL): 279A.

281. Goldman, J. D., et al. (2020). "Remdesivir for 5 or 10 Days in Patients with Severe Covid-19." N Engl J Med **383**(19): 1827-1837.

282. Goldstein, E. and M. Lipsitch (2020). "Temporal rise in the proportion of younger adults and older adolescents among coronavirus disease (COVID-19) cases following the introduction of physical distancing measures, Germany, March to April 2020." Eurosurveillance **25**(17).

283. Gollwitzer, A., et al. (2020). "Partisan differences in physical distancing are linked to health outcomes during the COVID-19 pandemic." Nature Human Behaviour **4**(11): 1186-1197.

284. Golubev, A. G. and A. V. Sidorenko (2020). "[Theory and practice of aging upon COVID-19 pandemic.]." Adv Gerontol **33**(2): 397-408.

285. Gómez-Salgado, J., et al. (2020). "Sense of coherence and psychological distress among healthcare workers during the COVID-19 pandemic in Spain." Sustainability (Switzerland) **12**(17).

286. Gosavi, A. and R. J. Marley (2020). "Public Policy in a Pandemic: A Hazard-Control Perspective and a Case Study of the BCG Vaccine for COVID-19." IEEE Engineering Management Review **48**(3): 111-117.

287. Gössling, S. (2020). "Risks, resilience, and pathways to sustainable aviation: A COVID-19 perspective." J Air Transp Manag **89**: 101933.

288. Gössling, S., et al. (2020). "Pandemics, tourism and global change: a rapid assessment of COVID-19." Journal of Sustainable Tourism: 1-20.

289. Gøtzsche, P. C. (2020). "The coronavirus pandemic: can we handle such epidemics better?" Journal of the Royal Society of Medicine **113**(5): 171-175.

290. Greco, A., et al. (2020). "Outcomes of renin-angiotensin-aldosterone system blockers in patients with COVID-19: A systematic review and meta-analysis." European Heart Journal - Cardiovascular Pharmacotherapy **6**(5): 335-337.

291. Green, M. S., et al. (2020). "The confounded crude case-fatality rates (CFR) for COVID-19 hide more than they reveal-a comparison of age-specific and age-adjusted CFRs between seven countries." PLoS One **15**(10 October).

292. Griffiths, F., et al. (2020). "Decision-making around admission to intensive care in the UK pre-COVID-19: a multicentre ethnographic study." Anaesthesia.

293. Grottesi, A., et al. (2020). "Computational studies of SARS-CoV-2 3clpro: Insights from md simulations." International Journal of Molecular Sciences **21**(15): 1-18.

294. Guallar, M. P., et al. (2020). "Inoculum at the time of SARS-CoV-2 exposure and risk of disease severity." Int J Infect Dis **97**: 290-292.

295. Guan, H., et al. (2020). "Promoting healthy movement behaviours among children during the COVID-19 pandemic." The Lancet Child and Adolescent Health **4**(6): 416-418.

296. Guckenberger, M., et al. (2020). "Practice Recommendations for Lung Cancer Radiotherapy During the COVID-19 Pandemic: An ESTRO-ASTRO Consensus Statement." Int J Radiat Oncol Biol Phys **107**(4): 631-640.

297. Guglielmi, S., et al. (2020). "Public acceptability of containment measures during the COVID-19 pandemic in Italy: how institutional confidence and specific political support matter." International Journal of Sociology and Social Policy **40**(9-10): 1069-1085.

298. Guijarro, C., et al. (2020). "Differential risk for COVID-19 in the first wave of the disease among Spaniards and migrants from different areas of the world living in Spain." Revista Clinica Espanola.

299. Günther, F., et al. (2020). "Nowcasting the COVID-19 pandemic in Bavaria." Biom J.

300. Gusenbauer, M. and N. R. Haddaway (2020). "What every Researcher should know about Searching - Clarified Concepts, Search Advice, and an Agenda to improve Finding in Academia." Res Synth Methods.

301. Gustavsson, J. and L. Beckman (2020). "Compliance to Recommendations and Mental Health Consequences among Elderly in Sweden during the Initial Phase of the COVID-19 Pandemic-A Cross Sectional Online Survey." Int J Environ Res Public Health **17**(15).

302. Guthrie, M. (2020). "SIRD Model of the Time Progression of Excess Deaths in NYC, the Total US, and Sweden Observed in the Spring of 2020." SSRN.

303. Guzmán Herrador, B. R., et al. (2020). "COVID-19 outbreaks in a transmission control scenario: Challenges posed by social and leisure activities, and for workers in vulnerable conditions, Spain, early summer 2020." Eurosurveillance **25**(35).

304. Guzzetta, G., et al. (2020). "Potential short-term outcome of an uncontrolled COVID-19 epidemic in Lombardy, Italy, February to March 2020." Eurosurveillance **25**(12).

305. Haake, C., et al. (2020). "Coronavirus Infections in Companion Animals: Virology, Epidemiology, Clinical and Pathologic Features." Viruses **12**(9).

306. Habib, H. (2020). "Has Sweden's controversial covid-19 strategy been successful?" The BMJ **369**.

307. Hägg, S., et al. (2020). "Age, Frailty, and Comorbidity as Prognostic Factors for Short-Term Outcomes in Patients With Coronavirus Disease 2019 in Geriatric Care." J Am Med Dir Assoc **21**(11): 1555-1559.e1552.

308. Hagman, K., et al. (2020). "SARS-CoV-2 RNA in serum as predictor of severe outcome in COVID-19: a retrospective cohort study." Clinical infectious diseases : an official publication of the Infectious Diseases Society of America.

309. Håkansson, A. (2020). "Changes in gambling behavior during the COVID-19 pandemic—A web survey study in Sweden." International Journal of Environmental Research and Public Health **17**(11): 1-16.

310. Halevy, N. (2020). "Strategic thinking and behavior during a pandemic." Judgment and Decision Making **15**(5): 648-659.

311. Hallberg, I. R. (2020). "Failure to protect the frailest old from Covid-19: Safety in the light of person-centered care." Nordic Journal of Nursing Research **40**(4): 171-175.

312. Halonen, J. I., et al. (2020). "The Helsinki Declaration 2020: Europe that protects." The Lancet Planetary Health **4**(11): e503-e505.

313. Hamlin, M., et al. (2020). "Attitudes of the public to receiving medical care during emergencies through remote physician– patient communications." International Journal of Environmental Research and Public Health **17**(14): 1-12.

314. Han, M., et al. (2020). "Utilizing microbiome approaches to assist source tracking, treatment and prevention of COVID-19: Review and assessment." Computational and Structural Biotechnology Journal **18**: 3615-3622.

315. Hanke, L., et al. (2020). "An alpaca nanobody neutralizes SARS-CoV-2 by blocking receptor interaction." Nature Communications **11**(1).

316. Hansson, E., et al. (2020). "Large differences in excess mortality in March-May 2020 by country of birth in Sweden." Lakartidningen **117**.

317. Haque, M., et al. (2020). "Availability and price changes of potential medicines and equipment for the prevention and treatment of covid-19 among pharmacy and drug stores in bangladesh; findings and implications." Bangladesh Journal of Medical Science **19**(Special issue): S36-S50.

318. Harries, A. D., et al. (2020). "Testing wastewater to detect severe acute respiratory syndrome coronavirus 2 in communities." Trans R Soc Trop Med Hyg **114**(10): 782-783.

319. Harvala, H., et al. (2020). "Convalescent plasma treatment for SARS-CoV-2 infection: Analysis of the first 436 donors in England, 22 April to 12 May 2020." Eurosurveillance **25**(28).

320. Hassan, M. M., et al. (2020). "Role of Environmental Temperature on the Attack rate and Case fatality rate of Coronavirus Disease 2019 (COVID-19) Pandemic." Infection Ecology and Epidemiology **10**(1).

321. Hedenstierna, G., et al. (2020). "Treatment of COVID-19 by inhaled NO to reduce shunt?" American Journal of Respiratory and Critical Care Medicine **202**(4): 618.

322. Hemkens, L. G., et al. (2020). "The worldwide clinical trial research response to the COVID-19 pandemic - the first 100 days." F1000Research **9**.

323. Herbert, G. (2020). "What Is Canada’s Average Cost of Policies Preventing a Death from COVID-19?" SSRN.

324. Herbst, K., et al. (2020). "Protocol: Leveraging a demographic and health surveillance system for Covid-19 Surveillance in rural KwaZulu-Natal." Wellcome Open Research **5**.

325. Hermans, A. N. L., et al. (2020). "On-demand mobile health infrastructures to allow comprehensive remote atrial fibrillation and risk factor management through teleconsultation." Clinical Cardiology **43**(11): 1232-1239.

326. Heymann, J., et al. (2020). "Protecting health during COVID-19 and beyond: A global examination of paid sick leave design in 193 countries*." Global Public Health **15**(7): 925-934.

327. Hidvégi, M. and M. Nichelatti (2020). "Bacillus calmette-guérin vaccination policy and consumption of ammonium chloride-enriched confectioneries may be factors in reducing COVID-19 death rates in Europe." Israel Medical Association Journal **22**(8): 435-438.

328. Hildenwall, H., et al. (2020). "Paediatric COVID-19 admissions in a region with open schools during the two first months of the pandemic." Acta Paediatrica, International Journal of Paediatrics **109**(10): 2152-2154.

329. Hodgson, L., et al. (2020). "Interassociation consensus recommendations for pitch-side emergency care and personal protective equipment for elite sport during the COVID-19 pandemic." Br J Sports Med.

330. Holmager, T. L., et al. (2020). "Geography of COVID-19 in Denmark." Scandinavian journal of public health: 1403494820975607.

331. Hong, H. G. and Y. Li (2020). "Estimation of time-varying reproduction numbers underlying epidemiological processes: A new statistical tool for the COVID-19 pandemic." PLoS One **15**(7 July).

332. Hooli, S. and C. King (2020). "Generalizability of Coronavirus Disease 2019 (COVID-19) Clinical Prediction Models." Clin Infect Dis **71**(15): 897.

333. Hornick, A., et al. (2020). "Anisocytosis is associated with short-term mortality in covid-19 and may reflect proinflammatory signature in uninfected ambulatory adults." Pathogens and Immunity **5**(1): 312-326.

334. Horton, R. (2020). "Offline: Science and politics in the era of COVID-19." The Lancet **396**(10259): 1319.

335. Hossain, M. S., et al. (2020). "Impact of weather on COVID-19 transmission in south Asian countries: An application of the ARIMAX model." Science of the Total Environment.

336. Hrusak, O., et al. (2020). "Flash survey on severe acute respiratory syndrome coronavirus-2 infections in paediatric patients on anticancer treatment." European Journal of Cancer **132**: 11-16.

337. Huang, H., et al. (2020). "Clinical characteristics of COVID-19 in patients with preexisting ILD: A retrospective study in a single center in Wuhan, China." Journal of Medical Virology **92**(11): 2742-2750.

338. Huet, T., et al. (2020). "Anakinra for severe forms of COVID-19: a cohort study." The Lancet Rheumatology **2**(7): e393-e400.

339. Hulsbergen, A. F. C., et al. (2020). "Ethical triage during the COVID-19 pandemic: a toolkit for neurosurgical resource allocation." Acta Neurochirurgica **162**(7): 1485-1490.

340. Hultström, M., et al. (2020). "Blood type A associates with critical COVID-19 and death in a Swedish cohort." Critical Care **24**(1).

341. Hungerford, D. and N. A. Cunliffe (2020). "Coronavirus disease (COVID-19) – impact on vaccine preventable diseases." Eurosurveillance **25**(18).

342. Hunt, K., et al. (2020). "Protocol for a mixed-method investigation of the impact of the covid-19 pandemic and gambling practices, experiences and marketing in the uk: The “betting and gaming covid-19 impact study”." International Journal of Environmental Research and Public Health **17**(22): 1-11.

343. Ilie, P. C., et al. (2020). "The role of vitamin D in the prevention of coronavirus disease 2019 infection and mortality." Aging Clinical and Experimental Research **32**(7): 1195-1198.

344. Im Kampe, E. O., et al. (2020). "Surveillance of COVID-19 school outbreaks, Germany, March to August 2020." Eurosurveillance **25**(38).

345. Imami, A. S., et al. (2020). "Oxytocin’s anti-inflammatory and proimmune functions in covid-19: A transcriptomic signature-based approach." Physiological Genomics **52**(9): 401-407.

346. Ioannidis, J. P. A., et al. (2020). "Population-level COVID-19 mortality risk for non-elderly individuals overall and for non-elderly individuals without underlying diseases in pandemic epicenters." Environmental Research **188**.

347. Iravani, B., et al. (2020). "Erratum: Relationship between odor intensity estimates and COVID-19 prevalence prediction in a Swedish population (Chem. Senses (2020) DOI: 10.1093/chemse/bjaa034)." Chemical Senses **45**(6): 491-492.

348. Iravani, B., et al. (2020). "Relationship between odor intensity estimates and COVID-19 prevalence prediction in a Swedish population." Chemical Senses **45**(6): 449-456.

349. Irwin, R. E. (2020). "Misleading media coverage of Sweden's response to covid-19." The BMJ **370**.

350. Ismail, L., et al. (2020). "Tailoring time series models for forecasting coronavirus spread: Case studies of 187 countries." Computational and Structural Biotechnology Journal **18**: 2972-3206.

351. Ivana, et al. (2020). The socio-economic impact on policy national food security: Study of the effects of agricultural product distribution by poor farmers on the pandemic covid19. Proceedings of the International Conference on Industrial Engineering and Operations Management.

352. Iyanda, A. E., et al. (2020). "A retrospective cross-national examination of COVID-19 outbreak in 175 countries: a multiscale geographically weighted regression analysis (January 11-June 28, 2020)." Journal of Infection and Public Health **13**(10): 1438-1445.

353. Jafferali, M. H., et al. (2020). "Benchmarking virus concentration methods for quantification of SARS-CoV-2 in raw wastewater." Science of the Total Environment.

354. Jamrozik, E. and G. S. Heriot (2020). "Pandemic public health policy: with great power comes great responsibility." Internal Medicine Journal **50**(10): 1169-1173.

355. Jandrić, P., et al. (2020). "Philosophy of education in a new key: Who remembers Greta Thunberg? Education and environment after the coronavirus." Educational Philosophy and Theory.

356. Jefferson, T., et al. (2020). "Physical interventions to interrupt or reduce the spread of respiratory viruses." Cochrane Database of Systematic Reviews(11).

357. Jemberie, W. B., et al. (2020). "Substance Use Disorders and COVID-19: Multi-Faceted Problems Which Require Multi-Pronged Solutions." Frontiers in Psychiatry **11**.

358. Jesenak, M., et al. (2020). "COVID-19, chronic inflammatory respiratory diseases and eosinophils—Observations from reported clinical case series." Allergy: European Journal of Allergy and Clinical Immunology **75**(7): 1819-1822.

359. Jia, J. S., et al. (2020). "Population flow drives spatio-temporal distribution of COVID-19 in China." Nature **582**(7812): 389-394.

360. Jiguet, F. (2020). "The Fox and the Crow. A need to update pest control strategies." Biological Conservation **248**.

361. Jit, M., et al. (2020). "Estimating number of cases and spread of coronavirus disease (COVID-19) using critical care admissions, United Kingdom, February to March 2020." Eurosurveillance **25**(18): 1-5.

362. Joelle, G. (2020). "States of Emergency." SSRN.

363. Johansson, F., et al. (2020). "Depression, Anxiety and Stress Symptomatology among Swedish University Students Before and During the COVID-19 Pandemic: A Cohort Study." ResearchSquare.

364. Johnson, H. C., et al. (2020). "Potential scenarios for the progression of a COVID-19 epidemic in the European Union and the European Economic Area, March 2020." Eurosurveillance **25**(9).

365. Jones, G. W., et al. (2020). "No small matter: A perspective on nanotechnology-enabled solutions to fight COVID-19." Nanomedicine **15**(24): 2411-2427.

366. Jonmarker, S., et al. (2020). "Dosing of thromboprophylaxis and mortality in critically ill COVID-19 patients." Crit Care **24**(1): 653.

367. Jung, F., et al. (2020). "Herd immunity or suppression strategy to combat COVID-19." Clin Hemorheol Microcirc **75**(1): 13-17.

368. Jung, J., et al. (2020). "The Effects of Pre-Response Before COVID-19 Outbreak on Strategic Decision Making." ResearchSquare.

369. Jung, S. Y., et al. (2020). "Real-World Implications of a Rapidly Responsive COVID-19 Spread Model with Time-Dependent Parameters via Deep Learning: Model Development and Validation." J Med Internet Res **22**(9): e19907.

370. Jurgiel, J., et al. (2020). "Do pets protect their owners in the COVID-19 era?" Medical Hypotheses **142**.

371. Juul, S., et al. (2020). "Interventions for treatment of COVID-19: A living systematic review with meta-analyses and trial sequential analyses (The LIVING Project)." PLoS Medicine **17**(9).

372. Jylhävä, et al. (2020). "Age, frailty and comorbidity as prognostic factors for short-term outcomes in patients with COVID-19 in geriatric care." Journal of the American Medical Directors Association.

373. Kaden, R. (2020). "Early Phylogenetic Diversification of SARS-CoV-2: Determination of Variants and the Effect on Epidemiology, Immunology, and Diagnostics." J Clin Med **9**(6).

374. Kadkhoda, K. (2020). "Letter to the editor: COVID-19: how accurate are seroprevalence studies?" Eurosurveillance **25**(30): 1-2.

375. Kafrelsheikh, U. (2020). "Recombinant Bacterial ACE2 Receptors -Like Enzyme of B38-CAP Could be Promising COVID-19 Infection- and Lung Injury Preventing Drug Better Than Recombinant Human ACE2." clinicaltrials.gov.

376. Kalin, N. H. (2020). "Insights into suicide and depression." American Journal of Psychiatry **177**(10): 877-880.

377. Kamarajah, S. K., et al. (2020). "The influence of the SARS-CoV-2 pandemic on esophagogastric cancer services: An international survey of esophagogastric surgeons." Diseases of the Esophagus **33**(7).

378. Kamerlin, S. C. L. and P. M. Kasson (2020). "Managing COVID-19 spread with voluntary public-health measures: Sweden as a case study for pandemic control." Clin Infect Dis.

379. Kammerer, N. B. and W. Stummer (2020). "Some dissimilarity measures of branching processes and optimal decision making in the presence of potential pandemics." Entropy **22**(8).

380. Kanberg, N., et al. (2020). "Neurochemical evidence of astrocytic and neuronal injury commonly found in COVID-19." Neurology **95**(12): e1754-e1759.

381. Kander, T. (2020). "Coagulation disorder in COVID-19." The Lancet Haematology **7**(9): e630-e632.

382. Kantová, M. and M. Arltová (2020). "Emerging from crisis: Sweden’s active labour market policy and vulnerable groups." Economic and Labour Relations Review **31**(4): 543-564.

383. Kao, K., et al. (2021). "The ABCs of Covid-19 prevention in Malawi: Authority, benefits, and costs of compliance." World Development **137**.

384. Kapoor, K. M., et al. (2020). "COVID-19 Pandemic: Consensus guidelines for preferred practices in an aesthetic clinic." Dermatol Ther **33**(4): e13597.

385. Kara, M., et al. (2020). "'Scientific Strabismus' or two related pandemics: Coronavirus disease and vitamin D deficiency." British Journal of Nutrition **124**(7): 736-741.

386. Karadağ, E. (2020). "Increase in Covid-19 Cases and Case Fatality and Case Recovery Rates in Europe: A Cross Temporal Meta-Analysis." Journal of medical virology **92**(9): 1511-1517.

387. Karlsson, J. O. G., et al. (2020). "May Mangafodipir or Other SOD Mimetics Contribute to Better Care in COVID-19 Patients?" Antioxidants (Basel) **9**(10).

388. Karlsson, U. and C. J. Fraenkel (2020). "Covid-19: Risks to healthcare workers and their families." The BMJ **371**.

389. Kashour, T., et al. (2020). "Angiotensin Converting Enzyme Inhibitors and Angiotensin Receptor Blockers and Mortality Among COVID-19 Patients: A Systematic Review and Meta-Analysis." American journal of therapeutics.

390. Kates, O. S., et al. (2020). "COVID-19 in solid organ transplant: A multi-center cohort study." Clinical infectious diseases : an official publication of the Infectious Diseases Society of America.

391. Kavaliunas, A., et al. (2020). "Swedish policy analysis for Covid-19." Health Policy Technol **9**(4): 598-612.

392. Kawala, B. A., et al. (2020). "Effect of covid-19 response in uganda on street children." Pan African Medical Journal **35**(2): 1-2.

393. Kc, A., et al. (2020). "Effect of the COVID-19 pandemic response on intrapartum care, stillbirth, and neonatal mortality outcomes in Nepal: a prospective observational study." The Lancet Global Health **8**(10): e1273-e1281.

394. Kerneis, M., et al. (2020). "Severe acute respiratory syndrome coronavirus 2 and renin-angiotensin system blockers: A review and pooled analysis." Archives of Cardiovascular Diseases.

395. Keszei, Z., et al. (2020). "100 days of solitude: The spring of COVID-19 through the eyes of 15 young virologists of the INITIATE program." Virus Research **287**.

396. Khafaie, M. A. and F. Rahim (2020). "Cross-country comparison of case fatality rates of Covid-19/SARS-CoV-2." Osong Public Health and Research Perspectives **11**(2): 74-80.

397. Khak, M., et al. (2020). "Descriptive epidemiology of traumatic injuries during the first lockdown period of COVID-19 crisis in iran: A multicenter study." Asian Journal of Sports Medicine **11**(2): 1-5.

398. Khalaj, K., et al. (2020). "Systematic review of extracellular vesicle-based treatments for lung injury: are EVs a potential therapy for COVID-19?" Journal of Extracellular Vesicles **9**(1).

399. Khalifa, S. A. M., et al. (2020). "Comprehensive overview on multiple strategies fighting covid-19." International Journal of Environmental Research and Public Health **17**(16): 1-13.

400. Khalife, J. (2020). "Thinking strategically for COVID-19: Suppress and lift, to flatten or to crush?" Eastern Mediterranean Health Journal **26**(8): 877-878.

401. Khan, A. A., et al. (2020). "Survival and estimation of direct medical costs of hospitalized covid-19 patients in the kingdom of saudi arabia (Short title: Covid-19 survival and cost in saudi arabia)." International Journal of Environmental Research and Public Health **17**(20): 1-13.

402. Khan, N. S. and M. A. Chishti (2020). "Security challenges in fog and iot, blockchain technology and cell tree solutions: A review." Scalable Computing **21**(3): 515-541.

403. Khan, Y. A., et al. (2020). "Machine learning-based mortality rate prediction using optimized hyper-parameter." Comput Methods Programs Biomed **197**: 105704.

404. Khorram-Manesh, A., et al. (2020). ""Does the prosperity of a country play a role in COVID-19 outcomes?"." Disaster medicine and public health preparedness: 1-20.

405. Khorram-Manesh, A., et al. (2020). "The Development of Swedish Military Healthcare System: Part II-Re-evaluating the Military and Civilian Healthcare Systems in Crises Through a Dialogue and Study Among Practitioners." Mil Med.

406. Kinross, P., et al. (2020). "Rapidly increasing cumulative incidence of coronavirus disease (COVID-19) in the European Union/European Economic Area and the United Kingdom, 1 January to 15 March 2020." Eurosurveillance **25**(11).

407. Kite, T., et al. (2020). "TCT CONNECT-215 Demographics and In-Hospital Outcomes of COVID-19 Patients Undergoing an Invasive Strategy for Acute Coronary Syndrome: The Global Multi-Centre Prospective COVID-ACS Registry." Journal of the American College of Cardiology **76**(17): B91.

408. Kjellberg, A., et al. (2020). "Can hyperbaric oxygen safely serve as an anti-inflammatory treatment for COVID-19?" Medical Hypotheses **144**.

409. Kofler, W., et al. (2020). "Is fighting against COVID-19 enough?" Scandinavian journal of public health: 1403494820969539.

410. Korhale, N., et al. (2020). "Disparity in ozone trends under COVID-19 lockdown in a closely located coastal and hillocky metropolis of India." Air Quality, Atmosphere and Health.

411. Kosiborod, M., et al. (2020). "Effects of Dapagliflozin on Prevention of Major Clinical Events and Recovery in Patients with Respiratory Failure due to COVID-19: The Design and Rationale for the DARE-19 study." Diabetes, obesity & metabolism.

412. Kovriguine, D. A. and S. P. Nikitenkova (2020). "Predictive monitoring of secondary epidemic waves of COVID-19 in Iran, Russia and other countries." Bulletin of Russian State Medical University(4): 27-32.

413. Kowalik, M. M., et al. (2020). "COVID-19 — Toward a comprehensive understanding of the disease." Cardiology Journal **27**(2): 99-114.

414. Kowalski, L. P., et al. (2020). "COVID-19 pandemic: Effects and evidence-based recommendations for otolaryngology and head and neck surgery practice." Head and Neck.

415. Krauss, E. S., et al. (2020). "Lessons Learned: Using the Caprini Risk Assessment Model to Provide Safe and Efficacious Thromboprophylaxis Following Hip and Knee Arthroplasty." Clinical and Applied Thrombosis/Hemostasis **26**.

416. Kremer, C., et al. (2020). "Authors' response: Estimating the generation interval for COVID-19 based on symptom onset data." Eurosurveillance **25**(29): 18-19.

417. Krysl, D., et al. (2020). "The COVID-19 outbreak and approaches to performing EEG in Europe." Epileptic Disorders **22**(5): 548-554.

418. Kuc-Czarnecka, M. (2020). "COVID-19 and digital deprivation in Poland." Oeconomia Copernicana **11**(3): 415-431.

419. Kucirka, L. M., et al. (2020). "Severity of COVID-19 in pregnancy: A review of current evidence." American Journal of Reproductive Immunology **84**(5).

420. Kuhn, U., et al. (2020). "Who is most affected by the Corona crisis? An analysis of changes in stress and well-being in Switzerland." European Societies.

421. Kumar, M., et al. (2020). "A chronicle of SARS-CoV-2: Seasonality, environmental fate, transport, inactivation, and antiviral drug resistance." Journal of Hazardous Materials.

422. Kumar, M., et al. (2021). "Decay of SARS-CoV-2 RNA along the wastewater treatment outfitted with Upflow Anaerobic Sludge Blanket (UASB) system evaluated through two sample concentration techniques." Sci Total Environ **754**: 142329.

423. Kurdi, A., et al. (2020). "A systematic review and meta-analysis of the use of renin-angiotensin system drugs and COVID-19 clinical outcomes: What is the evidence so far?" Pharmacology Research and Perspectives **8**(6).

424. Kwaan, H. C. (2020). "Coronavirus Disease 2019: The Role of the Fibrinolytic System from Transmission to Organ Injury and Sequelae." Seminars in Thrombosis and Hemostasis **46**(7): 841-844.

425. Kwok, K. O., et al. (2020). "Epidemiological characteristics of the first 53 laboratory-confirmed cases of COVID-19 epidemic in Hong Kong, 13 February 2020." Euro surveillance : bulletin Europeen sur les maladies transmissibles = European communicable disease bulletin **25**(16).

426. Laato, S., et al. (2020). "Unusual purchasing behavior during the early stages of the COVID-19 pandemic: The stimulus-organism-response approach." Journal of Retailing and Consumer Services **57**.

427. LaCourse, S. M., et al. (2020). "Low prevalence of SARS-CoV-2 among pregnant and postpartum patients with universal screening in Seattle, Washington." Clinical infectious diseases : an official publication of the Infectious Diseases Society of America.

428. LaCourse, S., et al. (2020). "Importance of inclusion of pregnant and breastfeeding women in covid-19 therapeutic trials." Clinical Infectious Diseases **71**(15): 879-881.

429. Lagi, F., et al. (2020). "Early experience of an infectious and tropical diseases unit during the coronavirus disease (COVID-19) pandemic, Florence, Italy, February to March 2020." Eurosurveillance **25**(17).

430. Laird, E., et al. (2020). "Vitamin D and inflammation: Potential implications for severity of Covid-19." Irish Medical Journal **113**(5).

431. Lan, F. Y., et al. (2020). "Work-related COVID-19 transmission in six Asian countries/areas: A follow-up study." PLoS One **15**(5).

432. Lane, J. C. E., et al. (2020). "Risk of depression, suicide and psychosis with hydroxychloroquine treatment for rheumatoid arthritis: a multinational network cohort study." Rheumatology (Oxford).

433. Lane, J. C. E., et al. (2020). "Risk of hydroxychloroquine alone and in combination with azithromycin in the treatment of rheumatoid arthritis: a multinational, retrospective study." Lancet Rheumatol **2**(11): e698-e711.

434. Larfors, G., et al. (2020). "Covid-19 intensive care admissions and mortality among swedish patients with cancer." Acta Oncologica.

435. Larsson, E., et al. (2020). "Characteristics and outcomes of patients with COVID-19 admitted to ICU in a tertiary hospital in Stockholm, Sweden." Acta Anaesthesiologica Scandinavica.

436. Last, M. (2020). "The first wave of COVID-19 in Israel—Initial analysis of publicly available data." PLoS One **15**(10 October).

437. Le, H. T., et al. (2020). "Feasibility of Intersectoral Collaboration in Epidemic Preparedness and Response at Grassroots Levels in the Threat of COVID-19 Pandemic in Vietnam." Frontiers in public health **8**.

438. Leal-Neto, O. B., et al. (2020). "Prioritizing COVID-19 tests based on participatory surveillance and spatial scanning." International Journal of Medical Informatics **143**.

439. Lebopo, C. M., et al. (2020). Explaining factors affecting telework adoption in South African organisations pre-COVID-19. ACM International Conference Proceeding Series.

440. Leclercq-Vandelannoitte, A. and J. Aroles (2020). "Does the end justify the means?Information systems and control society in the age of pandemics." European Journal of Information Systems.

441. Lee, K. B., et al. (2020). "COVID-19, flattening the curve, and Benford's law." Physica A: Statistical Mechanics and its Applications **559**.

442. Lee, K. H., et al. (2020). "Efficacy of Corticosteroids in Patients with SARS, MERS and COVID-19: A Systematic Review and Meta-Analysis." J Clin Med **9**(8).

443. Lenzo, V., et al. (2020). "Resilience Contributes to Low Emotional Impact of the COVID-19 Outbreak Among the General Population in Italy." Frontiers in Psychology **11**.

444. Leonardi, M., et al. (2020). "Avoiding the Banality of Evil in Times of COVID-19: Thinking Differently with a Biopsychosocial Perspective for Future Health and Social Policies Development." SN Compr Clin Med: 1-3.

445. Lerm, M. (2020). "On the relationship between BCG coverage and national COVID-19 outcome: could ‘heterologous’ herd immunity explain why some countries are better off?" Journal of Internal Medicine **288**(6): 682-688.

446. Lexchin, J. (2020). "COVID-19: still much to learn." Journal of the Royal Society of Medicine **113**(7): 244.

447. Li, B., et al. (2020). "Epidemiological and Clinical Characteristics of COVID-19 in Children: A Systematic Review and Meta-Analysis." Front Pediatr **8**: 591132.

448. Li, C., et al. (2020). "Retrospective analysis of the possibility of predicting the COVID-19 outbreak from Internet searches and social media data, China, 2020." Eurosurveillance **25**(10).

449. Li, Q., et al. (2020). "Prevalence and factors for anxiety during the coronavirus disease 2019 (COVID-19) epidemic among the teachers in China." Journal of Affective Disorders **277**: 153-158.

450. Liang, H. and G. Acharya (2020). "Novel corona virus disease (COVID-19) in pregnancy: What clinical recommendations to follow?" Acta Obstetricia et Gynecologica Scandinavica **99**(4): 439-442.

451. Lidskog, R. and A. Standring (2020). "The institutional machinery of expertise: Producing facts, figures and futures in COVID-19." Acta Sociologica (United Kingdom) **63**(4): 443-446.

452. Lidskog, R., et al. (2020). "COVID-19, the climate, and transformative change: Comparing the social anatomies of crises and their regulatory responses." Sustainability (Switzerland) **12**(16).

453. Lidström, A. K., et al. (2020). "Work at inpatient care units is associated with an increased risk of SARS-CoV-2 infection; a cross-sectional study of 8679 healthcare workers in Sweden." Ups J Med Sci **125**(4): 305-310.

454. Lin, C. Y., et al. (2020). "Investigating mediated effects of fear of COVID-19 and COVID-19 misunderstanding in the association between problematic social media use, psychological distress, and insomnia." Internet Interventions **21**.

455. Lin, C. Y., et al. (2020). "Using an integrated social cognition model to predict COVID-19 preventive behaviours." Br J Health Psychol **25**(4): 981-1005.

456. Lin, P. I., et al. (2020). "Methodology in the GBD study of China." The Lancet **396**(10243): 25.

457. Lin, S., et al. (2020). "Region-specific air pollutants and meteorological parameters influence COVID-19: A study from mainland China." Ecotoxicology and Environmental Safety **204**.

458. Lindahl, J. F., et al. (2020). "High seroprevalence of SARS-CoV-2 in elderly care employees in Sweden." Infect Ecol Epidemiol **10**(1): 1789036.

459. Lindner, P., et al. (2020). "Transitioning Between Online Gambling Modalities and Decrease in Total Gambling Activity, but No Indication of Increase in Problematic Online Gambling Intensity During the First Phase of the COVID-19 Outbreak in Sweden: A Time Series Forecast Study." Front Public Health **8**: 554542.

460. Lindström, M. (2020). "A commentary on “The trouble with trust: Time-series analysis of social capital, income inequality, and COVID-19 deaths in 84 countries”." Social Science and Medicine **263**.

461. Lindstrom, M. (2020). "The COVID-19 pandemic and the Swedish strategy: Epidemiology and postmodernism." SSM Popul. Health **11**: 100643.

462. Ling, J., et al. (2020). "Spatio-temporal mutational profile appearances of Swedish SARS-CoV-2 during the early pandemic." Viruses **12**(9).

463. Liu, A., et al. (2020). "PDB32 COST-EFFECTIVENESS OF ONCE-WEEKLY SEMAGLUTIDE 1 MG VS. CANAGLIFLOZIN 300 MG IN PATIENTS WITH TYPE 2 DIABETES IN A CANADIAN SETTING." Value in Health **23**: S114.

464. Liu, P., et al. (2020). "Combination treatments with hydroxychloroquine and azithromycin are compatible with the therapeutic induction of anticancer immune responses." OncoImmunology **9**(1).

465. Liu, Y., et al. (2020). "Clinical and biochemical indexes from 2019-nCoV infected patients linked to viral loads and lung injury." Science China Life Sciences **63**(3): 364-374.

466. Lloyd, A. J., et al. (2020). "PRO32 PREFERENCE WEIGHTS FOR QUALITY-ADJUSTED LIFE-YEARS ESTIMATION FOR TREATMENTS OF PAROXYSMAL NOCTURNAL HEMOGLOBINURIA IN FIVE COUNTRIES." Value in Health **23**: S334.

467. Locht, C. and M. Lerm (2020). "Good old BCG – what a century-old vaccine can contribute to modern medicine." Journal of Internal Medicine **288**(6): 611-613.

468. Löfvendahl, S., et al. (2020). "PIN20 SOCIETAL PRODUCTIVITY GAINS FROM NEW THERAPIES IN HEPATITIS C." Value in Health **23**: S172.

469. Lohiniva, A. L., et al. (2020). "Understanding coronavirus disease (COVID-19) risk perceptions among the public to enhance risk communication efforts: A practical approach for outbreaks, Finland, February 2020." Eurosurveillance **25**(13).

470. Lokken, E. M., et al. (2020). "Clinical characteristics of 46 pregnant women with a severe acute respiratory syndrome coronavirus 2 infection in Washington State." American Journal of Obstetrics and Gynecology **223**(6): 911.e911-911.e914.

471. Loomba, R. S., et al. (2021). "Disparities in case frequency and mortality of coronavirus disease 2019 (COVID-19) among various states in the United States." Annals of Medicine **53**(1): 151-159.

472. Lucero, A. D., et al. (2020). "Underutilization of the emergency department during the covid-19 pandemic." Western Journal of Emergency Medicine **21**(6).

473. Ludvigsson, J. (2020). "Misleading obsession to number of deaths with Covid-19." Lakartidningen **117**.

474. Ludvigsson, J. F. (2020). "Systematic review of COVID-19 in children shows milder cases and a better prognosis than adults." Acta Paediatrica, International Journal of Paediatrics **109**(6): 1088-1095.

475. Luigi, C. and O. Romina (2020). "D936Y and Other Mutations in the Fusion Core of the SARS-Cov-2 Spike Protein Heptad Repeat 1 Undermine the Post-Fusion Assembly." bioRxiv.

476. Lumley, S. F., et al. (2020). "SARS-CoV-2 antibody prevalence, titres and neutralising activity in an antenatal cohort, United Kingdom, 14 April to 15 June 2020." Eurosurveillance **25**(42).

477. Lundkvist, Å., et al. (2020). "Pronounced difference in Covid-19 antibody prevalence indicates cluster transmission in Stockholm, Sweden." Infection Ecology and Epidemiology **10**(1).

478. Luo, X., et al. (2020). "Prognostic Value of C-Reactive Protein in Patients with Coronavirus 2019." Clinical Infectious Diseases **71**(16): 2174-2179.

479. Luther, T., et al. (2020). "COVID-19 patients in intensive care develop predominantly oliguric acute kidney injury." Acta Anaesthesiologica Scandinavica.

480. Lv, H., et al. (2020). "Epidemiologic characteristics of traumatic fractures during the outbreak of coronavirus disease 2019 (COVID-19) in China: A retrospective & comparative multi-center study." Injury **51**(8): 1698-1704.

481. Lv, J., et al. (2020). "How can E-commerce businesses implement discount strategies through social media?" Sustainability (Switzerland) **12**(18).

482. Lv, M., et al. (2020). "Coronavirus disease (COVID-19): A scoping review." Eurosurveillance **25**(15).

483. Lyons, N. and G. Lăzăroiu (2020). "Addressing the covid-19 crisis by harnessing internet of things sensors and machine learning algorithms in data-driven smart sustainable cities." Geopolitics, History, and International Relations **12**(2): 65-71.

484. Lytras, T. and S. Tsiodras (2020). "Lockdowns and the COVID-19 pandemic: What is the endgame?" Scandinavian journal of public health: 1403494820961293.

485. Ma, L. L., et al. (2020). "Coronavirus Disease 2019 Related Clinical Studies: A Cross-Sectional Analysis." Frontiers in Pharmacology **11**.

486. Madjunkov, M., et al. (2020). "A comprehensive review of the impact of COVID-19 on human reproductive biology, assisted reproduction care and pregnancy: a Canadian perspective." Journal of Ovarian Research **13**(1).

487. Madsen, L. W., et al. (2021). "Low mortality of hospitalised patients with COVID-19 in a tertiary Danish hospital setting." International Journal of Infectious Diseases **102**: 212-219.

488. Mahmoud, A. B., et al. (2020). "A motivational standpoint of job insecurity effects on organizational citizenship behaviors: A generational study." Scandinavian Journal of Psychology.

489. Malik, Y. S., et al. (2020). "Coronavirus Disease Pandemic (COVID-19): Challenges and a Global Perspective." Pathogens **9**(7).

490. Malik, Y. S., et al. (2020). "Emerging coronavirus disease (COVID-19), a pandemic public health emergency with animal linkages: Current status update." Indian Journal of Animal Sciences **90**(3): 158-173.

491. Malik, Y. S., et al. (2020). "How artificial intelligence may help the Covid-19 pandemic: Pitfalls and lessons for the future." Reviews in Medical Virology.

492. Malmberg, H. and T. Britton (2020). "Inflow restrictions can prevent epidemics when contact tracing efforts are effective but have limited capacity: Inflow restrictions can prevent epidemics when contact tracing efforts are effective but have limited capacity." Journal of the Royal Society Interface **17**(170).

493. Mamun, M. A., et al. (2021). "The COVID-19 pandemic and serious psychological consequences in Bangladesh: A population-based nationwide study." J Affect Disord **279**: 462-472.

494. Mancia, G., et al. (2020). "Renin–angiotensin–aldosterone system blockers and the risk of COVID-19." New England Journal of Medicine **382**(25): 2431-2440.

495. Maqbool, A. and N. Z. Khan (2020). "Analyzing barriers for implementation of public health and social measures to prevent the transmission of COVID-19 disease using DEMATEL method." Diabetes and Metabolic Syndrome: Clinical Research and Reviews **14**(5): 887-892.

496. Marengoni, A., et al. (2020). "Beyond chronological age: Frailty and multimorbidity predict in-hospital mortality in patients with coronavirus disease 2019." J Gerontol A Biol Sci Med Sci.

497. Marengoni, A., et al. (2020). "The impact of delirium on outcomes for older adults hospitalised with COVID-19." Age Ageing **49**(6): 923-926.

498. Margraf, J., et al. (2020). "Behavioral measures to fight COVID-19: An 8-country study of perceived usefulness, adherence and their predictors." PLoS One **15**(12): e0243523.

499. Marinov, T. and R. Marinova (2020). "Inverse Problem for Identification of Infectivity and Recovery Rates in SIR Epidemic Models as Functions of Time Illustrated  with Corona Virus Dynamics  up to July 09, 2020." ResearchSquare.

500. Mark, K., et al. (2020). "Coronavirus disease (COVID-19) community testing team in Scotland: A 14-day review, 6 to 20 February 2020." Eurosurveillance **25**(12).

501. Marquioni, V. M. and M. A. M. de Aguiar (2020). "Quantifying the effects of quarantine using an IBM SEIR model on scalefree networks." Chaos, Solitons and Fractals **138**.

502. Martin, J. C., et al. (2020). "Lockdowns and COVID-19 Deaths in Scandinavia." SSRN.

503. Massad, E., et al. (2020). "Two complementary model-based methods for calculating the risk of international spreading of a novel virus from the outbreak epicentre. The case of COVID-19." Epidemiol Infect **148**: e109.

504. Matheeussen, V., et al. (2020). "International external quality assessment for SARSCoV-2 molecular detection and survey on clinical laboratory preparedness during the COVID-19 pandemic." Eurosurveillance **25**(27).

505. Mathiesen, T., et al. (2020). "A snapshot of European neurosurgery December 2019 vs. March 2020: just before and during the Covid-19 pandemic." Acta Neurochirurgica **162**(9): 2221-2233.

506. Mato, A. R., et al. (2020). "Outcomes of COVID-19 in patients with CLL: a multicenter international experience." Blood **136**(10): 1134-1143.

507. Matrajt, L. and T. Leung (2020). "Evaluating the effectiveness of social distancing interventions to delay or flatten the epidemic curve of Coronavirus disease." Emerging Infectious Diseases **26**(8): 1740-1748.

508. Mauri, D., et al. (2020). "Behind the numbers and the panic of a viral pandemic: Fixed restrictive oncology guidance may jeopardize patients’ survival." Journal of B.U.ON. **25**(3): 1277-1280.

509. Mauvais-Jarvis, F., et al. (2020). "Sex and gender: modifiers of health, disease, and medicine." Lancet **396**(10250): 565-582.

510. Mavian, C., et al. (2020). "Sampling bias and incorrect rooting make phylogenetic network tracing of SARS-COV-2 infections unreliable." Proceedings of the National Academy of Sciences of the United States of America **117**(23): 12522-12523.

511. Mayer, J. D. and N. D. Lewis (2020). "An inevitable pandemic: geographic insights into the COVID-19 global health emergency." Eurasian Geography and Economics **61**(4-5): 404-422.

512. McCarthy, J. J., et al. (2020). "Changing Outdated Methadone Regulations That Harm Pregnant Patients." Journal of addiction medicine.

513. McCartney, S. A., et al. (2020). "Obesity as a contributor to immunopathology in pregnant and non-pregnant adults with COVID-19." American Journal of Reproductive Immunology **84**(5).

514. McCauley, J. L. (2020). "Pandemic infection rates are deterministic but cannot be modeled." AIP Adv **10**(11): 115023.

515. McCracken, L. M., et al. (2020). "Psychological impact of COVID-19 in the Swedish population: Depression, anxiety, and insomnia and their associations to risk and vulnerability factors." European psychiatry : the journal of the Association of European Psychiatrists **63**(1): e81.

516. McGinlay, J., et al. (2020). "The impact of COVID-19 on the management of European protected areas and policy implications." Forests **11**(11): 1-15.

517. McKimm, J., et al. (2020). "Education for sustainable healthcare: Leadership to get from here to there." Med Teach **42**(10): 1123-1127.

518. Medina-Enríquez, M. M., et al. (2020). "ACE2: the molecular doorway to SARS-CoV-2." Cell and Bioscience **10**(1).

519. Mei, J., et al. (2020). "Development and external validation of a COVID-19 mortality risk prediction algorithm: A multicentre retrospective cohort study." BMJ Open **10**(12).

520. Melidou, A., et al. (2020). "Virological surveillance of influenza viruses in the WHO European Region in 2019/20 - impact of the COVID-19 pandemic." Euro Surveill **25**(46).

521. Menezes, M. O., et al. (2020). "Risk factors for adverse outcomes among pregnant and postpartum women with acute respiratory distress syndrome due to COVID-19 in Brazil." International Journal of Gynecology and Obstetrics **151**(3): 415-423.

522. Meo, S. A., et al. (2020). "Climate and COVID-19 pandemic: Effect of heat and humidity on the incidence and mortality in world's top ten hottest and top ten coldest countries." European Review for Medical and Pharmacological Sciences **24**(15): 8232-8238.

523. Michelozzi, P., et al. (2020). "Mortality impacts of the coronavirus disease (COVID-19) outbreak by sex and age: Rapid mortality surveillance system, Italy, 1 February to 18 April 2020." Eurosurveillance **25**(19).

524. Michielsen, K., et al. (2020). "International Sexual Health And REproductive health (I-SHARE) survey during COVID-19: study protocol for online national surveys and global comparative analyses." Sex Transm Infect.

525. Miles, D. K., et al. (2020). "“Stay at Home, Protect the National Health Service, Save Lives”: A cost benefit analysis of the lockdown in the United Kingdom." International Journal of Clinical Practice.

526. Miller, S., et al. (2020). "Suspension of Hip Surveillance for Children with Cerebral Palsy During the COVID-19 Outbreak: The Benefit of Hip Surveillance Does Not Outweigh the Risk of Infection." Indian Journal of Orthopaedics.

527. Misztal-Okońska, P., et al. (2020). "How Medical Studies in Poland Prepare Future Healthcare Managers for Crises and Disasters: Results of a Pilot Study." Healthcare (Basel) **8**(3).

528. Mizumoto, K., et al. (2020). "Estimating the asymptomatic proportion of coronavirus disease 2019 (COVID-19) cases on board the Diamond Princess cruise ship, Yokohama, Japan, 2020." Eurosurveillance **25**(10).

529. Moaath Mustafa, A., et al. (2020). "The Impact of Lockdown in England on Daily Confirmed Cases of COVID-19 and Related Deaths Compared to Sweden: A Comparative Interrupted Time Series Analysis." SSRN.

530. Modig, K., et al. (2020). "Covid-19 - deaths and analysis." Lakartidningen **117**.

531. Modig, K., et al. (2020). "EXCESS MORTALITY FROM COVID-19. WEEKLY EXCESS DEATH RATES BY AGE AND SEX FOR SWEDEN AND ITS MOST AFFECTED REGION." Eur J Public Health.

532. Mohamed, M. S., et al. (2020). "Sex differences in COVID-19: the role of androgens in disease severity and progression." Endocrine: 1-6.

533. Mohammad, M. A., et al. (2020). "Incidence and outcome of myocardial infarction treated with percutaneous coronary intervention during COVID-19 pandemic." Heart **106**(23): 1812-1818.

534. Mohammad, M. A., et al. (2020). "The association of mode of location activity and mobility with acute coronary syndrome: nationwide ecological study." J Intern Med.

535. Mohammadian, H. D., et al. (2020). Digital Transformation in Academic Society and Innovative Ecosystems in the World beyond Covid19-Pandemic with Using 7PS Model for IoT. Proceedings of 2020 IEEE Learning With MOOCS, LWMOOCS 2020.

536. Mohammadian, H. D., et al. (2020). The 5thWave and i-Sustainability plus Theories as Solutions for SocioEdu Consequences of Covid-19. Proceedings of 2020 IEEE Learning With MOOCS, LWMOOCS 2020.

537. Mohammed, A., et al. (2020). "Δ9-Tetrahydrocannabinol Prevents Mortality from Acute Respiratory Distress Syndrome through the Induction of Apoptosis in Immune Cells, Leading to Cytokine Storm Suppression." International Journal of Molecular Sciences **21**(17): 1-21.

538. Molina, J. A., et al. (2020). "Sustainable commuting: Results from a social approach and international evidence on carpooling." Sustainability (Switzerland) **12**(22): 1-12.

539. Mollaioli, D., et al. (2020). "Benefits of Sexual Activity on Psychological, Relational, and Sexual Health During the COVID-19 Breakout." Journal of Sexual Medicine.

540. Mondino, E., et al. (2020). "Public perceptions of multiple risks during the COVID-19 pandemic in Italy and Sweden." Scientific data **7**(1): 434.

541. Monge, S., et al. (2020). "Ambulance dispatch calls attributable to influenza A and other common respiratory viruses in the Netherlands (2014-2016)." Influenza Other Respir Viruses **14**(4): 420-428.

542. Monnet, D. L. and S. Harbarth (2020). "Will coronavirus disease (COVID-19) have an impact on antimicrobial resistance?" Euro Surveill **25**(45).

543. Monteil, V., et al. (2020). "Human soluble ACE2 improves the effect of remdesivir in SARS-CoV-2 infection." EMBO Mol Med: e13426.

544. Monteil, V., et al. (2020). "Inhibition of SARS-CoV-2 Infections in Engineered Human Tissues Using Clinical-Grade Soluble Human ACE2." Cell **181**(4): 905-913.e907.

545. Montemurro, P., et al. (2020). "Effects of COVID-19 on plastic surgery practices and medi-spas in different countries." Aesthetic Surgery Journal **40**(8): NP453-NP456.

546. Morales, D. R., et al. (2020). "Renin-angiotensin system blockers and susceptibility to COVID-19: an international, open science, cohort analysis." Lancet Digit Health.

547. Morbelli, S., et al. (2020). "COVID-19 and the brain: impact on nuclear medicine in neurology." European Journal of Nuclear Medicine and Molecular Imaging **47**(11): 2487-2492.

548. Morgantini, L. A., et al. (2020). "Factors contributing to healthcare professional burnout during the COVID-19 pandemic: A rapid turnaround global survey." PLoS One **15**(9 September).

549. Morris, S. R., et al. (2020). "Development of a standardized data collection tool for evaluation and management of coronavirus disease 2019." Open Forum Infectious Diseases **7**(9).

550. Moslem, S., et al. (2020). "Best-worst method for modelling mobility choice after COVID-19: Evidence from Italy." Sustainability (Switzerland) **12**(17).

551. Muchmore, B., et al. (2020). "Tracking potential COVID-19 outbreaks with influenzalike symptoms urgent care visits." Pediatrics **146**(4).

552. Muller, S. M. (2020). "The dangers of performative scientism as the alternative to anti-scientific policymaking: A critical, preliminary assessment of South Africa's Covid-19 response and its consequences." World Development.

553. Muniz-Pardos, B., et al. (2020). "Collateral Health Issues Derived from the Covid-19 Pandemic." Sports Medicine - Open **6**(1).

554. Muñoz Á, G., et al. (2020). "AeDES: a next-generation monitoring and forecasting system for environmental suitability of Aedes-borne disease transmission." Sci Rep **10**(1): 12640.

555. Muñoz, N. (2020). "Covid-19 in latin america: A first glance to the mortality." Colombia Medica **51**(2): 1-2.

556. Murray, J. (2020). "Has Sweden's controversial covid-19 strategy been successful or not?" The BMJ **370**.

557. Murugan, N. A., et al. (2020). "Searching for target-specific and multi-targeting organics for Covid-19 in the Drugbank database with a double scoring approach." Sci Rep **10**(1): 19125.

558. Musinguzi, G. and B. O. Asamoah (2020). "The science of social distancing and total lock down: Does it work? whom does it benefit?" Electronic Journal of General Medicine **17**(6).

559. Nadanovsky, P. and A. P. P. D. Santos (2020). "Strategies to deal with the COVID-19 pandemic." Brazilian oral research **34**: e068.

560. Naidu, S. A. G., et al. (2020). "COVID-19 during Pregnancy and Postpartum: Antiviral Spectrum of Maternal Lactoferrin in Fetal and Neonatal Defense." Journal of dietary supplements: 1-37.

561. Nakatani, R. (2020). "Macroprudential policy and the probability of a banking crisis." Journal of Policy Modeling.

562. Nanni, M., et al. (2020). "Give more data, awareness and control to individual citizens, and they will help COVID-19 containment." Transactions on Data Privacy **13**(1): 61-66.

563. Nasi, A., et al. (2020). "Reactive oxygen species as an initiator of toxic innate immune responses in retort to SARS-CoV-2 in an ageing population, consider N-acetylcysteine as early therapeutic intervention." Toxicology Reports **7**: 768-771.

564. Nataliia, S., et al. (2020). "Global and Local Determinants of Social Economy Models in Pandemic Times." SSRN.

565. Nct (2020). "Combination of Recombinant Bacterial ACE2 Receptors -Like Enzyme of B38-CAP and Isotretinoin Could be Promising COVID-19 Infection- and Lung Injury Preventing Drug Better Than Recombinant Human ACE2." https://clinicaltrials.gov/show/NCT04382950.

566. Nct (2020). "NO Prevention of COVID-19 for Healthcare Providers." https://clinicaltrials.gov/show/NCT04312243.

567. Nct (2020). "Vitamin D and COVID-19 Trial." https://clinicaltrials.gov/show/NCT04536298.

568. Neher, R. A., et al. (2020). "Potential impact of seasonal forcing on a SARS-CoV-2 pandemic." Swiss Med Wkly **150**: w20224.

569. Neil, S. J. D. and E. M. Campbell (2020). "Fake Science: XMRV, COVID-19, and the Toxic Legacy of Dr. Judy Mikovits." AIDS Research and Human Retroviruses **36**(7): 545-549.

570. Németh, N., et al. (2020). "PCV47 CHANGES IN THE MORTALITY RELATED TO CEREBROVASCULAR DISESASES IN WHO EUROPEAN REGION: 1990-2014." Value in Health **23**: S99.

571. Németh, N., et al. (2020). "PCV51 CHANGES IN THE EARLY MORTALITY RELATED TO ISCHAEMIC HEART DISEASE AMONG PEOPLE AGED 45-59 BETWEEN 1990-2014." Value in Health **23**: S99-S100.

572. Németh, N., et al. (2020). "PCV54 AGE-SPECIFIC EXAMINATION OF EARLY CEREBROVASCULAR MORTALITY: 1990-2014." Value in Health **23**: S100.

573. Neogi, U., et al. (2020). "Feasibility of known rna polymerase inhibitors as anti-sars-cov-2 drugs." Pathogens **9**(5).

574. Neubeck, L., et al. (2020). "Delivering healthcare remotely to cardiovascular patients during COVID-19: A rapid review of the evidence." European Journal of Cardiovascular Nursing **19**(6): 486-494.

575. Neumann-Podczaska, A., et al. (2020). "Clinical characteristics and survival analysis in a small sample of older COVID-19 patients with defined 60-day outcome." International Journal of Environmental Research and Public Health **17**(22): 1-12.

576. Neuwirth, C., et al. (2020). "Investigating duration and intensity of Covid-19 social-distancing strategies." Scientific reports **10**(1).

577. Ng, H., et al. (2020). "Circulating Markers of Neutrophil Extracellular Traps Are of Prognostic Value in Patients With COVID-19." Arteriosclerosis, thrombosis, and vascular biology: ATVBAHA120315267.

578. Nguyen, Q. C., et al. (2020). "Using 164 million google street view images to derive built environment predictors of COVID-19 cases." International Journal of Environmental Research and Public Health **17**(17): 1-13.

579. Nicholas, G. and A. Shapiro (2020). "Failed hybrids: The death and life of Bluetooth proximity marketing." Mobile Media and Communication.

580. Nicolay, N., et al. (2020). "Epidemiology of measles during the COVID-19 pandemic, a description of the surveillance data, 29 EU/EEA countries and the United Kingdom, January to May 2020." Euro Surveill **25**(31).

581. Niedzwiedz, C. L., et al. (2020). "Ethnic and socioeconomic differences in SARS-CoV-2 infection: Prospective cohort study using UK Biobank." BMC Medicine **18**(1).

582. Nikolopoulos, K., et al. (2020). "Forecasting and planning during a pandemic: COVID-19 growth rates, supply chain disruptions, and governmental decisions." European Journal of Operational Research.

583. Ning, L. and Y. Wang (2020). "Quantitative analysis of the COVID-19 pandemic shock to household consumption in China." Frontiers of Economics in China **15**(3): 355-379.

584. Norinder, U., et al. (2020). "Existing highly accumulating lysosomotropic drugs with potential for repurposing to target COVID-19." Biomed Pharmacother **130**: 110582.

585. Ntaios, G., et al. (2020). "Characteristics and Outcomes in Patients with COVID-19 and Acute Ischemic Stroke: The Global COVID-19 Stroke Registry." Stroke: 254-258.

586. Nyman, E., et al. (2020). "Mechanisms of a Sustained Anti-inflammatory Drug Response in Alveolar Macrophages Unraveled with Mathematical Modeling." CPT Pharmacometrics Syst Pharmacol.

587. O’Brien, M. L. and M. A. Eger (2020). "Suppression, Spikes, and Stigma: How COVID-19 Will Shape International Migration and Hostilities toward It." International Migration Review.

588. O'Connor, R. C., et al. (2020). "Multidisciplinary research priorities for the COVID-19 pandemic – Authors' reply." The Lancet Psychiatry **7**(7): e44-e45.

589. Oehmke, J. F., et al. (2020). "Dynamic panel surveillance of COVID-19 transmission in the united states to inform health policy: Observational statistical study." Journal of Medical Internet Research **22**(10).

590. Ogunleye, O. O., et al. (2020). "Response to the Novel Corona Virus (COVID-19) Pandemic Across Africa: Successes, Challenges, and Implications for the Future." Frontiers in Pharmacology **11**.

591. Ohrling, M., et al. (2020). "Management of the emergency response to the SARS-CoV-2 (COVID-19) outbreak in Stockholm, Sweden, and winter preparations." Journal of primary health care **12**(3): 207-214.

592. Olson, K., et al. (2020). "Pandemic-Driven Posttraumatic Growth for Organizations and Individuals." JAMA - Journal of the American Medical Association **324**(18): 1829-1830.

593. Oltean, M., et al. (2020). "Covid-19 in kidney transplant recipients: a systematic review of the case series available three months into the pandemic." Infectious Diseases: 1-8.

594. Omrani, A. S., et al. (2020). "The first consecutive 5000 patients with Coronavirus Disease 2019 from Qatar; a nation-wide cohort study." BMC Infectious Diseases **20**(1).

595. Ong, C. W. M., et al. (2020). "Epidemic and pandemic viral infections: Impact on tuberculosis and the lung." European Respiratory Journal **56**(4).

596. Ong, J., et al. (2020). "Burnout and work-related stressors in gastroenterology: A protocol for a multinational observational study in the ASEAN region." BMJ Open Gastroenterology **7**(1).

597. Orlowski, E. J. W. and D. J. A. Goldsmith (2020). "Four months into the COVID-19 pandemic, Sweden’s prized herd immunity is nowhere in sight." Journal of the Royal Society of Medicine **113**(8): 292-298.

598. Ossami Saidy, R. R., et al. (2020). "Successful implementation of preventive measures leads to low relevance of SARS-CoV-2 in liver transplant patients: Observations from a German outpatient department." Transplant Infectious Disease.

599. Otto, C. M. (2020). "Heartbeat: Is medical therapy for calcific aortic stenosis possible?" Heart **106**(23): 1783-1785.

600. Oum, T. H. and K. Wang (2020). "Socially optimal lockdown and travel restrictions for fighting communicable virus including COVID-19." Transport Policy **96**: 94-100.

601. Ouzounis, C. A. (2020). "A recent origin of Orf3a from M protein across the coronavirus lineage arising by sharp divergence." Computational and Structural Biotechnology Journal **18**: 4093-4102.

602. Overmyer, K. A., et al. (2020). "Large-Scale Multi-omic Analysis of COVID-19 Severity." Cell Systems.

603. Özdinç, M., et al. (2020). "Predicting the progress of COVID-19: The case for Turkey." Turkiye Klinikleri Journal of Medical Sciences **40**(2): 117-119.

604. Pachetti, M., et al. (2020). "Impact of lockdown on Covid-19 case fatality rate and viral mutations spread in 7 countries in Europe and North America." Journal of Translational Medicine **18**(1).

605. Paderno, A., et al. (2020). "Olfactory and Gustatory Outcomes in COVID-19: A Prospective Evaluation in Nonhospitalized Subjects." Otolaryngology - Head and Neck Surgery (United States) **163**(6): 1144-1149.

606. Paderno, A., et al. (2020). "Smell and taste alterations in COVID-19: a cross-sectional analysis of different cohorts." International Forum of Allergy and Rhinology **10**(8): 955-962.

607. Pairo-Castineira, E., et al. (2020). "Genetic mechanisms of critical illness in Covid-19." Nature.

608. Pakpour, A. H., et al. (2020). "A population-based nationwide dataset concerning the COVID-19 pandemic and serious psychological consequences in Bangladesh." Data Brief **33**: 106621.

609. Palacka, P., et al. (2020). "Q-VENT—A novel device for emergency ventilation of the lungs in patients with respiratory failure due to diseases such as COVID-19." Medical Devices and Sensors.

610. Palit, P., et al. (2020). "Phytopharmaceuticals mediated Furin and TMPRSS2 receptor blocking: can it be a potential therapeutic option for Covid-19?" Phytomedicine.

611. Pallarés Carratalá, V., et al. (2020). "COVID-19 and cardiovascular and kidney disease: Where are we? Where are we going?" Semergen **46**: 78-87.

612. Palmer, K., et al. (2020). "The potential long-term impact of the COVID-19 outbreak on patients with non-communicable diseases in Europe: consequences for healthy ageing." Aging Clinical and Experimental Research **32**(7): 1189-1194.

613. Panda, P. K., et al. (2020). "Structure-based drug designing and immunoinformatics approach for SARS-CoV-2." Science advances **6**(28).

614. Panossian, A. and T. Brendler (2020). "The role of adaptogens in prophylaxis and treatment of viral respiratory infections." Pharmaceuticals **13**(9): 1-32.

615. Papadopoulos, N. G., et al. (2020). "Impact of COVID-19 on Pediatric Asthma: Practice Adjustments and Disease Burden." Journal of Allergy and Clinical Immunology: In Practice **8**(8): 2592-2599.e2593.

616. Parasa, S., et al. (2020). "Prevalence of Gastrointestinal Symptoms and Fecal Viral Shedding in Patients with Coronavirus Disease 2019: A Systematic Review and Meta-analysis." JAMA Network Open **3**(6).

617. Park, R., et al. (2020). "Association of active oncologic treatment and risk of death in cancer patients with COVID-19: a systematic review and meta-analysis of patient data." Acta Oncologica.

618. Parlar, M. E., et al. (2020). "“You can’t touch this”: Delivery of inpatient neuropsychological assessment in the era of COVID-19 and beyond." Clinical Neuropsychologist **34**(7-8): 1395-1410.

619. Pasha, J., et al. (2020). "An Optimization Model and Solution Algorithms for the Vehicle Routing Problem with a 'Factory-in-a-Box'." IEEE Access **8**: 134743-134763.

620. Patel, N. (2020). "Lessons and Challenges to be learned from different countries policy implication on COVID 19 recovery cases- A cross-sectional descriptive study." ResearchSquare.

621. Paterlini, M. (2020). "Covid-19: Sweden considers tougher restrictions as ICU beds near capacity." The BMJ **371**.

622. Paterlini, M. (2020). "On the front lines of coronavirus: The Italian response to covid-19." The BMJ **368**.

623. Patrick, M. and M. David (2020). "A structural model of corona virus behaviour for testing on data behaviour."

624. Patzold, M. (2020). "The Role of 5G in Limiting the Impact of the COVID-19 Pandemic [Mobile Radio]." IEEE Vehicular Technology Magazine **15**(4): 6-12.

625. Pavliashvili, S. and D. E. Prasek (2020). "Accelerating transition to the circular economy in Georgia." Bulletin of the Georgian National Academy of Sciences **14**(3): 7-13.

626. Pavlíček, T., et al. (2020). "Oscillatory Dynamics in Infectivity and Death Rates of COVID-19." mSystems **5**(4).

627. Payne, J. L., et al. (2020). "COVID-19 and social distancing measures in Queensland, Australia, are associated with short-term decreases in recorded violent crime." Journal of Experimental Criminology.

628. Pearson, C. A. B., et al. (2020). "Projected early spread of COVID-19 in Africa through 1 June 2020." Eurosurveillance **25**(18).

629. Pedrosa, A. L., et al. (2020). "Emotional, Behavioral, and Psychological Impact of the COVID-19 Pandemic." Frontiers in Psychology **11**.

630. Percivalle, E., et al. (2020). "Prevalence of SARS-CoV-2 specific neutralising antibodies in blood donors from the Lodi Red Zone in Lombardy, Italy, as at 06 April 2020." Eurosurveillance **25**(24).

631. Pereira, P. F. D. C., et al. (2020). "Thermal comfort applied in hospital environments: A literature review." Applied Sciences (Switzerland) **10**(20): 1-22.

632. Perera, R. A., et al. (2020). "Serological assays for severe acute respiratory syndrome coronavirus 2 (SARS-CoV-2), March 2020." Euro surveillance : bulletin Europeen sur les maladies transmissibles = European communicable disease bulletin **25**(16).

633. Pergolizzi, J. V., et al. (2020). "The current clinically relevant findings on COVID-19 pandemic." Anesthesiology and Pain Medicine **10**(2).

634. Petersen, M. W., et al. (2020). "Low-dose hydrocortisone in patients with COVID-19 and severe hypoxia (COVID STEROID) trial—Protocol and statistical analysis plan." Acta Anaesthesiologica Scandinavica **64**(9): 1365-1375.

635. Petitta, L., et al. (2020). "Economic stress, emotional contagion and safety outcomes: A cross-country study." Work **66**(2): 421-435.

636. Petridou, E. (2020). "Politics and administration in times of crisis: Explaining the Swedish response to the COVID-19 crisis." European Policy Analysis.

637. Petrov, A. N., et al. (2020). "Spatiotemporal dynamics of the COVID-19 pandemic in the arctic: early data and emerging trends." International journal of circumpolar health **79**(1): 1835251.

638. Petruk, G., et al. (2020). "SARS-CoV-2 Spike protein binds to bacterial lipopolysaccharide and boosts proinflammatory activity." J Mol Cell Biol.

639. Pick, A. (2020). "Covid-19: Which country has the most effective lockdown?" The BMJ **370**.

640. Pierron, D., et al. (2020). "Smell and taste changes are early indicators of the COVID-19 pandemic and political decision effectiveness." Nature Communications **11**(1).

641. Pimenoff, V. N., et al. (2020). "Estimating total excess mortality during a COVID-19 outbreak in Stockholm, Sweden." Clinical infectious diseases : an official publication of the Infectious Diseases Society of America.

642. Ping, W., et al. (2020). "Evaluation of health-related quality of life using EQ-5D in China during the COVID-19 pandemic." PLoS One **15**(6).

643. Poletti, P., et al. (2020). "Age-specific SARS-CoV-2 infection fatality ratio and associated risk factors, Italy, February to April 2020." Eurosurveillance **25**(31).

644. Ponikowski, P., et al. (2020). "Ferric carboxymaltose for iron deficiency at discharge after acute heart failure: a multicentre, double-blind, randomised, controlled trial." The Lancet.

645. Pourghasemi, H. R., et al. (2020). "Spatial modeling, risk mapping, change detection, and outbreak trend analysis of coronavirus (COVID-19) in Iran (days between February 19 and June 14, 2020)." Int J Infect Dis **98**: 90-108.

646. Power, K., et al. (2020). "The development of an epilepsy electronic patient portal: Facilitating both patient empowerment and remote clinician-patient interaction in a post-COVID-19 world." Epilepsia **61**(9): 1894-1905.

647. Pozo-Rico, T., et al. (2020). "Teacher training can make a difference: tools to overcome the impact of COVID-19 on primary schools. An experimental study." International Journal of Environmental Research and Public Health **17**(22): 1-23.

648. Pradhan, A. and P. E. Olsson (2020). "Sex differences in severity and mortality from COVID-19: are males more vulnerable?" Biology of sex differences **11**(1): 53.

649. Prajapati, D. P., et al. (2020). "Association of subjective olfactory dysfunction and 12-item odor identification testing in ambulatory COVID-19 patients." International Forum of Allergy and Rhinology **10**(11): 1209-1217.

650. Preis, H., et al. (2020). "Psychometric properties of the Pandemic-Related Pregnancy Stress Scale (PREPS)." Journal of Psychosomatic Obstetrics and Gynecology **41**(3): 191-197.

651. Putri, K. Y. S., et al. (2020). "The antecedents and consequences of e-health literacy in the pharmaceutical industry: An agenda for future research." International Journal of Applied Pharmaceutics **12**(6): 1-6.

652. Qiu, R. T. R., et al. (2020). "Social costs of tourism during the COVID-19 pandemic." Annals of Tourism Research **84**.

653. Quilty, B. J., et al. (2020). "Effectiveness of airport screening at detecting travellers infected with novel coronavirus (2019-nCoV)." Eurosurveillance **25**(5).

654. Ragazzi, M., et al. (2020). "Municipal solid waste management during the SARS-COV-2 outbreak and lockdown ease: Lessons from Italy." Science of the Total Environment **745**.

655. Rahimi, F. and A. Talebi Bezmin Abadi (2020). "Criticality of physical/social distancing, handwashing, respiratory hygiene and face-masking during the COVID-19 pandemic and beyond." International Journal of Clinical Practice **74**(11).

656. Rahman, M. E., et al. (2020). "Physical inactivity and sedentary behaviors in the Bangladeshi population during the COVID-19 pandemic: An online cross-sectional survey." Heliyon **6**(10).

657. Ralph Edwards, I. and M. Lindquist (2020). "Dark present, but look to the future." International Journal of Risk and Safety in Medicine **31**(2): 43-44.

658. Ralph, P., et al. (2020). "Pandemic programming: How COVID-19 affects software developers and how their organizations can help." Empir Softw Eng **25**(6): 1-35.

659. Ramachandran, R. (2020). "COVID-19—a very visible pandemic." The Lancet **396**(10248): e13-e14.

660. Ramos, M., et al. (2020). "PDB52 IMPACT OF USING DIFFERENT TYPES OF HBA1C PROGRESSION IN THE IQVIA CORE DIABETES MODEL." Value in Health **23**: S117-S118.

661. Rani, U. and R. K. Dhir (2020). "Platform Work and the COVID-19 Pandemic." Indian Journal of Labour Economics **63**: 163-171.

662. Raoult, D., et al. (2020). "Coronavirus infections: Epidemiological, clinical and immunological features and hypotheses." Cell Stress **4**(4): 66-75.

663. Rapaccini, M., et al. (2020). "Navigating disruptive crises through service-led growth: The impact of COVID-19 on Italian manufacturing firms." Industrial Marketing Management **88**: 225-237.

664. Rashid-Abdi, M., et al. (2020). "Low rate of COVID-19 seroconversion in health-care workers at a Department of Infectious Diseases in Sweden during the later phase of the first wave; a prospective longitudinal seroepidemiological study." Infectious Diseases.

665. Rawaf, S., et al. (2020). "Unlocking towns and cities: Covid-19 exit strategy." Eastern Mediterranean Health Journal **26**(5): 499-502.

666. Remzi, F. H., et al. (2020). "International organization for the study of IBD recommendations for surgery in patients with IBD during the coronavirus disease 2019 pandemic." Diseases of the Colon and Rectum **63**(7): 870-873.

667. Renberg, M., et al. (2020). "Renal Resistive Index is Associated With Acute Kidney Injury in COVID-19 Patients Treated in the ICU." ResearchSquare.

668. Renigier-Biłozor, M., et al. (2020). "Hybridization of valuation procedures as a medicine supporting the real estate market and sustainable land use development during the covid-19 pandemic and afterwards." Land Use Policy **99**.

669. Reusken, C. B. E. M., et al. (2020). "Laboratory readiness and response for novel coronavirus (2019-nCoV) in expert laboratories in 30 EU/EEA countries, January 2020." Eurosurveillance **25**(6).

670. Reusken, C. B., et al. (2020). "Authors' response: Plenty of coronaviruses but no SARS-CoV-2." Eurosurveillance **25**(8).

671. Rexroth, U., et al. (2020). "Letter to the editor: Wide indication for SARS-CoV-2-testing allowed identification of international risk areas during the early phase of the COVID-19 pandemic in Germany." Eurosurveillance **25**(23).

672. Rhodes, J. M., et al. (2020). "Perspective: Vitamin D deficiency and COVID-19 severity – plausibly linked by latitude, ethnicity, impacts on cytokines, ACE2 and thrombosis." Journal of Internal Medicine.

673. Riccardo, F., et al. (2020). "Epidemiological characteristics of COVID-19 cases and estimates of the reproductive numbers 1 month into the epidemic, Italy, 28 January to 31 March 2020." Eurosurveillance **25**(49): 1-11.

674. Richard, N. A., et al. (2020). "Potential impact of seasonal forcing on a SARS-CoV-2 pandemic." Swiss Medical Weekly **150**(11-12).

675. Ricoca Peixoto, V., et al. (2020). "Initial Assessment of the Impact of the Emergency State Lockdown Measures on the 1st Wave of the COVID-19 Epidemic in Portugal." Acta Med Port **33**(11): 733-741.

676. Riddle, M. C., et al. (2020). "COVID-19 in People with Diabetes: Urgently Needed Lessons from Early Reports." Diabetes Care **43**(7): 1378-1381.

677. Riggioni, C., et al. (2020). "A compendium answering 150 questions on COVID-19 and SARS-CoV-2." Allergy **75**(10): 2503-2541.

678. Roberts, J. D. and S. O. Tehrani (2020). "Environments, behaviors, and inequalities: Reflecting on the impacts of the influenza and coronavirus pandemics in the united states." International Journal of Environmental Research and Public Health **17**(12): 1-27.

679. Rocklöv, J. and H. Sjödin (2020). "High population densities catalyse the spread of COVID-19." Journal of travel medicine **27**(3).

680. Rocklöv, J., et al. (2020). "COVID-19 outbreak on the Diamond Princess cruise ship: estimating the epidemic potential and effectiveness of public health countermeasures." J Travel Med **27**(3).

681. Rodriguez-Wallberg, K. A. and I. Wikander (2020). "A global recommendation for restrictive provision of fertility treatments during the COVID-19 pandemic." Acta Obstetricia et Gynecologica Scandinavica **99**(5): 569-570.

682. Ronchi, E. and R. Lovreglio (2020). "EXPOSED: An occupant exposure model for confined spaces to retrofit crowd models during a pandemic." Saf Sci **130**: 104834.

683. Root-Bernstein, R. (2020). "Age and Location in Severity of COVID-19 Pathology: Do Lactoferrin and Pneumococcal Vaccination Explain Low Infant Mortality and Regional Differences?" BioEssays **42**(11).

684. Rosell, A., et al. (2020). "Patients With COVID-19 Have Elevated Levels of Circulating Extracellular Vesicle Tissue Factor Activity That Is Associated With Severity and Mortality." Arteriosclerosis, thrombosis, and vascular biology: ATVBAHA120315547.

685. Rosén, M. and M. Stenbeck (2020). "Interventions to suppress the coronavirus pandemic will increase unemployment and lead to many premature deaths." Scandinavian journal of public health: 1403494820947974.

686. Roumier, M., et al. (2020). "Tocilizumab for Severe Worsening COVID-19 Pneumonia: a Propensity Score Analysis." J Clin Immunol: 1-12.

687. Routy, B., et al. (2020). "COVID-19: a challenge for oncology services." OncoImmunology **9**(1).

688. Rubino, F., et al. (2020). "Bariatric and metabolic surgery during and after the COVID-19 pandemic: DSS recommendations for management of surgical candidates and postoperative patients and prioritisation of access to surgery." The Lancet Diabetes and Endocrinology **8**(7): 640-648.

689. Rudberg, A. S., et al. (2020). "SARS-CoV-2 exposure, symptoms and seroprevalence in healthcare workers in Sweden." Nature Communications **11**(1).

690. Ruiu, M. L., et al. (2020). "Similarities and differences in managing the Covid-19 crisis and climate change risk." Journal of Knowledge Management **24**(10): 2597-2614.

691. Russell, F. M. and B. Greenwood (2020). "Who should be prioritised for COVID-19 vaccination?" Human Vaccines and Immunotherapeutics.

692. Russell, T. W., et al. (2020). "Estimating the infection and case fatality ratio for coronavirus disease (COVID-19) using age-adjusted data from the outbreak on the Diamond Princess cruise ship, February 2020." Eurosurveillance **25**(12).

693. Russo, F., et al. (2020). "Epidemiology and public health response in early phase of COVID-19 pandemic, Veneto Region, Italy, 21 February to 2 April 2020." Euro surveillance : bulletin Europeen sur les maladies transmissibles = European communicable disease bulletin **25**(47).

694. Ryan, M. (2020). "In defence of digital contact-tracing: human rights, South Korea and Covid-19." International Journal of Pervasive Computing and Communications.

695. Rypdal, K. and M. Rypdal (2020). "A Parsimonious Description and Cross-Country Analysis of COVID-19 Epidemic Curves." Int J Environ Res Public Health **17**(18).

696. Saglietto, A., et al. (2020). "COVID-19 in Europe: the Italian lesson." The Lancet **395**(10230): 1110-1111.

697. Saguti, F., et al. (2020). "Surveillance of wastewater revealed peaks of SARS-CoV-2 preceding those of hospitalized patients with COVID-19." Water Res **189**: 116620.

698. Saguti, F., et al. (2021). "Surveillance of wastewater revealed peaks of SARS-CoV-2 preceding those of hospitalized patients with COVID-19." Water Research **189**.

699. Sahlgrenska University Hospital, S. (2020). "COPE - COVID-19 in Pregnancy and Early Childhood." clinicaltrials.gov.

700. Salisbury, C., et al. (2020). "Private video consultation services and the future of primary care." Journal of Medical Internet Research **22**(10).

701. Sallam, M., et al. (2020). "COVID-19 misinformation: Mere harmless delusions or much more? A knowledge and attitude cross-sectional study among the general public residing in Jordan." PLoS One **15**(12 December).

702. Salterio, S. E. (2020). "Accounting for the unaccountable – coping with COVID." Journal of Accounting and Organizational Change.

703. Samah, I. H. A., et al. (2020). "The impact of healthcare expenditure and healthcare sector growth on CO2 emission using dynamic panel data system GMM estimation model during COVID 19 crisis." International Journal of Energy Economics and Policy **10**(6): 235-241.

704. Sánchez-Teruel, D., et al. (2020). "Do psychological strengths protect college students confined by COVID-19 to emotional distress? The role of gender." Personality and Individual Differences.

705. Santos, J. (2020). "Using input-output analysis to model the impact of pandemic mitigation and suppression measures on the workforce." Sustainable Production and Consumption **23**: 249-255.

706. Santos-Hövener, C., et al. (2020). "Serology- and PCR-based cumulative incidence of SARS-CoV-2 infection in adults in a successfully contained early hotspot (CoMoLo study), Germany, May to June 2020." Euro surveillance : bulletin Europeen sur les maladies transmissibles = European communicable disease bulletin **25**(47).

707. Santos-Roldán, L., et al. (2020). "Sustainable tourism as a source of healthy tourism." International Journal of Environmental Research and Public Health **17**(15): 1-15.

708. Sarzani, R., et al. (2020). "Disequilibrium between the classic renin-angiotensin system and its opposing arm in SARS-CoV-2-related lung injury." American Journal of Physiology - Lung Cellular and Molecular Physiology **319**(2): L325-L336.

709. Sato, Y., et al. (2020). An Education Model for Game Development by A Swedish-Japanese Industry-Academia Alliance. IEEE Conference on Computatonal Intelligence and Games, CIG.

710. Savarese, G., et al. (2020). "Association between renin–angiotensin–aldosterone system inhibitor use and COVID-19 hospitalization and death: a 1.4 million patient nationwide registry analysis." European Journal of Heart Failure.

711. Savulescu, J. (2020). "Good Reasons to Vaccinate: Mandatory or Payment for Risk?" Journal of Medical Ethics.

712. Scabini, L. F. S., et al. (2021). "Social interaction layers in complex networks for the dynamical epidemic modeling of COVID-19 in Brazil." Physica A: Statistical Mechanics and its Applications **564**.

713. Scarpone, C., et al. (2020). "A multimethod approach for county-scale geospatial analysis of emerging infectious diseases: A cross-sectional case study of COVID-19 incidence in Germany." International Journal of Health Geographics **19**(1).

714. Schippers, M. C. (2020). "For the Greater Good? The Devastating Ripple Effects of the Covid-19 Crisis." Frontiers in Psychology **11**.

715. Schraff, D. (2020). "Political trust during the Covid-19 pandemic: Rally around the flag or lockdown effects?" European Journal of Political Research.

716. Schwank, S. E., et al. (2020). "Mental health of Urban Mothers (MUM) study: A multicentre randomised controlled trial, study protocol." BMJ Open **10**(11).

717. Scott, R., et al. (2020). "Covid-19 response and recovery in smart sustainable city governance and management: Data-driven internet of things systems and machine learning-based analytics." Geopolitics, History, and International Relations **12**(2): 16-22.

718. Sebhatu, A., et al. (2020). "Explaining the homogeneous diffusion of COVID-19 nonpharmaceutical interventions across heterogeneous countries." Proceedings of the National Academy of Sciences of the United States of America **117**(35): 21201-21208.

719. Sebhatu, A., et al. (2020). "Explaining the homogeneous diffusion of COVID-19 nonpharmaceutical interventions across heterogeneous countries." Proceedings of the National Academy of Sciences of the United States of America **117**(35): 21201-21208.

720. Sedov, L., et al. (2020). "Modeling quarantine during epidemics and mass-testing using drones." PLoS One **15**(6): e0235307.

721. Şenel, K., et al. (2020). "Instantaneous r for COVID-19 in turkey: Estimation by bayesian statistical inference." Turkiye Klinikleri Journal of Medical Sciences **40**(2): 127-131.

722. Senel, K., et al. (2020). "Single Parameter Estimation Approach for Robust Estimation of SIR Model With Limited and Noisy Data: The Case for COVID-19." Disaster Med Public Health Prep: 1-15.

723. Senel, K., et al. (2020). "SPE Approach for Robust Estimation of SIR Model with Limited and Noisy Data: The Case for COVID-19." Disaster medicine and public health preparedness: 1-22.

724. Sengeh, P., et al. (2020). "Community knowledge, perceptions and practices around COVID-19 in Sierra Leone: a nationwide, cross-sectional survey." BMJ Open **10**(9): e040328.

725. Sepulveda, E. R., et al. (2020). "A Comparison of COVID-19 Mortality Rates Among Long-Term Care Residents in 12 OECD Countries." Journal of the American Medical Directors Association **21**(11): 1572-1574.e1573.

726. Seyed Hashemi, S. G., et al. (2020). "The mediating effect of the cyberchondria and anxiety sensitivity in the association between problematic internet use, metacognition beliefs, and fear of COVID-19 among Iranian online population." Heliyon **6**(10): e05135.

727. Seyran, M., et al. (2020). "The structural basis of accelerated host cell entry by SARS-CoV-2†." Febs j.

728. Shafaghi, A. H., et al. (2020). "on the effect of the respiratory droplet generation condition on COVID-19 transmission." Fluids.

729. Shah, N., et al. (2020). "Early COVID-19 outbreak, individuals’ mask attitudes and purchase intentions: a cohesive care." Journal of Science and Technology Policy Management.

730. Shahzad, K., et al. (2020). "Effects of climatological parameters on the outbreak spread of COVID-19 in highly affected regions of Spain." Environmental Science and Pollution Research **27**(31): 39657-39666.

731. Shangguan, Z., et al. (2020). "What caused the outbreak of COVID-19 in China: From the perspective of crisis management." International Journal of Environmental Research and Public Health **17**(9).

732. Sharma, A., et al. (2020). "Liver disease and outcomes among COVID-19 hospitalized patients – A systematic review and meta-analysis." Annals of Hepatology.

733. Sharma, M. M. and B. Shayak (2020). Public health implications of a delay differential equation model for COVID 19. CEUR Workshop Proceedings.

734. Sharov, K. S. (2020). "Creating and applying SIR modified compartmental model for calculation of COVID-19 lockdown efficiency." Chaos Solitons Fractals **141**: 110295.

735. She, Q., et al. (2020). "Is “Born Global” a Viable Market Entry Mode for the Internationalization of SMEs? Evidence from China before COVID-19." Emerging Markets Finance and Trade **56**(15): 3599-3612.

736. Sheridan, A., et al. (2020). "Social distancing laws cause only small losses of economic activity during the COVID-19 pandemic in Scandinavia." Proceedings of the National Academy of Sciences of the United States of America **117**(34): 20468-20473.

737. Shi, L., et al. (2020). "Laboratory Abnormalities in Pregnant Women with Novel Coronavirus Disease 2019." American Journal of Perinatology **37**(1): 1070-1073.

738. Shoer, S., et al. (2020). "A prediction model to prioritize individuals for SARS-CoV-2 test built from national symptom surveys." Med (N Y).

739. Shokoohi, M., et al. (2020). "COVID-19 Pandemic: What Can the West Learn From the East?" Int J Health Policy Manag **9**(10): 436-438.

740. Silalahi, F. E. S., et al. (2020). "GIS-based approaches on the accessibility of referral hospital using network analysis and the spatial distribution model of the spreading case of COVID-19 in Jakarta, Indonesia." BMC Health Services Research **20**(1).

741. Silva, V. and N. Paul (2020). "Potential impact of earthquakes during the 2020 COVID-19 pandemic." Earthquake Spectra.

742. Silva, W. (2020). "Per capita death and infection rates should be avoided in international comparisons." Public Health **186**: 18-19.

743. Singh, S., et al. (2020). "Revisiting the role of vitamin D levels in the prevention of COVID-19 infection and mortality in European countries post infections peak." Aging Clinical and Experimental Research **32**(8): 1609-1612.

744. Sinnathamby, M. A., et al. (2020). "All-cause excess mortality observed by age group and regions in the first wave of the COVID-19 pandemic in England." Eurosurveillance **25**(28).

745. Siripongdee, K., et al. (2020). "A blended learning model with IoT-based technology: Effectively used when the COVID-19 pandemic?" Journal for the Education of Gifted Young Scientists **8**(2): 905-917.

746. Sivan, M., et al. (2020). "Development of an integrated rehabilitation pathway for individuals recovering from COVID-19 in the community." Journal of rehabilitation medicine **52**(8): jrm00089.

747. Sjödin, H., et al. (2020). "COVID-19 healthcare demand and mortality in Sweden in response to non-pharmaceutical mitigation and suppression scenarios." Int J Epidemiol **49**(5): 1443-1453.

748. Sjödin, H., et al. (2020). "Erratum to: COVID-19 healthcare demand and mortality in Sweden in response to non-pharmaceutical mitigation and suppression scenarios." International journal of epidemiology.

749. Sjödin, H., et al. (2020). "Only strict quarantine measures can curb the coronavirus disease (COVID-19) outbreak in Italy, 2020." Eurosurveillance **25**(13).

750. Skevaki, C., et al. (2020). "Asthma-associated risk for COVID-19 development." Journal of Allergy and Clinical Immunology **146**(6): 1295-1301.

751. Skoog, I. (2020). "COVID-19 and mental health among older people in Sweden." International Psychogeriatrics **32**(10): 1173-1175.

752. Smith, M. L., et al. (2020). "Biosurfactants: A Covid-19 Perspective." Frontiers in Microbiology **11**.

753. Smolander, J. and A. Bruchfeld (2020). "COVID-19 short-term outcomes of AKI and chronic hemodialysis." Journal of the American Society of Nephrology **31**: 291.

754. Sm-Rahman, A., et al. (2020). "Home-based care for people with alzheimer’s disease and related dementias (Adrd) during covid-19 pandemic: From challenges to solutions." International Journal of Environmental Research and Public Health **17**(24): 1-11.

755. Sokolski, M., et al. (2020). "Impact of Coronavirus Disease 2019 (COVID-19) Outbreak on Acute Admissions at the Emergency and Cardiology Departments Across Europe." American Journal of Medicine.

756. Song Hee, H., et al. (2020). "A New Tech-Powered Epidemic Control Versus Flattening the Curve: Insights from South Korea's Experience in COVID-19 Control." SSRN.

757. Søreide, K., et al. (2020). "Immediate and long-term impact of the COVID-19 pandemic on delivery of surgical services." British Journal of Surgery **107**(10): 1250-1261.

758. Soto-Rubio, A., et al. (2020). "Effect of emotional intelligence and psychosocial risks on burnout, job satisfaction, and nurses’ health during the covid-19 pandemic." International Journal of Environmental Research and Public Health **17**(21): 1-14.

759. Sowerby, L. J., et al. (2020). "International registry of otolaryngologist–head and neck surgeons with COVID-19." International Forum of Allergy and Rhinology **10**(11): 1201-1208.

760. Spaccaferri, G., et al. (2020). "Early assessment of the impact of mitigation measures to control COVID-19 in 22 French metropolitan areas, October to November 2020." Euro surveillance : bulletin Europeen sur les maladies transmissibles = European communicable disease bulletin **25**(50).

761. Spelsberg, A. and U. Keil (2020). "Should we continue covid-19 suppression measures based on a transmission model that ignored pre-existing human immunity?" The BMJ **371**.

762. Spence, D. (2020). "Covid-19 in Sweden and the UK: Risk, the Game of Life." The BMJ **371**.

763. Spiteri, G., et al. (2020). "First cases of coronavirus disease 2019 (COVID-19) in the WHO European Region, 24 January to 21 February 2020." Euro Surveill **25**(9).

764. Spooner, A. (2020). "Covid-19 in Sweden and UK: medical leadership should energise wider debate." The BMJ **371**.

765. Squazzoni, F., et al. (2020). "Computational models that matter during a global pandemic outbreak: A call to action." JASSS **23**(2).

766. Sramka, M., et al. (2020). "Possible consequences of Covid-19 on the nervous system." Neuro endocrinology letters **41**(4): 166-172.

767. Srinivasan, P. and C. D. Smolke (2020). "Biosynthesis of medicinal tropane alkaloids in yeast." Nature **585**(7826): 614-619.

768. Stackelberg, O., et al. (2020). "Rapid point-of-care serology testing for sars-cov-2." Lakartidningen **117**.

769. Staller, N. and C. Randler (2020). "Changes in sleep schedule and chronotype due to COVID-19 restrictions and home office." Somnologie.

770. Stambulova, N. B., et al. (2020). "The COVID-19 pandemic and Olympic/Paralympic athletes’ developmental challenges and possibilities in times of a global crisis-transition." International Journal of Sport and Exercise Psychology.

771. Stebbing, J., et al. (2020). "JAK inhibition reduces SARS-CoV-2 liver infectivity and modulates inflammatory responses to reduce morbidity and mortality." Science advances.

772. Stebbing, J., et al. (2020). "Mechanism of baricitinib supports artificial intelligence-predicted testing in COVID-19 patients." EMBO Molecular Medicine **12**(8).

773. Steffens, I. (2020). "Editorial A hundred days into the coronavirus disease (COVID-19) pandemic." Eurosurveillance **25**(14): 1-4.

774. Stephen, S., et al. (2020). "COVID-19: Weighing the endeavors of nations, with time to event analysis." Osong Public Health and Research Perspectives **11**(4): 149-157.

775. Sterne, J. A. C., et al. (2020). "Corticosteroid therapy for critically ill patients with COVID-19: A structured summary of a study protocol for a prospective meta-analysis of randomized trials." Trials **21**(1): 734.

776. Stervbo, U., et al. (2020). "Epitope similarity cannot explain the pre-formed T cell immunity towards structural SARS-CoV-2 proteins." Scientific reports **10**(1).

777. Stockwell, T., et al. (2020). "The burden of alcohol on health care during COVID-19." Drug and alcohol review.

778. Stoecklin, S. B., et al. (2020). "First cases of coronavirus disease 2019 (COVID-19) in France: Surveillance, investigations and control measures, January 2020." Eurosurveillance **25**(6).

779. Storci, G., et al. (2020). "The role of extracellular DNA in COVID-19: clues from inflamm-aging." Ageing Res Rev: 101234.

780. Strang, P., et al. (2020). "Dying From COVID-19: Loneliness, End-of-Life Discussions, and Support for Patients and Their Families in Nursing Homes and Hospitals. A National Register Study." Journal of Pain and Symptom Management **60**(4): e2-e13.

781. Strang, P., et al. (2020). "Excess deaths from COVID-19 correlate with age and socio-economic status. A database study in the Stockholm region." Upsala journal of medical sciences **125**(4): 297-304.

782. Strang, P., et al. (2020). "Symptom Relief Is Possible in Elderly Dying COVID-19 Patients: A National Register Study." Journal of palliative medicine.

783. Struben, J. (2020). "The coronavirus disease (COVID-19) pandemic: simulation-based assessment of outbreak responses and postpeak strategies." Syst Dyn Rev.

784. Sujit Kumar, N., et al. (2020). "Infection Kinetics of COVID-19 and Lockdown Implications." SSRN.

785. Sulentic, R. O., et al. (2020). "Perinatal COVID-19 outcomes: evaluating the strength of current evidence." Journal of Maternal-Fetal and Neonatal Medicine.

786. Sunden-Cullberg, J. (2020). "Chronic Use of Angiotensin-Converting Enzyme Inhibitors and Angiotensin II Receptor Blockers Is High among Intensive Care Unit Patients with Non-COVID-19 Sepsis but Carries a Moderately Increased Risk of Death." Hypertension **75**(6): e15-e16.

787. Swar, M. O. (2020). "COVID-19 and lessons learned from the pandemic wave of meningococcal meningitis (1985-1990)." Sudan J Paediatr **20**(1): 77-88.

788. Swedo, E., et al. (2020). "Trends in U.S. Emergency Department Visits Related to Suspected or Confirmed Child Abuse and Neglect Among Children and Adolescents Aged <18 Years Before and During the COVID-19 Pandemic - United States, January 2019-September 2020." MMWR Morb Mortal Wkly Rep **69**(49): 1841-1847.

789. Sycinska-Dziarnowska, M. and I. Paradowska-Stankiewicz (2020). "Dental Challenges and the Needs of the Population during the Covid-19 Pandemic Period. Real-Time Surveillance Using Google Trends." Int J Environ Res Public Health **17**(23).

790. Szepietowski, J. C., et al. (2020). "Face mask-induced itch: A self-questionnaire study of 2,315 responders during the COVID-19 pandemic." Acta Dermato-Venereologica **100**(10): 1-5.

791. Szymkowiak, A., et al. (2020). "The impact of emotions on shopping behavior during epidemic. What a business can do to protect customers." Journal of Consumer Behaviour.

792. Tadiri, C. P., et al. (2020). "The influence of sex and gender domains on COVID-19 cases and mortality." CMAJ **192**(36): E1041-E1045.

793. Taha, M. K. and A. E. Deghmane (2020). "Impact of COVID-19 pandemic and the lockdown on invasive meningococcal disease." BMC Research Notes **13**(1).

794. Takemoto, M. L. S., et al. (2020). "Clinical characteristics and risk factors for mortality in obstetric patients with severe COVID-19 in Brazil: a surveillance database analysis." BJOG: An International Journal of Obstetrics and Gynaecology **127**(13): 1618-1626.

795. Takemoto, M. L. S., et al. (2020). "Maternal mortality and COVID-19." Journal of Maternal-Fetal and Neonatal Medicine: 1-7.

796. Taleb, N. N., et al. (2020). "On single point forecasts for fat-tailed variables." International Journal of Forecasting.

797. Tamagusko, T. and A. Ferreira (2020). "Data-driven approach to understand the mobility patterns of the portuguese population during the covid-19 pandemic." Sustainability (Switzerland) **12**(22): 1-12.

798. Tan, B. Y. Q., et al. (2020). "Burnout and Associated Factors Among Health Care Workers in Singapore During the COVID-19 Pandemic." Journal of the American Medical Directors Association **21**(12): 1751-1758.e1755.

799. Tehrani, S., et al. (2021). "Risk factors for death in adult COVID-19 patients: Frailty predicts fatal outcome in older patients." International Journal of Infectious Diseases **102**: 415-421.

800. Termorshuizen, J. D., et al. (2020). "Early impact of COVID-19 on individuals with self-reported eating disorders: A survey of ~1,000 individuals in the United States and the Netherlands." International Journal of Eating Disorders **53**(11): 1780-1790.

801. Thapa, S. B., et al. (2020). "Maternal mental health in the time of the COVID-19 pandemic." Acta Obstetricia et Gynecologica Scandinavica **99**(7): 817-818.

802. Thomas, G., et al. (2020). "The novel immunomodulatory biologic LMWF5A for pharmacological attenuation of the "cytokine storm" in COVID-19 patients: a hypothesis." Patient Saf Surg **14**: 21.

803. Thompson, R. N., et al. (2019). "Improved inference of time-varying reproduction numbers during infectious disease outbreaks." Epidemics **29**: 100356.

804. Thompson, R. N., et al. (2020). "Key questions for modelling COVID-19 exit strategies." Proc Biol Sci **287**(1932): 20201405.

805. Tobias, G. and A. B. Spanier (2020). "Developing a mobile app (iGAM) to promote gingival health by professional monitoring of dental selfies: User-centered design approach." JMIR mHealth and uHealth **8**(8).

806. Toftaker, I., et al. (2020). "Herd level estimation of probability of disease freedom applied on the Norwegian control program for bovine respiratory syncytial virus and bovine coronavirus." Prev Vet Med **181**: 104494.

807. Tola, M., et al. (2020). "Molecular detection of drug resistant polymorphisms in Plasmodium falciparum isolates from Southwest, Nigeria." BMC Research Notes **13**(1).

808. Tolksdorf, K., et al. (2020). "Influenza-associated pneumonia as reference to assess seriousness of coronavirus disease (COVID-19)." Eurosurveillance **25**(11).

809. Tomasoni, D., et al. (2020). "Impact of heart failure on the clinical course and outcomes of patients hospitalized for COVID-19. Results of the Cardio-COVID-Italy multicentre study." European Journal of Heart Failure.

810. Tony, B., et al. (2020). "Integrated Quantification of the Health and Economic Impacts of Differing Strategies to Control the COVID-19 Pandemic." SSRN.

811. Top, K. A., et al. (2020). "Active surveillance of acute paediatric hospitalisations demonstrates the impact of vaccination programmes and informs vaccine policy in Canada and Australia." Eurosurveillance **25**(24).

812. Tostmann, A., et al. (2020). "Strong associations and moderate predictive value of early symptoms for SARS-CoV-2 test positivity among healthcare workers, the Netherlands, March 2020." Euro surveillance : bulletin Europeen sur les maladies transmissibles = European communicable disease bulletin **25**(16).

813. Townsend, M. J., et al. (2020). "Outcomes of COVID-19: disparities in obesity and by ethnicity/race." International Journal of Obesity **44**(9): 1807-1809.

814. Tran, B. X., et al. (2020). "Studies of novel coronavirus disease 19 (Covid-19) pandemic: A global analysis of literature." International Journal of Environmental Research and Public Health **17**(11): 1-20.

815. Tran, T. H., et al. (2020). "Associations between restrictions on public mobility and slowing of new COVID-19 case rates in three countries." Medical Journal of Australia **213**(10): 471-473.

816. Tschöpe, C., et al. (2020). "Myocarditis and inflammatory cardiomyopathy: current evidence and future directions." Nature Reviews Cardiology.

817. Tuccori, M., et al. (2020). "The Impact of the COVID-19 “Infodemic” on Drug-Utilization Behaviors: Implications for Pharmacovigilance." Drug Safety **43**(8): 699-709.

818. Tuite, A. R., et al. (2020). "Estimation of COVID-19 burden in Egypt - Authors' reply." Lancet Infect Dis **20**(8): 897-898.

819. Tverring, J., et al. (2020). "Helmet continuous positive airway pressure versus high-flow nasal cannula in COVID-19: a pragmatic randomised clinical trial (COVID HELMET)." Trials **21**(1).

820. Udrea, A. M., et al. (2020). "Laser irradiated phenothiazines: New potential treatment for COVID-19 explored by molecular docking." J Photochem Photobiol B **211**: 111997.

821. Ulfberg, J. and R. Stehlik (2020). "Finland's handling of selenium is a model in these times of coronavirus infections." Br J Nutr: 1-2.

822. Ürün, Y., et al. (2020). "Survey of the impact of COVID-19 on oncologists' decision making in cancer." JCO Global Oncology(6): 1248-1257.

823. Uzzaman, M. N., et al. (2020). "Continuing professional education for general practitioners on chronic obstructive pulmonary disease: Feasibility of a blended learning approach in Bangladesh." BMC Family Practice **21**(1).

824. Vaid, S., et al. (2020). "Risk of a second wave of Covid-19 infections: using artificial intelligence to investigate stringency of physical distancing policies in North America." Int Orthop **44**(8): 1581-1589.

825. Väliverronen, E., et al. (2020). "Liberalists and data-solutionists: redefining expertise in Twitter debates on coronavirus in Finland." Journal of Science Communication **19**(5): 1-21.

826. Van Bulck, L., et al. (2020). "Impact of the COVID-19 pandemic on ongoing cardiovascular research projects: considerations and adaptations." European Journal of Cardiovascular Nursing **19**(6): 465-468.

827. Van Damme, W., et al. (2020). "The COVID-19 pandemic: Diverse contexts; Different epidemics - How and why?" BMJ Global Health **5**(7).

828. Vanni, G., et al. (2020). "Lockdown of breast cancer screening for COVID-19: Possible scenario." In Vivo **34**(5): 3047-3053.

829. Vannoni, M., et al. (2020). "Using volunteered geographic information to assess mobility in the early phases of the COVID-19 pandemic: A cross-city time series analysis of 41 cities in 22 countries from March 2nd to 26th 2020." Globalization and Health **16**(1).

830. Varsavsky, T., et al. (2020). "Detecting COVID-19 infection hotspots in England using large-scale self-reported data from a mobile application: a prospective, observational study." Lancet Public Health.

831. Verelst, F., et al. (2020). "Indications for healthcare surge capacity in European countries facing an exponential increase in coronavirus disease (COVID-19) cases, March 2020." Eurosurveillance **25**(13).

832. Verikios, G. (2020). "The dynamic effects of infectious disease outbreaks: The case of pandemic influenza and human coronavirus." Socio-Economic Planning Sciences **71**.

833. Verna, E. C., et al. (2020). "Clinical Research in Hepatology in the COVID-19 Pandemic and Post-Pandemic Era: Challenges and the Need for Innovation." Hepatology **72**(5): 1819-1837.

834. Vestergaard, L. S. and K. Mølbak (2020). "Timely monitoring of total mortality associated with COVID-19: Informing public health and the public." Eurosurveillance **25**(34).

835. Vestergaard, L. S., et al. (2020). "Excess all-cause mortality during the COVID-19 pandemic in Europe – preliminary pooled estimates from the EuroMOMO network, March to April 2020." Eurosurveillance **25**(26).

836. Vihinen, M. (2020). "Strategy for Disease Diagnosis, Progression Prediction, Risk Group Stratification and Treatment-Case of COVID-19." Front Med (Lausanne) **7**: 294.

837. Villani, E. R., et al. (2020). "Impact of COVID-19-Related Lockdown on Psychosocial, Cognitive, and Functional Well-Being in Adults With Down Syndrome." Front Psychiatry **11**: 578686.

838. Vinceti, M., et al. (2020). "Lockdown timing and efficacy in controlling COVID-19 using mobile phone tracking." EClinicalMedicine **25**: 100457.

839. Volpp, K. G., et al. (2020). "Innovation in Home Care: Time for a New Payment Model." JAMA - Journal of the American Medical Association **323**(24): 2474-2475.

840. von Meijenfeldt, F. A., et al. (2020). "Prothrombotic changes in patients with COVID-19 are associated with disease severity and mortality." Research and Practice in Thrombosis and Haemostasis.

841. Walach, H. and S. Hockertz (2020). "Wuhan Covid19 data – more questions than answers." Toxicology **440**.

842. Wallentin, L., et al. (2020). "Angiotensin-converting enzyme 2 (ACE2) levels in relation to risk factors for COVID-19 in two large cohorts of patients with atrial fibrillation." European Heart Journal **41**(41): 4037-4046.

843. Wallis, C. J. D., et al. (2020). "Risks from Deferring Treatment for Genitourinary Cancers: A Collaborative Review to Aid Triage and Management During the COVID-19 Pandemic[Formula presented]." European Urology **78**(1): 29-42.

844. Wang, B., et al. (2020). "Airborne particulate matter, population mobility and COVID-19: a multi-city study in China." BMC Public Health **20**(1).

845. Wang, Y., et al. (2020). "COVID-19 outbreak-related psychological distress among healthcare trainees: a cross-sectional study in China." BMJ Open **10**(10): e041671.

846. Wang, Y., et al. (2020). "COVID-19 outbreak-related psychological distress among healthcare trainees: a cross-sectional study in China." BMJ Open **10**(10).

847. Wasserman, D., et al. (2020). "Adaptation of evidence-based suicide prevention strategies during and after the COVID-19 pandemic." World Psychiatry **19**(3): 294-306.

848. Wensman, J. J. and M. Stokstad (2020). "Could Naturally Occurring Coronaviral Diseases in Animals Serve as Models for COVID-19? A Review Focusing on the Bovine Model." Pathogens **9**(12).

849. Westgren, M. and G. Acharya (2020). "Intensive care unit admissions for pregnant and nonpregnant women with coronavirus disease 2019." American Journal of Obstetrics and Gynecology **223**(5): 779-780.

850. Westgren, M., et al. (2020). "Severe maternal morbidity and mortality associated with COVID-19: The risk should not be downplayed." Acta Obstetricia et Gynecologica Scandinavica **99**(7): 815-816.

851. While, A. (2020). "Is altruism dying?" British Journal of Community Nursing **25**(12): 622-622.

852. Whitelaw, S., et al. (2020). "Applications of digital technology in COVID-19 pandemic planning and response." The Lancet Digital Health **2**(8): e435-e440.

853. Wieland, T. (2020). "A phenomenological approach to assessing the effectiveness of COVID-19 related nonpharmaceutical interventions in Germany." Safety Science **131**.

854. Wijkmark, C. H., et al. (2020). Remote virtual simulation for incident commanders: Opportunities and possibilities. 11th IEEE International Conference on Cognitive Infocommunications, CogInfoCom 2020 - Proceedings.

855. Wikramaratna, P. S., et al. (2020). "Estimating the false-negative test probability of SARS-CoV-2 by RT-PCR." Euro surveillance : bulletin Europeen sur les maladies transmissibles = European communicable disease bulletin **25**(50).

856. Wille, M., et al. (2020). "Evolutionary genetics of canine respiratory coronavirus and recent introduction into Swedish dogs." Infect Genet Evol **82**: 104290.

857. Willis, M., et al. (2020). "PDB26 ESTIMATED RENAL AND CARDIOVASCULAR OUTCOMES AND COST OFFSETS IN PATIENTS WITH TYPE 2 DIABETES (T2D) AND DIABETIC NEPHROPATHY (DKD) TREATED WITH CANAGLIFLOZIN." Value in Health **23**: S112.

858. Wingfield, T., et al. (2020). "Tackling two pandemics: a plea on World Tuberculosis Day." The Lancet Respiratory Medicine **8**(6): 536-538.

859. Winters, M., et al. (2020). "Creating misinformation: How a headline in the BMJ about covid-19 spread virally." The BMJ **369**.

860. Wirsiy, F. S., et al. (2020). "COVID-19 pandemic: implementing control measures in Africa using the 'SHEF2' model." Pan Afr Med J **37**(Suppl 1): 3.

861. Wollenberg, A., et al. (2020). "European Task Force on Atopic Dermatitis statement on severe acute respiratory syndrome coronavirus 2 (SARS-Cov-2) infection and atopic dermatitis." Journal of the European Academy of Dermatology and Venereology **34**(6): e241-e242.

862. Wood, Y. I., et al. (2020). "Conventional, remote, virtual and simulated work-integrated learning: A meta-analysis of existing practice." International Journal of Work-Integrated Learning **21**(4): 331-354.

863. Woodcock, B. G. (2020). "The COVID-19 pandemonium pandemic." International Journal of Clinical Pharmacology and Therapeutics **58**(7): 363-365.

864. Wu, P., et al. (2020). "Real-time tentative assessment of the epidemiological characteristics of novel coronavirus infections in Wuhan, China, as at 22 January 2020." Eurosurveillance **25**(3).

865. Wurtzer, S., et al. (2020). "Evaluation of lockdown effect on SARS-CoV-2 dynamics through viral genome quantification in waste water, Greater Paris, France, 5 March to 23 April 2020." Euro surveillance : bulletin Europeen sur les maladies transmissibles = European communicable disease bulletin **25**(50).

866. Xia, Y., et al. (2020). "How to Understand “Herd Immunity” in COVID-19 Pandemic." Frontiers in Cell and Developmental Biology **8**.

867. Xie, J. and Y. Zhu (2020). "Association between ambient temperature and COVID-19 infection in 122 cities from China." Science of the Total Environment **724**.

868. Xing, Y., et al. (2020). "Post-discharge surveillance and positive virus detection in two medical staff recovered from coronavirus disease 2019 (COVID-19), China, January to February 2020." Eurosurveillance **25**(10).

869. Yadav, S., et al. (2020). "Internet of things (IoT) based coordination system in Agri-food supply chain: development of an efficient framework using DEMATEL-ISM." Operations Management Research.

870. Yamagishi, T., et al. (2020). "Descriptive study of COVID-19 outbreak among passengers and crew on Diamond Princess cruise ship, Yokohama Port, Japan, 20 January to 9 February 2020." Eurosurveillance **25**(23).

871. Yan, A. F., et al. (2020). "Perceived risk, behavior changes and Health-related outcomes during COVID-19 pandemic: Findings among adults with and without diabetes in China." Diabetes Research and Clinical Practice **167**.

872. Yang, Y. T., et al. (2020). "Characteristic changes of traumatic dental injuries in a teaching hospital of Wuhan under transmission control measures during the COVID-19 epidemic." Dental Traumatology **36**(6): 584-589.

873. Yaya, S., et al. (2020). "Ethnic and racial disparities in COVID-19-related deaths: Counting the trees, hiding the forest." BMJ Global Health **5**(6).

874. Yu, D., et al. (2020). "Low prevalence of bloodstream infection and high blood culture contamination rates in patients with COVID-19." PLoS One **15**(11 November).

875. Zaigham, M. and O. Andersson (2020). "Maternal and perinatal outcomes with COVID-19: A systematic review of 108 pregnancies." Acta Obstetricia et Gynecologica Scandinavica **99**(7): 823-829.

876. Zala, D., et al. (2020). "Costing the COVID-19 Pandemic: An Exploratory Economic Evaluation of Hypothetical Suppression Policy in the United Kingdom." Value in Health **23**(11): 1432-1437.

877. Zhang, J., et al. (2020). "Quality Assessment of the Chinese Clinical Trial Protocols Regarding Treatments for Coronavirus Disease 2019." Frontiers in Pharmacology **11**.

878. Zhang, J., et al. (2020). "The interaction of RAAS inhibitors with COVID-19: Current progress, perspective and future." Life Sciences **257**.

879. Zhang, Q., et al. (2020). "Inborn errors of type I IFN immunity in patients with life-threatening COVID-19." Science **370**(6515).

880. Zhang, X., et al. (2020). "A preliminary simulation study about the impact of COVID-19 crisis on energy demand of a building mix at a district in Sweden." Appl Energy **280**: 115954.

881. Zhang, Y., et al. (2020). "Safety, tolerability, and immunogenicity of an inactivated SARS-CoV-2 vaccine in healthy adults aged 18–59 years: a randomised, double-blind, placebo-controlled, phase 1/2 clinical trial." The Lancet Infectious Diseases.

882. Zhao, N. and F. You (2021). "Food-energy-water-waste nexus systems optimization for New York State under the COVID-19 pandemic to alleviate health and environmental concerns." Applied Energy **282**.

883. Zhao, X., et al. (2020). "Perceived stress and sleep quality among the non-diseased general public in China during the 2019 coronavirus disease: a moderated mediation model." Sleep Medicine.

884. Zhou, J., et al. (2020). "SARS-CoV-2 Spike Protein Evolution may Cause Difficulties for Vaccine." ResearchSquare.

885. Zhou, S. J., et al. (2020). "Prevalence and socio-demographic correlates of psychological health problems in Chinese adolescents during the outbreak of COVID-19." European Child and Adolescent Psychiatry **29**(6): 749-758.

886. Zhu, X. and K. Liu (2020). "A systematic review and future directions of the sharing economy: business models, operational insights and environment-based utilities." Journal of Cleaner Production.

887. Zhu, Y., et al. (2020). "Association between short-term exposure to air pollution and COVID-19 infection: Evidence from China." Science of the Total Environment **727**.

888. Zhu, Y., et al. (2020). "The mediating effect of air quality on the association between human mobility and COVID-19 infection in China." Environmental Research **189**.

889. Zimon, G., et al. (2020). "An influence of group purchasing organizations on financial security of SMEs operating in the renewable energy sector-case for Poland." Energies **13**(11).

890. Zobbi, M. A., et al. (2020). "Measurement method for evaluating the lockdown policies during the COVID-19 pandemic." International Journal of Environmental Research and Public Health **17**(15): 1-9.

891. Zoghbi, W. A., et al. (2020). "Multimodality Cardiovascular Imaging in the Midst of the COVID-19 Pandemic: Ramping Up Safely to a New Normal." JACC: Cardiovascular Imaging **13**(7): 1615-1626.

892. Zweifel, P. (2020). "The COVID-19 crisis: A public choice view." Economic Affairs **40**(3): 395-405.

## Preprints n=566

1.    1-C Nonlinear Covid-19 Epidemic Model and Application to the Epidemic Prediction in France. Jean-Pierre Quadrat. medRxiv 2020.05.24.20111807; doi:<https://doi.org/10.1101/2020.05.24.20111807>.

2.     [4P Model for Dynamic Prediction of the Covid-19: A Statistical and Machine Learning Approach](https://discover.hsls.pitt.edu/vivisimo/cgi-bin/query-meta?v%3afile=viv_vnkHDb&v%3astate=root%7croot&url=https%3a%2f%2fdoi.org%2f10.21203%2frs.3.rs-55610%2fv3&rid=Ndoc2&v%3aframe=redirect&v%3aredirect-hash=57b897e9f4a554f9609776f949007283&) [new window](https://discover.hsls.pitt.edu/vivisimo/cgi-bin/query-meta?v%3afile=viv_vnkHDb&v%3astate=root%7croot&url=https%3a%2f%2fdoi.org%2f10.21203%2frs.3.rs-55610%2fv3&rid=Ndoc2&v%3aframe=redirect&v%3aredirect-hash=57b897e9f4a554f9609776f949007283&). Hasan KT, Rahman MM, Ahmmed MM, Chowdhury AA, Islam MK. Research Square. Doi:<https://doi.org/10.21203/rs.3.rs-55610/v3>

3.      A cell phone data driven time use analysis of the COVID-19 epidemic. Eli P. Fenichel, Kevin Berry, Jude Bayham, Gregg Gonsalves. medRxiv 2020.04.20.20073098; doi:<https://doi.org/10.1101/2020.04.20.20073098>

4.     [A Clash of Minds in a Post-Virus World](https://discover.hsls.pitt.edu/vivisimo/cgi-bin/query-meta?v%3afile=viv_vnkHDb&v%3astate=root%7croot&url=https%3a%2f%2fdoi.org%2f10.2139%2fssrn.3721628&rid=Ndoc13&v%3aframe=redirect&v%3aredirect-hash=6962c974d009a5614f71ec54e107f725&) [new window](https://discover.hsls.pitt.edu/vivisimo/cgi-bin/query-meta?v%3afile=viv_vnkHDb&v%3astate=root%7croot&url=https%3a%2f%2fdoi.org%2f10.2139%2fssrn.3721628&rid=Ndoc13&v%3aframe=redirect&v%3aredirect-hash=6962c974d009a5614f71ec54e107f725&). Dargel D, Mahammadalizada N, Tandon P, Cho S. SSRN. Doi:<https://doi.org/10.2139/ssrn.3721628>

5.      A Comparison of Monthly Global Indicators for Forecasting Growthnew window. Baumeister C, Guérin P. SSRN. Doi:<https://doi.org/10.2139/ssrn.3718840>

6.     [A Counterfactual Economic Analysis of Covid-19 Using a Threshold Augmented Multi-Country Model](https://discover.hsls.pitt.edu/vivisimo/cgi-bin/query-meta?v%3afile=viv_vnkHDb&v%3astate=root%7croot&url=https%3a%2f%2fdoi.org%2f10.2139%2fssrn.3697706&rid=Ndoc21&v%3aframe=redirect&v%3aredirect-hash=7d670e5c817ae38792805d28786d38ab&). Chudik A, Mohaddes K, Pesaran MH, Raissi M, Rebucci A. SSRN 2020-09-23. Doi: https://doi.org/10.2139/ssrn.3697706 .

7.      A COVID-19 Model for Local Authorities of the United Kingdom. Swapnil Mishra, Jamie Scott, Harrison Zhu, Neil M. Ferguson, Samir Bhatt, Seth Flaxman, Axel Gandy. medRxiv 2020.11.24.20236661; doi:<https://doi.org/10.1101/2020.11.24.20236661>

8.      A COVID-19 transmission model informing medication development and supply chain needs. Annabelle Lemenuel-Diot, Barry Clinch, Aeron C. Hurt, Paul Boutry, Johann Laurent, Mathias Leddin, Stefan Frings, Jean Eric Charoin. medRxiv 2020.11.23.20237404; doi:<https://doi.org/10.1101/2020.11.23.20237404>

9.      A data first approach to modelling Covid-19. Jayanti Prasad. medRxiv 2020.05.22.20110171; doi:<https://doi.org/10.1101/2020.05.22.20110171>

10.   A delayed modulation of solar radiation on the COVID-19 transmission reflects an incubation period. Maosheng He, Keyan Fang, Feifei Zhou, Tinghai Ou, Deliang Chen. medRxiv 2020.10.13.20183111; doi:<https://doi.org/10.1101/2020.10.13.20183111>

11.   A demographic scaling model for estimating the total number of COVID-19 infections. Christina Bohk-Ewald, Christian Dudel, Mikko Myrskylä. medRxiv 2020.04.23.20077719; doi:<https://doi.org/10.1101/2020.04.23.20077719>

12.   A geotemporal survey of hospital bed saturation across England during the first wave of the COVID-19 Pandemic. Bilal A Mateen, Harrison Wilde, John m Dennis, Andrew Duncan, Nicholas John Meyrick Thomas, Andrew P McGovern, Spiros Denaxas, Matt J Keeling, Sebastian J Vollmer. medRxiv 2020.06.24.20139048; doi:<https://doi.org/10.1101/2020.06.24.20139048>

13.   A Global Scale Estimate of Novel Coronavirus (COVID-19) Cases Using Extreme Value Distributions. M. Aadhityaa, K. S. Kasiviswanathan, Idhayachandhiran Ilampooranan, B. Soundharajan, M. Balamurugan, Jianxun He. medRxiv 2020.04.17.20069500; doi:<https://doi.org/10.1101/2020.04.17.20069500>

14.   A Heuristic Model for Spreading of COVID 19 in Singapore. Fook Hou Lee. medRxiv 2020.04.15.20067264; doi:<https://doi.org/10.1101/2020.04.15.20067264>

15.   A Large-Scale Clinical Validation Study Using nCapp Cloud Plus Terminal by Frontline Doctors for the Rapid Diagnosis of COVID-19 and COVID-19 pneumonia in China. Dawei Yang, Tao Xu, Xun Wang, Deng Chen, Ziqiang Zhang, Lichuan Zhang, Jie Liu, Kui Xiao, Li Bai, Yong Zhang, Lin Zhao, Lin Tong, Chaomin Wu, Yaoli Wang, Chunling Dong, Maosong Ye, Yu Xu, Zhenju Song, Hong Chen, Jing Li, Jiwei Wang, Fei Tan, Hai Yu, Jian Zhou, Jinming Yu, Chunhua Du, Hongqing Zhao, Yu Shang, Linian Huang, Jianping Zhao, Yang Jin, Charles A. Powell, Yuanlin Song, Chunxue Bai. medRxiv 2020.08.07.20163402; doi:<https://doi.org/10.1101/2020.08.07.20163402>

16.   A longitudinal study of the impact of human mobility on the incidence of COVID-19 in India. Sarbeswar Praharaj, Hoon Han. medRxiv 2020.12.21.20248523; doi:<https://doi.org/10.1101/2020.12.21.20248523>

17.   A machine learning aided global diagnostic and comparative tool to assess effect of quarantine control in Covid-19 spread. Raj Dandekar, Chris Rackauckas, George Barbastathis. medRxiv 2020.07.23.20160697; doi:<https://doi.org/10.1101/2020.07.23.20160697>

18.   A Machine Learning Solution Framework for Combatting COVID-19 in Smart Cities from Multiple Dimensions. Ibrahim Abaker Targio Hashem, Absalom E Ezugwu, Mohammed A. Al-Garadi, Idris N. Abdullahi, Olumuyiwa Otegbeye, Queeneth O Ahman, Godwin C. E. Mbah, Amit K Shukla, Haruna Chiroma. medRxiv 2020.05.18.20105577; doi:<https://doi.org/10.1101/2020.05.18.20105577>

19.   A mathematical model to investigate the transmission of COVID-19 in the Kingdom of Saudi Arabia. Fehaid Salem Alshammari. medRxiv 2020.05.02.20088617; doi:<https://doi.org/10.1101/2020.05.02.20088617>

20.   A modified SEIR Model with Confinement and Lockdown of COVID-19 for Costa Rica. Tomas de-Camino-Beck. medRxiv 2020.05.19.20106492; doi: https://doi.org/10.1101/2020.05.19.20106492

21.   A Multi-Task Pipeline with Specialized Streams for Classification and Segmentation of Infection Manifestations in COVID-19 Scans. Shimaa El-bana, Ahmad Al-Kabbany, Maha Sharkas. medRxiv 2020.06.24.20139238; doi:<https://doi.org/10.1101/2020.06.24.20139238>

22.   A multivariate spatiotemporal spread model of COVID-19 using ensemble of ConvLSTM networks. Swarna kamal Paul, Saikat Jana, Parama Bhaumik. medRxiv 2020.04.17.20069898; doi:<https://doi.org/10.1101/2020.04.17.20069898>

23.   A new estimation method for COVID-19 time-varying reproduction number using active cases. Agus Hasan, Hadi Susanto, Venansius Tjahjono, Rudy Kusdiantara, Endah Putri, Panji Hadisoemarto, Nuning Nuraini. medRxiv 2020.06.28.20142158; doi:<https://doi.org/10.1101/2020.06.28.20142158>

24.   A New Mathematical Approach for the Estimation of epidemic Model Parameters with Demonstration on COVID-19 Pandemic in Libya. Mohamed E Saleh, Zeinab Elmehdi Saleh. medRxiv 2020.07.19.20157115; doi:<https://doi.org/10.1101/2020.07.19.20157115>.

25.   A new, simple method of describing the COVID-19 trajectory and dynamics in any country based on Johnson Cumulative Distribution Function fitting. Adam M. Ćmiel, Bogdan Ćmiel. medRxiv 2020.12.05.20244178; doi: https://doi.org/10.1101/2020.12.05.20244178

26.   A New, Simple Projection Model for COVID-19 Pandemic. Jian Lu. medRxiv 2020.03.21.20039867; doi: https://doi.org/10.1101/2020.03.21.20039867

27.   A Novel Approach for Estimating the Final Outcome of Global Diseases Like COVID-19. Demetris T Christopoulos. medRxiv 2020.07.03.20145672; doi: https://doi.org/10.1101/2020.07.03.20145672

28.   A novel comprehensive metric to assess COVID-19 testing outcomes: Effects of geography, government, and policy response. Anthony C Kuster, Hans J Overgaard. medRxiv 2020.06.17.20133389; doi:<https://doi.org/10.1101/2020.06.17.20133389>

29.   A novel deterministic forecast model for the Covid-19 epidemic based on a single ordinary integro-differential equation. Felix Koehler-Rieper, Claudius H. F. Roehl, Enrico De Micheli. Medrxiv 2020.04.29.20084376; doi:<https://doi.org/10.1101/2020.04.29.20084376>

30.   A novel epidemiological model for COVID-19. Mauro Gaspari. medRxiv 2020.07.23.20160580; doi:<https://doi.org/10.1101/2020.07.23.20160580>

31.   A Novel Heuristic Global Algorithm to Predict the COVID-19 Pandemic Trend. Panagiotis G. Asteris, Maria Douvika, Christina Karamani, Athanasia Skentou, Tryfon Daras, Liborio Cavaleri, Danial Jahed Armaghani, Katerina Chlichlia, Theoklis E. Zaoutis. medRxiv 2020.04.16.20068445; doi:<https://doi.org/10.1101/2020.04.16.20068445>

32.   A Novel Method for the Estimation of a Dynamic Effective Reproduction Number (Dynamic-R) in the CoViD-19 Outbreak. Yi Chen Chong. medRxiv 2020.02.22.20023267; doi:<https://doi.org/10.1101/2020.02.22.20023267>

33.   A novel predictive mathematical model for COVID-19 pandemic with quarantine, contagion dynamics, and environmentally mediated transmission. Diego Carvalho, Rafael Barbastefano, Dayse Pastore, Maria Clara Lippi. medRxiv 2020.07.27.20163063; doi:<https://doi.org/10.1101/2020.07.27.20163063>

34.   A pandemic at the Tunisian scale. Mathematical modelling of reported and unreported COVID-19 infected cases. Ines Abdeljaoued-Tej. medRxiv 2020.05.21.20108621; doi:<https://doi.org/10.1101/2020.05.21.20108621>

35.   A parsimonious description and cross-country analysis of COVID-19 epidemic curve. [Kristoffer Rypdal](https://arxiv.org/search/?searchtype=author&query=Rypdal%2C+K), [Martin Rypdal](https://arxiv.org/search/?searchtype=author&query=Rypdal%2C+M). [arXiv:2008.02475](https://arxiv.org/abs/2008.02475)

36.  [A phenomenological algorithm for short-range predictions of the Covid-19 pandemics 2020](https://discover.hsls.pitt.edu/vivisimo/cgi-bin/query-meta?v%3afile=viv_vnkHDb&v%3astate=root%7croot-50-25%7c0&url=https%3a%2f%2fdoi.org%2f10.1101%2f2020.05.22.20098350&rid=Ndoc59&v%3aframe=redirect&v%3aredirect-hash=e1f152b80f92fd848b2214d7289d7f52&). Chrusciel PT, Szybka SJ. medRxiv 2020-05-26. Doi:<https://doi.org/10.1101/2020.05.22.20098350>

37.   A pitfall in estimating the effective reproductive number Rt for COVID-19. daniel wyler, markus petermann. medRxiv 2020.05.12.20099366; doi:<https://doi.org/10.1101/2020.05.12.20099366>

38.   A pragmatic model to forecast the COVID-19 epidemic in different countries and allowing for daily updates. Carlos Nordt, Marcus Herdener. medRxiv 2020.04.07.20056481; doi:<https://doi.org/10.1101/2020.04.07.20056481>

39.   A Predictive Model for the Evolution of COVID-19. Rajneesh Bhardwaj. medRxiv 2020.04.13.20063271; doi:<https://doi.org/10.1101/2020.04.13.20063271>

40.   A prototype for decision support tool to help decision-makers with the strategy of handling the COVID-19 UK epidemic. Anatoly Zhigljavsky, Ivan Fesenko, Henry Wynn, Roger Whitaker, Kobi Kremnizer, Jack Noonan, Jonathan Gillard. medRxiv 2020.04.24.20077818; doi:<https://doi.org/10.1101/2020.04.24.20077818>

41.   A Real-Time Statistical Model for Tracking and Forecasting COVID-19 Deaths, Prevalence and Incidence. Jack A. Syage. medRxiv 2020.05.16.20104430; doi:<https://doi.org/10.1101/2020.05.16.20104430>

42.   A retrospective analysis of the dynamic transmission routes of the COVID-19 in mainland China. Xiandeng Jiang, Le Chang, Yanlin Shi. medRxiv 2020.03.01.20029645; doi:<https://doi.org/10.1101/2020.03.01.20029645>

43.   A simple mathematical model for Coronavirus (COVID-19). Said Melliani, Abdelati El Allaoui, Lalla Saadia Chadli. medRxiv 2020.04.23.20076919; doi:<https://doi.org/10.1101/2020.04.23.20076919>

44.   A Simple Method of Finding an Approximate Pattern of the COVID-19 Spread. Hemanta Kumar Baruah. medRxiv 2020.05.24.20112292; doi:<https://doi.org/10.1101/2020.05.24.20112292>

45.   A simple model to fit the time evolution of the daily death rate of Covid-19 in European Union countries. Tristan Beau, Julien Browaeys, Olivier Dadoun. medRxiv 2020.05.06.20093062; doi:<https://doi.org/10.1101/2020.05.06.20093062>

46.   A Surprising formula for the spread of Covid-19 Under Aggressive Management. Ivan Cherednik. medRxiv 2020.04.29.20084483; doi:<https://doi.org/10.1101/2020.04.29.20084483>

47.   A Two-Region SEIR COVID-19 Epidemic Model for the Island of Ireland. James J. Grannell, James R. Grannell. medRxiv 2020.10.31.20223727; doi:<https://doi.org/10.1101/2020.10.31.20223727>

48.   A two-wave epidemiological model of COVID-19 outbreaks using MS-Excel® Agenor De Noni Junior, Bernardo Araldi da Silva, Felipe Dal-Pizzol, Luismar Marques Porto. medRxiv 2020.05.08.20095133; doi:<https://doi.org/10.1101/2020.05.08.20095133>

49.   Accounting for super-spreading gives the basic reproduction number R0 of COVID-19 that is higher than initially estimated. Marek Kochanczyk, Frederic Grabowski, Tomasz Lipniacki. medRxiv 2020.04.26.20080788; doi:<https://doi.org/10.1101/2020.04.26.20080788>

50.   Adaptive short term COVID-19 prediction for India. Shuvrangshu Jana, Debasish Ghose. medRxiv 2020.07.18.20156745; doi:<https://doi.org/10.1101/2020.07.18.20156745>

51.   Adjusted Dynamics of COVID-19 Pandemic due to Herd Immunity in Bangladesh. Enamul Hoque, Md. Shariful Islam, Mohammad Ruhul Amin, Susanta Kumar Das, Dipak Kumar Mitra. medRxiv 2020.09.03.20186957; doi:<https://doi.org/10.1101/2020.09.03.20186957>

52.   Adjusted fatality rates of COVID19 pandemic: a comparison across countries. Carlos Canelo-Aybar, Jessica Beltran, Marilina Santero, Pablo Alonso-Coello. medRxiv 2020.05.13.20099796; doi:<https://doi.org/10.1101/2020.05.13.20099796>

53.   Adjusting COVID-19 Reports for Countries Age Disparities: A Comparative Framework for Reporting Performances. Enes Eryarsoy, Dursun Delen, Behrooz Davazdahemami. medRxiv 2020.08.31.20185223; doi: https://doi.org/10.1101/2020.08.31.20185223

54.   Agent-Based Simulation for Evaluation of Contact-Tracing Policies Against the Spread of SARS-CoV-2. Martin Richard Bicher, Claire Rippinger, Christoph Urach, Dominik Brunmeir, Uwe Siebert, Niki Popper. medRxiv 2020.05.12.20098970; doi:<https://doi.org/10.1101/2020.05.12.20098970>

55.   Age-targeted dose allocation can halve COVID-19 vaccine requirements. Michael T. Meehan, Daniel G. Cocks, Jamie M. Caldwell, James M. Trauer, Adeshina I. Adekunle, Romain R. Ragonnet, Emma S. McBryde. medRxiv 2020.10.08.20208108; doi:<https://doi.org/10.1101/2020.10.08.20208108>

56.   An attempt to optimize human resources allocation based on spatial diversity of COVID-19 cases in Poland. Andrzej Jarynowski, Monika Wójta-Kempa, Łukasz Krzowski. medRxiv 2020.10.14.20090985; doi: https://doi.org/10.1101/2020.10.14.20090985

57.   An improved method to estimate the effective reproduction number of the COVID-19 pandemic: lessons from its application in Greece. Theodore Lytras, Vana Sypsa, Demosthenes Panagiotakos, Sotirios Tsiodras. medRxiv 2020.09.19.20198028; doi:<https://doi.org/10.1101/2020.09.19.20198028>

58.   An improved methodology for estimating the prevalence of SARS-CoV-2. Virag Patel, Catherine McCarthy, Rachel A Taylor, Ruth Moir, Louise A Kelly, Emma L Snary. medRxiv 2020.08.04.20168187; doi:<https://doi.org/10.1101/2020.08.04.20168187>

59.   An integrated deterministic-stochastic approach for predicting the long-term trajectories of COVID-19. Indrajit Ghosh, Tanujit Chakraborty. medRxiv 2020.05.13.20101303; doi:<https://doi.org/10.1101/2020.05.13.20101303>

60.   An SEIR Model for Assessment of Current COVID-19 Pandemic Situation in the UK. Peiliang Sun, Kang Li. medRxiv 2020.04.12.20062588; doi:<https://doi.org/10.1101/2020.04.12.20062588>

61.   An SEIR Model with Contact Tracing and Age-Structured Social Mixing for COVID-19 outbreak. Ali Teimouri. medRxiv 2020.07.05.20146647; doi:<https://doi.org/10.1101/2020.07.05.20146647>

62.   Analyses and Forecast for COVID-19 epidemic in India. Rudra Banerjee, Srijit Bhattacharjee, Pritish Kumar Varadwaj. medRxiv 2020.06.26.20141077; doi:<https://doi.org/10.1101/2020.06.26.20141077>

63.   Analysis and prediction of Covid-19 spreading through Bayesian modelling with a case study of Uttar Pradesh, India. Deepmala, Nishant Kumar Srivastava, Vineet Kumar, Sanjay Kumar Singh. medRxiv 2020.08.25.20180265; doi:<https://doi.org/10.1101/2020.08.25.20180265>

64.   Analysis of Covid-19 Data for Eight European Countries and the United Kingdom Using a Simplified SIR Model. Gyan Bhanot, Charles DeLisi. medRxiv 2020.05.26.20114058; doi: https://doi.org/10.1101/2020.05.26.20114058

65.   Analysis of geo-temporal evolution and modeling of the COVID-19 epidemic in Libya. Amin Bredan, Hani Benamer, Omran Bakoush- medRxiv 2020.09.19.20197822; doi: https://doi.org/10.1101/2020.09.19.20197822

66.   Analysis of the COVID-19 pandemic in Bavaria: adjusting for misclassification. Felix Guenther, Andreas Bender, Michael Höhle, Manfred Wildner, Helmut Küchenhoff. medRxiv 2020.09.29.20203877; doi:<https://doi.org/10.1101/2020.09.29.20203877>

67.   Analysis of the early Covid-19 epidemic curve in Germany by regression models with change points. Helmut Küchenhoff, Felix Günther, Michael Höhle, Andreas Bender. medRxiv 2020.10.29.20222265; doi:<https://doi.org/10.1101/2020.10.29.20222265>

68.   Analysis of the mitigation strategies for COVID-19: from mathematical modelling perspective. S. M. Kassa, H.J.B. Njagarah, Y. A. Terefe. medRxiv 2020.04.15.20066308; doi:<https://doi.org/10.1101/2020.04.15.20066308>

69.  [Analysis of Covid-19 Data for Eight European Countries and the United Kingdom Using a Simplified SIR Model](https://discover.hsls.pitt.edu/vivisimo/cgi-bin/query-meta?v%3afile=viv_vnkHDb&v%3astate=root%7croot&url=https%3a%2f%2fdoi.org%2f10.21203%2frs.3.rs-97697%2fv1&rid=Ndoc14&v%3aframe=redirect&v%3aredirect-hash=1b0a557254ae254f8806cc0bbbfc6b37&) [new window](https://discover.hsls.pitt.edu/vivisimo/cgi-bin/query-meta?v%3afile=viv_vnkHDb&v%3astate=root%7croot&url=https%3a%2f%2fdoi.org%2f10.21203%2frs.3.rs-97697%2fv1&rid=Ndoc14&v%3aframe=redirect&v%3aredirect-hash=1b0a557254ae254f8806cc0bbbfc6b37&). Bhanot G, DeLisi C. Research Square. Doi:<https://doi.org/10.21203/rs.3.rs-97697/v1>

70.   Analytical solution of equivalent SEIR and agent-based model of COVID-19; showing the bounds of contact tracing. Huseyin Tunc, Fatma Zehra Sari, Busra Nur Darendeli, Ramin Nashebi, Murat Sari, Seyfullah Kotil. medRxiv 2020.10.20.20212522; doi:<https://doi.org/10.1101/2020.10.20.20212522>

71.   Analyzing Covid-19 Data using SIRD Models. Abhijit Chakraborty, Jiaying Chen, Amelie Desvars-Larrive, Peter Klimek, Erwin Flores Tames, David Garcia, Leonhard Horstmeyer, Michaela Kaleta, Jana Lasser, Jenny Reddish, Beate Pinior, Johannes Wachs, Peter Turchin. medRxiv 2020.05.28.20115527; doi:<https://doi.org/10.1101/2020.05.28.20115527>

72.   Anosmia and other SARS-CoV-2 positive test-associated symptoms, across three national, digital surveillance platforms as the COVID-19 pandemic and response unfolded: an observation study. Carole H. Sudre, Ayya Keshet, Mark S. Graham, Amit D. Joshi, Smadar Shilo, Hagai Rossman, Benjamin Murray, Erika Molteni, Kerstin Klaser, Liane D Canas, Michela Antonelli, Marc Modat, Joan Capdevila Pujol, Sajaysurya Ganesh, Jonathan Wolf, Tomer Meir, Andrew T. Chan, Claire J. Steves, Tim D. Spector, John S. Brownstein, Eran Segal, Sebastien Ourselin, Christina M. Astley. medRxiv 2020.12.15.20248096; doi:<https://doi.org/10.1101/2020.12.15.20248096>

73.   Assessing the effect of global travel and contact reductions to mitigate the COVID-19 pandemic and resurgence. Shengjie Lai, Nick W Ruktanonchai, Alessandra Carioli, Corrine Ruktanonchai, Jessica Floyd, Olivia Prosper, Chi Zhang, Xiangjun Du, Weizhong Yang, Andrew J Tatem. medRxiv 2020.06.17.20133843; doi:<https://doi.org/10.1101/2020.06.17.20133843>

74.   Assessing the risk of spread of COVID-19 to the Asia Pacific region. Freya M. Shearer, James Walker, Nefel Tellioglu, James M. McCaw, Jodie McVernon, Andrew Black, Nic Geard. medRxiv 2020.04.09.20057257; doi:<https://doi.org/10.1101/2020.04.09.20057257>

75.   Assessing the Tendency of 2019-nCoV (COVID-19) Outbreak in China. Qinghe Liu, Zhicheng Liu, Deqiang Li, Zefei Gao, Junkai Zhu, Junyan Yang, Qiao Wang. medRxiv 2020.02.09.20021444; doi:<https://doi.org/10.1101/2020.02.09.20021444>

76.   Association between epidemic dynamics of Covid-19 infection and ABO blood group types. Yuefei Liu, Juergen M. Steinacker, Lisa Haeussinger, Alexander Dinse-Lambracht. medRxiv 2020.07.12.20152074; doi:<https://doi.org/10.1101/2020.07.12.20152074>

77.   Autocatalytic Model for Covid-19 Progression in a Country. Anatoly Chernyshev. medRxiv 2020.04.03. 20052985; https://doi.org/10.1101/2020.04.03.20052985

78.   Automated Contact Tracing: a game of big numbers in the time of COVID-19. Hyunju Kim, Ayan Paul. medRxiv 2020.04.22.20071043; doi:<https://doi.org/10.1101/2020.04.22.20071043>

79.   Base Reproduction Number of COVID-19: Statistic Analysis. Hongjun Zhu, JIANGPING HUANG, XIN LIU. medRxiv 2020.09.26.20202010; doi:<https://doi.org/10.1101/2020.09.26.20202010>

80.   Baseline phenotype and 30-day outcomes of people tested for COVID-19: an international network cohort including >3.32 million people tested with real-time PCR and >219,000 tested positive for SARS-CoV-2 in South Korea, Spain and the United States. Asieh Golozar, Lana YH Lai, Anthony G. Sena, David Vizcaya, Lisa M. Schilling, Vojtech Huser, Fredrik Nyberg, Scott L. Duvall, Daniel R. Morales, Thamir M Alshammari, Hamed Abedtash, Waheed-Ul-Rahman Ahmed, Osaid Alser, Heba Alghoul, Ying Zhang, Mengchun Gong, Yin Guan, Carlos Areia, Jitendra Jonnagaddala, Karishma Shah, Jennifer C.E. Lane, Albert Prats-Uribe, Jose D. Posada, Nigam H. Shah, Vignesh Subbian, Lin Zhang, Maria Tereza Fernandes Abrahão, Peter R. Rijnbeek, Seng Chan You, Paula Casajust, Elena Roel, Martina Recalde, Sergio Fernández-Bertolín, Alan Andryc, Jason A. Thomas, Adam B. Wilcox, Stephen Fortin, Clair Blacketer, Frank DeFalco, Karthik Natarajan, Thomas Falconer, Matthew Spotnitz, Anna Ostropolets, George Hripcsak, Marc Suchard, Kristine E. Lynch, Michael E. Matheny, Andrew Williams, Christian Reich, Talita Duarte-Salles, Kristin Kostka, Patrick B. Ryan, Daniel Prieto-Alhambra. medRxiv 2020.10.25.20218875; doi:<https://doi.org/10.1101/2020.10.25.20218875>

81.   Bayesian approach for modelling the dynamic of COVID-19 outbreak on the Diamond Princess Cruise Ship. Chao-Chih Lai, Chen-Yang Hsu, Hsiao-Hsuan Jen, Ming-Fang Yen, Chang-Chuan Chan, Hsiu-Hsi Chen. medRxiv 2020.06.21.20136465; doi:<https://doi.org/10.1101/2020.06.21.20136465>

82.   Bayesian back-calculation and nowcasting for line list data during the COVID-19 pandemic. Tenglong Li, Laura F. White. medRxiv 2020.12.08.20238154; doi:<https://doi.org/10.1101/2020.12.08.20238154>.

83.   Bayesian nowcasting with adjustment for delayed and incomplete reporting to estimate COVID-19 infections in the United States. Melanie H Chitwood, Marcus Russi, Kenneth Gunasekera, Joshua Havumaki, Virginia E. Pitzer, Joshua L Warren, Daniel Weinberger, Ted Cohen, Nicolas A Menzies. medRxiv 2020.06.17.20133983; doi:<https://doi.org/10.1101/2020.06.17.20133983>

84.   BCG vaccination in infancy does not protect against COVID-19. Evidence from a natural experiment in Sweden. [Clément de Chaisemartin](https://arxiv.org/search/?searchtype=author&query=de+Chaisemartin%2C+C), [Luc de Chaisemartin](https://arxiv.org/search/?searchtype=author&query=de+Chaisemartin%2C+L). [arXiv:2006.05504](https://arxiv.org/abs/2006.05504)

85.   Behavioral changes before lockdown, and decreased retail and recreation mobility during lockdown, contributed most to the successful control of the COVID-19 epidemic in 35 Western countries. Koen Deforche, Jurgen Vercauteren, Viktor Müller, Anne-Mieke Vandamme. medRxiv 2020.06.20.20136382; doi:<https://doi.org/10.1101/2020.06.20.20136382>

86.   Behavioral dynamics of COVID-19: estimating under-reporting, multiple waves, and adherence fatigue across 91 nations. Hazhir Rahmandad, Tse Yang Lim, John Sterman. medRxiv 2020.06.24.20139451; doi:<https://doi.org/10.1101/2020.06.24.20139451>

87.   Better Strategies for Containing COVID-19 Epidemics–A Study of 25 Countries via an Extended SEIR Model. Jia Gu, Han Yan, Yaxuan Huang, Yuru Zhu, Haoxuan Sun, Xinyu Zhang, Yuqing Wang, Yumou Qiu, Song Xi Chen. medRxiv 2020.04.27.20081232; doi:<https://doi.org/10.1101/2020.04.27.20081232>

88.   Beware That COVID-19 Would Be Worse in Winter: A Study of a Global Panel of 1236 Regions. Chen Zhang, Hua Liao, Eric Strol, Hui Li, Ru Li, Steen Solvang Jensen, Ying Zhang. medRxiv 2020.07.29.20164152; doi:<https://doi.org/10.1101/2020.07.29.20164152>

89.   Bibliometric Analysis of Global Scientific Research on SARS-CoV-2 (COVID-19). Fatemeh Rafiei Nasab, Fakher Rahim. medRxiv 2020.03.19.20038752; doi:<https://doi.org/10.1101/2020.03.19.20038752>

90.   Brazilian Modeling of COVID-19(BRAM-COD): a Bayesian Monte Carlo approach for COVID-19 spread in a limited data set context. Samy Dana, Alexandre B. Simas, Bruno A. Filardi, Rodrigo N. Rodriguez, Leandro da Costa Lane Valiengo, Jose Gallucci-Neto. medRxiv 2020.04.29.20081174; doi:<https://doi.org/10.1101/2020.04.29.20081174>

91.  [Building Better Retirement Systems in the Wake of the Global Pandemic](https://discover.hsls.pitt.edu/vivisimo/cgi-bin/query-meta?v%3afile=viv_vnkHDb&v%3astate=root%7croot&url=https%3a%2f%2fdoi.org%2f10.2139%2fssrn.3731009&rid=Ndoc9&v%3aframe=redirect&v%3aredirect-hash=5e609b86a61a582c46389db8ce644f00&) [new window](https://discover.hsls.pitt.edu/vivisimo/cgi-bin/query-meta?v%3afile=viv_vnkHDb&v%3astate=root%7croot&url=https%3a%2f%2fdoi.org%2f10.2139%2fssrn.3731009&rid=Ndoc9&v%3aframe=redirect&v%3aredirect-hash=5e609b86a61a582c46389db8ce644f00&). Mitchell OS. SSRN. Doi:<https://doi.org/10.2139/ssrn.3731009>

92.   Causal Modeling of Twitter Activity During COVID-19. Oguzhan Gencoglu, Mathias Gruber. medRxiv 2020.05.16.20103903; doi:<https://doi.org/10.1101/2020.05.16.20103903>

93.   Challenges in control of Covid-19: short doubling time and long delay to effect of interventions. Lorenzo Pellis, Francesca Scarabel, Helena B Stage, Christopher E Overton, Lauren H K Chappell, Katrina A Lythgoe, Elizabeth Fearon, Emma Bennett, Jacob Curran-Sebastian, Rajenki Das, Martyn Fyles, Hugo Lewkowicz, Xiaoxi Pang, Bindu Vekaria, Luke Webb, Thomas A House, Ian Hall. medRxiv 2020.04.12.20059972; doi:<https://doi.org/10.1101/2020.04.12.20059972>

94.   Change points in the spread of COVID-19 question the effectiveness of nonpharmaceutical interventions in Germany. Thomas Wieland. medRxiv 2020.0. 7.05.20146837; doi:<https://doi.org/10.1101/2020.07.05.20146837>

95.   Changing transmission dynamics of COVID-19 in China: a nationwide population-based piecewise mathematical modelling study. Jiawen Hou, Jie Hong, Boyun Ji, Bowen Dong, Yue Chen, Michael P. Ward, Wei Tu, Zhen Jin, Jian Hu, Qing Su, Wenge Wang, Zheng Zhao, Shuang Xiao, Jiaqi Huang, Wei Lin, Zhijie Zhang. medRxiv 2020.03.27.20045757; doi:<https://doi.org/10.1101/2020.03.27.20045757>

96.   Chaos theory applied to the outbreak of Covid-19: an ancillary approach to decision-making in pandemic context. S. Mangiarotti, M. Peyre, Y. Zhang, M. Huc, F. Roger, Y. Kerr. medRxiv 2020.04.02.20051441; doi:<https://doi.org/10.1101/2020.04.02.20051441>

97.   China’s effective control and other countries’ uncharted challenge against COVID-19: an epidemiological and modelling study. Lingling Zheng, Qin Kang, Weiyao Liao, Xiujuan Chen, Shuai Huang, Dong Liu, Huimin Xia, Jinling Tang, Huiying Liang. medRxiv 2020.04.28.20083899; doi:<https://doi.org/10.1101/2020.04.28.20083899>

98.   Clarifying predictions for COVID-19 from testing data: the example of New-York State. Quentin Griette, Pierre Magal. medRxiv 2020.10.10.20203034; doi:<https://doi.org/10.1101/2020.10.10.20203034>

99.   Clinical characteristics of COVID-19 infection in pregnant women: a systematic review and meta-analysis. Sina Arabi, Golnaz Vaseghi, Zahra Heidari, Laleh Shariati, Bahareh Amin, Harunor Rashid, Shaghayegh Haghjooy Javanmard. medRxiv 2020.04.05.20053983; doi:<https://doi.org/10.1101/2020.04.05.20053983>

100. Comparative Analysis of Early Dynamic Trends in Novel Coronavirus Outbreak: A Modeling Framework. Huazhen Lin, Wei Liu, Hong Gao, Jinyu Nie, Qiao Fan. medRxiv 2020.02.21.20026468; doi:<https://doi.org/10.1101/2020.02.21.20026468>

101. Comparing the impact on COVID-19 mortality of self-imposed behavior change and of government regulations across 13 countries. Julian Jamison, Donald Bundy, Dean Jamison, Jacob Spitz, Stephane Verguet. medRxiv 2020.08.02.20166793; doi:<https://doi.org/10.1101/2020.08.02.20166793>

102. Comparison of COVID-19 outcomes among shielded and non-shielded populations: A general population cohort study of 1.3 million. Bhautesh D Jani, Frederick K Ho, David J Lowe, Jamie P Traynor, Sean MacBride-Stewart, Patrick B Mark, Frances S Mair, Jill P Pell. medRxiv 2020.09.17.20196436; doi: https://doi.org/10.1101/2020.09.17.20196436

103. Comparison of Healthcare costs and benefits of the UK’s Covid-19 response with four European countries: Decision Modelling Study. Howard Thom, Josephine Walker, Peter Vickerman, Will Hollingworth. medRxiv 2020.12.14.20248201; doi:<https://doi.org/10.1101/2020.12.14.20248201>

104. Complexity signatures in the COVID-19 epidemic: power law behaviour in the saturation regime of fatality curves. Giovani L. Vasconcelos, Antônio M.S. Macêdo, Gerson C. Duarte-Filho, Arthur A. Araújo, Raydonal Ospina, Francisco A. G. Almeida. medRxiv 2020.07.12.20152140; doi: https://doi.org/10.1101/2020.07.12.20152140

105. Confirmed central olfactory system lesions on brain MRI in COVID-19 patients with anosmia: a case-series. Yannick Girardeau, Yoan GALLOIS, Guillaume DE BONNECAZE, Bernard ESCUDE, Clarisse LAFONT, Gilles CHATELLIER, Mathieu MARX. medRxiv 2020.07.08.20148692; doi:<https://doi.org/10.1101/2020.07.08.20148692>

106. Confronting COVID-19: Surging critical care capacity in Italy. Jose M Rodriguez-Llanes, Rafael Castro Delgado, Morten Gram Pedersen, Pedro Arcos González, Matteo Meneghini. medRxiv 2020.04.01.20050237; doi:<https://doi.org/10.1101/2020.04.01.20050237>

107. [Constitutions and Contagion. European Constitutional Systems and the COVID-19 Pandemic](https://discover.hsls.pitt.edu/vivisimo/cgi-bin/query-meta?v%3afile=viv_vnkHDb&v%3astate=root%7croot&url=https%3a%2f%2fdoi.org%2f10.2139%2fssrn.3727240&rid=Ndoc10&v%3aframe=redirect&v%3aredirect-hash=7728c690244b6cdfd87c5a69ceb0c196&) [new window](https://discover.hsls.pitt.edu/vivisimo/cgi-bin/query-meta?v%3afile=viv_vnkHDb&v%3astate=root%7croot&url=https%3a%2f%2fdoi.org%2f10.2139%2fssrn.3727240&rid=Ndoc10&v%3aframe=redirect&v%3aredirect-hash=7728c690244b6cdfd87c5a69ceb0c196&). Golia A, Hering L, Moser C, Sparks T. SSRN. Doi:<https://doi.org/10.2139/ssrn.3727240>

108. Contact tracing efficiency, transmission heterogeneity, and accelerating COVID-19 epidemics. Billy J Gardner, A. Marm Kilpatrick. medRxiv 2020.09.04.20188631; doi:<https://doi.org/10.1101/2020.09.04.20188631>

109. Containing Covid-19 outbreaks with spatially targeted short-term lockdowns and mass-testing. Justin Alsing, Naïri Usher, Philip JD Crowley. medRxiv 2020.05.05.20092221; doi:<https://doi.org/10.1101/2020.05.05.20092221>

110. Control Strategies to Curtail Transmission of COVID-19. Nita H. Shah, Ankush H. Suthar, Ekta N. Jayswal. medRxiv 2020.04.04.20053173; doi:<https://doi.org/10.1101/2020.04.04.20053173>.

111. Controlled Avalanche – A Regulated Voluntary Exposure Approach for Addressing Covid-19. Eyal Klement, Alon Klement, David Chinitz, Alon Harel, Eyal Fattal, Ziv Klausner. medRxiv 2020.04.12.20062687; doi:<https://doi.org/10.1101/2020.04.12.20062687>

112. Controlling the Spread of COVID-19: Optimal Control Analysis. Chinwendu Emilian Madubueze, Dachollom Sambo, Isaac O. Onwubuya. medRxiv 2020.06.08.20125393; doi:<https://doi.org/10.1101/2020.06.08.20125393>

113. Cooperative virus propagation in COVID-19 transmission Ziwei Dai, Jason W Locasale. medRxiv 2020.05.05.20092361; doi:<https://doi.org/10.1101/2020.05.05.20092361>

114. Correlating Covid-19 mortality and infection levels. Mugdha Gadgil, Chetan Gadgil. medRxiv 2020.05.01.20087320; doi:<https://doi.org/10.1101/2020.05.01.20087320>

115. Correlation between daily infections and fatality rate due to Covid-19 in Germany. Dieter Mergel. medRxiv 2020.08.03.20167304; doi:<https://doi.org/10.1101/2020.08.03.20167304>

116. Countries are Clustered but Number of Tests is not Vital to Predict Global COVID-19 Confirmed Cases: A Machine Learning Approach. Md Hasinur Rahaman Khan, Ahmed Hossain. medRxiv 2020.04.24.20078238; doi:<https://doi.org/10.1101/2020.04.24.20078238>

117. COVID 19 healthcare facility demand forecasts for rural residents. Andrio Adwibowo. medRxiv 2020.06.05. 20123380; doi:<https://doi.org/10.1101/2020.06.05.20123380>

118. COVID-19 :Determinants of Hospitalization, ICU and Death among 20,293 reported cases in Portugal. Vasco Ricoca Peixoto, Andre Vieira, Pedro Aguiar, Paulo Sousa, Carlos Carvalho, Daniel Rhys Thomas, Alexandre Abrantes, Carla Nunes. medRxiv 2020.05.29.20115824; doi:<https://doi.org/10.1101/2020.05.29.20115824>

119. COVID-19 Asymptomatic Infection Estimation. Yang Yu, Yu-Ren Liu, Fan-Ming Luo, Wei-Wei Tu, De-Chuan Zhan, Guo Yu, Zhi-Hua Zhou. medRxiv 2020.04.19.20068072; doi:<https://doi.org/10.1101/2020.04.19.20068072>

120. COVID-19 case forecasting model for Sri Lanka based on Stringency Index. Achala U. Jayatilleke, Sanjeewa Dayarathne, Padmal de Silva, Pandula Siribaddana, Rushan A.B. Abeygunawardana, Olivia Nieveras, Nilanthi de Silva, Janaka de Silva. medRxiv 2020.05.20.20103887; doi: https://doi.org/10.1101/2020.05.20.20103887

121. COVID-19 case-fatality rate and demographic and socioeconomic influencers: a worldwide spatial regression analysis based on country-level data. Yang Cao, Ayako Hiyoshi, Scott Montgomery. medRxiv 2020.07.31.20165811; doi:<https://doi.org/10.1101/2020.07.31.20165811>

122. COVID-19 data analysis and modeling in Palestine. Ines Abdeljaoued-Tej. medRxiv 2020.04.24.20078279; doi:<https://doi.org/10.1101/2020.04.24.20078279>

123. COVID-19 death rates by age and sex and the resulting mortality vulnerability of countries and regions in the world. Christophe Z Guilmoto. medRxiv 2020.05.17.20097410; doi:<https://doi.org/10.1101/2020.05.17.20097410>

124. COVID–19 Disease Dynamics in Germany: First Models and Parameter Identification. Thomas Götz, Peter Heidrich. medRxiv 2020.04.23.20076992; doi:<https://doi.org/10.1101/2020.04.23.20076992>

125. Covid-19 dynamics considering the influence of hospital infrastructure: an investigation of brazilian scenarios. Pedro M.C.L. Pacheco, Marcelo A. Savi, Pedro V. Savi. medRxiv 2020.06.03.20121608; doi:<https://doi.org/10.1101/2020.06.03.20121608>

126. COVID-19 effective reproductive ratio determination: An application, and analysis of issues and influential factors. Luis Alfredo Bautista Balbás, Mario Gil Conesa, Gil Rodríguez Caravaca, Blanca Bautista Balbás. medRxiv 2020.07.15.20154039; doi: https://doi.org/10.1101/2020.07.15.20154039

127. COVID-19 epidemic in Sri Lanka: A mathematical and computational modelling approach to control. WPTM Wickramaarachchi, SSN Perera, S Jayasignhe. medRxiv 2020.04.21.20073734; doi:<https://doi.org/10.1101/2020.04.21.20073734>

128. COVID-19 Epidemic Outside China: 34 Founders and Exponential Growth. Yi Li, Meng Liang, Xianhong Yin, Xiaoyu Liu, Meng Hao, Zixin Hu, Yi Wang, Li Jin. medRxiv 2020.03.01.20029819; doi:<https://doi.org/10.1101/2020.03.01.20029819>

129. COVID-19 healthcare demand and mortality in Sweden in response to non-pharmaceutical (NPIs) mitigation and suppression scenarios. Henrik Sjödin, Anders F. Johansson, Åke Brännström, Zia Farooq, Hedi Katre Kriit, Annelies Wilder-Smith, Christofer Åström, Johan Thunberg, Mårten Söderquist, Joacim Rocklöv. medRxiv 2020.03.20.20039594; doi:<https://doi.org/10.1101/2020.03.20.20039594>

130. COVID-19 in England: spatial patterns and regional outbreaks. Claudio Fronterre, Jonathan M Read, Barry Rowlingson, Simon Alderton, Jessica Bridgen, Peter J Diggle, Chris P Jewell. medRxiv 2020.05.15.20102715; doi:<https://doi.org/10.1101/2020.05.15.20102715>

131. CoViD-19 in Italy: a mathematical model to analyze the epidemic containment strategy and the economic impacts. Fabio Verachi, Luca G Trussoni, Luciano Lanzi. medRxiv 2020.05.28.20115790; doi:<https://doi.org/10.1101/2020.05.28.20115790>

132. CoVID-19 in Singapore: Impact of Contact Tracing and Self-awareness on Healthcare Demand. Qiuyang Huang, Lin Wang, Yongjian Yang, Liping Huang, Zhanwei Du, Gaoxi Xiao. medRxiv 2020.06.04.20122879; doi:<https://doi.org/10.1101/2020.06.04.20122879>

133. COVID-19 incidence and R decreased on the Isle of Wight after the launch of the Test, Trace, Isolate programme. Michelle Kendall, Luke Milsom, Lucie Abeler-Dorner, Chris Wymant, Luca Ferretti, Mark Briers, Chris Holmes, David Bonsall, Johannes Abeler, Christophe Fraser. medRxiv 2020.07.12.20151753; doi:<https://doi.org/10.1101/2020.07.12.20151753>

134. Covid-19 Incidence Rate Evolution Modeling using Dual Wave Gaussian-Lorentzian Composite Functions. Radhakrishnan Poomari. medRxiv 2020.06.07.20124966; doi:<https://doi.org/10.1101/2020.06.07.20124966>

135. COVID-19 Infection Forecasting based on Deep Learning in Iran. Mehdi Azarafza, Mohammad Azarafza, Jafar Tanha. medRxiv 2020.05.16.20104182; doi:<https://doi.org/10.1101/2020.05.16.20104182>

136. COVID-19 lockdowns cause global air pollution declines with implications for public health risk. Zander S. Venter, Kristin Aunan, Sourangsu Chowdhury, Jos Lelieveld. medRxiv 2020.04.10.20060673; doi:<https://doi.org/10.1101/2020.04.10.20060673>

137. COVID-19 mortality rate in Russia: forecasts and reality evaluation. Marina Lifshits, Natalia Neklyudova. medRxiv 2020.09.25.20201376; doi:<https://doi.org/10.1101/2020.09.25.20201376>

138. COVID-19 outbreak in Algeria: A mathematical Model to predict cumulative cases. Mohamed Hamidouche Sr. medRxiv 2020.03.20.20039891; doi:<https://doi.org/10.1101/2020.03.20.20039891>

139. COVID-19 outbreak in Mauritius: Logistic growth and SEIR modelling with quarantine and an effective reproduction number. Antoine Gehin, Smita Goorah, Khemanand Moheeput, Satish Ramchurn. medRxiv 2020.09.22.20199364; doi:<https://doi.org/10.1101/2020.09.22.20199364>

140. COVID-19 Outbreak Prediction with Machine Learning. Sina F. Ardabili, Amir Mosavi, Pedram Ghamisi, Filip Ferdinand, Annamaria R. Varkonyi-Koczy, Uwe Reuter, Timon Rabczuk, Peter M. Atkinson. medRxiv 2020.04.17.20070094; doi:<https://doi.org/10.1101/2020.04.17.20070094>

141. Covid-19 Outbreak Progression in Italian Regions: Approaching the Peak by March 29^th^. Cosimo Distante, Prisco Piscitelli, Alessandro Miani. medRxiv 2020.03.30.20043612; doi:<https://doi.org/10.1101/2020.03.30.20043612>

142. COVID-19 Outbreak, Social Response, and Early Economic Effects: A Global VAR Analysis of Cross-Country Interdependencies. Fabio Milani. medRxiv 2020.05.07.20094748; doi:<https://doi.org/10.1101/2020.05.07.20094748>

143. COVID-19 pandemic brings a sedentary lifestyle: a cross-sectional and longitudinal study. Chen Zheng, Wendy Yajun Huang, Sinead Sheridan, Cindy Hui-Ping Sit, Xiang-Ke Chen, Stephen Heung-Sang Wong. medRxiv 2020.05.22.20110825; doi:<https://doi.org/10.1101/2020.05.22.20110825>

144. COVID-19 peak estimation and effect of nationwide lockdown in India. R V Belfin, Piotr Bródka, B L Radhakrishnan, V Rejula. medRxiv 2020.05.09.20095919; doi:<https://doi.org/10.1101/2020.05.09.20095919>

145. Covid-19 Prediction in USA using modified SIR derived model. Jathin desan- medRxiv 2020.12.20.20248600; doi:<https://doi.org/10.1101/2020.12.20.20248600>

146. COVID-19 Scenarios: an interactive tool to explore the spread and associated morbidity and mortality of SARS-CoV-2. Nicholas B Noll, Ivan Aksamentov, Valentin Druelle, Abrie Badenhorst, Bruno Ronzani, Gavin Jefferies, Jan Albert, Richard A Neher. medRxiv 2020.05.05.20091363; doi:<https://doi.org/10.1101/2020.05.05.20091363>

147. COVID-19 serial interval estimates based on confirmed cases in public reports from 86 Chinese cities. Zhanwei Du, Xiaoke Xu, Ye Wu, Lin Wang, Benjamin J. Cowling, Lauren Ancel Meyers. medRxiv 2020.04.23.20075796; doi:<https://doi.org/10.1101/2020.04.23.20075796>

148. Covid-19 testing strategies and lockdowns: the European closed curves, analysed by ``skew-normal'' distributions, the forecasts for the UK, Sweden, and the USA, and the ongoing outbreak in Brazil.. Stefano De Leo. medRxiv 2020.06.01.20119461; doi:<https://doi.org/10.1101/2020.06.01.20119461>

149. Covid-19 trajectories: Monitoring pandemic in the worldwide context. Henry Loeffler-Wirth, Maria Schmidt, Hans Binder. medRxiv 2020.06.04.20120725; doi:<https://doi.org/10.1101/2020.06.04.20120725>

150. COVID-19 Transmission Dynamics and Effectiveness of Public Health Interventions in New York City during the 2020 Spring Pandemic Wave. Wan Yang, Jaimie Shaff, Jeffrey Shaman. medRxiv 2020.09.08.20190710; doi:<https://doi.org/10.1101/2020.09.08.20190710>

151. Covid-19 transmission dynamics during the unlock phase and significance of testing. Abhijit Paul, Samrat Chatterjee, Nandadulal Bairagi. medRxiv 2020.08.18.20176354; doi:<https://doi.org/10.1101/2020.08.18.20176354>

152. COVID-19 transmission in Mainland China is associated with temperature and humidity: a time-series analysis. Hongchao Qi, Shuang Xiao, Runye Shi, Michael P. Ward, Yue Chen, Wei Tu, Qing Su, Wenge Wang, Xinyi Wang, Zhijie Zhang. medRxiv 2020.03.30.20044099; doi:<https://doi.org/10.1101/2020.03.30.20044099>

153. COVID-19 trend in Bangladesh: deviation from epidemiological model and critical analysis of the possible factors. Asif Ahmed, Mohammad Mahmudur Rahman. medRxiv 2020.05.31.20118745; doi:<https://doi.org/10.1101/2020.05.31.20118745>

154. COVID-19 Utilization and Resource Visualization Engine (CURVE) to Forecast In-Hospital Resources. Shih-Hsiung Chou, James T Kearns, Philip Turk, Marc A. Kowalkowski, Jason Roberge, Jennifer S. Priem, Yhenneko J. Taylor, Ryan Burns, Pooja Palmer, Andrew D. McWilliams. medRxiv 2020.05.01.20087973; doi:<https://doi.org/10.1101/2020.05.01.20087973>

155. [Covid-19 Incidence Rate Evolution Modeling using Dual Wave Gaussian-Lorentzian Composite Functions](https://discover.hsls.pitt.edu/vivisimo/cgi-bin/query-meta?v%3afile=viv_vnkHDb&v%3astate=root%7croot-25-25%7c0&url=https%3a%2f%2fdoi.org%2f10.1101%2f2020.06.07.20124966&rid=Ndoc47&v%3aframe=redirect&v%3aredirect-hash=1629f21ba8366d0e71ab7c31bdbeade3&). Poomari R. medRxiv 2020-06-09. Doi:<https://doi.org/10.1101/2020.06.07.20124966>

156. [COVID-19 lockdowns cause global air pollution declines with implications for public health risk](https://discover.hsls.pitt.edu/vivisimo/cgi-bin/query-meta?v%3afile=viv_vnkHDb&v%3astate=root%7croot-75-25%7c0&url=https%3a%2f%2fdoi.org%2f10.1101%2f2020.04.10.20060673&rid=Ndoc87&v%3aframe=redirect&v%3aredirect-hash=d1f7017cdbbf7f2cb3882775a0431c34&). Venter ZS, Aunan K, Chowdhury S, Lelieveld J. medRxiv 2020-04-14. Doi:<https://doi.org/10.1101/2020.04.10.20060673>

157. [COVID-19 Pandemic and Stress Testing the Eurozone Credit Portfolios](https://discover.hsls.pitt.edu/vivisimo/cgi-bin/query-meta?v%3afile=viv_vnkHDb&v%3astate=root%7croot&url=https%3a%2f%2fdoi.org%2f10.2139%2fssrn.3705474&rid=Ndoc17&v%3aframe=redirect&v%3aredirect-hash=500aed386984330fb9fe1fb4fd0a32ec&). Yarovaya L, Mirza N, Rizvi SKA, Naqvi B. SSRN. Doi: https://doi.org/10.2139/ssrn.3705474 . Rizvi SKA, Yarovaya L, Mirza N, Naqvi B. SSRN 2020-10-11. Doi: https://doi.org/10.2139/ssrn.3705462

158. [COVID-19 trend in Bangladesh: deviation from epidemiological model and critical analysis of the possible factors](https://discover.hsls.pitt.edu/vivisimo/cgi-bin/query-meta?v%3afile=viv_vnkHDb&v%3astate=root%7croot-25-25%7c0&url=https%3a%2f%2fdoi.org%2f10.1101%2f2020.05.31.20118745&rid=Ndoc50&v%3aframe=redirect&v%3aredirect-hash=57aff2488481ba35d88b96ebbe0261e1&). Ahmed A, Rahman MM. medRxiv 2020-06-03. Doi:<https://doi.org/10.1101/2020.05.31.20118745>

159. COVID-19: Forecasting short term hospital needs in France. Clément Massonnaud, Jonathan Roux, Pascal Crépey. medRxiv 2020.03.16.20036939; doi:<https://doi.org/10.1101/2020.03.16.20036939>

160. COVID-19: Predictive Mathematical Models for the Number of Deaths in South Korea, Italy, Spain, France, UK, Germany, and USA. Athanasios S. Fokas, Nikolaos Dikaios, George A. Kastis. medRxiv 2020.05.08.20095489; doi:<https://doi.org/10.1101/2020.05.08.20095489>

161. COVID-19: Short term prediction model using daily incidence data. Hongwei Zhao, Naveed N Merchant, Alyssa McNulty, Tiffany A Radcliff, Murray J Cote, Rebecca Fischer, Huiyan Sang, Marcia G Ory. medRxiv 2020.11.23.20237024; doi:<https://doi.org/10.1101/2020.11.23.20237024>

162. COVID-19: The unreasonable effectiveness of simple models. Timoteo Carletti, Duccio Fanelli, Francesco Piazza. medRxiv 2020.05.26.20110957; doi: https://doi.org/10.1101/2020.05.26.20110957

163. COVID-19: Time-Dependent Effective Reproduction Number and Sub-notification Effect Estimation Modeling. Eduardo Atem De Carvalho, Rogerio Atem De Carvalho. medRxiv 2020.07.28.20164087; doi: https://doi.org/10.1101/2020.07.28.20164087

164. Crash-sensitive Kelly Strategy built on a modified Kreuser-Sornette bubble model tested over three decades of twenty equity indices. Gerlach J, Kreuser JL, Sornette D. SSRN  2020-10-09. Doi: https://doi.org/10.2139/ssrn.3708035

165. Cross-Cultural Polarity and Emotion Detection Using Sentiment Analysis and Deep Learning -- a Case Study on COVID-19. [Ali Shariq Imran](https://arxiv.org/search/?searchtype=author&query=Imran%2C+A+S), [Sher Mohammad Doudpota](https://arxiv.org/search/?searchtype=author&query=Doudpota%2C+S+M), [Zenun Kastrati](https://arxiv.org/search/?searchtype=author&query=Kastrati%2C+Z), [Rakhi Bhatra](https://arxiv.org/search/?searchtype=author&query=Bhatra%2C+R). [arXiv:2008.10031](https://arxiv.org/abs/2008.10031)

166. Cultural values predict national COVID-19 death rates. Damian J Ruck, Joshua Borycz, R. Alexander Bentley. medRxiv 2020.07.17.20156091; doi:<https://doi.org/10.1101/2020.07.17.20156091>

167. Cumulative Active and Recovery Rates Based Criterion for Gradual Lockdown Exit: A Global Observation of SARS Cov-2 Management. Dhananjay V Raje, Abhay Bajaj, Moumita Chakraborty, Hemant J. Purohit. medRxiv 2020.06.05.20123364; doi:<https://doi.org/10.1101/2020.06.05.20123364>

168. Curve-fitting approach for COVID-19 data and its physical background. Yoshiro Nishimoto, Kenichi Inoue. medRxiv 2020.07.02.20144899; doi: https://doi.org/10.1101/2020.07.02.20144899

169. Cyclic exit strategies to suppress COVID-19 and allow economic activity. Omer Karin, Yinon M. Bar-On, Tomer Milo, Itay Katzir, Avi Mayo, Yael Korem, Boaz Dudovich, Eran Yashiv, Amos J. Zehavi, Nadav Davidovitch, Ron Milo, Uri Alon. medRxiv 2020.04.04.20053579; doi:<https://doi.org/10.1101/2020.04.04.20053579>

170. Cytokine biomarkers of COVID-19. Hai-Jun Deng, Quan-Xin Long, Bei-Zhong Liu, Ji-Hua Ren, Pu Liao, Jing-Fu Qiu, Xiao-Jun Tang, Yong Zhang, Ni Tang, Yin-Yin Xu, Zhan Mo, Juan Chen, Jieli Hu, Ai-Long Huang. medRxiv 2020.05.31.20118315; doi:<https://doi.org/10.1101/2020.05.31.20118315>

171. Data driven inference of the reproduction number (R0) for COVID-19 before and after interventions for 51 European countries. Petr Karnakov, George Arampatzis, Ivica Kičić, Fabian Wermelinger, Daniel Wälchli, Costas Papadimitriou, Petros Koumoutsakos. medRxiv 2020.05.21.20109314; doi:<https://doi.org/10.1101/2020.05.21.20109314>

172. Data-driven modeling reveals a universal dynamic underlying the COVID-19 pandemic under social distancing. Robert Marsland III, Pankaj Mehta. medRxiv 2020.04.21.20073890; doi:<https://doi.org/10.1101/2020.04.21.20073890>

173. Data-driven Optimized Control of the COVID-19 Epidemics. Afroza Shirin, Yen Ting Lin, Francesco Sorrentino. medRxiv 2020.08.27.20183574; doi:<https://doi.org/10.1101/2020.08.27.20183574>

174. Deep Learning and Holt-Trend Algorithms for predicting COVID-19 pandemic. Theyazn H.H Aldhyani, Melfi Alrasheed Sr., Ahmed i Abdullah Alqarn Sr., Mohammed Y. Alzahrani, Ahmed H., Alahmadi. medRxiv 2020.06.03.20121590; doi:<https://doi.org/10.1101/2020.06.03.20121590>

175. Demand for hospitalization services for COVID-19 patients in Brazil. Marcia C. Castro, Lucas Resende de Carvalho, Taylor Chin, Rebecca Kahn, Giovanny V. A. França, Eduardo Marques Macário, Wanderson Kleber de Oliveira. medRxiv 2020.03.30.20047662; doi:<https://doi.org/10.1101/2020.03.30.20047662>

176. Demographic science aids in understanding the spread and fatality rates of COVID-19. Jennifer Beam Dowd, Liliana Andriano, Valentina Rotondi, David M. Brazel, Per Block, Xuejie Ding, Yan Liu, Melinda C. Mills. medRxiv 2020.03.15.20036293; doi:<https://doi.org/10.1101/2020.03.15.20036293>

177. Descriptive Epidemiological Assessment of the Relationship between the Global Burden of Influenza from 2017-2019 and COVID-19. Stefan D Baral, Katherine B Rucinski, Jean Olivier Twahirwa Rwema, Amrita Rao, Neia Prata Menezes, Daouda Diouf, Adeeba Kamarulzaman, Nancy Phaswana-Mafuya, Sharmistha Mishra. medRxiv 2020.06.18.20134346; doi:<https://doi.org/10.1101/2020.06.18.20134346>

178. Detecting COVID-19 infection hotspots in England using large-scale self-reported data from a mobile application: a prospective, observational study. Thomas Varsavsky, Mark S. Graham, Liane S. Canas, Sajaysurya Ganesh, Joan Capdevila Pujol, Carole H. Sudre, Benjamin Murray, Marc Modat, M. Jorge Cardoso, Christina M. Astley, David A Drew, Long H. Nguyen, Tove Fall, Maria F Gomez, Paul W. Franks, Andrew T. Chan, Richard Davies, Jonathan Wolf, Claire J. Steves, Tim D. Spector, Sebastien Ourselin. medRxiv 2020.10.26.20219659; doi:<https://doi.org/10.1101/2020.10.26.20219659>

179. Development and validation of an automated radiomic CT signature for detecting COVID-19. J. Guiot, A. Vaidyanathan, L. Deprez, F. Zerka, L. Danthine, A.N. Frix, M. Thys, M. Henket, G. Canivet, S. Mathieu, E. Eftaxia, P. Lambin, N. Tsoutzidis, B. Miraglio, S. Walsh, M. Moutschen, R. Louis, P. Meunier, W. Vos, R.T.H. Leijenaar, P. Lovinfosse. medRxiv 2020.04.28.20082966; doi:<https://doi.org/10.1101/2020.04.28.20082966>

180. Did COVID-19 infections decline before UK lockdown? [Simon N. Wood](https://arxiv.org/search/?searchtype=author&query=Wood%2C+S+N). [arXiv:2005.02090](https://arxiv.org/abs/2005.02090)

181. Did lockdowns serve their purpose? [Serena Bradde](https://arxiv.org/search/?searchtype=author&query=Bradde%2C+S), [Benedetta Cerruti](https://arxiv.org/search/?searchtype=author&query=Cerruti%2C+B), [Jean-Philippe Bouchaud](https://arxiv.org/search/?searchtype=author&query=Bouchaud%2C+J). [arXiv:2006.09829](https://arxiv.org/abs/2006.09829)

182. Differential Effects of Intervention Timing on COVID-19 Spread in the United States. Sen Pei, Sasikiran Kandula, Jeffrey Shaman. medRxiv 2020.05.15.20103655; doi:<https://doi.org/10.1101/2020.05.15.20103655>

183. Diminishing Marginal Benefit of Social Distancing in Balancing COVID-19 Medical Demand-to-Supply. Pai Liu, Payton Beeler, Rajan K. Chakrabarty. medRxiv 2020.04.09.20059550; doi:<https://doi.org/10.1101/2020.04.09.20059550>

184. Discrete simulation analysis of COVID-19 and prediction of isolation bed numbers. Xinyu Li, Yufeng Cai, Yinghe Ding, Jia-da Li, Guoqing Huang, Ye Liang, Linyong Xu. medRxiv 2020.07.13.20152330; doi:<https://doi.org/10.1101/2020.07.13.20152330>

185. Distributional challenges regarding data on death and incidences during the SARS-CoV-2 pandemic up to July 2020. Kirsi Manz, Ulrich Mansmann. medRxiv 2020.07.24.20161257; doi:<https://doi.org/10.1101/2020.07.24.20161257>

186. Do Weather Temperature and Median-age affect COVID-19 Transmission?. Aly Zein Elabdeen Kassem. medRxiv 2020.04.16.20067355; doi: https://doi.org/10.1101/2020.04.16.20067355

187. Don’t wait, re-escalate: delayed action results in longer duration of COVID-19 restrictions. Amy Hurford, James Watmough. medRxiv 2020.11.04.20226316; doi:<https://doi.org/10.1101/2020.11.04.20226316>

188. Doubling Time of the COVID-19 Epidemic by Chinese Province. Kamalich Muniz-Rodriguez, Gerardo Chowell, Chi-Hin Cheung, Dongyu Jia, Po-Ying Lai, Yiseul Lee, Manyun Liu, Sylvia K. Ofori, Kimberlyn M. Roosa, Lone Simonsen, Cecile Viboud, Isaac Chun-Hai Fung. medRxiv 2020.02.05.20020750; doi:<https://doi.org/10.1101/2020.02.05.20020750>

189. Dynamic Estimation of Epidemiological Parameters of COVID-19 Outbreak and Effects of Interventions on Its Spread. Hongzhe Zhang, Xiaohang Zhao, Kexin Yin, Yiren Yan, Wei Qian, Bintong Chen, Xiao Fang. medRxiv 2020.04.01.20050310; doi:<https://doi.org/10.1101/2020.04.01.20050310>

190. Dynamical balance between the transmission, intervention of COVID-19 and economic development. Zhaowang Zhang, Hualiang Lin, Guanghu Zhu. medRxiv 2020.08.13.20174755; doi:<https://doi.org/10.1101/2020.08.13.20174755>

191. Dynamical model for social distancing in the U.S. during the COVID-19 epidemic. Shirish M. Chitanvis. medRxiv 2020.05.18. 20105411; doi:<https://doi.org/10.1101/2020.05.18.20105411>

192. Dynamical SPQEIR model assesses the effectiveness of non-pharmaceutical interventions against COVID-19 epidemic outbreaks. Daniele Proverbio, Françoise Kemp, Stefano Magni, Andreas Husch, Atte Aalto, Laurent Mombaerts, Alexander Skupin, Jorge Gonçalves, Jose Ameijeiras-Alonso, Christophe Ley. medRxiv 2020.04.22.20075804; doi:<https://doi.org/10.1101/2020.04.22.20075804>

193. Dynamics and future of SARS-CoV-2 in the human host. Michael Gillman, Nuno Crokidakis. medRxiv 2020.07.14.20153270; doi:<https://doi.org/10.1101/2020.07.14.20153270>

194. Dynamics of the covid-19 pandemics: global pattern and between countries variations. Julien Krywyk, Walther Oettgen, Marc Messier, Matthieu Mulot, Laurent Toubiana. medRxiv 2020.07.20. 20155390; doi:<https://doi.org/10.1101/2020.07.20.20155390>

195. Early detection of seasonality and second-wave prediction in the COVID-19 pandemic. Márcio Watanabe. medRxiv 2020.09.02.20187203; doi: https://doi.org/10.1101/2020.09.02.20187203

196. Easing social distancing index after COVID-19 pandemic. Li-Sheng Chen, Ming-Fang Yen, Chao-Chih Lai, Chen-Yang Hsu, Hsiu-Hsi Chen. medRxiv 2020.06.11.20128165; doi:<https://doi.org/10.1101/2020.06.11.20128165>

197. Eco-epidemiological assessment of the COVID-19 epidemic in China, January-February 2020. Peter Byass. medRxiv 2020.03.29.20046565; doi:<https://doi.org/10.1101/2020.03.29.20046565>

198. Effectiveness of quarantine measure on transmission dynamics of COVID-19 in Hong Kong. Hsiang-Yu Yuan, Axiu Mao, Guiyuan Han, Hsiangkuo Yuan, Dirk Pfeiffer. medRxiv 2020.04.09.20059006; doi:<https://doi.org/10.1101/2020.04.09.20059006>

199. Effects of non-pharmaceutical interventions on COVID-19: A Tale of Three Models. Vincent Chin, John P.A. Ioannidis, Martin A. Tanner, Sally Cripps. medRxiv 2020.07.22.20160341; doi:<https://doi.org/10.1101/2020.07.22.20160341>

200. Effects of temperature and humidity on the spread of COVID-19: A systematic review. Paulo Mecenas, Renata Travassos da Rosa Moreira Bastos, Antonio Carlos Rosário Vallinoto, David Normando. medRxiv 2020.04.14.20064923; doi:<https://doi.org/10.1101/2020.04.14.20064923>

201. Effects of the COVID-19 Pandemic on Population Mobility under Mild Policies: Causal Evidence from Sweden. [Matz Dahlberg](https://arxiv.org/search/?searchtype=author&query=Dahlberg%2C+M), [Per-Anders Edin](https://arxiv.org/search/?searchtype=author&query=Edin%2C+P), [Erik Grönqvist](https://arxiv.org/search/?searchtype=author&query=Gr%C3%B6nqvist%2C+E), [Johan Lyhagen](https://arxiv.org/search/?searchtype=author&query=Lyhagen%2C+J), [John Östh](https://arxiv.org/search/?searchtype=author&query=%C3%96sth%2C+J), [Alexey Siretskiy](https://arxiv.org/search/?searchtype=author&query=Siretskiy%2C+A), [Marina Toger](https://arxiv.org/search/?searchtype=author&query=Toger%2C+M). [arXiv:2004.09087](https://arxiv.org/abs/2004.09087)

202. Effects of voluntary event cancellation and school closure as countermeasures against COVID−19 outbreak in Japan. Yoshiyuki Sugishita, Junko Kurita, Tamie Sugawara, Yasushi Ohkusa. medRxiv 2020.03.19.20037945; doi:<https://doi.org/10.1101/2020.03.19.20037945>

203. Environmental indicator for effective control of COVID-19 spreading. Xinbo Lian, Jianping Huang Sr., Li Zhang, Chuwei Liu, Xiaoyue Liu, Lina Wang. medRxiv 2020.05.12.20099804; doi:<https://doi.org/10.1101/2020.05.12.20099804>

204. Epidemic analysis of COVID-19 Outbreak and Counter-Measures in France. Eren Unlu, Hippolyte Léger, Oleksandr Motornyi, Alia Rukubayihunga, Thibaud Ishacian, Mehdi Chouiten. medRxiv 2020.04.27.20079962; doi:<https://doi.org/10.1101/2020.04.27.20079962>

205. Epidemic Model Guided Machine Learning for COVID-19 Forecasts in the United States. Difan Zou, Lingxiao Wang, Pan Xu, Jinghui Chen, Weitong Zhang, Quanquan Gu. medRxiv 2020.05.24.20111989; doi:<https://doi.org/10.1101/2020.05.24.20111989>

206. Epidemics forecast from SIR-modeling, verification and calculated effects of lockdown and lifting of interventions. Reinhard Schlickeiser, Martin Kroger. medRxiv 2020.08.12.20173294; doi:<https://doi.org/10.1101/2020.08.12.20173294>

207. Epidemiological model for the inhomogeneous spatial spreading of COVID-19 and other diseases. Yoav Tsori, Rony Granek. medRxiv 2020.07.08.20148767; doi:<https://doi.org/10.1101/2020.07.08.20148767>

208. Epidemiological Profile and Transmission Dynamics of COVID-19 in the Philippines. Nel Jason Ladiao Haw, Jhanna Uy, Karla Therese L. Sy. medRxiv 2020.07.15.20154336; doi:<https://doi.org/10.1101/2020.07.15.20154336>

209. Estimates of the proportion of SARS-CoV-2 infected individuals in Sweden. [Henrik Hult](https://arxiv.org/search/?searchtype=author&query=Hult%2C+H), [Martina Favero](https://arxiv.org/search/?searchtype=author&query=Favero%2C+M). [arXiv:2005.13519](https://arxiv.org/abs/2005.13519)

210. Estimating and forecasting COVID-19 attack rates and mortality. David I. Ketcheson, Hernando C. Ombao, Paula Moraga, Tarig Ballal, Carlos M. Duarte. medRxiv 2020.05.11.20097972; doi:<https://doi.org/10.1101/2020.05.11.20097972>

211. Estimating critical care capacity needs and gaps in Africa during the COVID-19 pandemic. Jessica Craig, Erta Kalanxhi, Gilbert Osena, Isabel Frost. medRxiv 2020.06.02.20120147; doi:<https://doi.org/10.1101/2020.06.02.20120147>

212. Estimating Impact of Austerity policies in COVID-19 fatality rates: Examining the dynamics of economic policy and Case Fatality Rates (CFR) of COVID-19 in OECD countries. Dawa Sherpa. medRxiv 2020.04.03.20047530; doi:<https://doi.org/10.1101/2020.04.03.20047530>

213. Estimating the Changing Infection Rate of COVID-19 Using Bayesian Models of Mobility. Luyang Liu, Sharad Vikram, Junpeng Lao, Xue Ben, Alexander D'Amour, Shawn O'Banion, Mark Sandler, Rif A. Saurous, Matthew D. Hoffman. medRxiv 2020.08.06.20169664; doi:<https://doi.org/10.1101/2020.08.06.20169664>

214. Estimating the COVID-19 epidemic trajectory and hospital capacity requirements in South West England: a mathematical modelling framework. Ross D Booton, Louis MacGregor, Lucy Vass, Katharine J Looker, Catherine Hyams, Philip D Bright, Irasha Harding, Rajeka Lazarus, Fergus Hamilton, Daniel Lawson, Leon Danon, Adrian Pratt, Richard Wood, Ellen Brooks-Pollock, Katherine M E Turner. medRxiv 2020.06.10.20084715; doi:<https://doi.org/10.1101/2020.06.10.20084715>

215. Estimating the Cumulative Incidence of COVID-19 in the United States Using Four Complementary Approaches. Fred S Lu, Andre T Nguyen, Nicholas B Link, Jessica T Davis, Matteo Chinazzi, Xinyue Xiong, Alessandro Vespignani, Marc Lipsitch, Mauricio Santillana. medRxiv 2020.04.18.20070821; doi:<https://doi.org/10.1101/2020.04.18.20070821>

216. Estimating the effect of social inequalities in the mitigation of COVID-19 across communities in Santiago de Chile. Nicolò Gozzi, Michele Tizzoni, Matteo Chinazzi, Leo Ferres, Alessandro Vespignani, Nicola Perra. medRxiv 2020.10.08.20204750; doi:<https://doi.org/10.1101/2020.10.08.20204750>

217. Estimating the establishment of local transmission and the cryptic phase of the COVID-19 pandemic in the USA. Jessica T Davis, Matteo Chinazzi, Nicola Perra, Kunpeng Mu, Ana Pastore y Piontti, Marco Ajelli, Natalie E Dean, Corrado Gioannini, Maria Litvinova, Stefano Merler, Luca Rossi, Kaiyuan Sun, Xinyue Xiong, M. Elizabeth Halloran, Ira M Longini Jr., Cécile Viboud, Alessandro Vespignani. medRxiv 2020.07.06.20140285; doi:<https://doi.org/10.1101/2020.07.06.20140285>

218. Estimating the impact of mobility patterns on COVID-19 infection rates in 11 European countries. Patrick Bryant, Arne Elofsson. medRxiv 2020.04.13.20063644; doi:<https://doi.org/10.1101/2020.04.13.20063644>

219. Estimating the infection fatality risk of COVID-19 in New York City during the spring 2020 pandemic wave. Wan Yang, Sasikiran Kandula, Mary Huynh, Sharon K Greene, Gretchen Van Wye, Wenhui Li, Hiu Tai Chan, Emily McGibbon, Alice Yeung, Don Olson, Anne Fine, Jeffrey Shaman. medRxiv 2020.06.27.20141689; doi:<https://doi.org/10.1101/2020.06.27.20141689>

220. Estimating the number of SARS-CoV-2 infections in the United States. Dayton G. Thorpe, Kelsey Lyberger. medRxiv 2020.04.13.20064519; doi:<https://doi.org/10.1101/2020.04.13.20064519>

221. Estimating the reproduction number of COVID-19 in Iran using epidemic modeling. Ebrahim Sahafizadeh, Samaneh Sartoli. medRxiv 2020.03.20.20038422; doi:<https://doi.org/10.1101/2020.03.20.20038422>

222. Estimating the size of COVID-19 epidemic outbreak. Chakrit Pongkitivanichkul, Daris Samart, Takol Tangphati, Phanit Koomhin, Pimchanok Pimton, Punsiri Dam-O, Apirak Payaka, Phongpichit Channuie. medRxiv 2020.03.28.20044339; doi:<https://doi.org/10.1101/2020.03.28.20044339>

223. Estimating the size of undetected cases of the SARS-CoV-2 outbreak in Europe: An upper bound estimator. Irene Rocchetti, Dankmar Boehning, Heinz Holling, Antonello Maruotti. medRxiv 2020.07.14.20153445; doi:<https://doi.org/10.1101/2020.07.14.20153445>

224. Estimating the true (population) infection rate for COVID-19: A Backcasting Approach with Monte Carlo Methods. Steven J. Phipps, R. Quentin Grafton, Tom Kompas. medRxiv 2020.05.12.20098889; doi:<https://doi.org/10.1101/2020.05.12.20098889>

225. Estimating underdiagnosis of covid-19 with nowcasting and machine learning: experience from brazil. Leandro Pereira Garcia, Andre Vinicius Goncalves, Matheus Pacheco de Andrade, Lucas Alexandre Pedebos, Ana Cristina Vidor, Roberto Zaina, Graziela de Luca Canto, Gustavo Medeiros de Araujo, Fernanda Vargas Amaral. medRxiv 2020.07.01.20144402; doi:<https://doi.org/10.1101/2020.07.01.20144402>

226. Estimating weekly excess mortality at sub-national level in Italy during the COVID-19 pandemic. Marta Blangiardo, Michela Cameletti, Monica Pirani, Gianni Corsetti, Marco Battaglini, Gianluca Baio. medRxiv 2020.06.08.20125211; doi: https://doi.org/10.1101/2020.06.08.20125211

227. Estimation and worldwide monitoring of the effective reproductive number of SARS-CoV-2. Jana S. Huisman, Jérémie Scire, Daniel C. Angst, Richard A. Neher, Sebastian Bonhoeffer, Tanja Stadler. medRxiv 2020.11.26.20239368; doi:<https://doi.org/10.1101/2020.11.26.20239368>

228. Estimation of COVID-19 risk-stratified epidemiological parameters and policy implications for Los Angeles County through an integrated risk and stochastic epidemiological model. Abigail L. Horn, Lai Jiang, Faith Washburn, Emil Hvitfeldt, Kayla de la Haye, William Nicholas, Paul Simon, Maryann Pentz, Wendy Cozen, Neeraj Sood, David V. Conti. medRxiv 2020.12.11.20209627; doi: https://doi.org/10.1101/2020.12.11.20209627

229. Estimation of COVID-19 spread curves integrating global data and borrowing information. Se Yoon Lee, Bowen Lei, Bani K. Mallick. medRxiv 2020.04.23.20077065; doi: https://doi.org/10.1101/2020.04.23.20077065

230. Estimation of novel coronavirus (covid-19) reproduction number and case fatality rate: a systematic review and meta-analysis. Tanvir Ahammed, Aniqua Anjum, Mohammad Meshbahur Rahman, Najmul Haider, Richard Kock, Md. Jamal Uddin. medRxiv 2020.09.30.20204644; doi:<https://doi.org/10.1101/2020.09.30.20204644>

231. Estimation of the final size of the COVID-19 epidemic. Milan Batista. medRxiv 2020.02.16.20023606; doi:<https://doi.org/10.1101/2020.02.16.20023606>

232. Estimation of Transmission Potential and Severity of COVID–19 in Romania and Pakistan. Muhammad Ozair, Takasar Hussain, Mureed Hussain, Aziz Ullah Awan, Dumitru Baleanu. medRxiv 2020.05.02.20088989; doi:<https://doi.org/10.1101/2020.05.02.20088989>

233. [Estimation of COVID-19 spread curves integrating global data and borrowing information](https://discover.hsls.pitt.edu/vivisimo/cgi-bin/query-meta?v%3afile=viv_vnkHDb&v%3astate=root%7croot-75-25%7c0&url=https%3a%2f%2fdoi.org%2f10.1101%2f2020.04.23.20077065&rid=Ndoc77&v%3aframe=redirect&v%3aredirect-hash=e759885104c9b48d75b9f13340caeafd&). Lee SY, Lei B, Mallick BK. medRxiv 2020-04-29. Doi:<https://doi.org/10.1101/2020.04.23.20077065>

234. European lockdowns and the consequences of relaxation during the COVID-19 pandemic. David H Glass. medRxiv 2020.05.19.20106542; doi:<https://doi.org/10.1101/2020.05.19.20106542>

235. Evaluating Data-Driven Forecasting Methods for Predicting SARS-CoV2 Cases: Evidence From 173 Countries. Ghufran Ahmad, Furqan Ahmed, Muhammad Suhail Rizwan, Javed Muhammad, Hira Fatima, Aamer Ikram, Hajo Zeeb. medRxiv 2020.08.03.20167189; doi:<https://doi.org/10.1101/2020.08.03.20167189>

236. Evaluating growth pattern and assessing future scenario of COVID-19 epidemic of India. Nandan L. Patil, Lakshmi R. Gangavati. medRxiv 2020.05.02.20087544; doi:<https://doi.org/10.1101/2020.05.02.20087544>

237. Evaluating reduction in CoViD-19 cases by isolation and protective measures in São Paulo State, Brazil, and scenarios of release. Hyun Mo Yang, Luis Pedro Pedro Lombardi Junior, Fábio Fernandes Morato Castro, Ariana Campos Yang. medRxiv 2020.05.19.20099309; doi:<https://doi.org/10.1101/2020.05.19.20099309>

238. Evaluating the effect of public health intervention on the global-wide spread trajectory of Covid-19. Zixin Hu, Qiyang Ge, Shudi Li, Li Jin, Momiao Xiong. medRxiv 2020.03.11.20033639; doi:<https://doi.org/10.1101/2020.03.11.20033639>

239. Evaluating the effectiveness of social distancing interventions against COVID-19. Laura Matrajt, Tiffany Leung. medRxiv 2020.03.27.20044891; doi:<https://doi.org/10.1101/2020.03.27.20044891>

240. Evaluating the impact of international airline suspensions on the early global spread of COVID-19. Aniruddha Adiga, Srinivasan Venkatramanan, James Schlitt, Akhil Peddireddy, Allan Dickerman, Andrei Bura, Andrew Warren, Brian D Klahn, Chunhong Mao, Dawen Xie, Dustin Machi, Erin Raymond, Fanchao Meng, Golda Barrow, Henning Mortveit, Jiangzhuo Chen, Jim Walke, Joshua Goldstein, Mandy L Wilson, Mark Orr, Przemyslaw Porebski, Pyrros A Telionis, Richard Beckman, Stefan Hoops, Stephen Eubank, Young Yun Baek, Bryan Lewis, Madhav Marathe, Chris Barrett. medRxiv 2020.02.20.20025882; doi:<https://doi.org/10.1101/2020.02.20.20025882>

241. Evaluating the use of the reproduction number as an epidemiological tool, using spatio-temporal trends of the Covid-19 outbreak in England. Katharine Sherratt, Sam Abbott, Sophie R Meakin, Joel Hellewell, James D Munday, Nikos Bosse, CMMID Covid-19 working group, Mark Jit, Sebastian Funk. medRxiv 2020.10.18.20214585; doi:<https://doi.org/10.1101/2020.10.18.20214585>

242. Evaluation of Nowcasting for Real-Time COVID-19 Tracking — New York City, March–May 2020. Sharon K. Greene, Sarah F. McGough, Gretchen M. Culp, Laura E. Graf, Marc Lipsitch, Nicolas A. Menzies, Rebecca Kahn. medRxiv 2020.10.18.20209189; doi:<https://doi.org/10.1101/2020.10.18.20209189>

243. Evidence of the effectiveness of travel-related measures during the early phase of the COVID- 19 pandemic: a rapid systematic review. Karen A. Grépin, Tsi Lok Ho, Zhihan Liu, Summer Marion, Julianne Piper, Catherine Z. Worsnop, Kelley Lee. medRxiv 2020.11.23.20236703; doi:<https://doi.org/10.1101/2020.11.23.20236703>

244. [Evolution of disease transmission rate during the course of SARS-COV-2: Patterns and determinants](https://discover.hsls.pitt.edu/vivisimo/cgi-bin/query-meta?v%3afile=viv_vnkHDb&v%3astate=root%7croot-25-25%7c0&url=https%3a%2f%2fdoi.org%2f10.21203%2frs.3.rs-44647%2fv1&rid=Ndoc34&v%3aframe=redirect&v%3aredirect-hash=80078f32306fbc40213bae8b05570fc6&). Zhu J, Gallego B. Research Square 2020-07-21. Doi:<https://doi.org/10.21203/rs.3.rs-44647/v1>

245. Examining the status of improved air quality due to COVID-19 lockdown and an associated reduction in anthropogenic emissions. Srikanta Sannigrahi, Anna Molter, Prashant Kumar, Qi Zhang, Bidroha Basu, Arunima Sarkar Basu, Francesco Pilla. medRxiv 2020.08.20.20177949; doi:<https://doi.org/10.1101/2020.08.20.20177949>

246. Extended SEIQR type model for COVID-19 epidemic and data analysis. Swarnali Sharma, Vitaly Volpert, Malay Banerjee. medRxiv 2020.08.10.20171439; doi:<https://doi.org/10.1101/2020.08.10.20171439>

247. Extending the susceptible-exposed-infected-removed(seir) model to handle the high false negative rate and symptom-based administration of covid-19 diagnostic tests: SEIR-fansy. Ritwik Bhaduri, Ritoban Kundu, Soumik Purkayastha, Mike Kleinsasser, Lauren J Beesley, Bhramar Mukherjee. medRxiv 2020.09.24.20200238; doi:<https://doi.org/10.1101/2020.09.24.20200238>

248. Extrapolation of Infection Data for the CoVid-19 Virus in 21 Countries and States and Estimate of the Efficiency of Lock Down. Walter Langel. medRxiv 2020.06.17.20134254; doi:<https://doi.org/10.1101/2020.06.17.20134254>

249. Fast initial Covid-19 response means greater caution may be needed later. Joel Joel-Marie Hirschi. medRxiv 2020.05.26.20112680; doi:<https://doi.org/10.1101/2020.05.26.20112680>

250. Feasibility of Controlling COVID-19 Outbreaks in the UK by Rolling Interventions. Po Yang, Jun Qi, Shuhao Zhang, Xulong wang, Gaoshan Bi, Yun Yang, Bin Sheng, Xuxin Mao. medRxiv 2020.04.05.20054429; doi:<https://doi.org/10.1101/2020.04.05.20054429>

251. Fine-tuned Forecasting Techniques for COVID-19 Prediction in India. Abhinav Gola, Ravi Kumar Arya, Animesh Animesh, Ravi Dugh, Zuber Khan. medRxiv 2020.08.10.20167247; doi:<https://doi.org/10.1101/2020.08.10.20167247>

252. Flatten the Curve! Modeling SARS-CoV-2/COVID-19 Growth in Germany at the County Level. Thomas Wieland. medRxiv 2020.05.14.20101667; doi:<https://doi.org/10.1101/2020.05.14.20101667>

253. Forecasting Confirmed Cases and Mortalities of COVID-19 in the US. Babak Jamshidi, Mohsen Kakavandi, Shahriar Jamshidi Zargaran, Amir Talaei-Khoei. medRxiv 2020.10.30.20223412; doi:<https://doi.org/10.1101/2020.10.30.20223412>

254. Forecasting COVID-19 cases and deaths in epidemic-mitigating European countries by Richards function-based regression analyses. Cheng Long, Xinmiao Fu. medRxiv 2020.05.18.20106146; doi:<https://doi.org/10.1101/2020.05.18.20106146>

255. Forecasting COVID-19 cases using Machine Learning models. Yuan Tian, Ishika Luthra, Xi Zhang. medRxiv 2020.07.02.20145474; doi:<https://doi.org/10.1101/2020.07.02.20145474>

256. Forecasting COVID-19 impact in India using pandemic waves Nonlinear Growth Models. Pavan Kumar, Ram Kumar Singh, Chintan Nanda, Himangshu Kalita, Shashikanta Patairiya, Yagya Datt Sharma, Meenu Rani, Akshaya Srikanth Bhagavathula

257. Forecasting covid-19 pandemic: a data-driven analysis. Khondoker Nazmoon Nabi. medRxiv 2020.05.12.20099192; doi:<https://doi.org/10.1101/2020.05.12.20099192>

258. Forecasting intensive care unit demand during the COVID-19 pandemic: A spatial age-structured microsimulation model. Sebastian Kluesener, Ralf Schneider, Matthias Rosenbaum-Feldbruegge, Christian Dudel, Elke Loichinger, Nikola Sander, Andreas Backhaus, Emanuele Del Fava, Janina Esins, Martina Fischer, Linus Grabenhenrich, Pavel Grigoriev, Andre Grow, Jason Hilton, Bastian Koller, Mikko Myrskyla, Francesco Scalone, Martin Wolkewitz, Emilio Zagheni, Michael M. Resch. medRxiv 2020.12.23.20248761; doi:<https://doi.org/10.1101/2020.12.23.20248761>

259. Forecasting the dynamics of COVID-19 Pandemic in Top 15 countries in April 2020: ARIMA Model with Machine Learning Approach. Pavan Kumar, Himangshu Kalita, Shashikanta Patairiya, Yagya Datt Sharma, Chintan Nanda, Meenu Rani, Jamal Rahmani, Akshaya Srikanth Bhagavathula. medRxiv 2020.03.30.20046227; doi:  <https://doi.org/10.1101/2020.03.30.20046227>

260. Forecasting the impact of the first wave of the COVID-19 pandemic on hospital demand and deaths for the USA and European Economic Area countries. IHME COVID-19 health service utilization forecasting team, Christopher JL Murray. medRxiv 2020.04.21.20074732; doi:<https://doi.org/10.1101/2020.04.21.20074732>

261. [Forecasting the Olympic Medal Distribution during a Pandemic: A Socio-Economic Machine Learning Model](https://discover.hsls.pitt.edu/vivisimo/cgi-bin/query-meta?v%3afile=viv_vnkHDb&v%3astate=root%7croot&url=https%3a%2f%2fdoi.org%2f10.2139%2fssrn.3745595&rid=Ndoc3&v%3aframe=redirect&v%3aredirect-hash=272a518445b590283ef8524d227ae387&) [new window](https://discover.hsls.pitt.edu/vivisimo/cgi-bin/query-meta?v%3afile=viv_vnkHDb&v%3astate=root%7croot&url=https%3a%2f%2fdoi.org%2f10.2139%2fssrn.3745595&rid=Ndoc3&v%3aframe=redirect&v%3aredirect-hash=272a518445b590283ef8524d227ae387&). Schlembach C, Schmidt SL, Schreyer D, Wunderlich L. SSRN:<https://doi.org/10.2139/ssrn.3745595>

262. Forecasting the scale of the COVID-19 epidemic in Kenya. Samuel P. C. Brand, Rabia Aziza, Ivy K. Kombe, Charles N. Agoti, Joe Hilton, Kat S. Rock, Andrea Parisi, D. James Nokes, Matt J. Keeling, Edwine W. Barasa. medRxiv 2020.04.09.20059865; doi:<https://doi.org/10.1101/2020.04.09.20059865>

263. Forecasting the Worldwide Spread of COVID-19 based on Logistic Model and SEIR Model. Xiang Zhou, Xudong Ma, Na Hong, Longxiang Su, Yingying Ma, Jie He, Huizhen Jiang, Chun Liu, Guangliang Shan, Weiguo Zhu, Shuyang Zhang, Yun Long. medRxiv 2020.03.26.20044289; doi:<https://doi.org/10.1101/2020.03.26.20044289>

264. Forecasting Trajectories of an Emerging Epidemic with Mathematical Modeling in an Online Dashboard: the Case of COVID-19. Wojciech Młocek, Robert Lew. medRxiv 2020.05.21.20108753; doi:<https://doi.org/10.1101/2020.05.21.20108753>

265. Fractal kinetics of COVID-19 pandemic (with update 3/1/20). Anna L. Ziff, Robert M. Ziff. medRxiv 2020.02.16.20023820; doi:<https://doi.org/10.1101/2020.02.16.20023820>

266. From 5Vs to 6Cs: Operationalizing Epidemic Data Management with COVID-19 Surveillance. Akhil Sai Peddireddy, Dawen Xie, Pramod Patil, Mandy L. Wilson, Dustin Machi, Srinivasan Venkatramanan, Brian Klahn, Przemyslaw Porebski, Parantapa Bhattacharya, Shirish Dumbre, Erin Raymond, Madhav Marathe. medRxiv 2020.10.27.20220830; doi:<https://doi.org/10.1101/2020.10.27.20220830>

267. Full lockdown policies in Western Europe countries have no evident impacts on the COVID-19 epidemic. Thomas Meunier. medRxiv 2020.04.24.20078717; doi:<https://doi.org/10.1101/2020.04.24.20078717>

268. Fusing a Bayesian case velocity model with random forest for predicting COVID-19 in the U.S. Gregory L. Watson, Di Xiong, Lu Zhang, Joseph A. Zoller, John Shamshoian, Phillip Sundin, Teresa Bufford, Anne W. Rimoin, Marc A. Suchard, Christina M. Ramirez. medRxiv 2020.05.15.20102608; doi:<https://doi.org/10.1101/2020.05.15.20102608>

269. Genetic drift and regional spreading dynamics of COVID-19. Saverio Alberti, Roberta Di Pietro, Mariangela Basile, Laura Antolini. medRxiv 2020.05.08.20095448; doi:<https://doi.org/10.1101/2020.05.08.20095448>

270. Geographic access to COVID-19 healthcare in Brazil using a balanced float catchment area approach. Rafael H. M. Pereira, Carlos Kaue Vieira Braga, Luciana Mendes Servo, Bernardo Serra, Pedro Amaral, Nelson Gouveia, Antonio Paez. medRxiv 2020.07.17.20156505; doi:<https://doi.org/10.1101/2020.07.17.20156505>

271. Global Analysis of an SEIRS Model for COVID-19 Capturing Saturated Incidence with Treatment Response. David Adeyemi Oluyori, Helen O. Adebayo, Ángel G. C. Pérez. medRxiv 2020.05.15.20103630; doi:<https://doi.org/10.1101/2020.05.15.20103630>

272. Global analysis of daily new COVID-19 cases reveals many static-phase countries including US and UK potentially with unstoppable epidemics. Xinmiao Fu. medRxiv 2020.05.08.20095356; doi:<https://doi.org/10.1101/2020.05.08.20095356>

273. Global and local mobility as a barometer for COVID-19 dynamics. Kevin Linka, Alain Goriely, Ellen Kuhl. medRxiv 2020.06.13.20130658; doi:<https://doi.org/10.1101/2020.06.13.20130658>

274. Global Economic Cost of Deaths Attributable to Ambient Air Pollution: Disproportionate Burden on the Ageing Population. Hao Yin, Michael Brauer, Junfeng (Jim) Zhang, Wenjia Cai, Ståle Navrud, Richard Burnett, Courtney Howard, Zhu Deng, Daniel M. Kammen, Hans Joachim Schellnhuber, Kai Chen, Haidong Kan, Zhanming Chen, Bin Chen, Ning Zhang, Zhifu Mi, D’Maris Coffman, Yiming Wei, Aaron Cohen, Dabo Guan, Qiang Zhang, Peng Gong, Zhu Liu. medRxiv 2020.04.28.20083576; doi:<https://doi.org/10.1101/2020.04.28.20083576>

275. Global prediction of unreported SARS-CoV2 infection from observed COVID-19 cases. Carson C. Chow, Joshua C. Chang, Richard C. Gerkin, Shashaank Vattikuti. medRxiv 2020.04.29.20083485; doi:<https://doi.org/10.1101/2020.04.29.20083485>

276. Global projections of lives saved from COVID-19 with universal mask use. medRxiv 2020.10.08.20209510; doi:<https://doi.org/10.1101/2020.10.08.20209510>

277. Global transmission network of SARS-CoV-2: from outbreak to pandemic. Pavel Skums, Alexander Kirpich, Pelin Icer Baykal, Alex Zelikovsky, Gerardo Chowell. medRxiv 2020.03.22.20041145; doi:<https://doi.org/10.1101/2020.03.22.20041145>

278. Health and Economic Costs of Early, Delayed and No Suppression of COVID-19: The Case of Australia. Tom Kompas, R Quentin Grafton, Tuong Nhu Che, Long Chu, James Camac. medRxiv 2020.06.21.20136549; doi:<https://doi.org/10.1101/2020.06.21.20136549>

279. Herd immunity vs suppressed equilibrium in COVID-19 pandemic: different goals require different models for tracking. Norden E. Huang, Fangli Qiao, Wang Qian, Ka-Kit Tung. medRxiv 2020.03.28.20046177; doi:<https://doi.org/10.1101/2020.03.28.20046177>

280. Hindsight is 2020 vision: a characterisation of the global response to the COVID-19 pandemic. David J. Warne, Anthony Ebert, Christopher Drovandi, Wenbiao Hu, Antonietta Mira, Kerrie Mengersen. medRxiv 2020.04.30.20085662; doi:<https://doi.org/10.1101/2020.04.30.20085662>

281. Household Secondary Attack Rate of COVID-19 and Associated Determinants. Qin-Long Jing, Ming-Jin Liu, Jun Yuan, Zhou-Bin Zhang, An-Ran Zhang, Natalie E. Dean, Lei Luo, Mengmeng Ma, Ira Longini, Eben Kenah, Ying Lu, Yu Ma, Neda Jalali, Li-Qun Fang, Zhi-Cong Yang, Yang Yang. medRxiv 2020.04.11.20056010; doi:<https://doi.org/10.1101/2020.04.11.20056010>

282. How did governmental interventions affect the spread of COVID-19 in European countries? Richard Andries Jacobus Post, Marta Regis, Zhuozhao Zhan, Edwin R van den Heuvel. medRxiv 2020.05.27.20114272; doi:<https://doi.org/10.1101/2020.05.27.20114272>

283. How much leeway is there to relax COVID-19 control measures?. Sean C. Anderson, Nicola Mulberry, Andrew M. Edwards, Jessica E. Stockdale, Sarafa A. Iyaniwura, Rebeca C. Falcao, Michael C. Otterstatter, Naveed Z. Janjua, Daniel Coombs, Caroline Colijn. medRxiv 2020.06.12.20129833; doi: https://doi.org/10.1101/2020.06.12.20129833

284. How to evaluate the success of the COVID-19 measures implemented by the Norwegian government by analyzing changes in doubling time. Biljana Stangeland. medRxiv 2020.03.29.20045187; doi:<https://doi.org/10.1101/2020.03.29.20045187>

285. Identification of Patterns in Epidemic Cycles and Methods for Estimating Their Duration: COVID-19 Case Study. Eduardo Atem De Carvalho, Rogerio Atem De Carvalho. medRxiv 2020.07.13.20153080; doi:<https://doi.org/10.1101/2020.07.13.20153080>

286. Identifying Optimal COVID-19 Testing Strategies for Schools and Businesses: Balancing Testing Frequency, Individual Test Technology, and Cost. Gregory D. Lyng, Natalie E. Sheils, Caleb J. Kennedy, Daniel Griffin, Ethan M. Berke. medRxiv 2020.10.11. 20211011; doi:<https://doi.org/10.1101/2020.10.11.20211011>

287. Impact of COVID-19 Second Wave on Healthcare Networks in the United States. Emad M. Hassan, Hussam Mahmoud. medRxiv 2020.07.11.20151217; doi:<https://doi.org/10.1101/2020.07.11.20151217>

288. Impact of lock down relaxation on the COVID-19 epidemic trajectory in Bangladesh. Shafiun Nahin Shimul, Mofakhar Hussain, Abu Jamil Faisel, Syed Abdul Hamid. medRxiv 2020.07.20.20158527; doi:<https://doi.org/10.1101/2020.07.20.20158527>

289. Impact of national lockdown on COVID-19 deaths in select European countries and the US using a Changes-in-Changes model. [Mudit Kapoor](https://arxiv.org/search/?searchtype=author&query=Kapoor%2C+M), [Shamika Ravi](https://arxiv.org/search/?searchtype=author&query=Ravi%2C+S). [arXiv:2006.12251](https://arxiv.org/abs/2006.12251)

290. Impact of relaxing Covid-19 social distancing measures on rural North Wales: a simulation analysis. Rhodri P Hughes, Dyfrig A Hughes. medRxiv 2020.05.15.20102764; doi:<https://doi.org/10.1101/2020.05.15.20102764>

291. Impact of Social Distancing Measures on COVID-19 Healthcare Demand in Central Texas. Xutong Wang, Remy F Pasco, Zhanwei Du, Michaela Petty, Spencer J Fox, Alison P Galvani, Michael Pignone, S. Claiborne Johnston, Lauren Ancel Meyers. medRxiv 2020.04.16.20068403; doi:<https://doi.org/10.1101/2020.04.16.20068403>

292. Impact of Superspreaders on dissemination and mitigation of COVID-19. Kim Sneppen, Robert J Taylor, Lone Simonsen. medRxiv 2020.05.17.20104745; doi: https://doi.org/10.1101/2020.05.17.20104745

293. Impact of the COVID-19 pandemic on the Internet latency: a large-scale study. [Massimo Candela](https://arxiv.org/search/?searchtype=author&query=Candela%2C+M), [Valerio Luconi](https://arxiv.org/search/?searchtype=author&query=Luconi%2C+V), [Alessio Vecchio](https://arxiv.org/search/?searchtype=author&query=Vecchio%2C+A) [arXiv:2005.06127](https://arxiv.org/abs/2005.06127)

294. Impacts of people's learning behavior in fighting the COVID-19 epidemic. Baolian Cheng, Yi-Ming Wang. medRxiv 2020.08.02.20166967; doi: https://doi.org/10.1101/2020.08.02.20166967

295. Importance of Interaction Structure and Stochasticity for Epidemic Spreading: A COVID-19 Case Study. Gerrit Grossmann, Michael Backenkoehler, Verena Wolf. medRxiv 2020.05.05.20091736; doi:<https://doi.org/10.1101/2020.05.05.20091736>

296. Importance of suppression and mitigation measures in managing COVID-19 outbreaks. Michael E. Hochberg. medRxiv 2020.03.31.20048835; doi:<https://doi.org/10.1101/2020.03.31.20048835>

297. Incubation Period and Reproduction Number for novel coronavirus (COVID-19) infections in India. Seema Patrikar, Atul Kotwal, Vijay Bhatti, Amitav Banerjee, Kunal Chatterjee, Renuka Kunte, Murlidhar Tambe. medRxiv 2020.06.27.20141424; doi:<https://doi.org/10.1101/2020.06.27.20141424>

298. Indian community’s Knowledge, Attitude & Practice towards COVID-19. Balvir Singh Tomar, Pratima Singh, Supriya Suman, Preeti Raj, Deepak Nathiya, Sandeep Tripathi, Dushyant Singh Chauhan. medRxiv 2020.05.05.20092122; doi:<https://doi.org/10.1101/2020.05.05.20092122>

299. [Inferring MHC interacting SARS-CoV-2 epitopes recognized by TCRs towards designing T cell-based vaccines](https://discover.hsls.pitt.edu/vivisimo/cgi-bin/query-meta?v%3afile=viv_vnkHDb&v%3astate=root%7croot-25-25%7c0&url=https%3a%2f%2fdoi.org%2f10.1101%2f2020.09.12.294413&rid=Ndoc26&v%3aframe=redirect&v%3aredirect-hash=c470cff3632df2bcca34da1947c17d9a&). Mohseni AH, Taghinezhad-S S, Su B, Wang F. bioRxiv 2020-09-12. Doi:<https://doi.org/10.1101/2020.09.12.294413>

300. Inferring the effective start dates of non-pharmaceutical interventions during COVID-19 outbreaks. Ilia Kohanovski, Uri Obolski, Yoav Ram. medRxiv 2020.05.24.20092817; doi:<https://doi.org/10.1101/2020.05.24.20092817>

301. Influence of countries adopted policies for COVID-19 reduction under the view of the airborne transmission framework. Charles Roberto Telles. medRxiv 2020.05.20.20107763; doi:<https://doi.org/10.1101/2020.05.20.20107763>

302. Inhomogeneous mixing and asynchronic transmission between local outbreaks account for the spread of COVID-19 epidemics. Carlos I Mendoza. medRxiv 2020.08.04.20168443; doi:<https://doi.org/10.1101/2020.08.04.20168443>

303. Initialization of a Disease Transmission Model.  [Håkan Runvik](https://arxiv.org/search/?searchtype=author&query=Runvik%2C+H), [Alexander Medvedev](https://arxiv.org/search/?searchtype=author&query=Medvedev%2C+A), [Robin Eriksson](https://arxiv.org/search/?searchtype=author&query=Eriksson%2C+R), [Stefan Engblom](https://arxiv.org/search/?searchtype=author&query=Engblom%2C+S). [arXiv:2007.08925](https://arxiv.org/abs/2007.08925)

304. Innate immunity plays a key role in controlling viral load in COVID-19: mechanistic insights from a whole-body infection dynamics model. Prashant Dogra, Javier Ruiz-Ramírez, Kavya Sinha, Joseph D. Butner, Maria J Peláez, Manmeet Rawat, Venkata K. Yellepeddi, Renata Pasqualini, Wadih Arap, H. Dirk Sostman, Vittorio Cristini, Zhihui Wang. medRxiv 2020.10.30.20215335; doi:<https://doi.org/10.1101/2020.10.30.20215335>

305. Insufficient social distancing may be related to COVID-19 outbreak: the case of Ijuí city in Brazil. Thiago Gomes Heck, Rafael Zancan Frantz, Matias Nunes Frizzo, Carlos Henrique Ramires François, Mirna Stela Ludwig, Marilia Arndt Mesenburg, Giovano Pereira Buratti, Lígia Beatriz Bento Franz, Evelise Moraes Berlezi. medRxiv 2020.06.22.20132910; doi:<https://doi.org/10.1101/2020.06.22.20132910>

306. Intervention strategies against COVID-19 and their estimated impact on Swedish healthcare capacity. Jasmine M Gardner, Lander Willem, Wouter Van Der Wijngaart, Shina Caroline Lynn Kamerlin, Nele Brusselaers, Peter Kasson. medRxiv 2020.04.11.20062133; doi: <https://doi.org/10.1101/2020.04.11.20062133>

307.Inverse Problem for Identification of Infectivity and Recovery Rates in SIR Epidemic Models as Functions of Time Illustrated  with Corona Virus Dynamics  up to July 09, 2020. Marinov T, Marinova R. Research Square 2020-07-27. Doi: <https://doi.org/10.21203/rs.3.rs-44704/v1>

308. Investigating duration and intensity of Covid-19 social-distancing strategies. C. Neuwirth, C. Gruber, T Murphy. medRxiv 2020.04.24.20078022; doi:<https://doi.org/10.1101/2020.04.24.20078022>.

309. Is Nigeria really on top of COVID-19? Message from effective reproduction number. A. I. Adekunle, O. A. Adegboye, E. Gayawan, E. S. McBryde. medRxiv 2020.05.16.20104471; doi:<https://doi.org/10.1101/2020.05.16.20104471>

310. Isolation Considered Epidemiological Model for the Prediction of COVID-19 Trend in Tokyo, Japan. Motoaki Utamura, Makoto Koizumi, Seiichi Kirikami. medRxiv 2020.07.31.20165829; doi:<https://doi.org/10.1101/2020.07.31.20165829>

311. Latent Blowout of COVID-19 Globally: An Effort to Healthcare Alertness via Medical GIS Approach. Laxmi Kant Sharma, Rajani Kant Verma. medRxiv 2020.04.27.20082503; doi:<https://doi.org/10.1101/2020.04.27.20082503>

312. Lifestyle acquired immunity, decentralized intelligent infrastructures and revised healthcare expenditures may limit pandemic catastrophe: a lesson from COVID-19. Asif Ahmed, Tasnima Haque, Mohammad Mahmudur Rahman. medRxiv 2020.05.23.20111104; doi:<https://doi.org/10.1101/2020.05.23.20111104>

313. Local protection bubbles: an interpretation of the decrease in the velocity of coronavirus's spread in the city of Sao Paulo. Jose Paulo Guedes Pinto, Patricia Camargo Magalhaes, Gerusa Maria Figueiredo, Domingos Alves, Diana Maritza Segura-Angel. medRxiv 2020.08.11.20173039; doi:<https://doi.org/10.1101/2020.08.11.20173039>

314. Long time frames to detect the impact of changing COVID-19 control measures. Jessica E Stockdale, Renny Doig, Joosung Min, Nicola Mulberry, Liangliang Wang, Lloyd T Elliott, Caroline Colijn. medRxiv 2020.06.14.20131177; doi:<https://doi.org/10.1101/2020.06.14.20131177>

315. Machine learning in predicting respiratory failure in patients with COVID-19 pneumonia - challenges, strengths, and opportunities in a global health emergency. Ferrari Davide, Milic Jovana, Tonelli Roberto, Ghinelli Francesco, Meschiari Marianna, Volpi Sara, Faltoni Matteo, Franceschi Giacomo, Iadisernia Vittorio, Yaacoub Dina, Ciusa Giacomo, Bacca Erica, Rogati Carlotta, Tutone Marco, Burastero Giulia, Raimondi Alessandro, Menozzi Marianna, Franceschini Erica, Cuomo Gianluca, Corradi Luca, Orlando Gabriella, Santoro Antonella, Di Gaetano Margherita, Puzzolante Cinzia, Carli Federica, Bedini Andrea, Fantini Riccardo, Tabbì Luca, Castaniere Ivana, Busani Stefano, Clini Enrico, Girardis Massimo, Sarti Mario, Cossarizza Andrea, Mussini Cristina, Mandreoli Federica, Missier Paolo, Guaraldi Giovanni. medRxiv 2020.05.30.20107888; doi:<https://doi.org/10.1101/2020.05.30.20107888>

316. Magnitude, demographics and dynamics of the impact of the first phase of the Covid-19 pandemic on all-cause mortality in 17 industrialised countries. Vasilis Kontis, James E Bennett, Theo Rashid, Robbie M Parks, Jonathan Pearson-Stuttard, Michel Guillot, Perviz Asaria, Bin Zhou, Marco Battaglini, Gianni Corsetti, Martin McKee, Mariachiara Di Cesare, Colin D Mathers, Majid Ezzati. medRxiv 2020.07.26.20161570; doi:<https://doi.org/10.1101/2020.07.26.20161570>

317. Mathematical framework to model Covid-19 daily deaths. Poulami Barman, Nabarun Deb, Sumit Mukherjee. medRxiv 2020.05.18.20106104; doi:<https://doi.org/10.1101/2020.05.18.20106104>

318. Mathematical model of COVID-19 intervention scenarios for Sao Paulo- Brazil. Osmar Pinto Neto, Josa Clark Reis, Ana Carolina Brisola Brizzi, Gustavo Jose Zambrano, Joabe Marcos de Souza, Wellington Pedroso E. Amorim, Rodrigo Cunha de Mello Pedreiro, Bruno de Matos Brizzi, Ellysson Oliveira Abinader, Deanna M. Kennedy, Renato A Zangaro. medRxiv 2020.04.26.20081208; doi:<https://doi.org/10.1101/2020.04.26.20081208>

319. Mathematical modeling for transmissibility of covid-19 via motorcycles. Benard Okelo. medRxiv 2020.04.18.20070797; doi:<https://doi.org/10.1101/2020.04.18.20070797>

320. Mathematical modeling of the transmission of SARS-CoV-2 ″ Evaluating the impact of isolation in São Paulo State (Brazil) and lockdown in Spain associated with protective measures on the epidemic of covid-19. Hyun Mo Yang, Luis Pedro Lombardi Jr., Fabio Fernandes Morato Castro, Ariana Campos Yang. medRxiv 2020.07.30.20165191; doi:<https://doi.org/10.1101/2020.07.30.20165191>

321. Mathematical modelling of dynamics and containment of COVID-19 in Ukraine. Yuliya N Kyrychko, Konstantin B Blyuss, Igor Brovchenko. medRxiv 2020.07.24.20161497; doi:<https://doi.org/10.1101/2020.07.24.20161497>

322. Medical Capacity Shortages Facilitated the Rapid Dissemination of COVID-19 in Wuhan, New York State, and Italy. Yuehao Xu, Cheng Zhang, Lixian Qian. medRxiv 2020.11.05.20226530; doi:<https://doi.org/10.1101/2020.11.05.20226530>

323. Meta-analysis of several epidemic characteristics of COVID-19. Panpan Zhang, Tiandong Wang, Sharon Xiangwen Xie. medRxiv 2020.05.31.20118448; doi:<https://doi.org/10.1101/2020.05.31.20118448>

324. Metapopulation modeling of COVID-19 advancing into the countryside: an analysis of mitigation strategies for Brazil. Guilherme S. Costa, Wesley Cota, Silvio C. Ferreira. medRxiv 2020.05.06.20093492; doi:<https://doi.org/10.1101/2020.05.06.20093492>

325. Model Based Covid-19 Case Studies in the UK, the USA and India. Santanu Basu. medRxiv 2020.05.31.20118760; doi:<https://doi.org/10.1101/2020.05.31.20118760>

326. Model calibration, nowcasting, and operational prediction of the COVID-19 pandemic. J. D. Annan, J. C. Hargreaves. medRxiv 2020.04.14.20065227; doi:<https://doi.org/10.1101/2020.04.14.20065227>

327. Model studies on the COVID-19 pandemic in Sweden. [Chong Qi](https://arxiv.org/search/?searchtype=author&query=Qi%2C+C), [Daniel Karlsson](https://arxiv.org/search/?searchtype=author&query=Karlsson%2C+D), [Karl Sallmen](https://arxiv.org/search/?searchtype=author&query=Sallmen%2C+K), [Ramon Wyss](https://arxiv.org/search/?searchtype=author&query=Wyss%2C+R). [arXiv:2004.01575](https://arxiv.org/abs/2004.01575)

328. Model-based and model-free characterization of epidemic outbreaks. Jonas Dehning, F. Paul Spitzner, Matthias C. Linden, Sebastian B. Mohr, Joao Pinheiro Neto, Johannes Zierenberg, Michael Wibral, Michael Wilczek, Viola Priesemann. medRxiv 2020.09.16.20187484; doi:<https://doi.org/10.1101/2020.09.16.20187484>

329. Modeling and Forecasting Trend of COVID-19 Epidemic in Iran until May 13, 2020. Ali Ahmadi, Yasin Fadaei, Majid Shirani, Fereydoon Rahmani. medRxiv 2020.03.17.20037671; doi:<https://doi.org/10.1101/2020.03.17.20037671>

330. Modeling and Short-Term Forecasts of Indicators for COVID-19 Outbreak in 25 Countries at the end of March. Handan Ankaralı, Nadire Erarslan, Özge Pasin. medRxiv 2020.04.26.20080754; doi:<https://doi.org/10.1101/2020.04.26.20080754>

331. Modeling Control, Lockdown & Exit Strategies for COVID-19 Pandemic in India. Madhab Barman, Snigdhashree Nayak, Manoj Kumar Yadav, Soumyendu Raha, Nachiketa Mishra. medRxiv 2020.07.25.20161992; doi:<https://doi.org/10.1101/2020.07.25.20161992>

332. Modeling COVID-19 dynamics in Illinois under non-pharmaceutical interventions. George N Wong, Zachary J Weiner, Alexei Tkachenko, Ahmed Elbanna, Sergei Maslov, Nigel Goldenfeld. medRxiv 2020.06.03.20120691; doi:<https://doi.org/10.1101/2020.06.03.20120691>

333. Modeling Exit Strategies from COVID-19 Lockdown with a Focus on Antibody Tests. Reinhard German, Anatoli Djanatliev, Lisa Maile, Peter Bazan, Holger Hackstein. medRxiv 2020.04.14.20063750; doi:<https://doi.org/10.1101/2020.04.14.20063750>

334. Modeling the “Bomb-Like” Dynamics of COVID-19 with Undetected Transmissions and the Implications for Policy. Gary Lin, Anindya Bhaduri, Alexandra T. Strauss, Maxwell Pinz, Diego A. Martinez, Katie K. Tseng, Oliver Gatalo, Andrew T. Gaynor, Efrain Hernandez-Rivera, Emily Schueller, Yupeng Yang, Simon A. Levin, Eili Y. Klein, For the CDC MInD-Healthcare Program. medRxiv 2020.04.05.20054338; doi:<https://doi.org/10.1101/2020.04.05.20054338>

335. Modeling the COVID-19 dissemination in the South Region of Brazil and testing gradual mitigation strategies. Rafael Marques Da Silva. medRxiv 2020.07.02.20145136; doi:<https://doi.org/10.1101/2020.07.02.20145136>

336. Modeling the COVID-19 epidemic in Okinawa. Simone Pigolotti, Davide Chiuchiu, Paula Villa Martin, Deepak Bhat. medRxiv 2020.04.20.20071977; doi:<https://doi.org/10.1101/2020.04.20.20071977>

337. Modeling the COVID-19 outbreaks and the effectiveness of the containment measures adopted across countries. Edward De Brouwer, Daniele Raimondi, Yves Moreau. medRxiv 2020.04.02.20046375; doi:<https://doi.org/10.1101/2020.04.02.20046375>

338. Modeling the Effective Control Strategy for the Transmission Dynamics of Global Pandemic COVID-19. . H. A. Biswas, M. S. Khatun, A. K. Paul, M. R. Khatun, M. A. Islam, S. A. Samad, U. Ghosh. medRxiv 2020.04.22.20076158; doi:<https://doi.org/10.1101/2020.04.22.20076158>

339. Modeling the spread of COVID-19 under active management. Ivan Cherednik. medRxiv 2020.11.20.20235903; doi:<https://doi.org/10.1101/2020.11.20.20235903>

340. [Modeling the Covid-19 Epidemic Using Time Series Econometrics](https://discover.hsls.pitt.edu/vivisimo/cgi-bin/query-meta?v%3afile=viv_vnkHDb&v%3astate=root%7croot-50-25%7c0&url=https%3a%2f%2fdoi.org%2f10.1101%2f2020.06.01.20118612&rid=Ndoc53&v%3aframe=redirect&v%3aredirect-hash=087efdcf87527a1f5f7fa611026adef9&). Goliński A, Spencer P. me2020-06-02. Doi:<https://doi.org/10.1101/2020.06.01.20118612>

341. Modelling COVID-19 contagion: Risk assessment and targeted mitigation policies. Rama Cont. Artur Kotlicki. Renyuan Xu. medRxiv 2020.08.26.20182477;<https://doi.org/10.1101/2020.08.26.20182477>

342. Modelling COVID-19 contagion: Risk assessment and targeted mitigation policies. Rama Cont, RenYuan Xu, Artur Kotlicki. medRxiv 2020.08.26.20182477; doi:<https://doi.org/10.1101/2020.08.26.20182477>

343. Modelling of covid-19 outbreak indicators in china between january and April. Senol Çelik, Handan Ankarali, Ozge Pasin. medRxiv 2020.04.26.20080465; doi:<https://doi.org/10.1101/2020.04.26.20080465>

344. Modelling Singapore COVID-19 pandemic with a SEIR multiplex network model. Ning Ning Chung, Lock Yue Chew. medRxiv 2020.05.31.20118372; doi:<https://doi.org/10.1101/2020.05.31.20118372>

345. Modelling the first wave of the COVID-19 epidemic in the Czech Republic and the role of government interventions. Ondrej Majek, Ondrej Ngo, Jiri Jarkovsky, Martin Komenda, Jarmila Razova, Ladislav Dusek, Tomas Pavlik. medRxiv 2020.09.10.20192070; doi:<https://doi.org/10.1101/2020.09.10.20192070>

346. Modelling the impact of lockdown easing measures on cumulative COVID-19 cases and deaths in England. Hisham Ziauddeen, Naresh Subramaniam, Deepti Gurdasani. medRxiv 2020.06.21.20136853; doi:<https://doi.org/10.1101/2020.06.21.20136853>

347. Modelling the Impact of Nationwide BCG Vaccine Recommendations on COVID-19 Transmission, Severity, and Mortality. Nita H. Shah, Ankush H. Suthar, Moksha H. Satia, Yash Shah, Nehal Shukla, Jagdish Shukla, Dhairya Shukla. medRxiv 2020.05.10.20097121; doi:<https://doi.org/10.1101/2020.05.10.20097121>

348. Modelling the Occurrence of the Novel Pandemic COVID-19 Outbreak; A Box and Jenkins Approach. Nurudeen Ayobami Ajadi, Isqeel Adesegun Ogunsola, Saddam Adams Damisa. medRxiv 2020.06.15.20131136; doi:<https://doi.org/10.1101/2020.06.15.20131136>

349. Modelling the potential impact of social distancing on the COVID-19 epidemic in South Africa. F. Nyabadza, F. Chirove, W. Chukwu, M.V. Visaya. medRxiv 2020.04.21.20074492; doi:<https://doi.org/10.1101/2020.04.21.20074492>

350. Modelling to Predict Hospital Bed Requirements for Covid-19 Patients in California. Santanu Basu. medRxiv 2020.05.17.20104919; doi:<https://doi.org/10.1101/2020.05.17.20104919>

351. Momentum managing epidemic spread and Bessel functions. [Ivan Cherednik](https://arxiv.org/search/?searchtype=author&query=Cherednik%2C+I). [arXiv:2004.06021](https://arxiv.org/abs/2004.06021)

352. Monitoring COVID-19 progression: Look at Us Today, See Yourself Tomorrow. Jungsik Noh. medRxiv 2020.04.20.20072991; doi:<https://doi.org/10.1101/2020.04.20.20072991>

353. Monitoring Italian COVID-19 spread by an adaptive SEIRD model. Elena Loli Piccolomini, Fabiana Zama. medRxiv 2020.04.03.20049734; doi:<https://doi.org/10.1101/2020.04.03.20049734>

354. Monitoring life expectancy levels during the COVID-19 pandemic: Example of the unequal impact in Spanish regions. Sergi Trias-Llimos, Tim Riffe, Usama Bilal. medRxiv 2020.06.03.20120972; doi:<https://doi.org/10.1101/2020.06.03.20120972>

355. Monitoring the propagation of COVID-19-pandemic first waves. William Knafo. medRxiv 2020.05.09.20096768; doi:<https://doi.org/10.1101/2020.05.09.20096768>

356. More prevalent, less deadly? Bayesian inference of the COVID19 Infection Fatality Ratio from mortality data. G. W. Delius, B. J. Powell, M. A. Bees, G. W. A. Constable, N. J. MacKay, J. W. Pitchford. medRxiv 2020.04.19.20071811; doi:<https://doi.org/10.1101/2020.04.19.20071811>

357. Morphology and numerical characteristics of epidemic curves for SARS-Cov-II using Moyal distribution. [Jose de Jesus Bernal-Alvarado](https://arxiv.org/search/?searchtype=author&query=Bernal-Alvarado%2C+J+d+J), [David Delepine](https://arxiv.org/search/?searchtype=author&query=Delepine%2C+D). [arXiv:2006.04954](https://arxiv.org/abs/2006.04954)

358. Mortality from COVID in Colombia and Peru: Analyses of Mortality Data and Statistical Forecasts. Patrick E Brown. Zoë R Greenwald. Luis Ernesto Salinas. Gabriel Aguirre Martens. Leslie New‐combe. Peter S Rodriguez. Hellen Gelband. Jeremy Veillard. Prabhat Jha. medRxiv 2020.08.24.20181016; doi:<https://doi.org/10.1101/2020.08.24.20181016>

359. Mortality of the COVID-19 outbreak in Sweden in relation to previous severe disease outbreaks. Anders Ledberg. medRxiv 2020.05.22.20110320; doi:<https://doi.org/10.1101/2020.05.22.20110320>

360. Moving-average based index to timely evaluate the current epidemic situation after COVID-19 outbreak. He Yun-ting, Wang Xiao-jin, He Hao, Zhai Jing, Wang Bing-shun. medRxiv 2020.03.24.20027730; doi:<https://doi.org/10.1101/2020.03.24.20027730>

361. Multiscale dynamics of COVID-19 and model-based recommendations for 105 countries. Jithender J. Timothy, Vijaya Holla, Guenther Meschke. medRxiv 2020.06.05.20123547; doi:<https://doi.org/10.1101/2020.06.05.20123547>

362. Navigating hospitals safely through the COVID-19 epidemic tide: predicting case load for adjusting bed capacity. Tjibbe Donker, Fabian Bürkin, Martin Wolkewitz, Christian Haverkamp, Dominic Christoffel, Oliver Kappert, Thorsten Hammer, Hans-Jörg Busch, Paul Biever, Johannes Kalbhenn, Hartmut Bürkle, Winfried Kern, Frederik Wenz, Hajo Grundmann. medRxiv 2020.07.02.20143206; doi:<https://doi.org/10.1101/2020.07.02.20143206>

363. Nearly Perfect Forecasting of the Total COVID-19 Cases in India: A Numerical Approach. Hemanta Kumar Baruah. medRxiv 2020.06.13.20130096; doi:<https://doi.org/10.1101/2020.06.13.20130096>

364. Non-COVID-19 Deaths After Social Distancing in Norway. Ralph Catalano, Joan A Casey, Tim-Allen Bruckner, Alison Gemmill. medRxiv 2020.06.05.20123695; doi:<https://doi.org/10.1101/2020.06.05.20123695>

365. Non-COVID-19 deaths in the United States during the imposition of sheltering-in-place. Ralph Catalano, Joan A. Casey, Alison Gemmill, and Tim A. Bruckne. medRxiv 2020.07.26.20162396; doi:<https://doi.org/10.1101/2020.07.26.20162396>

366. Non-COVID-19 deaths in the United States during the imposition of sheltering-in-place. Ralph Catalano, Joan A. Casey, Alison Gemmill, Tim-Allen Bruckner. medRxiv 2020.07.26.20162396; doi:<https://doi.org/10.1101/2020.07.26.20162396>

367. Non-stationary Spatio-Temporal Modeling of COVID-19 Progression in The U.S. Yue Bai, Abolfazl Safikhani, George Michailidis. medRxiv 2020.09.14.20194548; doi:<https://doi.org/10.1101/2020.09.14.20194548>

368. Nowcasting and Forecasting the Spread of COVID-19 and Healthcare Demand In Turkey, A Modelling Study. Seyma Arslan, Muhammed Yusuf Ozdemir, Abdullah Ucar. medRxiv 2020.04.13.20063305; doi:<https://doi.org/10.1101/2020.04.13.20063305>

369. Nowcasting and Forecasting the Spread of COVID-19 in Iran. Hamidreza Masjedi, Jomar F. Rabajante, Fatemeh Bahranizadd, Mohammad Hosein Zare. medRxiv 2020.04.22.20076281; doi:<https://doi.org/10.1101/2020.04.22.20076281>

370. Nowcasting CoVID-19 Deaths in England by Age and Region. Shaun Seaman, Pantelis Samartsidis, Meaghan Kall, Daniela De Angelis. medRxiv 2020.09.15.20194209; doi:<https://doi.org/10.1101/2020.09.15.20194209>

371. Nowcasting Covid-19 statistics reported withdelay: a case-study of Sweden. [Adam Altmejd](https://arxiv.org/search/?searchtype=author&query=Altmejd%2C+A), [Joacim Rocklöv](https://arxiv.org/search/?searchtype=author&query=Rockl%C3%B6v%2C+J), [Jonas Wallin](https://arxiv.org/search/?searchtype=author&query=Wallin%2C+J). [arXiv:2006.06840](https://arxiv.org/abs/2006.06840)

372. Now-casting the COVID-19 epidemic: The use case of Japan, March 2020. Stephan Glöckner, Gérard Krause, Michael Höhle. medRxiv 2020.03.18.20037473; doi:<https://doi.org/10.1101/2020.03.18.20037473>

373. Nowcasting the COVID-19 Pandemic in Bavaria. Felix Guenther, Andreas Bender, Katharina Katz, Helmut Kuechenhoff, Michael Hoehle. medRxiv 2020.06.26.20140210; doi: [https://doi.org/10.1101/2020.06.26.20140210](https://www.socialstyrelsen.se/globalassets/1-globalt/covid-19-statistik/statistik-om-slutenvard-av-patienter-med-covid-19/statistik-covid19-inskrivna.xlsx?web=1)

374. On nonlinear incidence rate of Covid-19. Swarna Kamal Paul, Saikat Jana, Parama Bhaumik medRxiv 2020.10.19.20215665; doi:<https://doi.org/10.1101/2020.10.19.20215665>

375. On Reliability of the COVID-19 Forecasts. Hemanta Kumar Baruah. medRxiv 2020.06.01.20118844; doi:<https://doi.org/10.1101/2020.06.01.20118844>

376. On short-term trends and predictions for COVID-19 in France and the USA: comparison with Australia. Henry C. Tuckwell, Mohsen Dorraki, Stephen J. Salamon, Andrew Allison, Derek Abbott. medRxiv 2020.11.17.20233718; doi:<https://doi.org/10.1101/2020.11.17.20233718>

377. On the heterogeneity of infections, containment measures and the preliminary forecast of COVID-19 epidemic. Ichiro Nakamoto, Weiqing Zhuang, Sheng Wang, Yan Guo. medRxiv 2020.05.12.20096792; doi:<https://doi.org/10.1101/2020.05.12.20096792>

378. On the reliability of predictions on Covid-19 dynamics: a systematic and critical review of modelling techniques. Janyce E. Gnanvi, Val\`ere K. Salako, Brezesky Kotanmi, Romain Gl`el`e Kakai. medRxiv 2020.09.10.20192328; doi:<https://doi.org/10.1101/2020.09.10.20192328>

379. On the secondary waves of the pandemic launched in Iran and other countries. Dmitry Kovriguine, Svetlana Nikitenkova. medRxiv 2020.06.20.20136283; doi:<https://doi.org/10.1101/2020.06.20.20136283>

380. On the Temporal Analysis of COVID-19 Pandemic and Prediction of R 0. Patil K, Murali A, Ganguli P, Nandi S, Sarkar R. SSRN2020-09-23. Doi: <https://doi.org/10.2139/ssrn.3633240>

381. On the temporal spreading of the SARS-CoV-2. Francesca Bertacchini, Eleonora Bilotta, Pietro S. Pantano. medRxiv 2020.08.01.20166447; doi:<https://doi.org/10.1101/2020.08.01.20166447>

382. On Topological Properties of COVID-19: Predicting and Controling Pandemic Risk with Network Statistics. Mike K.P. So, Amanda M.Y. Chu, Agnes Tiwari, Jacky N.L. Chan. medRxiv 2020.09.17.20197020; doi:<https://doi.org/10.1101/2020.09.17.20197020>

383. [One Million or One Hundred Million Casualties? – The Impact of the COVID-19 Crisis on the Least Developed and Developing countries](https://discover.hsls.pitt.edu/vivisimo/cgi-bin/query-meta?v%3afile=viv_vnkHDb&v%3astate=root%7croot-50-25%7c0&url=https%3a%2f%2fdoi.org%2f10.2139%2fssrn.3597657&rid=Ndoc71&v%3aframe=redirect&v%3aredirect-hash=8c68fdb06746b5b2232f0a2e16828478&). Zetzsche DA, Consiglio R. SSRN 2020-05-10. Doi:<https://doi.org/10.2139/ssrn.3597657>

384. Ongoing outbreak of COVID-19 in Iran: challenges and signs of concern with under-reporting of prevalence and deaths. Mahan Ghafari, Bardia Hejazi, Arman Karshenas, Stefan Dascalu, Alireza Kadivar, Mohammad Ali Khosravi, Maryam Abbasalipour, Majid Heydari, Sirous Zeinali, Luca Ferretti, Alice Ledda, Aris Katzourakis. medRxiv 2020.04.18.20070904; doi:<https://doi.org/10.1101/2020.04.18.20070904>

385. Online analysis of epidemics with variable infection rate. [Yurii Nesterov](https://arxiv.org/search/?searchtype=author&query=Nesterov%2C+Y) [arXiv:2007.11429](https://arxiv.org/abs/2007.11429)

386. Onset of effects of non-pharmaceutical interventions on COVID-19 worldwide. Elisabeth Lucia Zeilinger, Ingo W. Nader, Dana Jomar, Clemens Zauchner. medRxiv 2020.09.02.20185660; doi: https://doi.org/10.1101/2020.09.02.20185660

387. Optimal test-assisted quarantine strategies for COVID-19. Bo Peng, Wen Zhou, Rowland W. Pettit, Patrick Yu, Peter G. Matos, Alexander L. Greninger, Julie McCashin, Christopher I. Amos. medRxiv 2020.11.06.20222398; doi:<https://doi.org/10.1101/2020.11.06.20222398>

388. Optimising social mixing strategies achieving COVID-19 herd immunity while minimising mortality in six European countries. Romain Ragonnet, Guillaume Briffoteaux, Bridget M. Williams, Julian Savulescu, Matthew Segal, Milinda Abayawardana, Rosalind M. Eggo, Daniel Tuyttens, Nouredine Melab, Ben J. Marais, Emma S. McBryde, James M. Trauer. medRxiv 2020.08.25.20182162; doi:<https://doi.org/10.1101/2020.08.25.20182162>

389. Oscillatory dynamics in infectivity and death rates of COVID-19. Tomáš Pavlíček, Pavel Rehak, Petr Král. medRxiv 2020.05.19.20107474; doi: https://doi.org/10.1101/2020.05.19.20107474

390. Outbreak analysis with a logistic growth model shows COVID-19 suppression dynamics in China. Yi Zou, Stephen Pan, Peng Zhao, Lei Han, Xiaoxiang Wang, Lia Hemerik, Johannes Knops, Wopke van der Werf. medRxiv 2020.03.25.20043539; doi:<https://doi.org/10.1101/2020.03.25.20043539>

391. Outbreak of Covid-19 worldwide is on the decline -----Recurrent Neural Reinforcement Learning and Health Interventions to Curb the Spread of Covid-19 in the world. Qiyang Ge, Zixin Hu, Kai Zhang, Shudi Li, Wei Lin, Li Jin, Momiao Xiong. medRxiv 2020.07.08.20149146; doi: https://doi.org/10.1101/2020.07.08.20149146

392. Outbreaks of publications about emerging infectious diseases: the case of SARS-CoV-2 and Zika virus. Aziz Mert Ipekci, Diana Buitrago-Garcia, Kaspar Walter Meili, Fabienne Krauer, Nirmala Prajapati, Shabnam Thapa, Lea Wildisen, Lucia Araujo Chaveron, Lukas Baumann, Sanam Shah, Tessa Whiteley, Gonzalo Solís-García, Foteini Tsotra, Ivan Zhelyazkov, Hira Imeri, Nicola Low, Michel Jacques Counotte. medRxiv 2020.11.20.20235242; doi:<https://doi.org/10.1101/2020.11.20.20235242>

393. Outdoor PM2.5 Concentration and Rate of Change in COVID-19 Infection in 31 Provincial Capital Cities in China. Yang Han, Jacqueline C.K. Lam, Victor O.K. Li, Jon Crowcroft, Peiyang Guo, Jinqi Fu, Qi Zhang, Andong Wang, Shanshan Wang, Illana Gozes, Zafar Gilani. medRxiv 2020.05.19.20106484; doi:<https://doi.org/10.1101/2020.05.19.20106484>

394. Pandemic, Shutdown and Consumer Spending: Lessons from Scandinavian Policy Responses to COVID-19. [Asger Lau Andersen](https://arxiv.org/search/?searchtype=author&query=Andersen%2C+A+L), [Emil Toft Hansen](https://arxiv.org/search/?searchtype=author&query=Hansen%2C+E+T), [Niels Johannesen](https://arxiv.org/search/?searchtype=author&query=Johannesen%2C+N), [Adam Sheridan](https://arxiv.org/search/?searchtype=author&query=Sheridan%2C+A). [arXiv:2005.04630](https://arxiv.org/abs/2005.04630)

395. Pathways of the COVID-19 Pandemic with Human Mobility across Countries. Cheng Zhang, Li-Xian Qian, Jian-Qiang Hu. medRxiv 2020.05.21.20108589; doi:<https://doi.org/10.1101/2020.05.21.20108589>

396. Patterns of the COVID19 epidemic spread around the world: exponential vs power laws. Natalia L. Komarova, Luis M. Schang, Dominik Wodarz. medRxiv 2020.03.30.20047274; doi:<https://doi.org/10.1101/2020.03.30.20047274>

397. Performance of progressive and adaptive COVID-19 exit strategies: a stress test analysis for managing intensive care unit rates. Jan-Diederik van Wees, Martijn van der Kuip, Sander Osinga, David van Westerloo, Michael Tanck, Maurice Hanegraaf, Maarten Pluymaekers, Olwijn Leeuwenburgh, Lonneke van Bijsterveldt, Pien Verreijdt, Logan Brunner, Marceline Tutu van Furth. medRxiv 2020.05.16.20102947; doi:<https://doi.org/10.1101/2020.05.16.20102947>

398. Poorly known aspects of flattening the curve of COVID-19. Alain Debecker, Theodore Modis. medRxiv 2020.06.09.20126128; doi:<https://doi.org/10.1101/2020.06.09.20126128>

399. Population vulnerability to COVID-19 in Europe: a burden of disease analysis. Grant MA Wyper, Ricardo MA Assunção, Sarah Cuschieri, Brecht Devleeschauwer, Eilidh Fletcher, Juanita A Haagsma, Henk Hilderink, Jane Idavain, Tina Lesnik, Elena Von der Lippe, Marek Majdan, Milena S Milicevic, Elena Pallari, José L Peñalvo, Sara M Pires, Dietrich Plaß, João V Santos, Diane L Stockton, Sofie T Thomsen, Ian Grant. medRxiv 2020.04.29.20064279; doi:<https://doi.org/10.1101/2020.04.29.20064279>

400. Positive rates predict death rates of Covid-19 locally and worldwide 13 days ahead. Jürgen Mimkes, Rainer Janssen. medRxiv 2020.11.24.20237842; doi:<https://doi.org/10.1101/2020.11.24.20237842>

401. Predicted COVID-19 fatality rates based on age, sex, comorbidities, and health system capacity. Selene Ghisolfi, Ingvild Ingvild Almas, Justin Sandefur, Tillman von Carnap, Jesse Heitner, Tessa Bold. medRxiv 2020.06.05.20123489; doi:<https://doi.org/10.1101/2020.06.05.20123489>

402. Predicting Onset of COVID-19 with Mobility-Augmented SEIR Model. Neo Wu, Xue Ben, Bradley Green, Kathryn Rough, Srinivasan Venkatramanan, Madhav Marathe, Paul Eastham, Adam Sadilek, Shawn O’Banion. medRxiv 2020.07.27.20159996; doi:<https://doi.org/10.1101/2020.07.27.20159996>

403. Predicting the cumulative medical load of COVID-19 outbreaks after the peak in daily fatalities. Claudius Gros, Roser Valenti, Lukas Schneider, Benedikt Gutsche, Dimitrije Markovic. medRxiv 2020.09.03.20183384; doi:<https://doi.org/10.1101/2020.09.03.20183384>

404. Predicting the Growth and Trend of COVID-19 Pandemic using Machine Learning and Cloud. Computing. Shreshth Tuli, Shikhar Tuli, Rakesh Tuli, Sukhpal Singh Gill. medRxiv 2020.05.06.20091900; doi:<https://doi.org/10.1101/2020.05.06.20091900>

405. Predicting the Trajectory of Any COVID19 Epidemic From the Best Straight Line. Michael Levitt, Andrea Scaiewicz, Francesco Zonta. medRxiv 2020.06.26.20140814; doi:<https://doi.org/10.1101/2020.06.26.20140814>

406. Prediction of Covid-19 Infections Through December 2020 for 10 US States Incorporating Outdoor Temperature and School Re-Opening Effects-August Update. Ty A Newell. medRxiv 2020.09.14.20193821; doi:<https://doi.org/10.1101/2020.09.14.20193821>

407. Prediction of Peak and Termination of Novel Coronavirus Covid-19 Epidemic in Iran. Amir-Pouyan Zahiri, Sepehr RafieeNasab, Ehsan Roohi. medRxiv 2020.03.29.20046532; doi:<https://doi.org/10.1101/2020.03.29.20046532>

408. Prediction of the COVID-19 Epidemic Trends Based on SEIR and AI Models. Shuo Feng, Zebang Feng, Chen Ling, Chen Chang, Zhongke Feng. medRxiv 2020.04.21.20074138; doi:<https://doi.org/10.1101/2020.04.21.20074138>

409. Prediction on Covid-19 epidemic for different countries: Focusing on South Asia under various precautionary measures. Abhijit Paul, Samrat Chatterjee, Nandadulal Bairagi. medRxiv 2020.04.08.20055095; doi:<https://doi.org/10.1101/2020.04.08.20055095>

410. [Prediction of curve flattening time for COVID-19 infected countries using trends from recovered countries](https://discover.hsls.pitt.edu/vivisimo/cgi-bin/query-meta?v%3afile=viv_vnkHDb&v%3astate=root%7croot-25-25%7c0&url=https%3a%2f%2fdoi.org%2f10.21203%2frs.3.rs-30358%2fv1&rid=Ndoc41&v%3aframe=redirect&v%3aredirect-hash=4df69859db6479a9e9d3764447eea4bb&). Kumar S. Research Square 2020-06-19. Doi:<https://doi.org/10.21203/rs.3.rs-30358/v1>

411. [Predictions for Europe for the Covid-19 pandemic from a SIR model](https://discover.hsls.pitt.edu/vivisimo/cgi-bin/query-meta?v%3afile=viv_vnkHDb&v%3astate=root%7croot-50-25%7c0&url=https%3a%2f%2fdoi.org%2f10.1101%2f2020.05.26.20114058&rid=Ndoc57&v%3aframe=redirect&v%3aredirect-hash=a658bf07b2541fc57e66c6fe7c14e51c&). Bhanot G, DeLisi C. medRxiv 2020-05-28. Doi:<https://doi.org/10.1101/2020.05.26.20114058>

412. Predictive accuracy of a hierarchical logistic model of cumulative SARS-CoV-2 case growth. Levente Kriston. medRxiv 2020.06.15.20130989; doi:<https://doi.org/10.1101/2020.06.15.20130989>.

413. Predictive Analysis for COVID-19 Spread in India by Adaptive Compartmental Model. Sudhansu Sekhar Singh, Dinakrushna Mohapatra. medRxiv 2020.07.08.20148619; doi:<https://doi.org/10.1101/2020.07.08.20148619>

414. Presence of SARS-CoV-2-reactive T cells in COVID-19 patients and healthy donors. Julian Braun, Lucie Loyal, Marco Frentsch, Daniel Wendisch, Philipp Georg, Florian Kurth, Stefan Hippenstiel, Manuela Dingeldey, Beate Kruse, Florent Fauchere, Emre Baysal, Maike Mangold, Larissa Henze, Roland Lauster, Marcus A. Mall, Kirsten Beyer, Jobst Röhmel, Jürgen Schmitz, Stefan Miltenyi, Ilja Demuth, Marcel A. Müller, Martin Witzenrath, Norbert Suttorp, Florian Kern, Ulf Reimer, Holger Wenschuh, Christian Drosten, Victor M. Corman, Claudia Giesecke-Thiel, Leif Erik Sander, Andreas Thiel. medRxiv 2020.04.17.20061440; doi:<https://doi.org/10.1101/2020.04.17.20061440>

415. Progression of COVID-19 in Indian States - Forecasting Endpoints Using SIR and Logistic Growth Models. Bhoomika Malhotra, Vishesh Kashyap. medRxiv 2020.05.15.20103028; doi:<https://doi.org/10.1101/2020.05.15.20103028>

416. Projected ICU and Mortuary load due to COVID-19 in Sydney. Andrew Francis, Yi Guo, Paul Hurley, Oliver Obst, Laurence Park, Mark Tanaka, Russell Thomson, X. Rosalind Wang. medRxiv 2020.03.31.20049312; doi:<https://doi.org/10.1101/2020.03.31.20049312>

417. Projecting contact matrices in 177 geographical regions: an update and comparison with empirical data for the COVID-19 era. Kiesha Prem, Kevin van Zandvoort, Petra Klepac, Rosalind M Eggo, Nicholas G Davies, Centre for the Mathematical Modelling of Infectious Diseases COVID-19 Working Group, Alex R Cook, Mark Jit. medRxiv 2020.07.22.20159772; doi:<https://doi.org/10.1101/2020.07.22.20159772>

418. Projecting the impact of a two-dose COVID-19 vaccination campaign in Ontario, Canada. Thomas N. Vilches, Kevin Zhang, Robert Van Exan, Joanne M. Langley, Seyed M. Moghadas. medRxiv 2020.12.10.20246827; doi:<https://doi.org/10.1101/2020.12.10.20246827>

419. Protocol of a population-based prospective COVID-19 cohort study Munich, Germany (KoCo19). Katja Radon, Elmar Saathoff, Michael Pritsch, Jessica Michelle Guggenbühl Noller, Inge Kroidl, Laura Olbrich, Verena Thiel, Max Diefenbach, Friedrich Riess, Felix Forster, Fabian Theis, Andreas Wieser, Michael Hoelscher, the KoCo19 collaboration group#. medRxiv 2020.04.28.20082743; doi:<https://doi.org/10.1101/2020.04.28.20082743>

420. Public policy and economic dynamics of COVID-19 spread: a mathematical modeling study. Uri Goldsztejn, David Schwartzman, Arye Nehorai. medRxiv 2020.04.13.20062802; doi:<https://doi.org/10.1101/2020.04.13.20062802>

421. Quantifying early COVID-19 outbreak transmission in South Africa and exploring vaccine efficacy scenarios. Z. Mukandavire, F. Nyabadza, N. J. Malunguza, D. F. Cuadros, T. Shiri, G. Musuka. medRxiv 2020.04.23.20077297; doi:<https://doi.org/10.1101/2020.04.23.20077297>

422. Quantifying the effect of quarantine control in Covid-19 infectious spread using machine learning. Raj Dandekar, George Barbastathis. medRxiv 2020.04.03.20052084; doi:<https://doi.org/10.1101/2020.04.03.20052084>

423. Quantitative COVID-19 infectiousness estimate correlating with viral shedding and culturability suggests 68% pre-symptomatic transmissions. Meher K. Prakash. medRxiv 2020.05.07.20094789; doi:<https://doi.org/10.1101/2020.05.07.20094789>

424. Rapid assessment of the impact of “lockdown” on the COVID-19 epidemic in Portugal. Vasco Ricoca Peixoto, André Vieira, Pedro Aguiar, Carlos Carvalho, Daniel Thomas, Alexandre Abrantes, Public Health Research Center, National School of Public Health, Portugal. medRxiv 2020.05.26.20098244; doi: https://doi.org/10.1101/2020.05.26.20098244

425. Rapid disappearance of influenza following the implementation of COVID-19 mitigation measures in Hamilton, Ontario. Kevin Zhang, Avika Misra, Patrick J. Kim, Seyed M. Moghadas, Joanne M. Langley, Marek Smieja. medRxiv 2020.11.27.20240036; doi:<https://doi.org/10.1101/2020.11.27.20240036>

426. Rapid estimation of excess mortality in times of COVID-19 in Portugal Beyond reported deaths. André Vieira, Vasco Peixoto Ricoca, Pedro Aguiar, Alexandre Abrantes. medRxiv 2020.05.14.20100909; doi:<https://doi.org/10.1101/2020.05.14.20100909>

427. Real-time forecasts and risk assessment of novel coronavirus (COVID-19) cases: A data-driven analysis. Tanujit Chakraborty, Indrajit Ghosh. medRxiv 2020.04.09.20059311; doi:<https://doi.org/10.1101/2020.04.09.20059311>

428. Real-time monitoring of COVID-19 dynamics using automated trend fitting and anomaly detection. Thibaut Jombart, Stephane Ghozzi, Dirk Schumacher, Quentin Leclerc, Mark Jit, Stefan Flasche, Felix Greaves, Tom Ward, Rosalind M Eggo, Emily Nightingale, Sophie Meakin, Oliver J Brady, Centre for Mathematical Modelling of Infectious Diseases COVID-19 Working Group, Graham Medley, Michael Hohle, John Edmunds. medRxiv 2020.09.02.20186502; doi: https://doi.org/10.1101/2020.09.02.20186502

429. Real-time monitoring the transmission potential of COVID-19 in Singapore, March 2020. Amna Tariq, Yiseul Lee, Kimberlyn Roosa, Seth Blumberg, Ping Yan, Stefan Ma, Gerardo Chowell. medRxiv 2020.02.21.20026435; doi:<https://doi.org/10.1101/2020.02.21.20026435>

430. Real-time Nowcasting and Forecasting of COVID-19 Dynamics in England: the first wave? Paul J Birrell, Joshua Blake, Edwin van Leeuwen, PHE Joint Modelling Cell, Nick Gent, Daniela De Angelis. medRxiv 2020.08.24.20180737; doi:<https://doi.org/10.1101/2020.08.24.20180737>

431. Real-time time-series modelling for prediction of COVID-19 spread and intervention assessment. Taha Hossein Rashidi, Siroos Shahriari, AKM Azad, Fatemeh Vafaee. medRxiv 2020.04.24.20078923; doi:<https://doi.org/10.1101/2020.04.24.20078923>

432. Real-time tracking and prediction of COVID-19 infection using digital proxies of population mobility and mixing. Kathy Leung, Joseph T Wu, Gabriel M Leung. medRxiv 2020.10.17.20214155; doi:<https://doi.org/10.1101/2020.10.17.20214155>

433. [REDIAL-2020: A Suite of Machine Learning Models to Estimate Anti-SARS-CoV-2 Activities](https://discover.hsls.pitt.edu/vivisimo/cgi-bin/query-meta?v%3afile=viv_vnkHDb&v%3astate=root%7croot-25-25%7c0&url=https%3a%2f%2fdoi.org%2f10.26434%2fchemrxiv.12915779.v2&rid=Ndoc25&v%3aframe=redirect&v%3aredirect-hash=0a9c8842b71701e6acb0175a46345c3c&). KC G, Bocci G, Verma S, Hassan M, Holmes J, Yang J, sirimulla s, Oprea T. ChemRxiv 2020-09-16. Doi:<https://doi.org/10.26434/chemrxiv.12915779.v2>

434. Reducing SARS-CoV-2 infectious spreading patterns by removing S and R compartments from SIR model equation. Charles Roberto Telles. medRxiv 2020.06.12.20127498; doi:<https://doi.org/10.1101/2020.06.12.20127498>

435. Relaxing lockdown measures in epidemic outbreaks using selective socio-economic containment with uncertainty. Giacomo Albi, Lorenzo Pareschi, Mattia Zanella. medRxiv 2020.05.12.20099721; doi:<https://doi.org/10.1101/2020.05.12.20099721>

436. Releasing the lockdown in the UK Covid-19 epidemic: a stochastic model. Anthony Lander. medRxiv 2020.04.28.20083329; doi:<https://doi.org/10.1101/2020.04.28.20083329>

437. Reproductive number of COVID-19: A systematic review and meta-analysis based on global level evidence. Md. Arif Billah, Md. Mamun Miah, Md. Nuruzzaman Khan. medRxiv 2020.05.23.20111021; doi:<https://doi.org/10.1101/2020.05.23.20111021>

438. Retarded logistic equation as a universal dynamic model for the spread of COVID-19. B Shayak, Mohit M Sharma. medRxiv 2020.06.09.20126573; doi:<https://doi.org/10.1101/2020.06.09.20126573>

439. Revealing the extent of the COVID-19 pandemic in Kenya based on serological and PCR-test data. John Ojal, Samuel PC Brand, Vincent Were, Emelda A Okiro, Ivy Kadzo Kombe, Caroline Mburu, Rabia Aziza, Morris Ogero, Ambrose Agweyu, George M Warimwe, Sophie Uyoga, Ifedayo M. O Adetifa, John Anthony Scott, Edward Otieno, Lynette I Ochola-Oyier, Charles Nyaigoti Agoti, Kadondi Kasera, Patrick Amoth, Mercy Mwangangi, Rashid Aman, Wangari Ng'ang'a, Benjamin Tsofa, Philip Bejon, Edwine Barasa, Matt J Keeling, D James Nokes. medRxiv 2020.09.02.20186817; doi:<https://doi.org/10.1101/2020.09.02.20186817>

440. Risk Assessment of Novel Coronavirus COVID-19 Outbreaks Outside China. Péter Boldog, Tamás Tekeli, Zsolt Vizi, Attila Dénes, Ferenc A. Bartha, Gergely Röst. medRxiv 2020.02.04.20020503; doi:<https://doi.org/10.1101/2020.02.04.20020503>

441. Risk Factors for ICU Admission, Mechanical Ventilation and Mortality in Hospitalized Patients with COVID-19 in Hubei, China. Hong Gang Ren, Xingyi Guo, Kevin Blighe, Fang Zhu, Janet Martin, Luqman Bin Safdar, Pengcheng Yang, Dao Wen Wang, Qinyong Hu, Nan Huo, Justin Stebbing, Davy Cheng. medRxiv 2020.08.31.20184952; doi:<https://doi.org/10.1101/2020.08.31.20184952>

442. Robot dance: a city-wise automatic control of Covid-19 mitigation levels. Paulo J. S. Silva, Tiago Pereira, Luis Gustavo Nonato. medRxiv 2020.05.11.20098541; doi:<https://doi.org/10.1101/2020.05.11.20098541>

443. Role of high-dose exposure in transmission hot zones as a driver of SARS-CoV2 dynamics. Dominik Wodarz, Natalia L. Komarova, Luis M. Schang. medRxiv 2020.10.07.20208231; doi:<https://doi.org/10.1101/2020.10.07.20208231>

444. [SARS-Cov-2 trajectory predictions and scenario simulations from a global perspective: a modelling study](https://discover.hsls.pitt.edu/vivisimo/cgi-bin/query-meta?v%3afile=viv_vnkHDb&v%3astate=root%7croot-25-25%7c0&url=https%3a%2f%2fdoi.org%2f10.21203%2frs.3.rs-36239%2fv1&rid=Ndoc43&v%3aframe=redirect&v%3aredirect-hash=68743533592ed5e269855e72482285cd&). Yang T, Liu Y, Deng W, Zhao W, Deng J. Research Square 2020-06-18. Doi:<https://doi.org/10.21203/rs.3.rs-36239/v1>

445. SCOAT-Net: A Novel Network for Segmenting COVID-19 Lung Opacification from CT Images. Shixuan Zhao, Zhidan Li, Yang Chen, Wei Zhao, Xingzhi Xie, Jun Liu, Di Zhao, Yongjie Li. medRxiv 2020.09.23.20191726; doi:<https://doi.org/10.1101/2020.09.23.20191726>

446. Scrutinizing the heterogeneous spreading of COVID-19 outbreak in Brazilian territory. Rafael Marques Da Silva, Carlos Fabio de Oliveira Mendes, Cesar Manchein. medRxiv 2020.06.05.20123604; doi:<https://doi.org/10.1101/2020.06.05.20123604>

447. Seasonality and Progression of COVID-19 among Countries With or Without Lock-downs. Dr. Jose-Luis Sagripanti. medRxiv 2020.12.06.20244780; doi:<https://doi.org/10.1101/2020.12.06.20244780>

448. Seasonality and uncertainty in COVID-19 growth rates. Cory Merow, Mark C. Urban. medRxiv 2020.04.19.20071951; doi: https://doi.org/10.1101/2020.04.19.20071951

449. Serial interval, basic reproduction number and prediction of COVID-19 epidemic size in Jodhpur, India. Suman Saurabh, Mahendra Kumar Verma, Vaishali Gautam, Akhil Goel, Manoj Kumar Gupta, Pankaj Bhardwaj, Sanjeev Misra. medRxiv 2020.07.03.20146167; doi:<https://doi.org/10.1101/2020.07.03.20146167>

450. Several countries in one: a mathematical modeling analysis for COVID-19 in inner Brazil. G.B. de Almeida, T.N. Vilches, C.P. Ferreira, C.M.C.B. Fortaleza. medRxiv 2020.04.23.20077438; doi:<https://doi.org/10.1101/2020.04.23.20077438>

451. Short-term forecasts and long-term mitigation evaluations for the COVID-19 epidemic in Hubei Province, China. Qihui Yang, Chunlin Yi, Aram Vajdi, Lee W Cohnstaedt, Hongyu Wu, Xiaolong Guo, Caterina M Scoglio. medRxiv 2020.03.27.20045625; doi:<https://doi.org/10.1101/2020.03.27.20045625>

452. Short-term forecasts to inform the response to the Covid-19 epidemic in the UK. S Funk, S Abbott, BD Atkins, M Baguelin, JK Baillie, P Birrell, J Blake, NI Bosse, J Burton, J Carruthers, NG Davies, D De Angelis, L Dyson, WJ Edmunds, RM Eggo, NM Ferguson, K Gaythorpe, E Gorsich, G Guyver-Fletcher, J Hellewell, EM Hill, A Holmes, TA House, C Jewell, M Jit, T Jombart, I Joshi, MJ Keeling, E Kendall, ES Knock, AJ Kucharski, KA Lythgoe, SR Meakin, JD Munday, PJM Openshaw, CE Overton, F Pagani, J Pearson, PN Perez-Guzman, L Pellis, F Scarabel, MG Semple, K Sherratt, M Tang, MJ Tildesley, E Van Leeuwen, LK Whittles, CMMID COVID-19 Working Group, Imperial College COVID-19 Response Team, ISARIC4C Investigators. medRxiv 2020.11.11.20220962; doi:<https://doi.org/10.1101/2020.11.11.20220962>

453. Shut and re-open: the role of schools in the spread of COVID-19 in Europe. [Helena B. Stage](https://arxiv.org/search/?searchtype=author&query=Stage%2C+H+B), [Joseph Shingleton](https://arxiv.org/search/?searchtype=author&query=Shingleton%2C+J), [Sanmitra Ghosh](https://arxiv.org/search/?searchtype=author&query=Ghosh%2C+S), [Francesca Scarabel](https://arxiv.org/search/?searchtype=author&query=Scarabel%2C+F), [Lorenzo Pellis](https://arxiv.org/search/?searchtype=author&query=Pellis%2C+L), [Thomas Finnie](https://arxiv.org/search/?searchtype=author&query=Finnie%2C+T). [arXiv:2006.14158](https://arxiv.org/abs/2006.14158)

454. Simple discrete-time self-exciting models can describe complex dynamic processes: a case study of COVID-19. Raiha Browning, Deborah Sulem, Kerrie Mengersen, Vincent Rivoirard, Judith Rousseau. medRxiv 2020.10.28.20221077; doi:<https://doi.org/10.1101/2020.10.28.20221077>

455. [Simple method for estimating daily and total COVID-19 deaths using a Gumbel model](https://discover.hsls.pitt.edu/vivisimo/cgi-bin/query-meta?v%3afile=viv_vnkHDb&v%3astate=root%7croot&url=https%3a%2f%2fdoi.org%2f10.21203%2frs.3.rs-120984%2fv1&rid=Ndoc1&v%3aframe=redirect&v%3aredirect-hash=1e4207868c3c86b9fac1cd1b724b0df3&) [new window](https://discover.hsls.pitt.edu/vivisimo/cgi-bin/query-meta?v%3afile=viv_vnkHDb&v%3astate=root%7croot&url=https%3a%2f%2fdoi.org%2f10.21203%2frs.3.rs-120984%2fv1&rid=Ndoc1&v%3aframe=redirect&v%3aredirect-hash=1e4207868c3c86b9fac1cd1b724b0df3&). Furutani H, Hiroyasu T, Okuhara Y. Research Square. Doi:<https://doi.org/10.21203/rs.3.rs-120984/v1>

456. Simple model for Covid-19 epidemics – back-casting in China and forecasting in the US. Slav W. Hermanowicz. medRxiv 2020.03.31.20049486; doi:<https://doi.org/10.1101/2020.03.31.20049486>

457. Simplified model of Covid-19 epidemic prognosis under quarantine and estimation of quarantine effectiveness. Algis Dziugys, Martynas Bieliunas, Gediminas Skarbalius, Edgaras Misiulis, Robertas Navakas. medRxiv 2020.04.28.20083428; doi:<https://doi.org/10.1101/2020.04.28.20083428>

458. Social disparities in the first wave of COVID-19 infections in Germany: A county-scale explainable machine learning approach. Gabriele Doblhammer, Constantin Reinke, Daniel Kreft. medRxiv 2020.12.22.20248386; doi:<https://doi.org/10.1101/2020.12.22.20248386>

459. Social Distancing with Movement Restrictions and the Effective Replication Number of COVID-19: Multi-Country Analysis Based on Phone Mobility Data. Mounir Ould Setti, Ari Voutilainen. medRxiv 2020.10.08.20209064; doi:<https://doi.org/10.1101/2020.10.08.20209064>

460. Social-distancing effectiveness tracking of the COVID-19 hotspot Stockholm. Joachim Oberhammer. medRxiv 2020.06.30.20143487; doi:<https://doi.org/10.1101/2020.06.30.20143487>

461. Solar geoengineering could redistribute malaria risk in developing countries. Colin J. Carlson, Rita Colwell, Mohammad Sharif Hossain, Mohammed Mofizur Rahman, Alan Robock, Sadie J. Ryan, Mohammad Shafiul Alam, Christopher H. Trisos. medRxiv 2020.10.21.20217257; doi:<https://doi.org/10.1101/2020.10.21.20217257>

462. Spatial Allocation of Scarce Vaccine and Antivirals for COVID-19. François M. Castonguay, Julie C. Blackwood, Emily Howerton, Katriona M. Shea, Charles Sims, James N. Sanchirico. medRxiv 2020.12.18.20248439; doi:<https://doi.org/10.1101/2020.12.18.20248439>

463. Spatial and temporal regularization to estimate COVID-19 Reproduction Number R(t): Promoting piecewise smoothness via convex optimization. patrice abry, nelly pustelnik, stephane roux, pablo jensen, Patrick Flandrin, remi gribonval, Charles G Lucas, eric guichard, Pierre Borgnat, nicolas garnier, benjamin audit. medRxiv 2020.06.10.20127365; doi:<https://doi.org/10.1101/2020.06.10.20127365>

464. Spread of Covid-19 in the United States is controlled. Zixin Hu, Qiyang Ge, Shudi Li, Tao Xu, Eric Boerwinkle, Li Jin, Momiao Xiong. medRxiv 2020.05.04.20091272; doi:<https://doi.org/10.1101/2020.05.04.20091272>

465. State heterogeneity of human mobility and COVID-19 epidemics in the European Union. Xiaoling Yuan, Kun Hu, Jie Xu, Xuchen Zhang, Wei Bao, Charles F Lynch, Lanjing Zhang. medRxiv 2020.06.10.20127530; doi:<https://doi.org/10.1101/2020.06.10.20127530>

466. State-by-State estimates of R0 at the start of COVID-19 outbreaks in the USA. Anthony R. Ives, Claudio Bozzuto. medRxiv 2020.05.17.20104653; doi:<https://doi.org/10.1101/2020.05.17.20104653>

467. State-specific Projection of COVID-19 Infection in the United States and Evaluation of Three Major Control Measures. Shi Chen, Qin Li, Song Gao, Yuhao Kang, Xun Shi. medRxiv 2020.04.03.20052720; doi:<https://doi.org/10.1101/2020.04.03.20052720>

468. Stay-at-home policy: is it a case of exception fallacy? An internet-based ecological study. Ricardo F. Savaris, Guilherme Pumi, Jovani Dalzochio, Rafael Kunst. medRxiv 2020.10.13.20211284; doi:<https://doi.org/10.1101/2020.10.13.20211284>

469. Strict lockdown versus flexible social distance strategy for COVID-19 disease: a cost-effectiveness analysis. Ben W. Mol, Jonathan Karnon. medRxiv 2020.09.14.20194605; doi:<https://doi.org/10.1101/2020.09.14.20194605>.

470. Studies of Novel Coronavirus Disease 19 (COVID-19) Pandemic: A Global Analysis of Literature. Bach Xuan Tran, Giang Hai Ha, Long Hoang Nguyen, Giang Thu Vu, Hai Thanh Phan, Huong Thi Le, Carl A. Latkin, Cyrus S.H. Ho, Roger C.M. Ho. medRxiv 2020.05.05.20092635; doi:<https://doi.org/10.1101/2020.05.05.20092635>

471. Summer vacation and COVID-19: effects of metropolitan people going to summer provinces. [Tom Britton](https://arxiv.org/search/?searchtype=author&query=Britton%2C+T), [Frank Ball](https://arxiv.org/search/?searchtype=author&query=Ball%2C+F). [arXiv:2006.00579](https://arxiv.org/abs/2006.00579)

472. Supporting Austria through the COVID-19 Epidemics with a Forecast-Based Early Warning System. Martin Bicher, Martin Zuba, Lukas Rainer, Florian Bachner, Claire Rippinger, Herwig Ostermann, Nikolas Popper, Stefan Thurner, Peter Klimek- medRxiv 2020.10.18.20214767; doi:<https://doi.org/10.1101/2020.10.18.20214767>

473. Survival-Convolution Models for Predicting COVID-19 Cases and Assessing Effects of Mitigation Strategies. Qinxia Wang, Shanghong Xie, Yuanjia Wang, Donglin Zeng. medRxiv 2020.04.16.20067306; doi:<https://doi.org/10.1101/2020.04.16.20067306>

474. Synchronized travel restrictions across cities can be effective in COVID-19 control. Haiyan Liu, Xuemei Bai, Huanfeng Shen, Xiaoping Pang, Zeyu Liang, Yue Liu. medRxiv 2020.04.02.20050781; doi:<https://doi.org/10.1101/2020.04.02.20050781>

475. Temporal dynamics in viral shedding and transmissibility of COVID-19. Xi He, Eric HY Lau, Peng Wu, Xilong Deng, Jian Wang, Xinxin Hao, Yiu Chung Lau, Jessica Y Wong, Yujuan Guan, Xinghua Tan, Xiaoneng Mo, Yanqing Chen, Baolin Liao, Weilie Chen, Fengyu Hu, Qing Zhang, Mingqiu Zhong, Yanrong Wu, Lingzhai Zhao, Fuchun Zhang, Benjamin J Cowling, Fang Li, Gabriel M Leung. medRxiv 2020.03.15.20036707; doi:<https://doi.org/10.1101/2020.03.15.20036707>

476. Test-adjusted results of mortality for Covid-19 in Germany, USA, UK. Jürgen Mimkes, Rainer Janssen. medRxiv 2020.11.03.20225268; doi:<https://doi.org/10.1101/2020.11.03.20225268>

477. Testing for tracing or testing just for treating? A comparative analysis of strategies to face COVID-19 pandemic. Ricardo Knudsen. medRxiv 2020.06.01.20119123; doi:<https://doi.org/10.1101/2020.06.01.20119123>

478. Testing informed sir based epidemiological model for covid-19 in Luxembourg. Thomas Sauter, Maria Pires Pacheco. medRxiv 2020.07.21.20159046; doi:<https://doi.org/10.1101/2020.07.21.20159046>

479. The amplified second outbreaks of global COVID-19 pandemic. Jianping Huang, Xiaoyue Liu, Li Zhang, Kehu Yang, Yaolong Chen, Zhongwei Huang, Chuwei Liu, Xinbo Lian, Danfeng Wang. medRxiv 2020.07.15.20154161; doi:<https://doi.org/10.1101/2020.07.15.20154161>

480. The association between COVID-19-imposed lockdowns and online searches for toothache using Google Trends. Ahmad Sofi-Mahmudi, Erfan Shamsoddin, Peyman Ghasemi, Mona Nasser, Bita Mesgarpour. medRxiv 2020.08.01.20157065; doi:<https://doi.org/10.1101/2020.08.01.20157065>

481. The autoregressive neural network model for COVID-19 outbreak predictions. Tanujit Chakraborty, Arinjita Bhattacharyya, Monalisha Pattnaik. medRxiv 2020.10.01.20205021; doi:<https://doi.org/10.1101/2020.10.01.20205021>

482. The change pattern and significance of IgM and IgG in the progress of COVID-19 disease. Xuzhen Qin, Jun Shen, Erhei Dai, Haolong Li, Guodong Tang, Lixia Zhang, Xin Hou, Minya Lu, Xian Wu, Simeng Duan, Jingjia Zhang, Yongzhe Li. medRxiv 2020.07.20.20157446; doi:<https://doi.org/10.1101/2020.07.20.20157446>

483. The Common Interests of Health Protection and the Economy: Evidence from Scenario Calculations of COVID-19 Containment Policies. Florian Dorn, Sahamoddin Khailaie, Marc Stoeckli, Sebastian C Binder, Berit Lange, Stefan Lautenbacher, Andreas Peichl, Patrizio Vanella, Timo Wollmershaeuser, Clemens Fuest, Michael Meyer-Hermann

484. The Covid-19 infection in Italy: a statistical study of an abnormally severe disease. Giuseppe De Natale, Valerio Ricciardi, Gabriele De Luca, Dario De Natale, Giovanni Di Meglio, Antonio Ferragamo, Vito Marchitelli, Andrea Piccolo, Antonio Scala, Renato Somma, Emanuele Spina, Claudia Troise medRxiv 2020.03.28.20046243; doi:<https://doi.org/10.1101/2020.03.28.20046243>

485. The COVID-19 Spread Patterns in Italy and India: A Comparison of the Current Situations. Hemanta Kumar Baruah. medRxiv 2020.06.21.20136630; doi:<https://doi.org/10.1101/2020.06.21.20136630>

486. The current COVID-19 wave will likely be mitigated in the second-line European countries. S. Soubeyrand, M. Ribaud, V. Baudrot, D. Allard, D. Pommeret, L. Roques. medRxiv 2020.04.17.20069179; doi: https://doi.org/10.1101/2020.04.17.20069179

487. The Effect of Gender on Covid-19 Infections and Mortality in Germany: Insights From Age- and Sex-Specific Modelling of Contact Rates, Infections, and Deaths. Achim Dörre, Gabriele Doblhammer. medRxiv 2020.10.06.20207951; doi:<https://doi.org/10.1101/2020.10.06.20207951>

488. The effect of multiple interventions to balance healthcare demand for controlling COVID-19 outbreaks: a modelling study. Po Yang, Jun Qi, Shuhao Zhang, Xulong Wang, Gaoshan Bi, Yun Yang, Bin Sheng, Xuxin Mad. medRxiv 2020.05.19.20107326; doi:<https://doi.org/10.1101/2020.05.19.20107326>

489. The effect of school closures and reopening strategies on COVID-19 infection dynamics in the San Francisco Bay Area: a cross-sectional survey and modeling analysis. Jennifer R Head, Kristin Andrejko, Qu Cheng, Philip A Collender, Sophie Phillips, Anna Boser, Alexandra K Heaney, Christopher M Hoover, Sean L Wu, Graham R Northrup, Karen Click, Robert Harrison, Joseph A Lewnard, Justin V Remais. medRxiv 2020.08.06.20169797; doi:<https://doi.org/10.1101/2020.08.06.20169797>

490. The effectiveness of eight nonpharmaceutical interventions against COVID-19 in 41 countriesJan M. Brauner, Sören Mindermann, Mrinank Sharma, David Johnston, John Salvatier, Tomáš Gavenčiak, Anna B. Stephenson, Gavin Leech, George Altman, Vladimir Mikulik, Alexander John Norman, Joshua Teperowski Monrad, Tamay Besiroglu, Hong Ge, Meghan A. Hartwick, Yee Whye Teh, Leonid Chindelevitch, Yarin Gal, Jan Kulveit. medRxiv 2020.05.28.20116129; doi:<https://doi.org/10.1101/2020.05.28.20116129>

491. The effectiveness of eight nonpharmaceutical interventions against COVID-19 in 41 countries. Jan M. Brauner, Sören Mindermann, Mrinank Sharma, David Johnston, John Salvatier, Tomáš Gavenčiak, Anna B. Stephenson, Gavin Leech, George Altman, Vladimir Mikulik, Alexander John Norman, Joshua Teperowski Monrad, Tamay Besiroglu, Hong Ge, Meghan A. Hartwick, Yee Whye Teh, [View ORCID Profile](http://orcid.org/0000-0002-6619-6013)Leonid Chindelevitch, Yarin Gal, Jan Kulveit. medRxiv 2020.05.28.20116129; doi:<https://doi.org/10.1101/2020.05.28.20116129>

492. The epidemiologic parameters for COVID-19: A Systematic Review and Meta-Analysis. Neda Izadi, Niloufar Taherpour, Yaser Mokhayeri, Sahar Sotoodeh Ghorbani, Khaled Rahmani, Seyed Saeed Hashemi Nazari. medRxiv 2020.05.02.20088385; doi:<https://doi.org/10.1101/2020.05.02.20088385>

493. The Epidemiologic Transition Theory and Evidence for Cancer Transitions in the US, Select European Nations, and Japan. Omer Gersten, Magali Barbieri. medRxiv 2020.11.25.20238832; doi:<https://doi.org/10.1101/2020.11.25.20238832>

494. The first three months of the COVID-19 epidemic: Epidemiological evidence for two separate strains of SARS-CoV-2 viruses spreading and implications for prevention strategies. Knut M. Wittkowski. medRxiv 2020.03.28.20036715; doi:<https://doi.org/10.1101/2020.03.28.20036715>

495. The First Wave of COVID-19 in Israel - Initial Analysis of Publicly Available Data. Mark Last. medRxiv 2020.05.05.20091645; doi:<https://doi.org/10.1101/2020.05.05.20091645>

496. The focus and timing of COVID-19 pandemic control measures under healthcare resource constraints. Chen Wei, Zhengyang Wang, Zhichao Liang, Quanying Liu. medRxiv 2020.04.16.20067611; doi:<https://doi.org/10.1101/2020.04.16.20067611>

497. The health sector cost of different policy responses to COVID-19 in low- and middle- income countries. Sergio Torres Rueda, Sedona Sweeney, Fiammetta Bozzani, Anna Vassall. medRxiv 2020.08.23.20180299; doi: https://doi.org/10.1101/2020.08.23.20180299

498. The impact of current and future control measures on the spread of COVID-19 in Germany. Maria Vittoria Barbarossa, Jan Fuhrmann, Jan H. Meinke, Stefan Krieg, Hridya Vinod Varma, Noemi Castelletti, Thomas Lippert. medRxiv 2020.04.18.20069955; doi:<https://doi.org/10.1101/2020.04.18.20069955>

499. The impact of lockdown on public health during the first wave of covid-19 pandemic: lessons learned for designing effective containment measures to cope with second wave. Mario Coccia. medRxiv 2020.10.22.20217695; doi:<https://doi.org/10.1101/2020.10.22.20217695>

500. The impact of non-pharmaceutical interventions on the prevention and control of COVID-19 in New York City. Jiannan Yang, Qingpeng Zhang, Zhidong Cao, Jianxi Gao, Dirk Pfeiffer, Lu Zhong, Daniel Dajun Zeng. medRxiv 2020.12.01.20242347; doi:<https://doi.org/10.1101/2020.12.01.20242347>

501. The Infection Rate of the Coronavirus Disease 2019 (COVID-19) in Wuhan, China. Hui-Qi Qu, Zhangkai J. Cheng, Zhifeng Duan, Lifeng Tian, Hakon Hakonarson. medRxiv 2020.05.02.20088724; doi:<https://doi.org/10.1101/2020.05.02.20088724>

502. The misleading illusion of COVID-19 confirmed case data: alternative estimates and a monitoring tool. Rogelio Macías-Ordóñez, Damián Villaseñor-Amador. medRxiv 2020.05.20.20107516; doi:<https://doi.org/10.1101/2020.05.20.20107516>

503. The overall mortality caused by COVID-19 in the European region is highly associated with demographic composition: A spatial regression-based approach. [Srikanta Sannigrahi](https://arxiv.org/search/?searchtype=author&query=Sannigrahi%2C+S), [Francesco Pilla](https://arxiv.org/search/?searchtype=author&query=Pilla%2C+F), [Bidroha Basu](https://arxiv.org/search/?searchtype=author&query=Basu%2C+B), [Arunima Sarkar Basu](https://arxiv.org/search/?searchtype=author&query=Basu%2C+A+S). [arXiv:2005.04029](https://arxiv.org/abs/2005.04029)

504. The overall mortality caused by COVID-19 in the European region is highly associated with demographic composition: A spatial regression-based approach, [Srikanta Sannigrahi](https://arxiv.org/search/?searchtype=author&query=Sannigrahi%2C+S), [Francesco Pilla](https://arxiv.org/search/?searchtype=author&query=Pilla%2C+F), [Bidroha Basu](https://arxiv.org/search/?searchtype=author&query=Basu%2C+B), [Arunima Sarkar Basu](https://arxiv.org/search/?searchtype=author&query=Basu%2C+A+S). [arXiv:2005.04029](https://arxiv.org/abs/2005.04029)

505. The performance of Mobile Cabin Hospital in combatting COVID-19 in China. Hongru Li, Jiaping Lin II, Hongmei Lian, Kang Chen, Yongtao Lyu, Yusheng Chen, Lili Ren, Li Zheng, Zhisheng Lin, Xueying Yu, Zihan Chen, Christopher Rensing, Xin Qian, Xinghai Yang. medRxiv 2020.07.26.20162206; doi:<https://doi.org/10.1101/2020.07.26.20162206>

506. The Prediction for the Outbreak of COVID-19 for 15 States in USA by Using Turning Phase Concepts as of April 10, 2020. George Xianzhi Yuan, Lan Di, Yudi Gu, Guoqi Qian, Xiaosong Qian. medRxiv 2020.04.13.20064048; doi:<https://doi.org/10.1101/2020.04.13.20064048>

507. [The Psychology Underlying Biased Forecasts of COVID-19 Cases and Deaths in the United States](https://discover.hsls.pitt.edu/vivisimo/cgi-bin/query-meta?v%3afile=viv_vnkHDb&v%3astate=root%7croot-25-25%7c0&url=https%3a%2f%2fdoi.org%2f10.2139%2fssrn.3665774&rid=Ndoc28&v%3aframe=redirect&v%3aredirect-hash=66463394949004a75925d7490f1a0375&). Shefrin H.SSRN 2020-08-10. Doi:https://doi.org/10.2139/ssrn.3665774

508. The Relationship between Weekly Periodicity and COVID-19 Progression. Sophia Li. medRxiv 2020.11.24.20238295; doi:<https://doi.org/10.1101/2020.11.24.20238295>

509. The reproduction number of COVID-19 and its correlation with public health interventions. Kevin Linka, Mathias Peirlinck, Ellen Kuhl. medRxiv 2020.05.01.20088047; doi: https://doi.org/10.1101/2020.05.01.20088047

510. The reproduction number R for COVID-19 in England: Why hasn′t ″lockdown″ been more effective? Alastair Grant. medRxiv 2020.07.02.20144840; doi:<https://doi.org/10.1101/2020.07.02.20144840>

511. The reproductive index from SEIR model of Covid-19 epidemic in Asean. Pongkeaw Udomsamuthirun, Grittichon Chanilkul, Pongkarn Tongkhonburi, Chatcharawan Meesubthong. medRxiv 2020.04.24.20078287; doi:<https://doi.org/10.1101/2020.04.24.20078287>

512. The scale and dynamics of COVID-19 epidemics across Europe. Christopher Dye, Russell C.H. Cheng, John S. Dagpunar, Brian G. Williams. medRxiv 2020.06.26.20131144; doi:<https://doi.org/10.1101/2020.06.26.20131144>

513. The socio-economic determinants of the coronavirus disease (COVID-19) pandemic. Viktor Stojkoski, Zoran Utkovski, Petar Jolakoski, Dragan Tevdovski, Ljupco Kocarev. medRxiv 2020.04.15.20066068; doi:<https://doi.org/10.1101/2020.04.15.20066068>

514. The time to offer treatments for covid-19. Binh T. Ngo, Paul Marik, Pierre Kory, Leland Shapiro, Raphael Thomadsen, Jose Iglesias, Stephen Ditmore, Marc Rendell, Joseph Varon, Michael Dubé, Neha Nanda, Gino In, Daniel Arkfeld, Preet Chaudhary, Vito M. Campese, Diana L. Hanna, David E. Sawcer, Glenn Ehresmann, David Peng, Miroslaw Smogorewski, April Armstrong, Rajkumar Dasgupta, Fred Sattler, Denise Brennan-Rieder, Cristina Mussini, Oriol Mitja, Vicente Soriano, Nicolas Peschanski, Gilles Hayem, Marco Confalonieri, Maria Carmela Piccirillo, Antonio Lobo-Ferreira, Iraldo Bello Rivero, Eivind H. Vinjevoll, Daniel Griffin, Ivan FN Hung. medRxiv 2020.05.27.20115238; doi:<https://doi.org/10.1101/2020.05.27.20115238>

515. The timing and effectiveness of implementing mild interventions of COVID-19 in large industrial regions via a synthetic control method. Ting Tian, Wenxiang Luo, Jianbin Tan, Yukang Jiang, Minqiong Chen, Wenliang Pan, Songpan Yang, Jiashu Zhao, Xueqin Wang, Heping Zhang. medRxiv 2020.06.22.20137380; doi:<https://doi.org/10.1101/2020.06.22.20137380>

516. The timing of contact restrictions and pro-active testing balances the socio-economic impact of a lockdown with the control of infections. Saptarshi Bej, Olaf Wolkenhauer. medRxiv 2020.05.08.20095596; doi:<https://doi.org/10.1101/2020.05.08.20095596>

517. [The COVID-19 Crash in the US Stock Market](https://discover.hsls.pitt.edu/vivisimo/cgi-bin/query-meta?v%3afile=viv_vnkHDb&v%3astate=root%7croot-25-25%7c0&url=https%3a%2f%2fdoi.org%2f10.2139%2fssrn.3632410&rid=Ndoc39&v%3aframe=redirect&v%3aredirect-hash=b270dcdaa7026a2f7aa19df1f4e8f01c&). Ziemba WT.SSRN 2020-07-01. Doi:<https://doi.org/10.2139/ssrn.3632410>

518. [The Prediction for the Outbreak of COVID-19 for 15 States in USA by Using Turning Phase Concepts as of April 10, 2020](https://discover.hsls.pitt.edu/vivisimo/cgi-bin/query-meta?v%3afile=viv_vnkHDb&v%3astate=root%7croot-75-25%7c0&url=https%3a%2f%2fdoi.org%2f10.1101%2f2020.04.13.20064048&rid=Ndoc85&v%3aframe=redirect&v%3aredirect-hash=3cb7f4a1c7e71f28c34660def7514fb7&). Yuan GX, Di L, Gu Y, Qian G, Qian X. medRxiv 2020-04-17. Doi:<https://doi.org/10.1101/2020.04.13.20064048>

519. Time Dynamics of COVID-19. Cody Carroll, Satarupa Bhattacharjee, Yaqing Chen, Paromita Dubey, Jianing Fan, Álvaro Gajardo, Xiner Zhou, Hans-Georg Müller, Jane-Ling Wang. medRxiv 2020.05.21.20109405; doi:<https://doi.org/10.1101/2020.05.21.20109405>

520. Time use and social mixing during and around festive periods: Potential changes in the age distribution of COVID-19 cases from increased intergenerational interactions. Edwin van Leeuwen, Frank G. Sandmann, Rosalind M. Eggo, PHE Joint modelling group, Peter J. White. medRxiv 2020.12.21.20248607; doi:<https://doi.org/10.1101/2020.12.21.20248607>

521. Time-dependent heterogeneity leads to transient suppression of COVID-19 epidemic, not herd immunity. Alexei V. Tkachenko, Sergei Maslov, Ahmed Elbanna, George N. Wong, Zachary J. Weiner, Nigel Goldenfeld. medRxiv 2020.07.26.20162420; doi:<https://doi.org/10.1101/2020.07.26.20162420>

522. Towards Integrated and Open COVID-19 Data. [Georgios M. Santipantakis](https://arxiv.org/search/?searchtype=author&query=Santipantakis%2C+G+M), [George A. Vouros](https://arxiv.org/search/?searchtype=author&query=Vouros%2C+G+A), [Christos Doulkeridis](https://arxiv.org/search/?searchtype=author&query=Doulkeridis%2C+C). [arXiv:2008.04045](https://arxiv.org/abs/2008.04045)

523. Tracking and Predicting COVID-19 Epidemic in China Mainland. Haoxuan Sun, Yumou Qiu, Han Yan, Yaxuan Huang, Yuru Zhu, Song Xi Chen. medRxiv 2020.02.17.20024257; doi:<https://doi.org/10.1101/2020.02.17.20024257>

524. Tracking ℛ of COVID-19: A New Real-Time Estimation Using the Kalman Filter. Francisco Arroyo-Marioli, Francisco Bullano, Simas Kučinskas, Carlos Rondón-Moreno. medRxiv 2020.04.19.20071886; doi:<https://doi.org/10.1101/2020.04.19.20071886>

525. Transmission dynamics and control measures of COVID-19 outbreak in China: a modelling study. XuSheng Zhang, Emilia Vynnycky, Andre Charlett, Daniela de Angelis, Zhengji Chen, Wei Liu. medRxiv 2020.07.09.20150086; doi:<https://doi.org/10.1101/2020.07.09.20150086>

526. Transmission dynamics and control of COVID-19 in Chile, March-October, 2020. Amna Tariq, Eduardo A. Undurraga, Carla Castillo Laborde, Katia Vogt-Geisse, Ruiyan Luo, Richard Rothenberg, Gerardo Chowell. medRxiv 2020.05.15.20103069; doi:<https://doi.org/10.1101/2020.05.15.20103069>

527. Transmission Dynamics of Coronavirus Disease 2019 (COVID-19) in the World: The Roles of Intervention and Seasonality. Shunxiang Huang, Lin Wu, Li Xu, Aihong Zhang, Li Sheng, Feng Liu, Long Zhou, Jing Li, Rongzhang Hao, Hua Qian, Sheng Fang, Zhongyi Wang, Yingru Li, Yuguo Li, Chan Lu, Qihong Deng. medRxiv 2020.07.17.20156430; doi:<https://doi.org/10.1101/2020.07.17.20156430>

528. Transmission dynamics of the COVID-19 epidemic in India and modelling optimal lockdown exit strategies. Mohak Gupta, Saptarshi Soham Mohanta, Aditi Rao, Giridara Gopal Parameswaran, Mudit Agarwal, Mehak Arora, Archisman Mazumder, Ayush Lohiya, Priyamadhaba Behera, Agam Bansal, Rohit Kumar, Ved Prakash Meena, Pawan Tiwari, Anant Mohan, Sushma Bhatnagar. medRxiv 2020.05.13.20096826; doi:<https://doi.org/10.1101/2020.05.13.20096826>

529. Transmission Dynamics of the COVID-19 Epidemics in England. Yang Liu, Julian W Tang, Tommy TY Lam. medRxiv 2020.06.30.20143743; doi:<https://doi.org/10.1101/2020.06.30.20143743>

530. Transmission interval estimates suggest pre-symptomatic spread of COVID-19. Lauren C. Tindale, Michelle Coombe, Jessica E. Stockdale, Emma S. Garlock, Wing Yin Venus Lau, Manu Saraswat, Yen-Hsiang Brian Lee, Louxin Zhang, Dongxuan Chen, Jacco Wallinga, Caroline Colijn. medRxiv 2020.03.03.20029983; doi:<https://doi.org/10.1101/2020.03.03.20029983>

531. Transmission of COVID-19 in the state of Georgia, United States: Spatiotemporal variation and impact of social distancing. Yuke Wang, Casey Siesel, Yangping Chen, Ben Lopman, Laura Edison, Michael Thomas, Carly Adams, Max Lau, Peter F.M. Teunis. medRxiv 2020.10.22.20217661; doi:<https://doi.org/10.1101/2020.10.22.20217661>

532. Transmission potential of COVID-19 in South Korea. Eunha Shim, Amna Tariq, Wongyeong Choi, Yiseul Lee, Gerardo Chowell. medRxiv 2020.02.27.20028829; doi:<https://doi.org/10.1101/2020.02.27.20028829>

533. Transparency Assessment of COVID-19 Models. Mohammad S. Jalali, Catherine DiGennaro, Devi Sridhar. medRxiv 2020.07.18.20156851; doi:<https://doi.org/10.1101/2020.07.18.20156851>

534. Trends of SARS-Cov-2 infection in 67 countries: Role of climate zone, temperature, humidity and curve behavior of cumulative frequency on duplication time. Jaime Berumen, Max Schmulson, Guadalupe Guerrero, Elizabeth Barrera, Jorge Larriva-Sahd, Gustavo Olaiz, Rebeca Garcia-Leyva, Rosa María Wong Chew, Miguel Betancourt-Cravioto, Héctor Gallardo, Germán Fajardo-Dolci, Roberto Tapia-Conyer. medRxiv 2020.04.18.20070920; doi:<https://doi.org/10.1101/2020.04.18.20070920>

535. Two alternative scenarios for easing COVID-19 lockdown measures: one reasonable and one catastrophic. A.S. Fokas, J. Cuevas-Maraver, P. G. Kevrekidis. medRxiv 2020.05.08.20095380; doi: https://doi.org/10.1101/2020.05.08.20095380

536. Uncertainty Quantification in Epidemiological Models for COVID-19 Pandemic. Leila Taghizadeh, Ahmad Karimi, Clemens Heitzinger. medRxiv 2020.05.30.20117754; doi: https://doi.org/10.1101/2020.05.30.20117754

537. [Uncertainty Quantification in Epidemiological Models for COVID-19 Pandemic](https://discover.hsls.pitt.edu/vivisimo/cgi-bin/query-meta?v%3afile=viv_vnkHDb&v%3astate=root%7croot-25-25%7c0&url=https%3a%2f%2fdoi.org%2f10.1101%2f2020.05.30.20117754&rid=Ndoc51&v%3aframe=redirect&v%3aredirect-hash=67dd560ea1d2b7fb41068d2d9bcff86f&). Taghizadeh L, Karimi A, Heitzinger C. medRxiv 2020-06-03. Doi:<https://doi.org/10.1101/2020.05.30.20117754>

538. [Understanding Credit Risk for Chinese Companies: A Default-based Approach](https://discover.hsls.pitt.edu/vivisimo/cgi-bin/query-meta?v%3afile=viv_vnkHDb&v%3astate=root%7croot&url=https%3a%2f%2fdoi.org%2f10.2139%2fssrn.3734053&rid=Ndoc6&v%3aframe=redirect&v%3aredirect-hash=fb5a24f3d1583cc2e15d9da40bb40ee8&) [new window](https://discover.hsls.pitt.edu/vivisimo/cgi-bin/query-meta?v%3afile=viv_vnkHDb&v%3astate=root%7croot&url=https%3a%2f%2fdoi.org%2f10.2139%2fssrn.3734053&rid=Ndoc6&v%3aframe=redirect&v%3aredirect-hash=fb5a24f3d1583cc2e15d9da40bb40ee8&). Altman EI, Hu X, Yu J. SSRN:<https://doi.org/10.2139/ssrn.3734053>

539. Understanding Economic and Health Factors Impacting the Spread of COVID-19 Disease. Aleksandr Farseev, Yu-Yi Chu-Farseeva, Yang Qi, Daron Benjamin Loo. medRxiv 2020.04.10.20058222; doi: https://doi.org/10.1101/2020.04.10.20058222

540. Understanding SARSCOV-2 propagation, impacting factors to derive possible scenarios and simulations. Lewis E. Mehl-Madrona, Francois Bricaire, Adrian Cuyugan, Jovan Barac, Asadullah Parvaiz, Ali Bin Jamil, Sajid Iqbal, Meryem Koliali, Ryan Vally, Mohamed Karim Sellier. medRxiv 2020.09.07.20190066; doi:<https://doi.org/10.1101/2020.09.07.20190066>

541. Understanding Spatial Heterogeneity of COVID-19 Pandemic Using Shape Analysis of Growth Rate Curves. Anuj Srivastava, Gerardo Chowell. medRxiv 2020.05.25.20112433; doi: https://doi.org/10.1101/2020.05.25.20112433

542. Understanding the asymmetric spread and case fatality rate (CFR) for COVID-19 among countries. Eldhose Iype, Sadhya Gulati. medRxiv 2020.04.21.20073791; doi:<https://doi.org/10.1101/2020.04.21.20073791>

543. Understanding the Collective Responses of Populations to the COVID-19 Pandemic in Mainland China. Haoyi Xiong, Ji Liu, Jizhou Huang, Siyu Huang, Haozhe An, Qi Kang, Ying Li, Dejing Dou, Haifeng Wang. medRxiv 2020.04.20.20068676; doi:<https://doi.org/10.1101/2020.04.20.20068676>

544. Universal scaling law for COVID-19 propagation in urban centers. Ben-Hur Francisco Cardoso, Sebastian Goncalves. medRxiv 2020.06.22.20137604; doi:<https://doi.org/10.1101/2020.06.22.20137604>

545. Updating Herd Immunity Models for the U.S. in 2020: Implications for the COVID-19 Response. Natalie E. Sheils, Gregory D. Lyng, Ethan M. Berke. medRxiv 2020.10.05.20207100; doi:<https://doi.org/10.1101/2020.10.05.20207100>

546. Use of Artificial Intelligence on spatio-temporal data to generate insights during COVID-19 pandemic: A Review. Gihan Jayatilaka, Jameel Hassan, Umar Marikkar, Rumali Perera, Suren Sritharan, Harshana Weligampola, Mevan Ekanayake, Roshan Godaliyadda, Parakrama Ekanayake, Vijitha Herath, G M Dilshan Godaliyadda, Anuruddhika Rathnayake, Samath D. Dharmaratne, Janaka Ekanayake. medRxiv 2020.11.22.20232959; doi:<https://doi.org/10.1101/2020.11.22.20232959>

547. Using COVID-19 deaths as a surrogate to measure the progression of the pandemics. Carlos Hernandez-Suarez, Efren Murillo-Zamora. medRxiv 2020.09.27.20202564; doi:<https://doi.org/10.1101/2020.09.27.20202564>

548. Using newspapers obituaries to nowcast daily mortality: evidence from the Italian COVID-19 hot-spots. Marcello Puca, Paolo Buonanno. medRxiv 2020.05.31.20117168; doi:<https://doi.org/10.1101/2020.05.31.20117168>

549. [Using Soccer Games as an Instrument to Forecast the Spread of COVID-19 in Europe](https://discover.hsls.pitt.edu/vivisimo/cgi-bin/query-meta?v%3afile=viv_vnkHDb&v%3astate=root%7croot&url=https%3a%2f%2fdoi.org%2f10.2139%2fssrn.3701022&rid=Ndoc20&v%3aframe=redirect&v%3aredirect-hash=cc7f0cc90eaafb7776883799bd411424&). Gomez J, Mironov M. SSRN2020-10-05. Doi: https://doi.org/10.2139/ssrn.3701022

550. Vaccine optimization for COVID-19: who to vaccinate first? Laura Matrajt, Julia Eaton, Tiffany Leung, Elizabeth R. Brown. medRxiv 2020.08.14.20175257; doi:<https://doi.org/10.1101/2020.08.14.20175257>

551. Variation in human mobility and its impact on the risk of future COVID-19 outbreaks in Taiwan. Meng-Chun Chang, Rebecca Kahn, Yu-An Li, Cheng-Sheng Lee, Caroline O Buckee, Hsiao-Han Chang. medRxiv 2020.04.07.20053439; doi:<https://doi.org/10.1101/2020.04.07.20053439>

552. Visual Scoring of Chest CT at Hospital Admission Predicts Hospitalization Time and Intensive Care Admission in Covid-19. Erik Ahlstrand, Sara Cajander, Per Cajander, Edvin Ingberg, Erika Löf, Matthias Wegener, Mats Lidén. medRxiv 2020.10.30.20222471; doi:<https://doi.org/10.1101/2020.10.30.20222471>

553. Warmer weather and global trends in the coronavirus COVID-19. Hong Li, Hongwei Xiao, Renguo Zhu, Chengxing Sun, Cheng Liu, Hua-Yun Xiao. medRxiv 2020.04.28.20084004; doi:<https://doi.org/10.1101/2020.04.28.20084004>

554. Waves of COVID-19 pandemic. Detection and SIR simulations. Igor Nesteruk. medRxiv 2020.08.03.20167098; doi:<https://doi.org/10.1101/2020.08.03.20167098>

555. What does simple power law kinetics tell about our response to coronavirus pandemic? Prateek K. Jha. medRxiv 2020.04.03.20051797; doi:<https://doi.org/10.1101/2020.04.03.20051797>

556. What triggers online help-seeking retransmission during the COVID-19 period? Empirical evidence from Chinese social media. Chen Luo, Yuru Li, Anfan Chen, Yulong Tang. medRxiv 2020.06.13.20130054; doi:<https://doi.org/10.1101/2020.06.13.20130054>

557. Which COVID policies are most effective? A Bayesian analysis of COVID-19 by jurisdiction. Phebo Wibbens, Wesley Wu-Yi Koo, Anita M. McGahan. medRxiv 2020.12.01.20241695; doi:<https://doi.org/10.1101/2020.12.01.20241695>

558. Who can go back to work when the COVID-19 pandemic remits? Luis Angel Hierro, David Cantarero, David Patiño, Daniel Rodríguez-Pérez de Arenaza. medRxiv 2020.05.06.20093344; doi:<https://doi.org/10.1101/2020.05.06.20093344>

559. Who should we test for COVID-19? A triage model built from national symptom surveys. Saar Shoer, Tal Karady, Ayya Keshet, Smadar Shilo, Hagai Rossman, Amir Gavrieli, Tomer Meir, Amit Lavon, Dmitry Kolobkov, Iris Kalka, Anastasia Godneva, Ori Cohen, Adam Kariv, Ori Hoch, Mushon Zer-Aviv, Noam Castel, Carole Sudre, Anat Ekka Zohar, Angela Irony, Timothy Spector, Benjamin Geiger, Dorit Hizi, Varda Shalev, Ran Balicer, Eran Segal. medRxiv 2020.05.18.20105569; doi:<https://doi.org/10.1101/2020.05.18.20105569>

560. Why COVID-19 models should incorporate the network of social interactions. Helena A Herrmann, Jean-Marc Schwartz. medRxiv 2020.04.02.20050468; doi:<https://doi.org/10.1101/2020.04.02.20050468>

561. Will the COVID-19 pandemic lead to a tsunami of suicides? A Swedish nationwide analysis of historical and 2020 data. Christian Rück, David Mataix-Cols, Kinda Malki, Mats Adler, Oskar Flygare, Bo Runeson, Anna Sidorchuk. medRxiv 2020.12.10.20244699; doi:<https://doi.org/10.1101/2020.12.10.20244699>

562. Women in power: Female leadership and public health outcomes during the COVID-19 pandemic. Luca Coscieme, Lorenzo Fioramonti, Lars F Mortensen, Kate E Pickett, Ida Kubiszewski, Hunter Lovins, Jacqueline McGlade, Kristin Vala Ragnarsdottir, Debra Roberts, Robert Costanza, Roberto De Vogli, Richard Wilkinson. medRxiv 2020.07.13.20152397; doi:<https://doi.org/10.1101/2020.07.13.20152397>

563. Worldwide and Regional Forecasting of Coronavirus (Covid-19) Spread using a Deep Learning Model. Cem Direkoglu, Melike Sah. medRxiv 2020.05.23.20111039; doi:<https://doi.org/10.1101/2020.05.23.20111039>

564. Worldwide case fatality ratio of covid-19 over time. Rohan Chaubal, Sadhana Kannan, Navin Khattry, Sudeep Gupta. medRxiv 2020.10.04.20206599; doi:<https://doi.org/10.1101/2020.10.04.20206599>

565. Years of life lost due to the psychosocial consequences of COVID19 mitigation strategies based on Swiss data. Dominik A. Moser, Jennifer Glaus, Sophia Frangou, Daniel S. Schechter. medRxiv 2020.04.17.20069716; doi:<https://doi.org/10.1101/2020.04.17.20069716>

566. Yuliya Kyrychko, Konstantin Blyuss, Igor Brovchenko. medRxiv 2020.07.24.20161497; doi:<https://doi.org/10.1101/2020.07.24.20161497>

## Grey literature n=20

1.   Fohm Rapport över möjligt scenario i Sverige baserat på utvecklingen i Wuhan. <https://www.folkhalsomyndigheten.se/contentassets/4b4dd8c7e15d48d2be744248794d1438/vardbehov-scenarier-wuhan-grafer-2020-03-nn.pdf>

2.   Fohm Rapport Uppdatering av modeller baserat på Wuhan, med tillhörande uppdaterad metodbeskrivning.<https://www.folkhalsomyndigheten.se/contentassets/1887947af0524fd8b2c6fa71e0332a87/skattning-av-vardplatsbehov-folkhalsomyndigheten.pdf>

3.   Fohm Rapport Möjligt scenario i Sverige baserat på utvecklingen i Lombardiet, Italien.<https://www.folkhalsomyndigheten.se/contentassets/4b4dd8c7e15d48d2be744248794d1438/skattning-av-behov-av-slutenvardsplatser-covid-lombardiet.pdf>

4.   2020-05-14 uppdaterar FHM sina underlag till regionerna och lägger till nya värsta scenarion. Nu baseras scenarion på svenska data, och inte på data ifrån Wuhan och Lombardiet. <https://www.folkhalsomyndigheten.se/contentassets/4b4dd8c7e15d48d2be744248794d1438/vardbehov-scenarier-vardbelastning-baserat-svenska-data-20200514.pdf>

5.   Den 2020-04-21 publicerar FHM en rapport över Skattning av peakdag och antal infekterade i covid-19-utbrottet i Stockholms län februari-april 2020.<https://www.folkhalsomyndigheten.se/publicerat-material/publikationsarkiv/s/skattning-av-peakdag-och-antal-infekterade-i-covid-19-utbrottet-i-stockholms-lan-februari-april-2020/>

6.   Den 2020-0701 utkom FHM med en uppdaterad version på rapport nr 5 ovan, nu även för Dalarna, Skåne, och Västra Götaland. [ttps://www.folkhalsomyndigheten.se/contentassets/e1702f53eea144cdb1ca2ef854b45c35/estimates-peak-day-infected-during-covid-19-outbreak-20103.pdf](https://www.folkhalsomyndigheten.se/contentassets/e1702f53eea144cdb1ca2ef854b45c35/estimates-peak-day-infected-during-covid-19-outbreak-20103.pdf)

7.   Den 2020-06-15 utkom FHM med en rapport över olika tänkbara scenarios fram till årsskiftet i Sveriges samtliga regioner beroende på kontaktintensiteten i regionerna. Modellerna tas fram genom VirSim. Huvudrapport: <https://www.folkhalsomyndigheten.se/contentassets/29b815266baa4b409905c096be773df5/effekter-okade-kontakter-okat-resande-sverige-sommaren-2020.pdf>

8.   Den 2020-08-26 presenterar FHM rapporten Scenarier – Tre smittspridningsscenarier inom regeringsuppdraget ”Plan inför eventuella nya utbrott av covid-19”<https://www.folkhalsomyndigheten.se/contentassets/de0d0ffc939f43c397a0de23c5aa9be6/scenarier-tre-smittspridningsscenarier-plan-eventuella-nya-utbrott-covid-19.pdf>

9.   Den 2020-10-22 publicerar FHM en rapport med scenarion specifikt för individer äldre än 70 år. <https://www.folkhalsomyndigheten.se/publicerat-material/publikationsarkiv/k/konsekvenser-for-personer-70-ar-och-aldre-av-smittskyddsatgarder-mot-covid-19/?pub=81272>

10.  Den 2020-12-21 presenterar FHM en uppdatering av rapport 8 ovan, inom regeringsuppdraget att löpande uppdatera scenarier för hur smittspridningen av det virus som orsakar sjukdomen som orsakar sjukdomen covid-19 kan komma att utvecklas framöver. <https://www.folkhalsomyndigheten.se/contentassets/fa087223be5c4bef8298ee2943f099ca/scenario-fortsatt-spridning.pdf>

11. Den 2020-12-30 publiceras nya prognoser av FHM med fokus på vårdbelastning. <https://www.folkhalsomyndigheten.se/contentassets/4b4dd8c7e15d48d2be744248794d1438/prognoser-inlaggningar-pa-sjukhusens-vanlig-vardavdelningar-vecka-53.pdf>

12. Den 2020-12-29 kom också FHM ut med en rapport som syftar till att skatta antalet nya fall per dag i regionerna. <https://www.folkhalsomyndigheten.se/contentassets/4b4dd8c7e15d48d2be744248794d1438/trendanalys_region_2020-12-29.pdf>

13. Den 2020-09 publicerar socialstyrelsen en rapport över planering i de olika regionerna. dessa är helt baserade på de rapporter FHM tagit fram tidigare. <https://www.socialstyrelsen.se/globalassets/sharepoint-dokument/artikelkatalog/ovrigt/2020-9-6886.pdf>

14. Den 2020-06 publicerar MSB en rapport med tre möjliga scenarion. <https://www.msb.se/contentassets/16bfbd5b4edb4e7bb3a8e2339002e959/scenarier-som-forstarkning-till-befintlig-analys-och-planering-msb1594.pdf>

15. Den 2020-09-17 publiceras projektioner över vårdbehov i EU <https://www.ecdc.europa.eu/sites/default/files/documents/ECDC-30-day-projections-Sept-2020.pdf>

16. Den 2020-11-23 kom en uppdatering av dessa projektioner. <https://www.ecdc.europa.eu/sites/default/files/documents/covid-forecasts-modelling-november-2020.pdf>

17.  Den 2020-04-13 publicerar ECDC en rapport över lämplig övervakningsstrategi i syfte att förbereda vården. <https://www.ecdc.europa.eu/sites/default/files/documents/COVID-19-surveillance-strategy-9-Apr-2020.pdf>

18.  Den 2020-05-17 publicerar ECDC en rapport om hur medlemsländer bör sätta upp uppföljningsstrategier för covid-19. <https://www.ecdc.europa.eu/sites/default/files/documents/covid-19-long-term-care-facilities-surveillance-guidance.pdf>

19.  Den 2020-01-11 publicera ECDC en rapport över egen utvärdering av framtagande av (prognos) underlag till medlemsländerna covid-19, 2020. <https://www.ecdc.europa.eu/en/publications-data/strategic-and-performance-analysis-ecdc-response-covid-19-pandemic>

20.  Den 26/5 baseline projections ECDC. <https://www.ecdc.europa.eu/sites/default/files/documents/Projected-baselines-COVID-19-for-assessing-impact-measures.pdf>
